# In silico Phylogenetic Analysis of *hAT* Transposable Elements in Plants

**DOI:** 10.3390/genes9060284

**Published:** 2018-06-06

**Authors:** Gökhan Karakülah, Athanasia Pavlopoulou

**Affiliations:** 1 Izmir International Biomedicine and Genome Institute, Dokuz Eylül University, Izmir 35340, Turkey; gokhan.karakulah@deu.edu.tr; 2 Izmir International Biomedicine and Genome Center (IBG), Izmir 35340, Turkey

**Keywords:** *hAT* transposable elements, plants, phylogeny, terminal inverted repeats, transposase

## Abstract

Transposable elements of the *hAT* family exhibit a cross-kingdom distribution. The plant *hAT* transposons are proposed to play a critical role in plant adaptive evolution and DNA damage repair. The sequencing of an increasing number of plant genomes has facilitated the discovery of a plethora of *hAT* elements. This enabled us to perform an in-depth phylogenetic analysis of consensus *hAT* sequences in the fully-sequenced genomes of 11 plant species that represent diverse taxonomic divisions. Four putative nucleotide sequences were detected in cottonwood that were similar to the corresponding animal *hAT* elements, which are possibly sequence artifacts. Phylogenetic trees were constructed based both on the known and putative *hAT* sequences, by employing two different methods of phylogenetic inference. On the basis of the reconstructed phylogeny, plant *hAT* elements have rather evolved through kingdom-specific vertical gene transfer and gene amplifications within eudicotyledons, monocotyledons, and chlorophytes. Furthermore, the plant *hAT* sequences were searched for conserved DNA and amino acid sequence features. In this way, diagnostic sequence patterns were detected which allowed us to assign functional annotations to the plant *hAT* sequences.

## 1. Introduction

Transposable elements (TEs), or “jumping genes”, are mobile genetic elements capable of moving (transposing) from one location to another in the genome. The first elements, Activator (Ac) and Dissociation (Ds) were identified in maize (*Zea mays*) by McClintock [1]. Transposons are ubiquitous and fairly abundant in the genomes of eukaryotic and prokaryotic organisms, occupying over two-thirds of the human genome [2,3] and a significant fraction of some plant genomes [4,5,6].

Transposable elements can be broadly classified into two major classes [7] on the basis of their transposition mechanism, that is, retrotransposons, which transpose by means of a ‘copy-and-paste’ mechanism (Class I) and DNA transposons (Class II), which transpose directly, without involving the reverse transcription of an RNA intermediate.

The *hAT* family of Class II transposons, named after the three prototypic members, the maize transposon Activator (Ac), the *Drosophila melanogaster* element *hobo* and the *Antirrhinum majus* (napdragon) *Tam3* transposon [8], transpose via a ‘cut-and-paste’ mechanism. The *hAT* transposons (i) require a TE-encoded transposase enzyme for insertion and excision; (ii) are flanked on both of their ends by terminal inverted repeats (TIRs), with a minimum length of 8 base pairs; and (iii) generate 4–8 bp target-site duplications upon insertion [9,10]. Members of the *hAT* family display a wide phylogenetic distribution across fungi, plants, and animals [11,12].

Transposable elements can be further divided into the autonomous elements, which encode the cellular machinery required to move independently, and the nonautonomous elements, which require the cellular apparatus of other autonomous TEs for their mobilization. For example, the nonautonomous element *Ds* depends on *Ac* to transpose (Ac/Ds TE system) [13].

In plants, transposon activity can dramatically affect the overall plant gene expression, structure, and function, often resulting in phenotypic changes, thereby contributing largely to adaptive plant evolution [14,15,16]. Of importance, transposon mobilization poses a major threat to the host genome by generating various types of DNA damage, including single-base mismatches and double-strand breaks (DBS). These transposition-inflicted DNA lesions lead to the activation of DNA damage repair pathways [17,18].

Despite the important role of the *hAT* family in the evolution of plants, to our knowledge, an in-depth study on the phylogeny of plant *hAT* elements is lacking. Previous phylogenetic studies of the plant *hAT* TEs, due to the scarcity of fully sequenced genomes, were restricted to few transposon sequences [11,12]. The current availability of completely sequenced and well-annotated plant genomes allowed us to perform a detailed phylogenetic analysis of the plant *hAT* transposons. To this end, the genomes of 11 plant species, representing diverse taxonomic divisions, including vascular plants, mosses, and green algae (Figure S1), were investigated for *hAT* homologs. Phylogenetic trees were reconstructed based on plant *hAT* DNA sequences, alone or together with fungi and plant *hAT* sequences, by employing two different methods of phylogenetic inference. Moreover, the plant *hAT* sequences were investigated for transposase amino acid sequences, as well as TIR sequence patterns, in order to define diagnostic sequence signatures that could be used to predict the functionality of these elements.

## 2. Methods

### 2.1. Sequence Dataset

Collectively 272 consensus *hAT* DNA sequences of 10 Viridiplantae species were downloaded from the Repbase Update, a database of eukaryotic transposable elements [7,19], in October 2017 in FASTA format (Text S1). However, there are currently no *hAT* sequences available in Repbase for *Physcomitrella patens*. The scientific names of those species, along with their generic names and RefSeq Genome project accession codes (shown within parentheses, respectively), are as follows: *Oryza sativa* (rice; PRJNA122), *Zea mays* (maize; PRJNA249074), *Triticum aestivum* (wheat; PRJNA392179), *Hordeum vulgare* (barley; PRJEB13020), *Arabidopsis thaliana* (PRJNA10719), *Nicotiana tabacum* (tobacco; PRJNA208209), *Medicago truncatula* (barrel medic; PRJNA10791), *Populus trichocarpa* (black cottonwood; PRJNA17973), *Chlamydomonas reinhardtii* (PRJNA21061), and *Volvox carteri* (PRJNA50441) (Figure S1). Moreover, a total of 221 *hAT* sequences from diverse fungal and metazoan taxonomic groups (Table S1) were retrieved from Repbase (Text S1) [7,19]. For convenience, all *hAT* sequences under study are commenced by their genus and species initial letters.

The retrieved plant, animal, and fungi *hAT* DNA sequences were used as ‘probes’ to search the genomes of the 11 plant organisms available in NCBI GenBank [20], by applying reciprocal BLASTn [21] with default parameters, in order to identify novel *hAT* sequences in plants. Each novel sequence was manually examined and only the sequence hits with an *E*-value lower than e^−10^ were accepted and subsequently used for iterative database searching until new hits could not be found. In this way, four novel *hAT* homologous sequences were detected in *P. trichocarpa*. In a similar manner, the plant *hAT* sequences were used as ‘seeds’ to parse the corresponding fungal and animal genomes.

### 2.2. Phylogenetic Analyses

The full-length consensus *hAT* nucleotide sequences were aligned by employing MAFFT v.7 [22]. The resulting multiple sequence alignments were manually edited with JalView 2.10.3 [23]. The trimmed alignments were subsequently used for reconstructing phylogenetic trees by two different methods, a neighbor-joining method as implemented in the software package MEGA version 7.0.26 [24], and a maximum-likelihood method as implemented in PhyML 3.0 [25]. ModelTest [26] was used to estimate the best-fit model of nucleotide substitution, that is, GTR + G. Bootstrap analyses (100 pseudo-replicates) were performed in order to evaluate the robustness of the inferred trees. Phylogenetic tree data were rendered with Dendroscope version 3.5.9 [27].

### 2.3. Detection of Putative Terminal Inverted Repeats

Ιn order to identify putative TIRs ≥ 8 bp in the plant *hAT* sequences, the corresponding entire DNA sequences of those elements were provided as input to TIRfinder [28], a software tool for detecting inverted repeats in Class II elements. For this purpose, the consensus “(T/C)A(A/G)NG” proposed by Rubin et al. [12] was used as reference; the maximum number of mismatches was set to 4 so as to increase sensitivity and detect more novel putative TIRs. Consensus TIR sequences were generated with EBI’s Mview [29].

### 2.4. Analysis of Transposase Amino Acid Sequences

The translated transposase amino acid sequences of the plant *hAT* DNA sequences were obtained from Repbase in October 2017 in EMBL data format. The retrieved sequences were queried against the InterPro [30] v. 66.0 protein signature database using InterProScan [31], in order to identify their constituent domains. In the case of the transposase protein sequence of a plant *hAT* element, which was not available in Repbase, its corresponding DNA sequence was translated in all six open reading frames (ORFs) using EBI’s EMBOSS Sixpack [32]. The predicted sequences were then compared to the known transposase sequences by alignment with PROMALS3D [33], using structural information from the resolved tertiary structure of the house fly Hermes *hAT* transposase (PDB ID: 4D1Q, chain A) [34], so as to improve alignment accuracy; their protein domain arrangement was examined using InterProScan [31]. 

Selected amino acid sequences, corresponding to “active” transposases (see Results) were aligned with PROMALS3D [33], as before, and their three-dimensional structure and function were further examined by employing Phyre2 [35]. Moreover, ungapped sequence blocks, representing highly conserved regions of proteins, were extracted from the multiple alignment with the usage of Utopia suite’s CINEMA alignment editor [36] and submitted to WebLogo3 [37] to generate consensus amino acid sequences. 

## 3. Results

### 3.1. Homologous hAT Sequences in Plants

A total of 276 plant *hAT* nucleotide sequences, including the four putative ones, were investigated in this study. Despite extensive homology-based searches, no moss (*Physcomitrella*) *hAT* homologous sequences were detected. Four putative *Populus trichocarpa hAT* homologous sequences were detected. These newly identified sequences were arbitrarily named Pt URR1L, Pt URR1aL, Pt Charlie3L, Pt Chap4L, (where “L” stands for “like”) by virtue of similarity to the *Xenopus tropicalis* sequences, Xt URR1, Xt URR1a, Xt Chap4a/4b, and Xt Charlie3, respectively (Table S2, Figure S2). Regarding these cottonwood sequences, they are vector-contaminated DNA sequences, based on the fact that they are in the same BAC clone ISB1-145J20 (Table S2). Of importance, the presence of sequence artifacts has an immense impact on the public databases, often resulting to misleading results and interpretation errors [38,39]. The number of *hAT* elements in the corresponding plant species is shown within parentheses: *Oryza sativa* (179), *Zea mays* (26), *Arabidopsis thaliana* (23), *Chlamydomonas reinhardtii* (13), *Volvox carteri* (11), *Populus trichocarpa* (9), *Medicago truncatula* (7), *Hordeum vulgare* (2), *Triticum aestivum* (1), and *Nicotiana tabacum* (1). Conversely, putative plant *hAT* sequences were not found in the fungal and animal genomes.

The members of the plant *hAT* family appear to vary greatly among individual organisms, both in abundance and length. In particular, the consensus rice *hAT* sequences appear to be the most prolific among plants, whereas a single consensus *hAT* element was identified in tobacco and wheat. Moreover, *C. reinhardtii* Gulliver is the longest consensus *hAT* element (7144 bp) and *O. sativa* DEBOAT (110 bp) the shortest one. 

### 3.2. Phylogenetic Reconstruction

**Figure 1:**
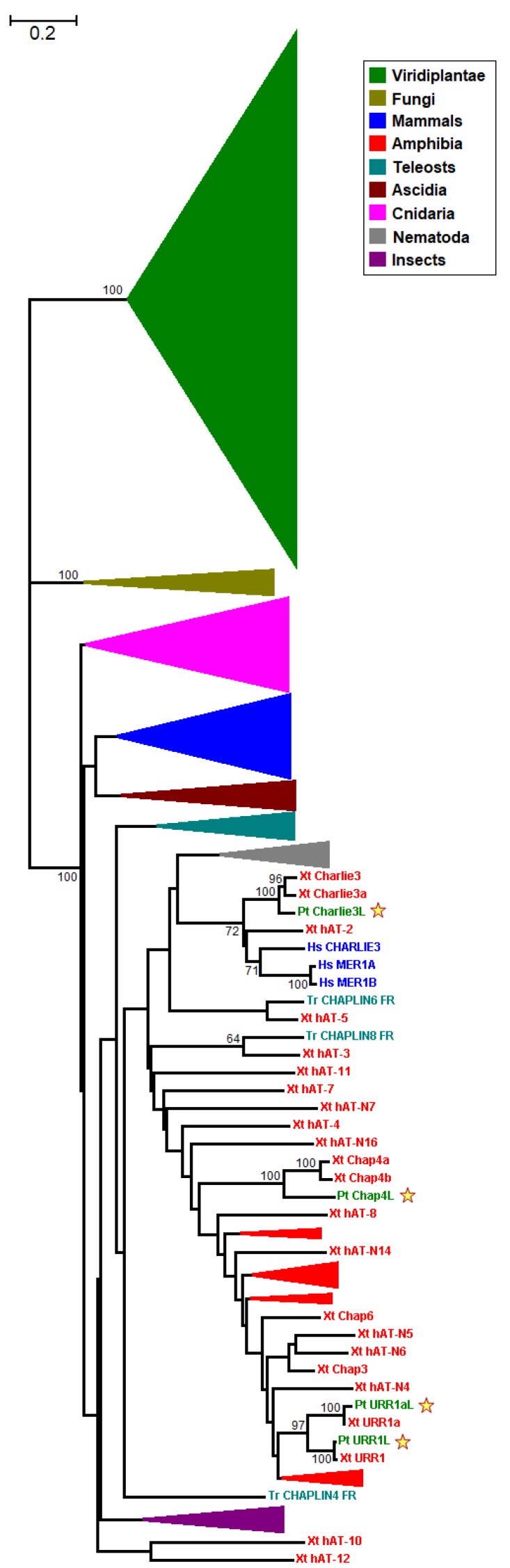
Unrooted neighbor-joining (NJ)-based tree of *hAT* transposon sequences. The major clades are collapsed for clarity. Taxa are represented by different coloring (inset). The four putative plant sequences are indicated by stars. The branch lengths depict evolutionary distance. Bootstrap values greater than 50% are shown at the nodes. The scale bar at the upper right indicates the length of nucleotide substitutions per site.

**Figure 2:**
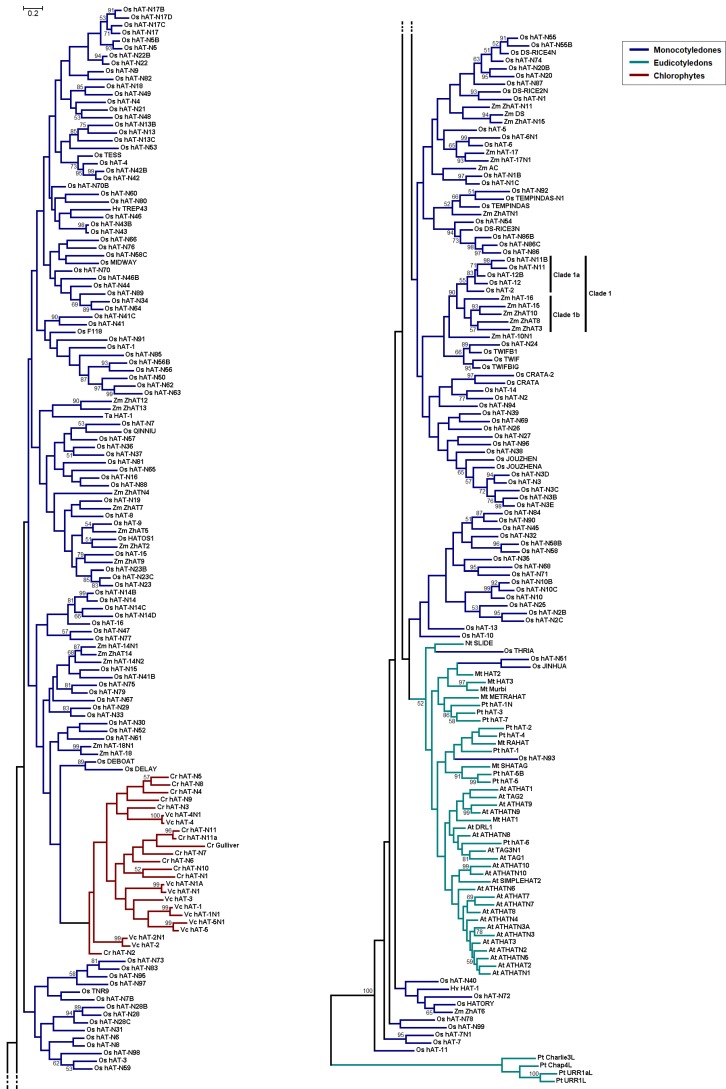
Unrooted maximum-likelihood (ML)-based tree of the plant *hAT* sequences. The tree is divided into two parts for clarity. Branch colors represent different taxonomic groups (insets). The conventions are the same as in Figure 1.

The entire length *hAT* nucleotide sequences were used in this study in order to extract as much information as possible from the individual sequences. To unravel the evolutionary relationships of *hAT* elements among kingdoms, fungal and metazoan *hAT* transposon sequences were also investigated along with their plant homologs. The neighbor-joining (NJ) method, which is based on a hierarchical clustering algorithm [40], was used to build an ‘interkingdom’ tree (Figure 1 and Figure S2). A maximum-likelihood (ML) method, a heuristic approach for finding the optimal tree that fits the observed data, was employed to assess the evolutionary relationships among plant *hAT* elements (Figure 2). The overall topology of the phylogenetic trees reconstructed with the NJ and ML methods is pretty congruent, especially in the major clades (Figure 2 and Figure S2). In both phylograms, three discrete, highly supported monophyletic clades are distinguished that correspond to the three kingdoms, suggesting that the emergence of *hAT* TEs followed the fungi–plant–animal divergence, according to the universal tree of life. 

The *hAT* sequences of the cereal plants (rice, maize, barley, and wheat) appear to cluster together. These elements have probably emerged after the monocotyledons-eudicotyledons divergence through monocot lineage-specific or species-specific gene expansion (Figure 2 and Figure S2). The consensus *O. sativa hAT* sequences appear to be the most abundant among the plant species under investigation, leading to the suggestion that multiple transposition events, mutations, and insertions/deletions might have taken place during the course of evolution that gave rise to the contemporary rice *hAT* elements. 

The well-supported Clade 1 in the trees, which was reconstructed with both methods, includes *hAT* sequences from *Oryza sativa* Clade 1a and *Zea mays* Clade 1b (Figure 2 and Figure S2). Clade 1 sequences might have been derived by an ancestral *hAT* sequence that existed before the rice-maize split (~70 million years ago [41]) through a series of species-specific gene duplications. 

The sequences Os hAT-N3/N3B/N3C/N3D/N3E, Os hAT-N10/N10B/N10C, Os hAT-N13/N13B/N13C, Os hAT-N17/N17B/N17C/N17D, Os hAT-N22/N22B, and Os hAT-N43/N43B, as well as Zm hAT-14/14N1/14N2 and Zm hAT-18/18N, form separate groups in both trees. Given that sequence duplications are very common in rice [42] and maize [43] genomes, the above sequences might reflect relatively recent duplication events that could have taken place in the corresponding species (Figure 2 and Figure S2). 

The eudicotyledonous plant (*Arabidopsis*, tobacco, barrel medic, and cottonwood) *hATs* form a distinct group, albeit moderately supported with 52% bootstrap support (Figure 2), suggesting propagation of *hAT* genes after eudicots branched off from monocots, approximately 200 million years ago (MYA) [44].

Of note, *Medicago truncatula* SHATAG clusters consistently, with high confidence, with the *P. trichocarpa* hAT5/5B (Figure 2 and Figure S2). These three sequences might have originated from a common ancestor before the separation of the orders Fabales and Malpighiales in flowering plants; intraspecies diversification followed.

In the reconstructed phylogeny (Figure 2 and Figure S2), the green algae (*Chlamydomonas* and *Volvox*) *hAT* elements appear to form a well-supported monophyletic group, indicating that *hAT* transposons might have evolved within the chlorophyte lineage independently of those in vascular plants. The Gulliver element in *C. reinhardtii* appears to have a long branch; probably due to the multiple mutations accumulated at this sequence over time. In *Volvox carteri*, Vc hAT-2/2N1, Vc hAT-4/4N1, and Vc hAT-N1/N1A are likely duplicate sequences with significant similarity, given their very short branch lengths. This could be also indicative of the immense contribution of sequence duplications to the evolution of transposable elements in *V. carteri*. 

The four novel *Populus trichocarpa hAT* sequences cluster with their orthologs in *Xenopus tropicalis* with 100% statistical confidence (Figure 1 and Figure S2). In the ML-based tree, the four ‘animal-like’ *P. trichocarpa hAT* sequences form their own well-supported group, very distantly related to the fellow plant *hAT* sequences (Figure 2). However, there is no evidence for horizontal gene transfer (HGT) events between *P. trichocarpa* and *X. tropicalis*. 

### 3.3. Terminal Inverted Repeats Patterns

**Figure 3:**
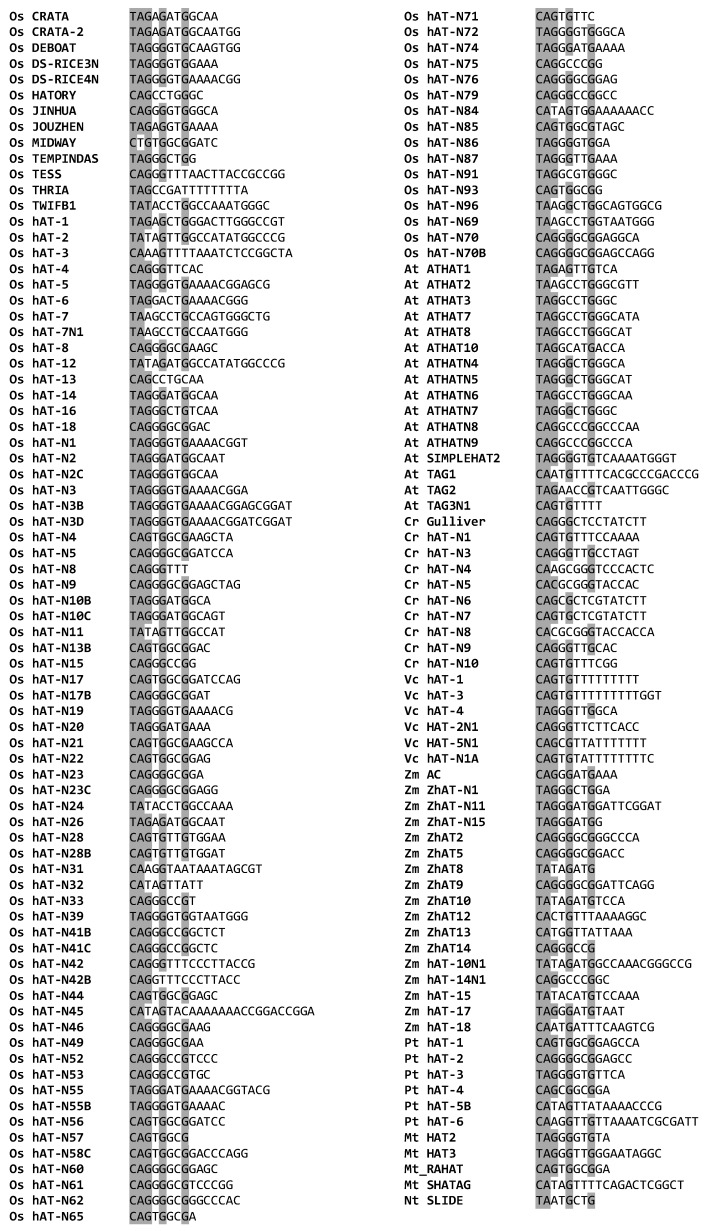
Alignment of the putative terminal inverted repeats (TIR) sequences flanking the plant *hAT* sequences. The conserved nucleotides are highlighted in grey.

The output TIR sequences were manually inspected and a total of 207 putative TIRs of 8 bp minimum length were retrieved (Table S3). To minimize redundancy, a set of 153 nonidentical TIRs (Figure 3), was selected in order to generate a consensus. A sequence pattern of eight nucleotides “[T/C]AGNGNNG” was deduced, where the letters within brackets indicate alternative nucleotides at this position. This consensus is a modification and extension of the consensus “[T/C]A[A/G]NG” suggested in a previous study by Rubin et al., (2011) [12]. Moreover, in the *hAT* TIR patterns of plants, the nucleotide ‘G’ occurs more frequently in the third and fifth position (Figure 3).

### 3.4. Transposase Conserved Structural Features

**Figure 4:**
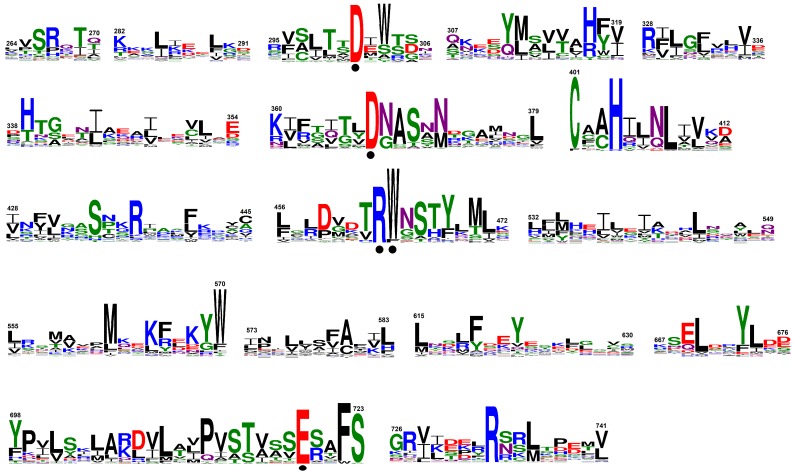
Conserved sequence blocks derived from the plant *hAT* transposases. The amino acid residues are numbered according to maize Activator (AC). The height of each letter depicts the frequency of the corresponding residues at that position, with the most frequent being on the top. The invariant amino acids essential for the activity of *hAT* transposases are indicated by dots.

Collectively, 69 transposase sequences were collected (Text S2). Of those, the sequences that contained the five amino acid key residues, D180, D248, R318, W319, and E572 (Hermes *hAT* transposase numbering; PDB ID: 4D1Q), essential for the function of transposases according to Hickman et al. [34] were subjected to Phyre2 [35] analysis (see Section 2.4). In this way, a total of 44 sequences, that harbored the five critical residues, were regarded as “active” transposases, whereas the sequences that lacked any of those residues were called “truncated” (Table S3). The “active” transposases consist of an N-terminal BED-type Zinc finger (138–199) [45], a site-specific DNA binding domain (227–271) [34], a catalytic domain with a long insertion (288–751) [34], and a highly conserved region (669–751) involved in dimerization in its C-terminus [34,46]; the numbers within the parentheses indicate the coordinates of each domain according to the maize Activator (AC) transposase sequence (Text S2). From the alignment of the “active” transposases, 17 conserved sequence blocks were identified (Figure 4). The five amino acids essential for the activity of *hAT* transposase enzymes [34] were also found unchanged in plant *hAT* transposases: D301, D367, R463, the aromatic residues [W/F] 464, and E719 (Figure 4). A series of highly conserved residues were also detected, apart from the known ones, including the conserved C401, H404, S723, and the invariant [F/W] 722 and R733 amino acids (Figure 4). These residues might also play an important role in maintaining the overall structure and function of plant *hAT* transposase enzymes.

### 3.5. Autonomous Elements

Nonautonomous elements, which are typically derived from autonomous TEs by internal deletion, do not contain the minimal sequences required for transposition, that is, an active-like transposase and TIR sequences [10]. Nonautonomous TEs (e.g., Os_TEMPINDAS) are often denoted by the addition of the letter ‘N’ into the name of their corresponding autonomous element (e.g., Os_TEMPINDAS-N1). Of note, in the reconstructed ML-based phylogram (Figure 2), several autonomous *hAT* elements appear to cluster with their corresponding nonautonomous derivatives with high confidence; for example, At ATHAT7/At ATHATN7, Zm ZhAT14/Zm hAT-14N1, Os hAT-6/Os hAT-6N1, and Vc hAT-4/Vc hAT-4N1. 

In our study, the *hAT* elements which were found to encode a candidate active transposase and also possess putative TIR sequences were considered as “autonomous.” In this way, a total of 35 potential autonomous plant *hAT* elements were identified (Table S3), representing 12.6% of the total elements. 

The founding member of the *hAT* family, Zm AC, is an autonomous element, as expected. The species, *Chlamydomonas* and *Volvox* are predicted to carry a single autonomous element, Cr Gulliver and Vc hAT-1, respectively. The cereal plants *Triticum* and *Hordeum*, although they encode transposases that contain the key amino acid residues required for their function, are probably not active elements since they lack putative TIRs (Table S3). 

It is also worth mentioning that the nonautonomous plant *hAT* elements, the number of which appears to markedly surpass the one of autonomous elements (Table S3), can potentially recruit the enzymatic machinery of the autonomous *hAT* elements for their transposition, such as the maize Ac/Ds TE system [10,13].

## 4. Conclusions

The members of the *hAT* family exhibit a wide distribution across all eukaryotic kingdoms. In the present study, the taxonomic distribution, evolution, and predicted functionality of the plant *hAT* family was investigated. Collectively, 276 nonredundant, concrete, plant *hAT* sequences were found (till October 2017). These sequences represent complete consensus *hAT* sequences [19] derived from each organism. However, there is a great discrepancy between the actual number of individual copies of *hAT* transposable elements in each species and the corresponding consensus sequences [47,48]. 

Phylogenetic analyses were performed with the full length of the *hAT* sequences so as to include all the available evolutionary information that is present in these sequences. Based on the reconstructed phylogeny, the *hAT* family of transposable elements has likely propagated in Viridiplantae through vertical gene transfer and subsequent gene proliferation within eudicotyledons, monocotyledons, and chlorophytes. The overall number of consensus *hAT* elements differs greatly among the individual plant genomes, ranging from a single consensus sequence in tobacco and wheat to 179 in rice, likely due to intraspecies gene duplications. 

In the transposases encoded by the plat *hAT* sequences, conserved protein segments and a series of sequentially invariant/conserved amino acid residues were identified. Furthermore, a TIR sequence pattern “(T/C)AGNGNNG” was also defined. The transposase protein blocks and characteristic amino acid residues, as well as the TIR pattern, could be used as diagnostic signatures for the identification of other plant *hAT* sequences. In our study, the elements that were found to encode a putative, likely active, transposase flanked by TIRs were referred to as “autonomous”. A total of 35 candidate autonomous elements were detected which, apparently, represent a small fraction of plant *hAT* elements. These likely active *hAT* elements can be co-opted by their host plant organisms, through an evolutionary process called ‘molecular domestication’ or ‘exaptation’ to carry out functions beneficial to the host [49,50]. 

Notably, four putative plant sequences of enigmatic origin were detected in cottonwood that shared a high degree of nucleotide identity with the amphibian *hAT* elements. These four animal-like *hAT* sequences which are likely nonautonomous, as they do not possess either a transposase-like sequence or TIRs, are probably sequence artifacts.

## Supplementary Materials

The following are available online at http://www.mdpi.com/2073-4425/9/6/284/s1. Text S1. Sequences analyzed in the present study in FASTA format. Text S2. Transposase amino acid sequences analyzed in the present study in FASTA format. Figure S1. A cladogram derived from NCBI’s Taxonomy database [51] showing the evolutionary relationships among plant species. Figure S2. Full view of the tree shown in Figure 1. The branches, the length of which reflects evolutionary distance, are colored according to the plant taxonomic group. Bootstrap values greater than 50% are shown at the nodes. The scale bar at the upper right indicates the length of nucleotide substitutions per position. Table S1. Taxonomy of the animal and fungi species under investigation based on NCBI’s Taxonomy database [51]. The abbreviated names of the species investigated in this study are shown within parentheses. Table S2 Accession numbers of the putative *Populus trichocarpa hAT* sequences. Table S3. The plant *hAT* sequences along with their putative TIRs and encoded transposases. The sequences indicated by red are the ones with identical TIRs that were not included in Figure 3. The elements that contain both a putative TIR and an active-like transposase are referred to as autonomous.
