# Supplementary material for: In silico Phylogenetic Analysis of hAT Transposable Elements in Plants
Source: Genes (Basel). 2018 Jun 6;9(6):284. doi: 10.3390/genes9060284 (PMC6027215; doi:10.3390/genes9060284)
Supplement: Supplementary file 1 [file genes-09-00284-s001.zip › TableS1.docx]

| **1. Metazoa** |
| --- |
| **1.1. Bilateria** |
| **1.1.1. Deuterostomia** |
| **1.1.1.1. Craniata** |
| **1.1.1.1.1. Sarcopterygii** |
| **1.1.1.1.1.1. Amniota** |
| **1.1.1.1.1.1.1. Mammalia** |
| *Homo sapiens* (Hs) |
| **1.1.1.1.1.2. Amphibia** |
| *Xenopus tropicalis* (Xt) |
| **1.1.1.1.2. Actinopterygii** |
| **1.1.1.1.2.1. Teleosts** |
| *Takifugu rubripes* (Tr) |
| **1.1.1.2. Ascidia** |
| *Ciona intestinalis* (Ci) |
| **1.1.2. Protostomia** |
| **1.1.2.1. Nematoda** |
| *Caenorhabditis elegans* (Ce) |
| **1.1.2.2. Arthropoda** |
| *Drosophila melanogaster* (Dm) |
| *Anopheles gambiae* (Ag) |
| **1.2. Cnidaria** |
| *Nematostella vectensis* (Nv) |
| **2. Fungi** |
| **2.1. Ascomycota** |
| *Ascobolus immersus* (Ai) |
| *Aspergillus nidulans* (An) |
| *Fusarium oxysporum* (Fo) |
| **2.2. Basidiomycota** |
| *Agaricus bisporus* (Ab) |
| *Laccaria bicolor* (Lb) |
| *Puccinia striiformis* f. sp. Tritici (Pst) |
