# Supplementary material for: In silico Phylogenetic Analysis of hAT Transposable Elements in Plants
Source: Genes (Basel). 2018 Jun 6;9(6):284. doi: 10.3390/genes9060284 (PMC6027215; doi:10.3390/genes9060284)
Supplement: Supplementary file 1 [file genes-09-00284-s001.zip › TableS2.docx]

| **Sequence** | **GenBank ID** | **Description** | **Range** |
| --- | --- | --- | --- |
| Pt URR1L | AC175581.2 | Populus trichocarpa clone ISB1-145J20, complete sequence | 29828 to 30013 |
| Pt URR1aL | AC175581.2 | Populus trichocarpa clone ISB1-145J20, complete sequence | 18891 to 19338 |
| Pt Charlie3L | AC175581.2 | Populus trichocarpa clone ISB1-145J20, complete sequence | 43537 to 43975 |
| Pt Chap4L | AC175581.2 | Populus trichocarpa clone ISB1-145J20, complete sequence | 11397 to 12581 |
