# Supplementary material for: In silico Phylogenetic Analysis of hAT Transposable Elements in Plants
Source: Genes (Basel). 2018 Jun 6;9(6):284. doi: 10.3390/genes9060284 (PMC6027215; doi:10.3390/genes9060284)
Supplement: Supplementary file 1 [file genes-09-00284-s001.zip › TableS3.docx]

| **Os CRATA** | TAGAGATGGCAA | Active TS | Autonomous |
| --- | --- | --- | --- |
| **Os CRATA-2** | TAGAGATGGCAATGG |  |  |
| **Os DEBOAT** | TAGGGGTGCAAGTGG |  |  |
| **Os DELAY** | TAGAGATGGCAATGG |  |  |
| **Os DS-RICE2N** | TAGGGGTGAAAACGGT |  |  |
| **Os DS-RICE3N** | TAGGGGTGGAAA |  |  |
| **Os DS-RICE4N** | TAGGGGTGAAAACGG |  |  |
| **Os F118** |  |  |  |
| **Os HATORY** | CAGCCTGGGC | Truncated TS |  |
| **Os HATOS1** |  | Active TS |  |
| **Os JINHUA** | CAGGGGTGGGCA |  |  |
| **Os JOUZHEN** | TAGAGGTGAAAA |  |  |
| **Os JOUZHENA** |  |  |  |
| **Os MIDWAY** | CTGTGGCGGATC |  |  |
| **Os QINNIU** | CAGTGGCGGAGCCA |  |  |
| **Os TEMPINDAS** | TAGGGCTGG | Active TS | Autonomous |
| **Os TEMPINDAS-N1** |  |  |  |
| **Os TESS** | CAGGGTTTAACTTACCGCCGG |  |  |
| **Os THRIA** | TAGCCGATTTTTTTTA |  |  |
| **Os TNR9** | CAGTGGCGGA |  |  |
| **Os TWIF** |  |  |  |
| **Os TWIFB1** | TATACCTGGCCAAATGGGC | Active TS | Autonomous |
| **Os hAT-1** | TAGAGCTGGGACTTGGGCCGT | Active TS | Autonomous |
| **Os hAT-2** | TATAGTTGGCCATATGGCCCG | Active TS | Autonomous |
| **Os hAT-3** | CAAAGTTTTAAATCTCCGGCTA | Truncated TS |  |
| **Os hAT-4** | CAGGGTTCAC | Active TS | Autonomous |
| **Os hAT-5** | TAGGGGTGAAAACGGAGCG | Truncated TS |  |
| **Os hAT-6** | TAGGACTGAAAACGGG | Truncated TS |  |
| **Os hAT-6N1** |  |  |  |
| **Os hAT-7** | TAAGCCTGCCAGTGGGCTG | Active TS | Autonomous |
| **Os hAT-7N1** | TAAGCCTGCCAATGGG |  |  |
| **Os hAT-8** | CAGGGGCGAAGC | Active TS | Autonomous |
| **Os hAT-9** |  | Active TS |  |
| **Os hAT-10** |  | Active TS |  |
| **Os hAT-11** |  | Truncated TS |  |
| **Os hAT-12** | TATAGATGGCCATATGGCCCG | Active TS | Autonomous |
| **Os hAT-12B** |  | Active TS |  |
| **Os hAT-13** | CAGCCTGCAA | Truncated TS |  |
| **Os hAT-14** | TAGGGATGGCAA | Active TS | Autonomous |
| **Os hAT-15** |  | Active TS |  |
| **Os hAT-16** | TAGGGCTGTCAA | Active TS | Autonomous |
| **Os hAT-18** | CAGGGGCGGAC | Truncated TS |  |
| **Os hAT-N1** | TAGGGGTGAAAACGGT |  |  |
| **Os hAT-N1B** | TAGGGGTGAAAACG |  |  |
| **Os hAT-N1C** | TAGGGGTGAAAACGGT |  |  |
| **Os hAT-N2** | TAGGGATGGCAAT |  |  |
| **Os hAT-N2B** |  |  |  |
| **Os hAT-N2C** | TAGGGGTGGCAA |  |  |
| **Os hAT-N3** | TAGGGGTGAAAACGGA |  |  |
| **Os hAT-N3B** | TAGGGGTGAAAACGGAGCGGAT |  |  |
| **Os hAT-N3C** | TAGGGGTGAAAACGGAGCGGAT |  |  |
| **Os hAT-N3D** | TAGGGGTGAAAACGGATCGGAT |  |  |
| **Os hAT-N3E** | TAGGGGTGAAAACGGAGCGGAT |  |  |
| **Os hAT-N4** | CAGTGGCGAAGCTA |  |  |
| **Os hAT-N5** | CAGGGGCGGATCCA |  |  |
| **Os hAT-N6** | CAGGGTTT |  |  |
| **Os hAT-N7** |  |  |  |
| **Os hAT-N7B** |  |  |  |
| **Os hAT-N8** | CAGGGTTT |  |  |
| **Os hAT-N9** | CAGGGGCGGAGCTAG |  |  |
| **Os hAT-N10** | TAGGGATGGCAAT |  |  |
| **Os hAT-N10B** | TAGGGATGGCA |  |  |
| **Os hAT-N10C** | TAGGGATGGCAGT |  |  |
| **Os hAT-N11** | TATAGTTGGCCAT |  |  |
| **Os hAT-N11B** | TATAGATGGCCATATGGCCCG |  |  |
| **Os hAT-N13** | CAGTGGCGGA |  |  |
| **Os hAT-N13B** | CAGTGGCGGAC |  |  |
| **Os hAT-N13C** |  |  |  |
| **Os hAT-N14** | TAGGGCTGTCAA |  |  |
| **Os hAT-N14B** | TAGGGCTGTCAA |  |  |
| **Os hAT-N14C** | TAGGGCTGTCAA |  |  |
| **Os hAT-N14D** | TAGGGCTGTCAA |  |  |
| **Os hAT-N15** | CAGGGCCGG |  |  |
| **Os hAT-N16** |  |  |  |
| **Os hAT-N17** | CAGTGGCGGATCCAG |  |  |
| **Os hAT-N17B** | CAGGGGCGGAT |  |  |
| **Os hAT-N17C** | CAGGGGCGGATCCA |  |  |
| **Os hAT-N17D** | CAGGGGCGGAT |  |  |
| **Os hAT-N18** |  |  |  |
| **Os hAT-N19** | TAGGGGTGAAAACG |  |  |
| **Os hAT-N20** | TAGGGATGAAA |  |  |
| **Os hAT-N20B** | TAGGGATGAAA |  |  |
| **Os hAT-N21** | CAGTGGCGAAGCCA |  |  |
| **Os hAT-N22** | CAGTGGCGGAG |  |  |
| **Os hAT-N22B** | CAGTGGCGGAG |  |  |
| **Os hAT-N23** | CAGGGGCGGA |  |  |
| **Os hAT-N23B** | CAGGGGCGGAG |  |  |
| **Os hAT-N23C** | CAGGGGCGGAGG |  |  |
| **Os hAT-N24** | TATACCTGGCCAAA |  |  |
| **Os hAT-N25** | TAGGGATGGCAAT |  |  |
| **Os hAT-N26** | TAGAGATGGCAAT |  |  |
| **Os hAT-N27** |  |  |  |
| **Os hAT-N28** | CAGTGTTGTGGAA |  |  |
| **Os hAT-N28B** | CAGTGTTGTGGAT |  |  |
| **Os hAT-N28C** | CAGTGTTGTGGAA |  |  |
| **Os hAT-N29** |  |  |  |
| **Os hAT-N30** | CAGGGCCGG |  |  |
| **Os hAT-N31** | CAAGGTAATAAATAGCGT |  |  |
| **Os hAT-N32** | CATAGTTATT |  |  |
| **Os hAT-N33** | CAGGGCCGT |  |  |
| **Os hAT-N34** | CAGTGGCGGA |  |  |
| **Os hAT-N35** |  |  |  |
| **Os hAT-N36** | CAGGGGCGGA |  |  |
| **Os hAT-N37** | CAGGGGCGGA |  |  |
| **Os hAT-N38** |  |  |  |
| **Os hAT-N39** | TAGGGGTGGTAATGGG |  |  |
| **Os hAT-N40** |  |  |  |
| **Os hAT-N41** |  |  |  |
| **Os hAT-N41B** | CAGGGCCGGCTCT |  |  |
| **Os hAT-N41C** | CAGGGCCGGCTC |  |  |
| **Os hAT-N42** | CAGGGTTTCCCTTACCG |  |  |
| **Os hAT-N42B** | CAGGTTTCCCTTACC |  |  |
| **Os hAT-N43** | CAGGGGCGGA |  |  |
| **Os hAT-N43B** |  |  |  |
| **Os hAT-N44** | CAGTGGCGGAGC |  |  |
| **Os hAT-N45** | CATAGTACAAAAAAACCGGACCGGA |  |  |
| **Os hAT-N46** | CAGGGGCGAAG |  |  |
| **Os hAT-N46B** | CAGGGGCGAAG |  |  |
| **Os hAT-N47** | AGGGGCGGATCCAG |  |  |
| **Os hAT-N48** | CAGTGGCGAAGCCA |  |  |
| **Os hAT-N49** | CAGGGGCGAA |  |  |
| **Os hAT-N50** | CAGGGGCGGGCCCA |  |  |
| **Os hAT-N51** |  |  |  |
| **Os hAT-N52** | CAGGGCCGTCCC |  |  |
| **Os hAT-N53** | CAGGGCCGTGC |  |  |
| **Os hAT-N54** |  |  |  |
| **Os hAT-N55** | TAGGGATGAAAACGGTACG |  |  |
| **Os hAT-N55B** | TAGGGGTGAAAAC |  |  |
| **Os hAT-N56** | CAGTGGCGGATCC |  |  |
| **Os hAT-N56B** |  |  |  |
| **Os hAT-N57** | CAGTGGCG |  |  |
| **Os hAT-N58** |  |  |  |
| **Os hAT-N58B** |  |  |  |
| **Os hAT-N58C** | CAGTGGCGGACCCAGG |  |  |
| **Os hAT-N59** |  |  |  |
| **Os hAT-N5B** | CAGGGGCGGATCCA |  |  |
| **Os hAT-N60** | CAGGGGCGGAGC |  |  |
| **Os hAT-N61** | CAGGGGCGTCCCGG |  |  |
| **Os hAT-N62** | CAGGGGCGGGCCCAC |  |  |
| **Os hAT-N63** | CAGGGGCGGGCCCA |  |  |
| **Os hAT-N64** |  |  |  |
| **Os hAT-N65** | CAGTGGCGA |  |  |
| **Os hAT-N66** | CAGTGGCGGA |  |  |
| **Os hAT-N67** | CAGGGCCGG |  |  |
| **Os hAT-N68** |  |  |  |
| **Os hAT-N69** | TAAGCCTGGTAATGGG |  |  |
| **Os hAT-N70** | CAGGGGCGGAGGCA |  |  |
| **Os hAT-N70B** | CAGGGGCGGAGCCAGG |  |  |
| **Os hAT-N71** | CAGTGTTC |  |  |
| **Os hAT-N72** | TAGGGGTGGGCA |  |  |
| **Os hAT-N73** |  |  |  |
| **Os hAT-N74** | TAGGGATGAAAA |  |  |
| **Os hAT-N75** | CAGGCCCGG |  |  |
| **Os hAT-N76** | CAGGGGCGGAG |  |  |
| **Os hAT-N77** |  |  |  |
| **Os hAT-N78** |  |  |  |
| **Os hAT-N79** | CAGGGCCGGCC |  |  |
| **Os hAT-N80** | CAGGGGCGAAGC |  |  |
| **Os hAT-N81** | CAGGGGCGGAGG |  |  |
| **Os hAT-N82** |  |  |  |
| **Os hAT-N83** |  |  |  |
| **Os hAT-N84** | CATAGTGGAAAAAACC |  |  |
| **Os hAT-N85** | CAGTGGCGTAGC |  |  |
| **Os hAT-N86** | TAGGGGTGGA |  |  |
| **Os hAT-N86B** | TAGGGGTGGA |  |  |
| **Os hAT-N86C** | TAGGGGTGGA |  |  |
| **Os hAT-N87** | TAGGGTTGAAA |  |  |
| **Os hAT-N88** |  |  |  |
| **Os hAT-N89** |  |  |  |
| **Os hAT-N90** |  |  |  |
| **Os hAT-N91** | TAGGCGTGGGC |  |  |
| **Os hAT-N92** | TAGGGGTGGA |  |  |
| **Os hAT-N93** | CAGTGGCGG |  |  |
| **Os hAT-N94** | TAGGGATGGCAA |  |  |
| **Os hAT-N95** |  |  |  |
| **Os hAT-N96** | TAAGGCTGGCAGTGGCG |  |  |
| **Os hAT-N97** |  |  |  |
| **Os hAT-N98** |  |  |  |
| **Os hAT-N99** |  |  |  |
| **At ATHAT1** | TAGAGTTGTCA | Truncated TS |  |
| **At ATHAT2** | TAAGCCTGGGCGTT | Active TS | Autonomous |
| **At ATHAT3** | TAGGCCTGGGC | Active TS | Autonomous |
| **At ATHAT7** | TAGGCCTGGGCATA | Active TS | Autonomous |
| **At ATHAT8** | TAGGCCTGGGCAT | Truncated TS |  |
| **At ATHAT9** |  | Truncated TS |  |
| **At ATHAT10** | TAGGCATGACCA | Active TS | Autonomous |
| **At ATHATN1** | TAGGCCTGGGC |  |  |
| **At ATHATN2** | TAGGCCTGGGCAT |  |  |
| **At ATHATN3** | TAGGCCTGGGC |  |  |
| **At ATHATN3A** | TAGGCCTGGGCAT |  |  |
| **At ATHATN4** | TAGGGCTGGGCA |  |  |
| **At ATHATN5** | TAGGGCTGGGCAT |  |  |
| **At ATHATN6** | TAGGCCTGGGCAA |  |  |
| **At ATHATN7** | TAGGGCTGGGC |  |  |
| **At ATHATN8** | CAGGCCCGGCCCAA |  |  |
| **At ATHATN9** | CAGGCCCGGCCCA |  |  |
| **At ATHATN10** | TAGGCATGACCA |  |  |
| **At DRL1** |  |  |  |
| **At SIMPLEHAT2** | TAGGGGTGTCAAAATGGGT |  |  |
| **At TAG1** | CAATGTTTTCACGCCCGACCCG |  |  |
| **At TAG2** | TAGAACCGTCAATTGGGC | Active TS | Autonomous |
| **At TAG3N1** | CAGTGTTTT |  |  |
| **Cr Gulliver** | CAGGGCTCCTATCTT | Active TS | Autonomous |
| **Cr hAT-N1** | CAGTGTTTCCAAAA |  |  |
| **Cr hAT-N2** |  |  |  |
| **Cr hAT-N3** | CAGGGTTGCCTAGT |  |  |
| **Cr hAT-N4** | CAAGCGGGTCCCACTC |  |  |
| **Cr hAT-N5** | CACGCGGGTACCAC |  |  |
| **Cr hAT-N6** | CAGCGCTCGTATCTT |  |  |
| **Cr hAT-N7** | CAGTGCTCGTATCTT |  |  |
| **Cr hAT-N8** | CACGCGGGTACCACCA |  |  |
| **Cr hAT-N9** | CAGGGTTGCAC |  |  |
| **Cr hAT-N10** | CAGTGTTTCGG |  |  |
| **Cr hAT-N11** |  |  |  |
| **Cr hAT-N11a** | CAGTGCTCGTATCTT |  |  |
| **Vc hAT-1** | CAGTGTTTTTTTTT | Active TS | Autonomous |
| **Vc hAT-2** |  | Truncated TS |  |
| **Vc hAT-3** | CAGTGTTTTTTTTTGGT | Truncated TS |  |
| **Vc hAT-4** | TAGGGTTGGCA | Truncated TS |  |
| **Vc HAT-1N1** | CAGTGTTTTTTTTT |  |  |
| **Vc HAT-2N1** | CAGGGTTCTTCACC |  |  |
| **Vc HAT-4N1** | TAGGGTTGGCA |  |  |
| **Vc HAT-5** |  |  |  |
| **Vc HAT-5N1** | CAGCGTTATTTTTTT |  |  |
| **Vc hAT-N1** |  |  |  |
| **Vc hAT-N1A** | CAGTGTATTTTTTTTC |  |  |
| **Zm AC** | CAGGGATGAAA | Active TS | Autonomous |
| **Zm DS** |  |  |  |
| **Zm ZhAT-N1** | TAGGGCTGGA |  |  |
| **Zm ZhAT-N11** | TAGGGATGGATTCGGAT |  |  |
| **Zm ZhAT-N15** | TAGGGATGG |  |  |
| **Zm ZhAT2** | CAGGGGCGGGCCCA | Active TS | Autonomous |
| **Zm ZhAT3** |  | Active TS |  |
| **Zm ZhAT5** | CAGGGGCGGACC | Active TS | Autonomous |
| **Zm ZhAT6** | TAGGGCTGGGCA | Truncated TS |  |
| **Zm ZhAT7** |  | Truncated TS |  |
| **Zm ZhAT8** | TATAGATG | Active TS | Autonomous |
| **Zm ZhAT9** | CAGGGGCGGATTCAGG | Truncated TS |  |
| **Zm ZhAT10** | TATAGATGTCCA | Active TS | Autonomous |
| **Zm ZhAT12** | CACTGTTTAAAAGGC | Truncated TS |  |
| **Zm ZhAT13** | CATGGTTATTAAA | Truncated TS |  |
| **Zm ZhAT14** | CAGGGCCG | Active TS | Autonomous |
| **Zm ZhATN4** |  |  |  |
| **Zm hAT-10N1** | TATAGATGGCCAAACGGGCCG |  |  |
| **Zm hAT-14N1** | CAGGCCCGGC |  |  |
| **Zm hAT-14N2** |  |  |  |
| **Zm hAT-15** | TATACATGTCCAAA | Active TS | Autonomous |
| **Zm hAT-16** |  | Active TS |  |
| **Zm hAT-17** | TAGGGATGTAAT | Active TS | Autonomous |
| **Zm hAT-17N1** | TAGGGATGTAAT |  |  |
| **Zm hAT-18** | CAATGATTTCAAGTCG | Truncated TS |  |
| **Zm hAT-18N1** | CAATGATTTCAAGTCG |  |  |
| **Pt hAT-1** | CAGTGGCGGAGCCA | Active TS | Autonomous |
| **Pt hAT-1N** |  |  |  |
| **Pt hAT-2** | CAGGGGCGGAGCC | Active TS | Autonomous |
| **Pt hAT-3** | TAGGGGTGTTCA | Active TS | Autonomous |
| **Pt hAT-4** | CAGCGGCGGA | Active TS | Autonomous |
| **Pt hAT-5** |  | Truncated TS |  |
| **Pt hAT-5B** | CATAGTTATAAAACCCG | Truncated TS |  |
| **Pt hAT-6** | TCAAGGTTGTTAAAATCGCGATT | Truncated TS |  |
| **Pt hAT-7** | TAGGGGTGTTCA | Active TS | Autonomous |
| **Pt Chap4L** |  |  |  |
| **Pt Charlie3L** |  |  |  |
| **Pt URR1aL** |  |  |  |
| **Pt URR1L** |  |  |  |
| **Mt HAT1** |  | Truncated TS |  |
| **Mt HAT2** | TAGGGGTGTA | Active TS | Autonomous |
| **Mt HAT3** | TAGGGTTGGGAATAGGC | Active TS | Autonomous |
| **Mt METRAHAT** |  | Truncated TS |  |
| **Mt Murbi** |  |  |  |
| **Mt RAHAT** | CAGTGGCGGA | Active TS | Autonomous |
| **Mt SHATAG** | CATAGTTTTCAGACTCGGCT | Truncated TS |  |
| **Hv HAT-1** |  | Active TS |  |
| **Hv TREP43** |  |  |  |
| **Ta HAT-1** |  | Active TS |  |
| **Nt SLIDE** | TAATGCTG | Active TS | Autonomous |
