# Supplementary material for: In silico Phylogenetic Analysis of hAT Transposable Elements in Plants
Source: Genes (Basel). 2018 Jun 6;9(6):284. doi: 10.3390/genes9060284 (PMC6027215; doi:10.3390/genes9060284)
Supplement: Supplementary file 1 [file genes-09-00284-s001.zip › TextS1.docx]

>Cr_Gulliver Chlamydomonas reinhardtii

cagggctcctatcttaatgtctccagacattaaacggccattttggccattttccagacaaacggagggg

ggggttcacgcacgcttttgaacaaaacaagcggtgtctgaggagaggcaaactctaccatagtgacata

tatattttgtagaaagtgagggaatgtcatggtcctttaggagattttcggcgatatgacgaggatgaag

gacaccccgatcaagtcttcctttttgcagatgcgcagagccggtgacgaggatttgccggggatgccgc

attttcagtcacgcagagttgtcaggggtgcaaagcccgctgcaactccttccacagtccatggtccagc

taacccgcgcacagtgaagaaaagctgagacgatagaggtcaggaatgaacagaagtcagggatggaccc

cgtgcacgggcggaaccgtggagctgcggaggggttgaagaaaacaggcagggacgaggcgcgcgggaga

gggggtatacagcagccttgacagcagcttcgtattggagtgcactgaaccactcgcactcagggcgggg

ctgctgtcaagctcaaccatgctactcctccatgcgtaggggaatcaacaagaacgggacctgggaaagg

acctgggaaaggaccggggaaaggaccggggaaaggaccagggaattgaccgaggaatggggagccatca

cgggacattgactaggacacagtgatattaggcatgcaacaggcaacacattacattctgccgtcggcac

acaacaaatggagaagcggggcacaaaattaggagcaaacgataatgttttcgtgagtagctgctgggct

acagtgtcaatagtgttttagtgcatctggaagctgatccccgttcagcgacggggtcttcggagcagag

taccgaccgcttactgggtcggctgtaccggcccgtccggccggcgtgtggtgacgttctggtcttcttg

tggtcccggagcccgttggcccccgacgactcttcctcaacgtgacttttgcacgggtgctagaacggtc

ctctacgctgggcatgcatggggatttgggggtgggttcagctggcgccggattcccatctcaactagac

accgctcccgactagggggcaagttggcacgcggaggtggggaccgcatgctaccatagtgcgggatatg

atggtgcgggagatgatgttgttgcaaaccttgtactgctagggacgcatgcagggcgtcaagcattctg

gagggcgcgggcgatgcaagaggcatgggctggcattgccgggcgtgtgattttggacggttgcatgtgc

ggcctggtgggcagcggcttggaatgccctgtctgcggtttctccgcctgacaacatgcgtgtgtcatgg

caaaccttgtactgctagggacgcatgcagggcgtcaagcattctggagggcgcgggcgatgcaagaggc

atgggctggcattgccgggcgtgtgattttggacggttgcatgggcggcgtggtgggcagcggcttggaa

tgccctgtctgcggtttctccgcctgacaacatgcgtgtgtcatggcaaaccttgtactgctagggacgc

atgcagggcgtcaagcattctggagggcgcgggcgatgcaagaggcatgggctggcattgccgggcgtgt

gattttggacggttgcatgggcggcgtggtgggcagcggcttggaatgccctgtctgcggtttctccgcc

tgacaacatgcgtgtgtcatggcaaaccttgtactgctagggacgcatgcagggcgtcaagcattctgga

gggcgcgggcgatgcaagaggcatgggctggcattgctgggcgtgtgattttggacggttgcatgggcgg

cctggtgggcagcggcttggtagaggctncatncngcccccaccagcgactcccgaagcttccagcgctg

taatcacatggtaccatgtgtttgcgcgcgcgttctacacgccaggcataccgcatcaacctacaggccc

cgtgcggatgggcgcgccgcggccctgtccctgtttatggttcctgacattgcctgagcacccagtgccc

ctgccggcggcagaggctgcattccgcccccaccagcgactccgaagcttccagtgctgtaatcacatgg

tacaatgtgttggggcacgcgttctacacgccaggcataccgcatcaaccgacaggccccgtgcggatgg

gcgcgccgctgccctgtccctgcttacggctgccagcagcagtggctgcatacggcccacaccagcgact

ccccaagcttccagcgctgtaatcacatggtaccatgcgtttgcgcgcgcgttctacacgccaggcatac

cgcatcaacctacaggccccgtgcggatgggcgcgccactgccctgtccctgtttacggttcctgacatc

ggctgagcacccagtgcccctgccggcggcagaggctgcataccgcccccaccagcgactccgaagcttc

cagtgctgtaatcacatggtacaatgtgttggggcacgcgttctacacgccaggcataccgcatcaaccg

acaggccccgtgcggatgggcgcgccactgccctgtccctgtttacggttcctgacatcgcctgagcacc

cagtgcccctgccggcggcagaggctgcataccgcccccaccagtgactccccaagctttcagcgctgta

atcacatggtacaatgtgttggggcacgcgttctacacgccaggcataccgcatcaaccgacaagccccg

tgcggatgggcgcgccgcggagacactccacctgcttgcagattggaataatgaatcagtgcgtattgcg

aagttcgtcaacatcaagaacaccagccgttggaatgggatcgggcacaatttcgtcaggactcctgagt

gcacgttcttgtgccttgcctgcatgaagcaccccggtgcgaaggcctctgtttggaccaagggcggcaa

caaagtgagtgtgcttgcaaccatgtagcctggcaatgcagcgtgtgagccggattaggagagactaggg

gtgtgcgcatactgcacgcaagcaagcgcatgcactgcggcgcaccgtctggggtacccatttacagctg

cagcctgacagctaagacaggacctgctacccagccgcctccccgcatgcttgcgtaagcttgcaaatcc

cttgtgttctgctgcaggtcatgaacatgactttgttcaacaagcaccttctctccaagaaccatctgga

cgttataggcgcggctaagaaccacacgaagatgtccaacattgtcgagaagcagaaggtcaagctcaac

ccaacgctgtgcagcctaatccgttcggcttacgcctgcggcaaggatgccatggtgcgtttgtgagcgc

ggcgcgtgggcaattgtgcatggcgcacatgtgggcgcggcgcgcgtgcgtgctggtgtgtgttcgtttg

gtgtgttggtgtgtggtggtgtagcaacgtttgtggtgtgttggtgtgtggtggtgtagcaacgtttgtg

gcacatttggtaagcggtactagtgggcgtagtgtgctgtagtgtatgcatacggcatgtacaacaggca

tggcgttcggctgcatctttatcggctgacacgttacaacgtgcacccttcctgcttgctcgcagcccct

gacccactatgtgaagatggtcaaactccaggctgcgaactgcgctgactgcaagcatgactgcaaggcg

catacgtgccgaaaatgcgatgcgtccaaggcatgcggcagtggtatcactatttccggtccataccaca

ccgcggagaaagcgtcggaaatgctggcttgcctgtctgaggtaagacctgaacatatgcagctgtggcg

ggagcttacgggattgggttcgtttttggcgtgatggtatgccattgcttggatgctggtgctgtccttg

tatcataccctcgccaagtataccggtgcgcatgctaacgactgacaacccgcgatctgcgcaggcaatt

tcagaggaacaactcaagaacatcagggcatctcctgtcatctccatgatgattgatgagtctactgacc

gcactgtttcgcacaacctggccgtgtacattacatacgtggcgcccgatgacagcatcaagactgagtt

tctgcaactggaggcaatgaacaatggtgccacggccgtgaacatctatgacaggctcaaggaggtgttc

acggtgcgttcttaacagtgtgaacacgtggccttctcgagcagtagattgtgcggttacgcagggacag

atcatgcatgtagagccacggacgtgcggacagtgtcggctgtgtgtgctgagctgtgtgttctgctccc

ccgcgtgtgcctgctgcaaccaattacgtacaggagagcaagattgattggagtaagcttgtcgccttca

ccagtgacggtgccaacgttatggttgggaagcactgtggagtggcgacccgcatcaagacggattggcc

ctgcgtcctgactagccactgcgctgcgcaccgactggctctggcctgtgcggacttcttcaaggagttc

cctgccctggtcaaggtggacaacatgctctctaagatctacaactacgccaagacctcgacaggtgcgc

ttgggcgcgctagtggttgttgtgtggcgagttgtgagtgaatggcttctttgatgtattgctgtaactt

gtgcacaccgtgtcctacagtgcgaaccgctgctctgaacgacatgtacaaggaaaggaaggcgaaggca

tacaagatcctgaagccgcacacgtaagccgcacacgtcgtgccttgctacctgctgacatgtgcgctgg

tgtgtgggtgtaagtgtgtatgtgggtgtgtggacggcggtagcctagcctgtggagtagggtgagacgg

cttcacaagctgcgtgctacctgctgctgaggctgaggggttgatatacatcccgcttgcacactgcgca

aacttacgcagggttcggtggctttcccggtctgagtgtgtgaggcggatcaagattacctacccgatcc

tgcttgccttcttcaatgagcggaagaaggacaagaaggatgtggccgcggccgagatttacgagtggct

caggtgtgtggatgccctgggatgtcaggaatgcaagcgtgtgcaagtaggcgtgggcgcgcactcgtgc

tctagtgcgtatgtccgctgcatgtgtgtccgtgactgcgttcctgtcttggactaaaacgcgatcacta

ccgcatgctatttcacacctgcaggcaggtggacaacctcctgctcatcacctgcattgacggagttctg

gctgcaacagcggaattgagcaagtggttccagcagagtgatctcgccctcgttgacgtgcatcagtacc

tggaactagggctacggcacctgatcaacgcctacacctaccacaaggagggcgagacgtcgaacgcacc

tccatcctttacggcacccatccaggccctgatcaaggaccttgcagcgaaggatggcgtgttccacggc

caccagatggtgctgacaacccctcccacgacgatcgcgcctgccaccaccaccaccgccaccacgacag

cggagtctgacagcgacaatgacggctctgacaccaacactgacacgacagcggagtctggcagcgacga

tgatgacacggcacctacggcaccgagctgggcggctttccctcagactctattccgcagcaagcttgct

ctcagcagcatggtcaagaagctggttgagaacatcaaaatgcgattccccgctgatgtctcagtggctg

caaagtttggggtgctgggcccccgtgcattggctgctgattccggtgtgccgaagtatggcgaggaaga

ggtggccgctttggcacagcactttaagcccgtcttgggtgatgattgcctgttggcagttgaccagtgg

gtgatggctcgggcgcgcctgattgcagtggcaaaggagcagaagaagagtggtgatgttatgaaggcga

ggccgttctatgagaggctcttgtcgtggctgtctggcatgggccgtgagctcactgtcctggtgcagat

tatgcttgtgctgcagccatctacggcagaagttgagcgtggcttcagcgcgatgaatgacatcaagacg

cctggccgggcaagcatgaagcttggcacccttgacgtactgatgcgtgtgcgccttgtgggcccaccaa

ttgcgtaccagcagcggcctgtggctggtgtctcccttgccccgtatgctgagtttgatgcaacgctgct

ggggcctgcagttcagaagttcgctgcaaagcttggccgcgtgccccagcgcagcagccacaacgcccgc

ccgtcccgtgtgaagcatcgtgtgtgtgagattgatgtcaaggcacttctgaaggaggctgaggaggagg

ccgttcagaatgtggatgtctcgtgctaataatttggcatacgaacgtttgaattctttgaaagcggtcc

atctagcaaatacatacccgatacgattgtgtatgattgaaacgttgaaagaagggacgatagtgtattg

gggattcgatgcacgagggtgtgtgccgacgtgtgaagatgactaaactggaagcggttaggccgcgatg

gcgctctgcctgtgtgtatgtgcggtagaactgtgactggtcatggctttggccccggcacttcggtgac

agcaagcagttagcaggtatagcatgtacgccgcgcaggcaagcgaaggctcagagttgcttggggaaac

gcgctgcgacatgcctgtgcatatgatcgcatttggacttgttcattagtgttgcactgcgtgaaacaga

tacaaaagccgccgtttacagttgcgaaaaagcgaaattgcaacatgtcaagatgtctagacattttaca

tggcttccacaaaggcactttgtctagacaaaaaaatcgtggatttgggggcctgtttgtctaccccctt

gacaaaaatttccgctccagaggccaggaatgtctgggtcaagacattcccccgcaaacaagatacgacc

cctg

>Cr_hAT-N11 Chlamydomonas reinhardtii

cagtgctcgtatcttccatctacgctgtagaaatttgccccaaatccgccgaaggcggagaaattttttg

gggtttggggccgcgcatctacggagtgtagacgttttgggcggatttggcggtatcttagacagggagc

gtagatgttttgggggtttggggggcgtgcccctagggaaaattttctgggcgtagatggcgcgggaggg

gtcatctacgctgtgtagacgccgggggcctaaagccatctacaagataccggcactg

>Cr_hAT-N1 Chlamydomonas reinhardtii

cagtgtttccaaaagcttatacaaggtattagcgttatagtaccccacgggtacctgcctgctcaagaca

ttttacttccagtacccgggggccgaaggccccgtctccccttcgtagggggggttcgggcgggcggaag

ccccccccaaaattttttttcacgaccataccaaaatacactgttttgagtgaattgcatgcgtttaagt

cttgccacgtcgtcctgtcttgcttccaaatcttgagtatcagccagcccgaaactgtttccgccccgct

acatgcagcttcctgcgtcagtctgcacacccacagccacagcaaccacaactactaagtgcattaatcg

ccccgccacagcttagtccgcccagcggaactcggcactgtcacgccagtgcttcagggctgccgcgtac

gggaagtccgacacagagaaccaccgctgcgccatcatgcgcacagccaggcacaggtgccgctgctcca

gccagttgcgctgcttgttcttgatgtagctcatggtgctgaacacacgctccgcctccactgatccggg

tacaatggtagccgccagcttggccaggcgcacgaactcactgcagctgtcggcaatgtactgcgcgcga

gtcagcgattgccagaacgccacgaagctgccctgcggcagcgtcacggtgccagatgcagcagcctcta

agacagagctcgcgtggtcgattgcctccgtgctgaacagggcccgggtagtatagggtccggcggatta

aatttttggtacccgtgtgggtactcgaggccccaataattagtttccagtacccgcgtcaatttcggcg

aattattttctcagtacccgaatgggtactagtacccggttttggaaacactg

>Cr_hAT-N2 Chlamydomonas reinhardtii

cagggctcctatcttaatgtctccagacattaattggccattttggccgtttcctagacattcctcccgg

gggctaatgtctggggcgagactttgttggcccgggtttggggcgggttttgtccgaattcctatggaga

agcccccaaaccgccatgtgcccagacaaaagacggcggccacccggccgtttttgtctggagctagaca

ttaccccgaaaacaagatacgacccctg

>Cr_hAT-N3 Chlamydomonas reinhardtii

cagggttgcctagttcgtagtacgcgtactatgtactacgtactgtactacgtactgtacgctgccctcg

tcggctagtacgttggaaaatacgttgtgctggggaaaccagcggggtgggccgtggcagcgccgtggct

gggcgaggggcgcgaggggtgctggaaaggatgtccgtcgaagtttgcgaaccatggctgctctccgcac

tgcagctgtgttctcttacaacaatatgagcataataagttgaaggaaattccgctagaagactatgacc

gcgcaaatccaaattcacaccgattcgggcatggtgcgggcacgcgtgccgtgtttgcgtcggcttcccc

cccggatttcctggcccagcgtattatgcaccgtattttaccgtactgtactaggaatcctagtacgctg

tattaatacgcgtattatggagagagggtcgcgtactaggcaaccctg

>Cr_hAT-N4 Chlamydomonas reinhardtii

caagcgggtcccactccaggaaaaagcgtgtccccagggaggcgcccacgggatggcaccaaaacccctt

ggtagattgtccttcaaggaagccgccacgtctacggactcatctcgtcgtcatctcggcgcctatggct

tgctgatgcttgaaggacaatctaccaggggggtttcaccgcgtgccgtccggggggtgcgtttccccag

ccaaacccgcacgccgaggaggccaggagacgtcgccccgagaaaaatgcagagcgggatacagactccg

ccacttaatgtatatgttacatgcaatctgttggagcaatagcgcttgacagcaggctaaaacgtgctcg

tcgaggctcaagtcgcgaaattgaccgaagccaacatcctacataggcgttgttttcgggctttatcgat

tgccgtcaacacagatatatgcataggtgtagttaggctactgtctggcctggtatggcgtgtcctggcg

aacgcgacatttagatgttcgaggtcggccccggctcgattcaatgcccccgggttactctaatgacctt

atgtgtggtataagatgctataggaaaggtattgggctggggacacgaccggcccgtagggccgtgcgcg

agtgggacccgcttg

>Cr_hAT-N5 Chlamydomonas reinhardtii

cacgcgggtaccactctggtcttttgctgtgtccaaactttgacagcctggtagacccaacaggtatcat

ctgaccaggccgccagcatacctacggcttcgtctcgttgtgtagtatacggttactatggtgcggagct

gagcagatgaaacctgaaaggccgtacggctgcagccgtgcatggggtgtgcagtgggacacgcaaatat

tttcgtcgtgaaaactaaaagcgtgatagcgtgatggatagcgtacgcacagtgtatgcaggatacgcaa

tcgccactgtaaaaagaataggcatcaaaaagttatgtgcacaattgtccgggggggtgcatgctcctag

ccaaacccgcacgctcggagggctctgggagtcgcccgaggcaaatcctaccgcagtatttagacgccgc

ctttttgtaggtacataacttacatcattagtggacattagcgctcggcagaggacaaaacatgttggtc

agggctcaagtcgcgaaattgaccagagcccatatctcgcataggcggtattttgggcctgtatctagtg

ccgtcgactaaggttcacctatakatgtcatccggacagcgtagggcgaattccagtaagtcctggcgag

cgcgacatttagcatttcgacttcggctcccgctcgagtttatgcccggaaatcattcagggaccttatt

atatgtcacatgataacatttcggcagtctagggcattggacacacgcagtccctagggacttgccggag

tggtacccgcgtg

>Cr_hAT-N6 Chlamydomonas reinhardtii

cagcgctcgtatcttccttcgttcggaacgaaatttgcccaaaaaggcctctggcggacgaaattttttt

gatcttgacttggaatttcggccaaaacgcacgggatcttggggggtctttcggcaaacagacgaagaca

tttttgggatttcggccacttttggccaaggacagggggttccagcggctactccacggccgaaacctgc

gtggcatatggtaagaaggctcaaagggctgtatctatgggtgttgacggcgggagacaggggttcccct

ggcgggagagtgaggttgggtcggcgggaagcaggggaggagtgggcgtgtcgtgtcggtgtcgacatcg

tgcgggtcgggggcggagcccccggaaaaattttgggggtgaacgaagcggcggcagcctgtttcgtcaa

ctaaacgaagccattttaaggaattcggccactgacgccgcccctgccttcgtaagatacgagcgctg

>Cr_hAT-N7 Chlamydomonas reinhardtii

cagtgctcgtatcttccttcttttcgacagaaatttgcccaaaaatgcctccggcggaagaaatttttga

ttcttgtggccaaaaatctgtggaaacgcttcaggtttggaggggtctttctgagattcgcagaagacat

tttagggcgtttctgtcacttgtccgggcccaaggcaagggtgactgaggttccccaggaaactctatct

acggccgtgtcctgctggcaccgtgggggcacaccaagatagggcagccttctactaggggagtcattac

gtgggtgggtgagcgtgatgagcgtgggagagggcgctggggtttggcgtggggatgggccacgttttgc

catggagccatagagccggtgctttttcagcaacgtagtgttaacgtggcaacagcaacctcaccccgca

cgctgtaacaggggcacatcccgtgcggcggaatattttgctgtgccggagccgaaacgtgggctggggg

caacagcaacctcaccccgcacgctgtaacagcggcacatgccgtgcggcggaatattttgctgtgccgg

agccggaacgtgggctgggggcaacagcaacctcaccccgcacgctgtaacagtggcacatcccgtgcgg

cggaatattttgctgtgccggagccggaacgtgggctgggggcaacagcaacctcaccccgcacgctgta

acatgggacatcccgtgcggcggaatattttgctgtgccggagccggaacgtgggctgggggcaacagca

acctcaccccgcatgctgtaacagaggcacatsccgtgcggcggaatattttgctgtgccggagccggaa

cgtgggctgggggcaacagcaaccttaccccgcacgctgtagtttgtgtggtgtggagtttctgttttga

gatggatgcacggcacgaatgggagattgagagattgctgacacggcctgccacacccctggcttgacac

gaaccgtgcataggtcgggggcggagcccccggaaaatttttgggatgaaagatgcggcggcagcgggtt

tctgaaaattaaagatgccctatttcggaaatctgtcactggcgccgcacctgccttcttaagatacgag

cactg

>Cr_hAT-N8 Chlamydomonas reinhardtii

cacgcgggtaccaccaggcggctggatggggtctacgggagccccttcttccgcgggccttttcactatg

agcaataactcgtacaaggatgctgacccgacctaccccccacaattcgatagcgctctatccgcctggc

tagttagatgtcttgtacgagctattgcccgtggtgaaaactgggctgccgtccggggggtgcgttttcc

cagccaaacccgcacgttggacgttgccccggggcaaatcctaccgcacggattagacaccgcctttctg

taggtacataaccaacatcatcatcagccagaagtggtcggcaaaggtccaaattatgcttatcggggct

caagtcgcgaaattgaccgaagcccatatctcgcatatgcgctgtttggggcctgaatctattgccgtcg

acattaattctcgtatagatgtaatcaaaatagcttcaggctaagttggcggggtcctggcgagcgcgac

atatagcatttcaacttgagctctcgctcaaaattatgcccgagcaccatccagggaccttattatgtgt

aatgggatgtcaattcatggtcggggcgacagtctgggcatagaccttgcgatcccgcccttgacccccg

gagtggtacccgcgtg

>Cr_hAT-N9 Chlamydomonas reinhardtii

cagggttgcacagtactcagtacgtgttagtacgtactagtacgtactgagtactagtacgcctctcctg

ggtctcagtacggcgcaaaatacgccaggggggcggggggcgggggtgtgggggtcgccggcgggtgccg

gtggccggtggtcgtgccgtgtcaatgcgtcggtggcacagtgcagtggcgagaggcaacatagacgcaa

cataggcaacgtcttaagcataatatgattctgtgtgtgtagttgctcacaaccaaaagcctgatatcag

tgacacgatggccgatgcgatgcgcgaaccgagcctgccctattggatacgcgcaatggagctctggatg

gataaacttgatggccaaacagtaggcaaaacaggctgcgcagttgtcactccgctcatttctcgacatt

taataacaagtatgcgtttccaacagcatagcgagctgtaacggcctcgagtatggtcatgtatagtagg

atacagggaaggatactaaggaggaacacggagagcgaaatttgcaatgggaccgggcataggctgcatg

ggacgtactatagcacgtactaacccttgctcgccggcgtattataagtgaattatggtgtattgtacta

gaaagctgtgtactgcgtattaatacgcgtattataagaaaatggctagtacagtgcaaccctg

>Cr_hAT-N10 Chlamydomonas reinhardtii

cagtgtttcggcaagtttttagggcctttactttcctagtgcacgggtgggcattggggtgcggtgactt

taatttccaatgcccaccagggggcgcagcccccccacctcgtacggggggtccggggggcggaagcccc

ccggaaaattttttcactatgggtatcaaaatacgcgtattttcgtatgtcatrcccctagtcctcgagc

agcgtttgtgctagccgacaaggcatcacgcatgcgtgcgcacgccacacatcttctcttcatactgcca

caccgcaactgatgagtctttccccatcctgccgtgtgcccatccgggcgtgcccgtcgccccggccccg

ccggtccggcccggccccggcgcgcccctctcccttcctggcgcgtgcaccgggccggggatttcgcagg

atgggtgggcacttgaggggtcagggaattattttttatagtgcccaccgggcactggggctccaggcat

ttaattttccggtgcccgccctccgggcccgacttatttttccagtgcccacgtgggcactaatgcccac

ctcccgaaacactg

>Cr_hAT-N11a Chlamydomonas reinhardtii

cagtgctcgtatcttccatctacactgtagaaatctgcccaaaatccgccgaaggcggagaaaaattttt

gggtttgggtgccacgcatctacgcagtgtagacggttttggccggttttggcgcaaaaatgggggaagc

gtagacgttttggggggcttgggggcttaagtgtagatgccgcggtcagggtcatctacgcatgtgtaga

tgccgggtgcctgagggcatctacaagatacgagcactg

>Vc_hAT-1N1 Volvox carteri

cagtgtttttttttaagtaatatattacttttaaggaataaagaggcctttttggcctttggaaataagg

aacaaagtgaggccgatccgtacattttcattccttcagccaaaaacgtccattgctgccagattgctcc

gtagtgtgcagaacttagtattatcgatcataaaatagatatgaggggccgtaagaaggtcaagcccgtg

agtcaacgcggcatagcagaatgtatgaccggtccttccatagacaaacacgcaaaaggcaacgacttga

acgccgaacaaacacaaaaacagcaagataagtctgaaatttttcctgataaaggcgtttgggacggcgg

tgctgttgtcggtaatgcatcggcaatgttgctgccagcggcgaccgatgacacgggcgccattacccct

gccttccatggcggttgtgatcagcataagcgcaagctgtcacccaatcagcttgacaacaacgaatgcg

gcgaggcgcccaacccgagcgctcgcagtgccaagcgggctcagacacagcgcatccgcaagggcatgag

cgacggtgcatggcgggacaagcagaaggaaaatccccacagatggcagattgcctgggccgccaggtat

ccctgggcccagcctgtgcatgctgacgtggacgccggagaagtgcacgaccaagtgcgctgcatggttt

gcagtggcgggtgacggcctttgatttgctatgcggttgcttgctttgggcctttgcttggtcggattta

ctttttgttcacaaacagtgccagaactatctatgcatgtgcatgaagagtgcgcacatgaacgaaaacg

cgccaacgcagtgcgtgcctcttggtctatgaggggagttgtaacttatcggggggggtctggggggggc

gtagccccccccagcgccccactcttaaggaatacataacggcgacgctagcaactttgttacttaggtc

ggaataaattgagaccccccctaaatgaaaaaaaaatcactg

>Vc_hAT-1 Volvox carteri

cagtgttttttttttaagtaatatattacttttaaggaataaagaggcctttttggcctttggaaataag

gaacaaagtgaggccgatccgtacattttcattccttcagccaaaaacgtccattgctgccagattgctc

cgtagtgtgcagaacttagtattatcgatcataaaatagatatgaggggccgtaagaaggtcaagcccgt

gagtcaacgcggcatagcagaatgtatgaccggtccttacatagccaaacacgcaaaaggcaacgacttg

aacgccgaacaaacaccaaaacagcaagataagcctgaaatttttcctgataaaggcgtttgggacggcg

gtgctgttgtcggtaatgcatcggcaatggtgttgctgccagcggcgaccgatgacacgggcgccattac

ccctgccttccatggcggttgtgatcagcataagcgcaagctgtcacccaatcagcttgacaacaacgaa

tgcggcgaggcgcccaacccgagcgctcgcagtgccaagcgggctcagacacagcgcatccgcaagggca

tgagcgacggtgcatggcgggacaagcagaaggaaaatccccacagatggcagattgcctgggccgccag

gtatccctgggcccagcctgtgcatgctgacgtggacgccggagaagtgcacaaccaagtgcgctgcatc

ccctgcacgttgatgaagaagagcgactacctgatcatgtgccgggcggctaccctcgacaagcacgtga

cgtctgaagtgcacaagacgtcagtggagcatcaacagagccagaaggcgcgcctggcactccagcagca

gcggaacgcgaccctgccacagctctacaccccgggcaacacggaggcagagaaggccaagcaccagcag

ctcatccagctcttctggcagcttacccgggggcggcccatcacggacttcatggcttctaaggaggcgt

tcgaggcgctgtccgtcccacacataagcagctcacactggagcggcttctcagcctggaacctcgctga

ggcgcttgacggggtactgatggacaaggtccgggagcttgtgcggagcgcgcgattcatttcgctgagc

atggacgagtgcgtgggcatcgacaagaattcgcgcctctccctgcacgtgtacgtcatggatgcgcagt

ggagccggttgccgctcctgatcgatgtgagttttaacgggtggacaggctgccgggactgggggtttgc

cgcatttgtcttgttctccacaagttgtaacatcattcctcacaagttcctatcttctgaacttgtgtcc

actttaagtatccctgcatccccgcagttggcgcatgtacggggcgcacctgacgctgagaacatcacgc

agttggtgctggatacactgaacgtggagggcgacctcggggagcaggatttggcgcgcaagctcgtagc

ggtcagcactgacggtgcaagcgtgatgacgggcgtgcacaatggggtgtcagcaaggctcagccagacc

gcaccgttcctgctgagcatccactgcatggcacaccgcacagacctcgccattgccgtgctggaaaaag

ccccggtcgtcaagatgattgtgagcgtcatcaaggacgtttacaactacttctcgggcagtgctaagcg

tgtgagcatgttcgaggatgtggccaaggctgcaggaacggatggcaaccggctgaagcgcatcgtagaa

acaaggtggatctgtgcacgagagccggcagcctccctactggccgagtacccagcgctgctgccgctgt

tctgtgaggaggaggagaagctgtataacacactacgggacgcacgcctgtatgtctcaatgcacgcgct

gatgcccgccctggtcaccatcgacatgttcatcaagctttgccagagccgtgggctgtatattggtgag

ctggccaaggcgcttcagcgcaccaaggatcggctgcgcgagatgtacctcaggccggagacccgctaca

ccaccgtggagtttggggacttcaaggagctgctgtcagtcgatggagacatcaagggcaccgcctgggt

gttcaatcccgacacgccgggggaggagcggatcggactgatgtgtggcagcagcggcttctaccccttc

accgctgatcctcctcgcactggccgagaccgcacaccaccgcctttgacaccggaccgcctggcccagg

tcgtgaagtcggtgcaggcagacatgtcagccgcggtcgaggagctgataggtgagctgtcttcgcggtt

tcccccacatccactgctggaggcggcgtcgattgtctacagtgagtactgggagaacaggcccacggca

gccgacttcaaggcgaagctgaagatcctcaaggattactactgccgtgagcgggtgggcgccaactcca

gcaaggtgccaccgatgctggacagcgccttgctggacgagcaggcgaacttgttcgcctccagcatgaa

tcagctcgccgcacagcagcgcctcaggcgggcgcgagagcagaccgagcggcagaggcagcaggcggca

gcagtggacccagacgcgcccacatccaagcgtgagcaccgggtgctgctcgatgacacacccacagcac

gggtgtggcaggccatcagcaactgtgcggtcctcgccggcggcttaacggagtattcggctttggccca

gctggtgttagtgatggtgccagggtcggtggaggatgagcggcggttctcggccatggggtacttgcag

gaccccacccgcaaccgcctggacgagcacctagcattgtgtgtgcggatgtttacccaggacctgttcg

acctgggcacgttcccgttccgtgacgcactgaggaagtggctggacagcagcagcaggggccggtacat

gatgaaccggtagagtcctagtgtgctgtggtttgcagtggcgggtgacggcctttgatttgctatgcgg

ttgcttgctttgggcctttgcttggtcggatttactttttgttcacaaacagtgccagaactatctatgc

atgtgcatgaagagtgcgcacatgaacgaaaacgcgccaacgcagtgcgtgcctcttggtctatgagggg

agttgtaacttatcggggggggggagtctggggggggcgtagccccccccagcgccccactcttaaggaa

tacattacggcgacgctagcaactttgttacttaggttggaataaattgagaccccccctaaatgaaaaa

aaaatcactg

>Vc_hAT-2N1 Volvox carteri

cagggttcttcaccccgattcgggcccgaacttttttgagatatttccgttttgcccgaaaggaaaatta

gtgcgccccggagcattccgaacggattttgggccttttggagcattttttcgggggccccaccgaagcc

ttttgaaggcattttcggagaaaaccgaacgatatttcagcgtaaatttgcatgttttcgggcgcagacg

gggcctggaaaccgggcttgccgtataaggcccgcttggtcaccagtcaaacaacaagtacaaatgtgca

ttgcggctgtttcgcagctgtatcgcagacattttcatacatctaagcatatccatatgccacattttca

tacatctaagcatatgcatagttaaacatgcccgagtcagctgatgatgagcgcccaaccaaggtgcccc

gcatctccctcgggattcgcggcttcggcaagctcgaaccgcctgtttgccttcgatgtcagcgaacttg

acgttttgcagaaagagttttcagttatgaagtctaagatggccttgcagttggagctgggccgcacctc

ctgggagcgggcctgggctggaatgcatcagtgggtccacttctttccacgcttggttcagttggtgtat

gtcgcttgtgtcctgcccactcaaacagcgtgtgttgagcgtggctttagtgtccaccgcattatcaaga

acaggctgcgtaaccggctggctgtcccgtccattgattcccttctgcgtgttggattactcggtccaac

cgtggatatgaaatgccaaccccttgtgatttaaagctgctgaggagtatgttgcaaagaacccggatgg

gctcctgcaccgtattgccaaggtgggcgactgtgttctgcctgatgagagtgatgatgagggcagtgtg

ggcagtgaaattgctgaggcctattatccttctgataacgaggaggtcgagagtgtggcaagtgatgagg

tgtacccgtctgaggagacggatgatgatgtggcaggtgtggtggaggcgattgctgcagaggtggatga

ggagcggcgcctccttggttttgggtaaaatccttgctgaaatatttctcccaggcgctatgttccaaaa

taaagtcaaacacctgttaattcagccgtcctagcatataacgatagaatccagctaaattttaccccga

aaaccgcttcagacagtttcggggtacccgaaaacactgtcaggctgacaggaaaattttcggggagggc

cccgaaaggttattcagagggtttcggacacggccgaaatttttgggtaccctggtgaagaaccctg

>Vc_hAT-2 Volvox carteri

cagggttttccaccccgattcggacatgaacatttttgagatttttccgttttgccccggagcatcccga

accgattttgagccgtttaaagctgctttaaatgctgcatttcttcgggggccccaacgaaaactcctga

aggcattttcggagaaaactgaaatatatacctaagcgaaaatttgcattttttcgggcacggaaggcgc

ctggaaaccgggcttgcagtgtaaggcccgcttggtcaccagtcgaaagtcaaacaacaagtacaaatgt

gcccacagtgtaacaccccatcaaacaagtacaaatgtgcattgcggctgtatcgcacatattttcatac

ttctaagcatatttaaccataattaaacatgcccgaatcagctgatgatgagcgcccaaccaaggtgccc

cgcatctccctcggaatacgcggcttctttaaggcatctggtgagttcctatcatacaagttgtatagta

gtggcgctatgaaagtatctggggaactgatgcggaatttacacctatgcagagaaatctcccgtcgttt

ctacacctgctgctaacggcaaagttgctgaacatctttcagggaacaatgactgtaagtttcgataagg

tcgctgcaatcttcaaacgttttgtcgaccgacagtcttgcatcgttactagaaagtagccagaaaacta

accagcaattgttattatacactgcagccgcacctggacagccaattatggctcagcccccgtctgctcc

ggctgcacctgaggtggtggacttgtcaggcaagctaccgaaggcgcctgtctctgacatggggcaagca

gccttcgtggagaagtacccctggtggcagcaaaccggcaaaaataagcaaggccaagcgattggcggct

gtagcatttgtcagcagcacaaggggtccggtggtggccctaagttaagtcctatcgtgaaggactcgaa

tgaccttaagaaacacgatgagagtgtcggccacgttgaggcggtgtgtattgcaaaggcagcaacaggt

cagggcccgattgacaaagccacacgtgtagctgcccagcaagtcgcagccggtcttgccagtgttctgc

agctgttgatcctgtgcgtcctgtggatggtaaaagagcacattccggcactgaagctttctagcctgct

actgtttctggcggtcgcgcgtgtcgatgttgtacgccacaagtacagccaccaccgctactttatgaag

gctctgcgcgctcttagtcaggtcgttttgtccatgcagatagccgaaatcttgcaaagcccttatttta

gtattttgtgtgacagtggtactgatatttcgggagaagatcaccttttgttttacgtacgttacctaaa

cacggacactttcatgtctgtcactcaatacctttgtgctgtgcgtatcatagacaagggagcagagaac

attcacttggtcactgaagctgtgcttgctgcccttcgtctcgatcagacgaagatggtggggtatggca

gtgatggggcaagcgtgtatctgggccgtaactctggggttgctgtgcgcctgcgtcaaaagctgtgcta

tttggttgccatacattgctgcgctcaccgcacagctctggtcatgggggatgtgaaggaggagttgcag

caaatgtctgcaatggatgccttgctaaagtccgtacacaacttgttccgttcgtccaccatgcgtgaga

agtgggagaagtttgctgctgagcggggctgcacacggttgaagtttcctgtctacaactccacccgctg

gtttagccgtatgcagtgcgttgtgactcttggcctgaacgcccacgttctcctcttgttccttaacaag

tacaaaaagaactggccgcaagggcgagcagttcacgagcagctgagcaatgtgtacatgctggctacgt

tgtttgccctgcgtgacctgctagccccctgtgagcggttgagcaaggccttccaagccgatagcttgaa

ggctcatgaaatccacgatacgctttcggacaccaaggacagcttactcaaggcatgtgggttttcgtct

ggtcgcttaaagggcaagcactatgccggctttaagagtggtctgcaagggcgatcctgggttgtggttt

ccaaccgtgtaaaggtctccggcacgctagatgagggaaagttacagtcttttgcttgtcggtttgtggg

tttaattttgaagcatttggatgcacgttttggagacactgagcttctgaagtgctttcgtatttttgat

cctgctacgtatgtgaatatgtctgaggaagagcggcgcggctacggccgtaaggagttcgtgaagttgc

tagtgcacttttgccactcggcaaaccctaaccgcctgtttgccttcgatggcaatgaacttgacggttt

gcacaaagagttttcagttatgaagtctaagatggccttgcagttggagctgggccgcacctcctgggag

cgggcctgggctggaatgcgtcagtgggtccacttctttccgcgcttgaatcagttggtgtatgtcgctt

gtgtccttcccacccacacagcgtgtgttgagcgtggctttagtgtccaccgcattatcaaaaacaggct

gcgtaaccggctgtctgtcccgtcgattgattcccttatgcgtgttggattactcggtccaaccgtggat

atgaaatgcaaaccccttgtgattaaagctgctgaggagtatgttgcaaaaaaacccggatgggctcctg

caccgtattgccaaggtgggcgactgtgctctgcctgatgagagtgatgatgagtgcagtgtgggcagtg

aaattgctgaggcctactacccttctgataacgaggaggtcgagagtgtcgcaaatgatgaggtgtaccc

gtctgaggagacggacgatgatgattagggctgggaattacggttacggtccttacggagttgcgcatta

cacctttcgcaattacggtgaccccccaaaaacacagtaaaatgacggtccttacggtgtaatgcaatta

cggttaccaaaaggtccaaattacggtcggaccgtaagcgtgcgagccaataccgacgaccacacctcga

aacatgccatttcgtcccttggagctacgggatagtcttaaaaagcttattatacattaacctatgatta

aattatcacaaaagacagccaaaaaatgctgaatgaggcgaaaacatttcaaccggagcaccgcgcgcgc

atcgagtactgcatctgttgtatgccatcatgccatatgctaaatcctcaaccaagaatgatgctcctaa

tatatacgggccgaacgtgcgtaacagtgtcagcctctgcctcctgggctgcgcattgccgctaggggca

tttcgtcatcatcgctgctggcgtcctcctcagtgtcattctgttgctgcagcggcacctgggtcttagt

tccactgttatcaaggtggtcccgctgctgctggccctccactgtcacctggatgtcacccatatcgcgg

ctaaatacaagcttaggtgcaagtaaccctgataaaactatgaaaaaaggtatgaaataagtttacatat

cgttatataaaagaaaagaaaggatttggctccgacaacgaggaccgtaaatcgttacggtccactttgc

cccggaacccaccgtaaatttcggtatttacggcgttgcgcattacacctttcgcaattacggtccattt

tatgggattacggtgtttttcccagccctaatgatgatgatgtggcaggtgtggtggaggcgattgatac

agaggtggatgaggaacggcgcctccttggttttgggtaaaatccttgctgaaatatttctcccaggcgc

tatgttccaaaataaagtcaaacacctgttaattcagccgtcctagcatataacgatagaatccagctaa

attttaccccgaaaaccgcttcagacagtttcggggtacccgaaaacactgtcaggctgacaggaaaatt

ttcggggagggccccgaaaggttattcagagggtttcggacacggccgaaatttttgggtaccctggtga

agaaccctg

>Vc_hAT-3 Volvox carteri

cagtgtttttttttggtaatatattacttttttgggaataaatgggggtctgagcctggaagaaatggaa

taattggtcagttggccgtactggttcattacttcggccataagtcgccccaaatgccttctgcacatat

aaagctgaacatttggagcttatacactatttatgtataaaatatttgatgatatcaccatgagacggcc

cgctgttgcattcaacttaggtcaacggggcattcgttcattcttgaccaaagcaaacggctcagaaagt

ccgcagacgcaatgccccaaacatttagaagaagaattagataatacagaagacaaggataggcatgccg

tagttgccgctcttgaagtcagtgaagccagcgatttgagcgaagctgaggatcccccggcgaagcggca

acacacaactgcagcaatcagcgactcagatgctgagcagcttgattctgacgaggaagggccaccgccc

caagatgtcgccacggcaccaccgccaccttcaagggcgggtgcttcgcagcacgcgccgctggtgattc

aaaagcaggcgcggcgccagcaaacacagcgtgtgccgaaaggccaaagcgatgggagctggcgggagaa

cgagggagtgcgtcggtgcggtccctgctccctcagccggcggcgggacgtcacgatggatgccaggcag

gccacgctaaaaagccacgagctctccaaaaaacactaagaagccatgtgcagccacgaagcgcgggccc

ttcagcacgagcggcaccagctgacagcgcggacggtcgacaaccttttgtccagagcactcagcaagtc

cgagaggggagttgtgcagcagctgcggcagctcttcttcatgctggatcacgggcgccccatcctcgac

tatgtcggctctaaggacctgttccaggtgctggacgtgccacactaccgtgataagcactggagctgca

acagtgcctggcagcttgccgaggccctggatgcggtgctcaaggacgaaatccgcaggctggtcgccgc

caaccgctttgtatccgtgagcctggacgagtccaggggcattgacaagaggtcccggctctgcgttcac

gtctatgtcatggagggctggagccggatcccgctgtgcattgatgtgaggccgggatgatgggaaggtt

taaaattaacgtaatgccggggtgaattgctgggagcccagctcttttaaccgttactcttttgtccaca

tttcctcagtctagacggcttcctccttcaaccgttttttttggccgtggatagaccccattaaagtctc

tgtttgtctctcttttcgtgtcctacagctggcaaaactggaggcggcaccgacagcggacaacatcacc

agcttgctgctggaggtgctggcggacagagctgggctgacggccaacgagcttgcgcagaagctggtcg

ccgtgagcactgacggggcaagcgtcatgacgggagtgcacaacggggtctcgactcagatgacggacct

tgctccgcacatcgttaccatccactgcatggctcaccggacgcagctggcagccggtgcccaggagcag

gcggcagtcatcagcaccctaaaggcactggtccgggtcatctacagtcacttctcgaacagccccaagc

ggctcttggccttcgaggccgctgcagaggaggctgataccgctggaaatcaccccctaagggacgtaga

gatcaggtacgctaagccgaggatatatggcgggtaaacatgagggctgcaagggccgcttagaacctgc

caaacgccgtttgctttatactattaacaccaccttactgtttgtttctgccgcccccgcaggtggctga

gcctgctgctgccgctgcagcgtgtcgtgagcgagtacggcgccctgctgctgtacatggaggaggcgga

agagcagttcatcttcacccagatgacggacgcccgcctgtacctggccatgcacgccctactacccgcg

ctgcggcgcaccaacttcttcatcaagctctgccagagccggcacctgttcatcggccacctaaacatcg

agctggagcgcctgaaggctgacctccgccggatgtacctggacagcgccacgtgctatcagggtgcgga

ttttgtggactacacccagctactccccacgggccctgcgggtgacagcctagacggcactcagtgggag

ctcagccctgaggacgagagcgtcggcctggtgtttggcgaccgcttcattgccttccacgcgatgcagc

agcggcggggcagaggcgggtgcatggtcagcctaacggcggagctcatcacgtccactgctgcagcagt

ccaggcggagcttactgccgcagtgcaggtgctggccgacgagctggaggcccgcttcccccccacggcc

ctgctggacgccttccaaatcctctacagcgagtactgggaggccaggccgacagcggcagacttccggt

caaagctgaaggtgatcaagacccactacggcaaggagtgcgagctggacgacgggagtaaagtgccgcc

aaagctcaacatcgcggcattggatgccaaggccacagagttcgagcagctcatgcgggccttggcaacc

aagcagcgactggcccggattcgggggcagaccaccaagctgcgtgagcagcttgaggcggagcgggcgg

ctgctggcagcaaggtcgtgcagtgaaagctacgtgaggagcaggaggagagccccaccatccagctttg

gaagcagctggagaacgcccctgtccatgcggagagcctaaaggagtacgtggctttggccaagctcgtg

ttcgtgatggtgccggggtcggtggaggacgagcgccggttttcggccatgaacttcgtgcagagcaagg

tccgcaaccgcctggacgagcacctggcgctctgcgtcaggatgttcacgcaggagctgtacgacctgcg

cacgttccccttcgacgtggcggtgaagaagtggcgtgaggccgtgatcaaggggcggtaccgcatgaac

gactagcttctgatgtggtgcagaggcctagccagcaactggtcggcggctggtgcaatgcagtggtgtt

gatggtgccgacagtgtacataagggtacatatttgtacataccatgtgagatagttatctaatggaaga

ctatacatacatatatataactttgccttcgcgtgctcacgacgtttatggacagtcaaagtggctccgc

gccctgctcacagagacactggcgcaaatgcagttaggtgcacagaacgatggtggtacggcatgtgtgt

ttccagacgtcaggtgttgtttacagcacaccgtacggcctttgaggggggggtctggggggggcgcagc

cccccccagcgactatacaaataggaattgtaaccgcaagaaacacccagtttattactctagggtacta

ggcccaaaacgaaaaaaaaacactg

>Vc_hAT-4N1 Volvox carteri

tagggttggcacgggtactatactagaaaagagtactatactataatagtactatgcatagtactattta

ctatgcagggctctaaactggtactagtttacgacgctcgtaccgcgttcgtacatagtaaaatagtata

gtaaaaatagtactagatagtactagatttgggtttggatagtgtccagtactagttggaccaagtcgag

atctgcagtcaagctgtattttgaagggctatagccgcgtcttcgagctgtgctacgtagttattgatga

ataatgtcaaaggaatccagtataaagaatgacaaggcgatcagaggcttgcatcctggtccagacgtcc

cccctgaagttctccgttctcggaacgagctttttaacaagctacgtaaccagaagaagaacgccaagac

gggtagtctttgggacaacttcgatgtggagtggtggggtggtctaccaaagctagtctgcctgttctgc

gatgagccgctcagcgccgtgaacccatcctttaccaacgcgcaccacatcaagcaaggtgcttgcaagg

ctatcaaacagcgtgcagcgtcactcagccagcaacagcaacagagtggaaccagctctgctgcggcggg

ccccagcacggcggatcagcaggccctctccaagcgccggcgcggcattgatggccagacccagcagcag

cagcagcggcagcagcagggaaacgtacatccctaacacgcagattgatgactgagcgacgatagattga

gctgcggcagtttgccgaattggagggttacgtactgggccgttgtagtgcaggaacttcactagtatgg

cgtccgtgtgttgtcttgtcaacactgggtcttctatctgtgattatgtctttatgtgccataacacgcc

ttgagacatggggtttggggtccacctaccgggcactgaactaacaaaaagtgatctggatgaccacagc

gatctgctttctcatgtctaagtagatatttagaaagaacaaaagaagacctttccaacggtgtaatttt

caggtacaacggttggtcgtacaccccccttctacagggagcgtgtacctgcctagtaatagtatcatag

tactatattactatatatagtactatttcttactatactatactatttgggtaaaataaatatagtattt

ttgccaacccta

>Vc_hAT-4 Volvox carteri

tagggttggcacgggtactatactagaaaagagtactatactataatagtactatatatagtactattta

ctatgcagggctctagactggtactagtttacgacgctcgtaccgcgttcgtacatagtaaaatagtata

gtaaaaatagtactatatagtactagatttggggttggatagtgtccagtactagttggaccaagtcgag

atctgcagtcaagctgtattttgaagggctatagccgcgtcttcgagctgtgctacgtagttattgatga

ataatatcaaaggaatccagtataaaggatgagaaggcgatcagaggcttgcttcctggtccagacgtcc

cgtctgaagttctccgttctcggaacgagctttttaacaagcttcgtaaccagaagaagaacgccaagac

gggtagtcattgggacaacttcaatgtggagtggtggggtggtgtaccaaagctagtctgcctgttctgc

gatgagccgctcagcgcagtgaacccatcctttaccaacgcgcaccacatcaagcaaggtgcttgcaagg

ctatcaaacagcgtgcagcgtcactcagccagcaacagcaacagagtggaaccagctctgctgcggcggg

ccccagcacggcggatcagcaggccctctccaagcgccggcgcggcattgatggccagaccctcctccca

gtgattcccgcgactctcacggggcaagtcgtggaggagcttggcatgttcttctacaggaacaacgtgc

ctattcacctgatcgagaagcccgagttgcggaacctattcgggctgctgggcgtcaccctgccgaaccg

gaagcagttgatggggccaatgctggaccgcgcctacagcaaggttaaggcgcaggtggcgcaggtcagg

cagcgcatgtgtggccaagctgcactctgctcagatggctggcgcaagcgtgccgctgagcagggggtgc

cgctcatcaacttcatgctgctgctgccaacgggctcggccttcctgcgtgtcgtgcgtgcagctggtgt

acggaaggacgcgcagtacatcaaaaaggtacgttatgtgcggatatgtttagtctttggtgccgtctgc

cgtgatcgtaatttattgaaaccgtacttaagctaattaacctgcactgcagatccacctggatgtggtg

gcggaagcgttcaccaagcctgacgactgcatcggcttcgtaatggacaacacggcgaccaacctggccg

ccatccagctgctgcgcattgaaaacccccgctggctgggtgtgggctgcggggtacacggcatggcgct

tgtttttaaggacctggcgaaggagaagaacgtcaagtgggcggccaagatcttcaacgcttctatcacc

atctccacagtggttggcgactcggagcgcatccgggctttgctgggcgtgcaccagatggagaagtacg

gcaagaagtcggccattaccgccaacaccccaacccgcttcgctgtgaactattttgttctaagtgacgt

gctggcgaatgaggatgcactgaaggccatggtgcgcaacaaggaggcgtgggctggcgccagcgacggc

acctcaaaggcgcaggagtttaaagccatggtcgagggtgagggcagttcccggaacttgggcctctttt

cgaagggcgccaagctggcggagttttgcgagcccatttccaaggcgctgcacaagctggagtgcgacag

accgatgctgagccaggtgcaaagatgatttaagggtttgggcgaaaggatgtgtgtcccatgtttgccc

cagaccccatcaggcatcacctttcactcagtgacccgtcccccatacgcttcccatgttttctcccatc

cctcccagatgtacccggtctggaaggccctgcttgcaacagcaagggactttgatgaggcaaacaccga

ctgcccagggctgtacgacctgctgaaacggcgctacgacaagcaggtcaacgcctgctggtttgcagca

ttcctgctggacccgcgcaatattgatgtgtcgcttaccaaccggtacatgttaccgctcggccagctgg

agctggaggagaagtcagcggcgaaggtcctaatccaggatctggccgcaggcaaagatgcagctaagcg

ggaccgggttggggaagagtggacacgcctgggactgggcattcccaaagcgttcgatgagtggtctgag

cttatgcgcatgcacaaggagaaattgttgcctgacagcagcacccgtgtgatgggggcttcgctggatg

actgcatcagctactgggatgtgcacctcgccgcacaattcccactgattgcatcggcagccattcgctt

gctgctgatgcacgcatcaacctgctcgtccgagcgcaactggtcggcatggggcctcgtctacacgaag

gcgcgcaatcggctggccattgagtgggcggagaagatcgtttacatccgtggcaatgcaggcttgcagg

gcaaccaaggcgctgcggcggatgaggtggagctgattctccagcagtgcgaggaggacgagcaggagca

ggaagcacagcagcagcagcagcagcagcagggaaacgtacaccctaacacgcagattgatgactgagca

acgatagattgagctgcggcagtttgctgaattggagggttacatactgggccgttgtagtgcaggaact

tcactagtatggcgtccttgtgttgtcttgtcaacgctgggtcttctatctgtgattatgtctttatgtg

ccataacacgccttgagacatggggtttgaggtccacctaccgggcaccgaagtaacaaaaagtgttctg

gatgaccacagcgatctgctttctcatgtctaagtaaatattcagaaagaacaaaagaagacctttccaa

cggtgtaattttcaggtacaacggttggtcgtacaccccccttctacagggagcttgtacctgcatagta

atagtatcatagtactatattactatatatagtactatttcatactatactatactatttgggaaaatca

atatagtatttttgccaacccta

>Vc_hAT-5N1 Volvox carteri

cagcgttatttttttgcggaatatattccgaattcggaataaaaaaatgttcgtggtcctgaaagttaag

aataaataatgttccgaccgtacaagttcattcctccagccaaaaatggactgtccatgccaaagccgct

agaatcttacaggagacatacaaacatatatgttaaccatatcatagtactttaatgccatggatactgt

taggaaggtcacgcccgaaagtcaacgaggaatcggactgtttctgaccggaccaaacgctgcaaaatat

tcggacccgaagctcccaaaacgtggaaggccattaaagcagatattaaacttcactgaagccattagcg

acgttaatgaagacggcctagaggtcgtaggcaatgtggacgcaatgctgcagccagccgacgacaatga

cgccactccggttgtcaacagcgagcgggccaaacgtcaacgttcttcatatggagacaatcgcgatggg

ccaggaaacacgggcgtcgaggtcgatggcagcagcgaccccggcgctgcagctaagactcctaacccca

cactccgcggcgcaaagacgccccgaccacagacgcagcgggtcgtgaaggggcagagcgacggcgcgtg

gctggagaagaaggacaagcatcccctcaagtggcagctgtacttcggagctatgtttacatgggcgcag

tgcattacggcggatgtgaggatgtgttcccctttgaggcggcactggagaagtggaaggccgccagcag

caggggccggtacatgatgaaccgctaactagcgtgcagcatcaacagcaggggctgcaaccaaccgcag

aggttgcgtttggcggttctggttcattgctgctatgcatcattacttttgcaatctggaaagtcgtgta

gtgttatctttcaacgtgatgcaaaacgaacgacggtgaacgaactctatttgatcagtgcttgagtagg

ggttaactggtctgcatgtgtttgcttgggctctgcaacagcagtcgcccgtgcaaccggggttcctgtc

aaagcgtccaagtgcgcgccgcttcctggcgtgcaagagacacctctatggagagattgtggtgtgcgcg

tgttgtgacattccccaaaccctgtgtttgtacgaactaaactataactcatcgggggggtctggggggg

ggtgtagccccccccagcgccttacagattcggaatacttttattcccgcgactaaaaacctattcctcc

gttcggactggaaaattgacgggcaaaaatgaaaaaaaaaacactg

>Vc_hAT-5 Volvox carteri

cagcgttatttttgaggaatattttgcgaattcggaataaaatattgttcgcggtcctgaaaattaagaa

tgttccgaccattcctccagccaaaaatggactgtccatgccaaagccgttagaatcttacaggagacat

acatgttaaccatatcatagtactttaatgccatggttactgttaggaaggtcacgcccgaaagtcaacg

aggaatcggactgtttctgaccggaccaaacgctgcaaaaaattcggacccgaagctcccaaagcgtggc

aggccactaaagcagatattaaacttcactgaagccattagcgacgttattgaagacggcgtagaggtcg

taggcaatgtgaacggaatgctgcatccagccgacgacaatggcgccattccggttgtcaacagcgagcg

ggccaaacgttcatatggagacaatcgcgatgggccaggaaacacgggcgagtagaggtcgatggcagca

gcgaccccggcgctgcagctaagactcctaaccccacactccgcggtgcaaagacgccccgaccgcagac

gcagctggtcgtgaaggggcagagcgacggcgtgtggctggagaagaaggacaagcatcccctcaagtgt

cagctgtacgtcggagctatgtttacatgggcgcagtgcattacggcggatgtgggtaagggggaggtgc

acgacatggtgcgctgtggcccatgcacccagcacgaagggcacgagtaagtcatttgagtgccggaggg

agacccttggatcgcacgatacctccgccgggcacatgaaggccgtcaatgcagctcagcttcgggaggg

gaaactggcactgcagaagctaaagatgccgagcctggcgcagctcttcacgccaggtaggtccgaggcg

gagcaggcccgcctgcagcagctccgcttcctctactggcagctggtgcggggccgccccatggccgact

acaaggccgctgaggacatgttccagctgctgcacgtccctcgcaccagcacgtcgcactggagcttcaa

tttggggtgtgagctggcagaagcaccggatggcatggtgatgaacaggaggcatcgacaagaagtcgtg

tacgtgatggatgccaactggagccgggtgccgctgctcatcaccgtgggtggcttcgttggctgtgcgt

tgtttggtccgtaactgttgcatttctgtcactttgtgtgttcactgttgctgcaatggtgctacatctg

cagggcacaccggaccgttccccatcccacccaaccgcagatggtgcatctgcagggcacgccggacgct

gctaacatcacactgctggtgctcgacacgctaagcgtggaggccaaacctgatactgcggacttagcgt

ggaagctgttggtgatcagtacggacggtgcaagcgtgatgactggtgtgcacaacggatgtcggtgaag

ctcagcctggctgcacctttcatcatcagcattcactgcatggcacaccgcacggaccttgcggtcggtg

cgttggagaaggcgcccgccgtcacggcgttcctgagcgccgtcaaggatgtgcacaactacttcaagca

cagtgcaaagcgtgtcagcatcttcgaaggtgcggccgaggaggcggggacgtccggcaaccagatcctg

agactcgcagacaccaggtgcggggacgtgactgactgccttaccttgtgtaatgatgcctgtttatcct

ttgtagtttcctttcggaatatggtttgggcggacttttggttgttagttaggttgtgttgctagggtca

gcagactcttttgttcaatgggttcaactcgtgtagcacacacatccgaacacggtttactttcccgtta

tgttccaggtggacctgcgcgctgctgccactgcgacggttgctctccgagtaccctgcactgctggtga

acttctacgaggaggacgaaaagctgtacagccagctgcgggacgcccgtctctacatatcaatgcacgc

cttaatgccagtgttgcagagcgtggatggatgtcttcatcaagctgtgccaaagccgtgggctaaacat

tggtgatctggtcaaggcacacacgcgcctgacggggcgcttgcgggagatgtacctagatccggcgacc

agatataagaccgttgagtttgctgactaccggcagctgctgcctgcagatggcgacatcacagcgacct

cgtaggcgtacaaaggtgacgatgaccaggaggttatcggccttaactgtgggggcgcggtgcacccctt

cacagctgtgccccctgatgccgggcgtgatggcacgccggtccacctgacgccgcagttgctatctgat

gtcgccactggcatgcttgtcggcgagctcacgacgcgttttccctcacacgcaccgctggatgcgttct

ctatcgtctacagcgaatactgggaggctgtgcaagcgccaaccgtgccagacatcaaggcgaagctcaa

gaagatcattaaggaccactactgcaaagagtgggtggggcccggcggcagcaaggtgccaccgatgctc

gatggcatgctgctagatgcacagcagcagctattcatcagcacaatgacgcagctggctgccgagcagc

gcctcacacgaatgagggggcagacggagcggcggcggcaggcagccgcgaaggcggaggcacagcaaag

caagcacgcctaccatgcactgctggacgacacaccaacctcacgacgatggcagcggatcagcaacacg

ccgggtcttgcgggctccttgacggagttgtcagctttggctaaggtggtgttcgtgatggtgcctgggt

ctgtggacgaagagcggcagttctcagcaatggcgtacttgcaggacccagcccgcaaccggctggacga

gcacctcgcactgtgcgagcacatgttcacgcaagactagtttacgaccagcgcacgttcccctttgagg

cggcactggaaaagtggaaggccgccagcagcaggggccggtacataatgaaccgctaactagcgtgcag

catgaacagcagggactgcaaccaaccgcagaggttgtgtcttgcggttctggttcattgctgctatgca

tcattacttttgcaatctggaaagtcgagtactgttatctttcaatgtgatgcaaaacgaacgagggtga

agaactctattcgatcggtgcttgagtaggggttaactgggctgtgtttgcttgggctctgcaacagcaa

tcgcccgtacaatcggggttcctgtcaaagcgtccaagtgcgcgccgcttcctggcgtacgcaagcgtgc

aagagacacctctatggagagattgtggtgtgcgcgtgttgcgacattccccaaaccctgcgtatgtacg

aactaaactataactcatcgggggtggtctggggggcccatagccccccccaacgccttacagattcgga

atacttttattcccgcgattaaaaacctattcctccgttcggactggaaaattgacgggaaaaaatgaaa

aaaactg

>Vc_hAT-N1A Volvox carteri

cagtgtattttttttccgtaatatattacggttccgtaataaaatggcatatttgcgttctgcgctccgt

catagaccatggtaaaggcctggtacggtcgttacggagccggggcgggccccagaaggcccccagagac

gttctattttagtataaataagttataaatgagcgatggggcccaagaaattaactactatagctggtca

gggtagcgtcaaagcattcctgaccaccggcaggccaaaacccagtagcggcgatgctggtaacacttca

aacaatatagcgagtacaagagagacattagaaccagatgacagggcgcctcccgcacggcgccacgctt

cagccagtcaatcccaccaacagcctgttgcaggccaggaagcagctcaggcgcaccaggctagacgccc

ggagccggttgtatcaccatcgaaacgacgtaagctcaatgagccatcggctggctctgctggggcaggt

ccatcaggcacactgccagcgatgtggccagcaacgcggctgtgtttgtgatggaggaacagcggaggat

gagaggcggttctcagcaatgtctttcgtcaagaataagcaccgcaaccagcttgataagcatctgggcc

tgtgcttgcgtatgtttgcccaggacttctacacccgctcgtccttcccgtatgacaaggcggtggagaa

gtggctgcagagggctgagaagcgtggccgttacatgcacaactgacgagtttgctatgccctggggcat

tgtactggctgtggtgtcgtggtgaaaacgtatgacataatcctgaacgcatgtggcaacggacggaaac

agctgagactctaatgaaatatttattaaatatgtgctggtgtatcaggtaccctgtgtgggcagggggg

gtctgggggggcttgcccccccagaaaaaaaattctggcagtaataaacgtattggactgatggcatatt

tattacggaactcgtaacagattcaggatcggcctccacgaaaaaaaacacactg

>Vc_hAT-N1 Volvox carteri

cagtgtatttttttccgtaatatattacggttccgtaataaaatggcatatttgcgttctgcgctccgtc

atagaccatggtaaaggcctggtacggtcgttacggagccggggcgggccgaaggcccccagagacgttc

tattttagtataaataagttataaatgagcgatggggcccaagaaattaactactatagctggtcagggt

agcgtcagaaggggaagaagcggcgtcataagcagagggagaggtttccgtccgccacggcctgctggcg

acggctgatgagccccgctgggcaggccatggccatttcagagtttgcaaagctggcggagattgtgttt

gtgatggtgccagggtcagtggaggatgagaggcggttctcagcaatgtctttcgtcaagaataagcacc

gcaaccagcttgataagcatctgggcctgtgcttgcgtatgtgcttgcccaggacttctacacccgctcg

tccttcccgtatgacaaggcggtggagaagtggctgcagagggctgagaagcgtggccgttacatgcaca

actgacgagtttgctatgccctggggcattgtactggctgtggtgtcgtggtgaaaacgtatgacataat

cctgaacgcatgtggcaacggacggaaacagctgagactctaatgaaatatttattaaatatgtgctggt

gtatcaggtaccctgtgtgggcagggggggtctgggggggcttgcccccccagaaaaaaaattctggcag

taataaacgtattggactgatggcatatttattacggaactcgtaacagattcaggatcggcctccacga

aaaaaaacacactg

>At_ATHAT1 Arabidopsis thaliana

tagagttgtcaagcgggccgctagcccgcgggctttgtccaagtagcccatggcggagcgggcttggacg

ttgatttttttgtccacaaattagcgggcctcgcgggcaggcccattacggactgcggattatagcagac

aagcccgttgcggactgcgggtatgcgggtagcccacgaaagaaaaaccaactcttttaaaacccttcat

ttctcatagaggctttcaaaccttttctctctattagagtttcttgtgcagttcaaaccctagaagtagc

agttcacgctcaattgcttaacaaggaaactgaaaagtcattctctatctcttcaagtttcttttttacg

atcattgaagcttcttagattcagatttcagatcaaaacagagcatctaacacttgtaatggtatgttat

ttgtgactacacataaacaattagatgttttacttttgtttgattgatttgctagtcttgactagttttt

gtattttgttaatgataggatccgttggattctgttgatgctgagtatttgagatcattagatgtggaat

ctcggagagtcattgagttggaacaagctagatatgaacaacaacaagctaatgatgaagcaggaggatc

taaaaagctgaaaagaaaactgagagatgaaacaggaacacctatgacgcagaggaaaaaagtgtgtgaa

ccagaagcatcaaaaacccaaagaagaagcaagggtgatgaagcttctgcctctaaaacagagagaagaa

aaaagtctgaagctgaagcacctaaaaaaccgaaaaaagtttttgagattagtgatgaagacgaagatga

tgatgatgaagaagatgacgatggggacaatggagctgatgatgttgatgatgatgaggacaatggaaag

tcatcgaaaccaaagaagagaaggcaatactcaaaagtctggaacgatttcatagtgattaaaaaggtta

atcatttgggaaaaagtgaagaaagagctatgtgcaagcattgcaaatcagattatgcgtacaatgcaca

caaaaatggtacgaatacatatcgacgtcatttgcaaatatgtaagctcgtgcctagaaatggtgatatt

agtcagatgatggtgaatgcagaagcaaaattccaagctagaaagatagatcaatctgttttccgggaat

tggtagctaagacaattatccaacatgatcttccattttcttatgtggagtatgagagagttagagaaac

gtggaagtatttaaatgcggatgtcaagttttttagtcgaaacacagctgctgcagatgtttataagttt

tatgagattgagacggataagctgaagagagagttagctcaacttcctggaaggattagtttaactacag

atttgtggtctgcgctcactcatgaaggttatatgtgtcttacagcacattacattgacagaaattggaa

gctcaataacaagatcctagtttttgtgcattccctccaccgcatacaggaatgaacttagcgatggaga

tacttgggaaattgaaggactggggaattgagaagaaagttttctccattacggtagacaacgcgggtaa

caatgacacaatgcaagaaattgtaaaatctcagcttgtactccgagatgatttgttgtgtaaaggagag

tttttccatgttagatgtgcaactcacattctcaacatcattgttcaaattgggctgaaagggattggtg

atacattagaaaaaattagagagagtatcaagtacgttaaaggatcagaacatcgtgagatcttatttgc

aaagtgtatggaaaacgttggtattaatctgaaagctggtttgttgttggatgtggcaaataggtggaat

tcaacattcaagatgcttgatagggctctgaagtatcgagctgcatttggtaatctcaaagtcattgatg

ccaaaaactacaaatttcatcctacagatgctgaatggcatcgactacagcaaatgagtgattttttaga

gtcgtttgatcaaataaccaatcttatatcaggttcgacatatccaacttcgaatttgtattttatgcaa

gtttggaagatccaaaactggttaacagtgaatgagtccaaccaagatgaagttatcagaaacatgattg

tcctaatgaaagagaggtttgataagtattgggcagaagtaagtgatatctttgcaatcgctacggtatt

tgatccgaggttgaagcttacacttgcagattattgttttgcaaaactagatatcagtactcgtgagaaa

agaatgaaacatttgcgtgctcaactacgtaaactttttgaagtttatgagaacaagtccaatgcggtat

cacctacaactgagtctcgtgaagatgttacacatgatgatgaaactgcaaaagggaattttagcaatta

cgatgtaagtttcttatgttttgtttttgttagttaatatgcagttttttttatgaaaaacagcaagtca

ttagtttttatgttatgttattttgatttcttatctttgttataggatttctttgcttttcgcaaagcaa

acgttgttgccaatggaaagtcgacgttggatatgtacttggatgaaccggcaatgaacgtaaaagggtt

tgagagtttagatattctcaattattggaaagataacggtccacggtttggaaaattggcttccatggct

tgtgacattctaagcattcctattacaacagttgcgtccgaatcatcgtttagcatcggaactcgagttc

taagcaagtatagaagtcgtcttttaccgagaaatgttcaagcattaatatgcagtcgcaattggttaaa

aggttttgaatcttatgaaaatggtaagtaagttgtttttcataagttttatagtcacacaatcttagtc

tatgaattaacattgttagtttgtatgatatttgtagaggagtatgaaaaatttgatgctgaagatgaga

ctctaccttccttccagtcgcttgtggatgatgcagatggatgttgagttgcagattttattttaagttt

gatattcatgtttggatttgagtttaagttcatgtttcagttatcatgtttgcgtttaagaagttgcagg

tctttctcttgacttaagttcattttctgtttcacatttgagtttaacttgttacaattaaggtttgaat

atgagtttaagttcagattaaggtttacaaatgagttgcaagatgagacacgacattgttccttttggga

tattttggatttgagtttaagttaaggttctgatagcacgatttcgcagcttagagctatctcagattat

cttgcttcagccaagcaggctgcagttcttcaggttcttttacctgcaaatgtacaaactgaaatcgatc

agatcggagcgaaactggattcttctgtagctactatcactgagaaatcgactagtaactccaatcacat

aagacatttccttgattctgtgtaacaaactcctcctaacagtctatctgttacagaatctttggatttt

gaaaaacaaatgtataatagtcatttctgtactcgcaggagagtggcgcttatcgtagtttcaatcgtca

tgcttgtggtgacttttcttggtctcggtaatgttttcatctttgactacatgtttctcaactatcaact

aatgtattgaccttagtgatcttgttatcatttcttctcctgcagtttcttcgatttgtactgcagacac

atgtgtggcaatgagcgaatgggttgagagaccatcaagcaatacggctctggacgagatattgccttgt

acagacaatgccacagctcaagaatccttgatgcgtagcagagaaatgcccgcgggccgacccgtatatc

cgctgggctttgcgggccgggcttggacactgataattttgtctgcggtcctaaacgggccaacccgtcg

cggcccgaaaaaagaaagcccactgcggggcgggctagatgggcgcgggccggcccgtctgacacctcta

>At_ATHAT10 Arabidopsis thaliana

taggcatgaccaaaatctaaccgtaaccggaccgtttttaaaccgtagccggaccgtaaccgtataaaac

ggttaataaccgtaaccgttaaccgtaaaaatatattatttacggttaaaaatatatactaaccgtaacc

gttaattaaccgtaaccgtatcaaaaccgttggttaaccgcacggttaaccgtamscttaaaaatatgaa

agctgaccaagttgcattattattaaaaagctggtcattagttttgcattattaacaatgataaatgatt

ttaaaaaatcctcaattctcataacccaaagaagtagtaaagttttttcaaaaccgataattgattgaca

agagaaaaaatgtgattaggaagagaaaacgagagagctaagtgtgatgtcatcaaatgaagataaaata

aaataaaatttatgtggcaaaacgcacgagcacttgaatgtagccggattgaaataaaataaaatttcaa

tctttaaaattttatgtggcaatccgttagttcatttcaatctttaaaatttatgaccaagtcaaatcct

acgtggcaaaactgcagaattagatcgttaaagacttgagcttgaccaagttttggaatgtagccgttta

tgaccaagtcatcaatgtttgtttaatttaaaaaatttcaaagattttttccataaatatagtaaccagt

ttttctataaatattgtttccattgttccatatgctaaaaatttctaaaataaagattattcttcttgct

tcatccaacgttaaagagatttttttttcatcatatttttgagctttattaattttgtttaaaaatgtct

tccgattcatttcgatctaacatcggcacacccatagaagatgttcataactcaacaccagccatcaata

ctcaagaaagtgcaactgaagaacaagctaacacaagctcaggaaagcaacgtctgaatgcttccgatat

tttcaaggtacactttcgaaaagaaaaaattgatggaaaaatgaaagcatgttgtaattactgcaataaa

agttttttttggaaatcaggttcaggatatggatcatatagatatcatttggttaacaatcatcctgaaa

aatatggtggagaaactcaaaatgctacttctgctaatctttcgaattttcattattctgataaaagaaa

tcgagaagaattagcaaaatgtgttgctgttgaccacctttcatttagctttggtgagaaactgggtttt

aataattattgccaaaaagcattaaatcctcaagctaagagagttcctcgaactactcttactcgtacat

taaaatcactttatcgaaaaacaaaaaaaagatttagaatgtttgttttctggtttacctaataatgttt

caatatgctctgatatttggagtgatcagtggcaaattcatcattatatgggtattacttctcattggct

tgatagtgattggattattcaaaagagaattattgcttttcgagttttttatgaaagacatacatctgat

aatatttttaaattaataaaaataattttagaagaatataatttaacaaacaaaatattttctatttctt

ttgataatgcatccgctaatactgcttccattgatgaattaataaataattgtagtcctattttaggagg

aaaatattttcatgttcgatgcatttgtcatgtcttaaatttatgtgttcaagatggtttattggtttct

caaaataatcttatttctccaattaaaactgctttgaattatttatggggtcatccacaattaatgaaaa

aatggtttcggttttgtaaaatgcataatgtatcccccaaaaggttttctcgagatgttcctactcgttg

gaattcaacatatgatatgcttgttgattctattggttataaagatttattgtgtagttttattcaacaa

aattgcagttctttaactttatggcccactcattgggatgattgtaatgcacttttaaagttattaaaat

gttttaatgatgcaacgtttttattatccggtgtttattatcatactagtcatttacttttatatgaatg

tgttaatattgctgatgtgatgcatgaacatgaaaataatactgttttgtcatcatgcataactagtatg

agagataaatggttaaaatactatagagaaatcccacctcttaatttgcttgcatctgtttttgatcctc

gaactaaatttgatggtttatatgattacctcgtcgcatattatgatctattacatttgtctgatagtat

taatgttccaagcattatttccaactcaagaaaagacatagaaaatttgtatgatgaatattatagatta

tatgagcatttggtaccacaaggaactgaatcatctctacaaaatgatatgtcatcaacttctacgttaa

gtcttgcagaaagaatgcggcgacgtaaaaggcaacgaccatcacaaggtaataatgctgaacttgaaaa

atatttatctactaattttgagtttagtgatgcagatgcaggtaataactttcaaatcattcattggtgg

aagagtcaccaatctcaatatccaattttagccatgatcgcaaaagacgttctttcttcacccgtttcta

cagtttccgtcgagcgagcttttagcatgggaggtcaaatactagatgaaactcgatcaagaatgagtcc

agattctcttgaagctcaagcatgtcttgacgattggaccagagccggatatagacaacaagaattcctt

cgagagaatgaagaagaattagaagatattgatagtgatgtatcatcaacgagatcagaagatagtgatt

aaagtttattttttattacttgttttagttagcttaataaacattgttataattaataaatatgaatttt

ttggttacttcatttttctaattacataattatttcacttaaatagtaccttaatcatttcgtagtttat

ttatttgctgtaaaaaaatagtttatataattattcttttaaaaagaaaattatttaagaaaatataata

ttttgcttcatatattaattgtattataattatcaaataaccgtaactgtatttaaccgtaaccgtttta

accgtatattcagcggttaaggttaaggttaaagaaaatttgtaaccgtaaccggtggttaataaaccgt

aaccgtaaccgtggccatgccta

>At_ATHAT2 Arabidopsis thaliana

taagcctgggcgtttaatccgaatccgataaaccgaaccgcatccgaactgggaaaatcggattaaaaac

cgaaccgaatccgctcaattaaccgttcggttattgatttctatatccacggttatcggattttatccga

tccgaaccgaaataatatggctatccgaaaccaaccgaagaacttcttctatacactactctttagcctt

cgaagtctggatccacttcccttatgatgattatcaaacaatatgattcgatccacttccctaatcttct

tgattttcctagcaatacgggatccacttcctctagattataaccataaaaccctaagctctttcaatat

ggaattcttaagctctctttcgtcacaatcgcctccatcagaagcatcaagcaatcgatctcgggtatgt

gtttgaacttcaattcttcttcttcattagctatcgtctttgttctttgattcttcttcttcattagaaa

aaaaattgaaactcatttaaattgatttagtcatatagctaatgataaattctttgtttggattttaaaa

atttgtgtatgttggagttaaattcttagtcgattctaatcctcttgttgtgtcaatggatgtttgttga

gtagcaagagtatatttttgattgtgagagtatagttttgagtagtgaacgtatatttagatggattctc

attattcttatgttaaaacttctcattattcttatgtttagatggattcatcaccatttccaagcaacag

tgatgtggggaatgagaaagtgactgaacctgaaagtgatgctgctaacaaaaggaaggatgttcaggaa

gatagtggagctacttcaaaaccacctaagacaaagaaaccaagtcaaaggtcgtttgtctgggaccatt

acacgagatttgaagataatcctaagagatgcaagtgcaactattgtcacagaacctacggatgtgattc

taaagatgggacttcaaacttgaagaatcatcttcgaatctgcaagcattatcaagcatggagtcagaaa

gcaaaacaaactgttttcaacaatcagggacatcttcagagtgggaaagttacataggagattttaagag

aagcttcaaatgagatgcttgtgctgggagaattaccactttctttcattgaaagcgcggcttggaaaca

tttatgtgaaaaggtaaacctctatgcgccacattctaggagaacttgtatgaaagacattgttaaaatg

tatgtgggaatgaaagcatctctgaagacttggattgcgactactaatccaagagtctctttgacaacag

acatctggacagctaaagctacagttgctagttacatggtgattactgctcactttgttgattctgcttg

gaaattaaggaagctaatcattgggtttaagtacattacggatcacaaaggtgcaacaatagcaaggatt

cttcttgagtgtttggctgagtgtggaatagagaagattttcactatcacagtggacaatgaaacaacaa

acacttctgctttgaagaaatttcaagaaacattcagcttaaggagtaatgaagcatttgttttagaagg

cgaaggtatgcatgtgaggtgtgctgctcacataatcaatttaattgtcaaggagggtttggttgaattg

ggtgatcatgtggcagccatacgcaattcagtgcagtatgtgaggtcttctacttcaaggtgtgattcat

ttgaccagaaagttgtctcaggcaagatgactagagggagtttaccgttggacatcaaaacaaggtggaa

ctctacttacttgatgctgaaaagggctatggagttcaggttggcatttgataaaatggaagctgaagat

aagttgtataatgactacttcaatgaagtagacgatggaaaagcaagatcgggccacctacaagagctga

ttggaatgcagtcgagaggttagtgagatttctgatcatattctacaactccactttggtcgtgtctgct

tctaattcagtagcttcttataagtgctatggtgagatagtgactatagagacgaatttggtgtcactag

gcaataattttgacagggatctgaaaattaaggctaaagctatgttaggaaaatttgttaagtattggga

tggaacaagaaacattaatgtttatctgatagttgcaagtgtctttgatccaaggaagaaaatgcagttt

gctaatatgtgttttgcaaagctttatgggaaagatactactgatgctaaagagatggctgaaaaggtca

acaatgttttgacttccttgtttaaggagtatagtagtcgttttcagaaaacatcaagtggtagtggtcc

atcatcacagtccactcagacttcgattactgctagccaaggtgagccctctgatttgatgtctgatagt

atggggtatgagaggatggactttgcttataaggagttggttgatgagattggggttgatgatggtaggg

acgagttagatgtgtatttgaaggagaaagtggagaatcctaaaactatcattggattagagtgggaagt

actttcttggtggaagctcaattgtggtaggtttcctgttttgtctgcaataactaaggatgtccttgca

atgcaagtgtcatcggttgcatctgaaagtgcttttagtaacagtggtcgagtcatagagccacatagga

gttgtttaactcattatatggttaaagtgttgttgtgcacagagcagtggatgaagaatgtgaatcactt

aggtaataaatctgttgttaccattaaagagttgcttgccgacattgttgagctagataacattgaaaaa

ggtaaattcatcccttatgctctatttcaattctctcttaatagttttaattgttcacagttttaatttc

ttgtttttcttgattacagagtttgatggcaactctgcttagaagactcttatcctcaaactttttaatc

ggctataaggtaaattgttttaaacttgatgtttttgattcatgtatttcatagattaagaagttaagta

ttttgttgtatcaaggttcaagaacatggtgaagtttgagaatgcggcaaagaagaagcttgaggttgct

gttgttggcttgttttcactctctttcacttttttttttactactttccaattgaattctgctacttctt

cactctttaagatgttatggtttacttttctttcaaaactttgatgtattggcatagtttatgcctcact

ttggtttcaaaaactttatggtttgctttgctatggtttcatttcatctactcattttttgcatagctcg

ttttttcggtttaagttcggattaatatcggttattcgggttcaatcggatgttaaatatcatattcata

ccgatccaaaatccaaaatatctaaaccgaaaccgatccgaattttataattatccatatggatattaga

atctaaatccaaaataaccgaaatccgaaacaaccaaaccaaatccgattcgataaccgaacgcccagac

cta

>At_ATHAT3 Arabidopsis thaliana

taggcctgggcaaaatacccgaacccgaaaatccgaaccgaacccgacccgtaagatccgatccgaatgc

actttggttccctgcctaatacccggtcggatctttctcttttgtaacttcggttctgggttcgggttcg

ggtaatacccaagaaccgaaagtacttttctcgatccgaacttatatacacacaatagcattaaccctta

cttgccttagcttgttcttcttcccaatttttgatttattaaaccctagagcgtatcaacacaaatcgca

acaagctggaaaccctaactcatttgtttctgccgtctcattcttcagagaagtcaagaactcgactcga

tccgaggtacttttcatttttctacttctgtgattttgcttttgtttctttgagtcactgattcaaatat

gtagtttataattgtaatttcgttttggttttgtatttctctgttgtattcttagttgatttcttctaat

ttatctgttttcattagttaaatttttcaatttatttgattctatttttgtatctgtggagaaaaaaaat

ctgagtttgtgtgagttttgatctccaaattttcatctcttaccaccaattgatctgttgtgcatcttaa

tttttttgaatataaatttacaagagcatcacttacccaaaagcatcgactccctcagattcaaattttg

gtgatttagctcaactttaggctcatctacaccacttctaactcaaacgttatattctattaccatgctc

tgtttttttttaagttataggactctgttttaagcatcgactttgtttcttttaagtaagcttgtaaata

taccttcatattttgatatataacattcacattttatcaaaataaatttgtccgactctcttttatatgt

tgacagttgtgtgtgtgtatgtgtttgatccaattgttttaaaattattaatagtaagttcactggatcg

tgaataatatagtttggtatgtatgcatccaagagtcttatagactattaattcattaattcctagtatg

catccaacgtgtgatagattgcattattaagtgtaactagactattaagttatagtttcatgctagtgct

gcaacgtgtgatagcttactttattaagtattattcttattcaattgttaaccgattattgtttatattt

tttttcttttgcagatggattcttcagcaaatccaaacaatactgaaagtaacacaaaaggaaacgaaga

tgagggtaggactcgacctattgataacagtaaagggaagagaaaaagcgatgtcgctgatgatggtgat

agtgctaacccttgtaagccaaagaagaagttgcaaacaaggtcttgggtttgggatcacttcacgagaa

aagatggtgatgatgaccaatgcaaatgtcattactacaagaggttctttggatgttcaacaaaatcagg

gacttcaaacttgaagaaacatttggattgctgcaagcattactcggcatggaaaggacgccagtctcag

aatgttattaatcaagagggaaatctacaaagtggaaaagtttctaaggaagtttttagagaggctacga

atgaaatgcttgttttgggacaactgccactttcatttgttgaaagtgttgcttggaaacatatatgcag

caaagctaatctctacaagcctcattcaagaagaacagctactagagatattattttaatgaatgtggcc

agaaaagcatctctgaaggatttggtttcagctaataaacgtagagtgtctcttacaactgacatttgga

ctgctcaagctacaggtgctagctacatggtaatcactgtgcatttcattgatgagtattggcgtttaag

aaagtttatcattggctttaagtacatcgcagatcacaagggagctacaatatcaagggttctacttgag

tgtttgactgaatggggaatagagaggatatttaccatcacagttgacaatgcaactgccaatactagtg

cactcaggaagttccagagagctttacaatcacagagagctgattctttggtgttaaatggagattttat

gcacatgaggtgttgtgctcatatcatcaacttgattgtgaaagaaggtctgcacaaattgggtaatcat

gtggaagccatccgcaatggagtgctatatgtgagatcttctacttctagatgtgactcatttgagcaga

aagttgtgacaggtaagatgacaagggggagtttgcctttggatgtcaaaacaaggtggaattccaccta

tttgatgttaacaagagcaatcaagtttaaggtggcatttgacaagatggaggctgaagacaagttgtac

aatgaccacttcttggaagtagtcgatggggaaaagaggattggaccgcctactacaattgattggaggg

aagtagagaggttagtaaagtttcttggcttattctacactgctactttggtcgtttcagcgtcaagtat

tgtttgctcttacaagtgttatggtgagatagtcaccatagaaaaaaatctgctgggaatgactcatagt

tatgataaagagttgagggaaaaagctgttgaaatgagggaaaaattcgacaagtattgggatggacaaa

agaatatcaataggatgttgataattgctagtgtgtttgatcctagacagaaaatggaatttgctaaaat

gtgctttgagaaactttatggtgtagacacttcagaagccaaggagatgtataattctgtttatgatgtc

atgaaggctatgctgaaggagtacactgtcattttcaagggaccaaacacacaatcatcacagtctaatc

cgccttcatctactgctgccagagatacttttgcatgtgagttagctgaagatagtaatgttgagtttga

aaggatggacaggtcatataaagagatggttaatgaaattagggttactgatcctaaagatgagttagat

atctatctgaaggcagaggtagaaaatcctaagactcttcctggaatggagtgggatgtcttgtcttggt

ggagactcaatagtcagaagtatccagttttatcagaaattgcaagagatgtacttgcaatgcaagtgtc

atctgtcgcatctgaaagtgcttttagcaccagtggtcgtcttttggagccaagtagaagctgccttact

cattatatggtagagactttggtttgccttgagcagtggttgaaaagtgaaatcaagctgagtgaaaaca

cttttctaacaaatgcacaacttcttgctgatatagaattacttgataagcttgagaaaggtaactttat

ctatgtagtttactagttttagttttagatgttgtttgaaaactctcttatgttctaacacaacttgggt

gttgattgaatctttttcagagtttcaaactgaagtgaacttggactaaaagactaatcaaggaaacgac

tacaaggtaacttcatcactccattttgttttcttttacttctgaacttggtttatagctcaatgttaat

atactagactcatgaatcaacgatcatttcgtatttgacttttacttggtttatggtttaatgtttactt

atggttttttatttttcaactgagcttatttggttttgatgtaacaggaacaagtcatgaagaatctatc

atgtttggatgttgctttggatttgaacttctttctttactttgaatgctttggatttgattctgaacga

tggttttatgtcttgctttggatttgaacttctttcttctatttggatggttttatttggatgttttgaa

tcttgctttttggttgaacgtgtgaaacttttattatgtcatgctttattgattttacttcaagtttttt

ttattacaaatttacaggttttatggtatatgtggttagtatcaagtatgttcgggtagttatgaactag

tatccaataatattcggttacttttcggattttcaatggaagtaccaatacctacccaaacccaaaatat

accggaccgaacccgaaccgaagttttcaaatacccgaacggggtctaaaattatagttccgaaaaatcc

gaacccgaaagaaccgatccgaacccgaaccgaatacccaaacgcccaggacta

>At_ATHAT7 Arabidopsis thaliana

taggcctgggcataataaccggacccgatacccaaaccgatacccaaactgaaaaagctgggttggggcg

ggttagggtaaaaggatattacctcattgggtttggttttggttaacccgtgggttcgggttcgggtagg

gtattactcattacccattccgggtacccatacacccgattcttgtgcttatatagatatccctatatat

catatataacctatacacctatacaccactacttactttttgtcgattcagttttcttcacgaatccctt

gaatatcggcttctcttgttaaattctctttgaaccatattctatcaaactggaaacccttgtcgaaaca

ctctcggcgacctcaccgtcaccgacgatcctgttaaacccaccacagacagctcaatcgcgaaatcatt

ctaaggtaagtacgttcatgcaatttcaattctaggtttcgattattgatctcaatctctgattagtttc

aaactttcaagtttcaattctcgatttctcagttcctagtttcgattgttgtttgcttgtttcaattcaa

ttctcgatttctcagttcctagctccgattgttgtttatgtagatggcgtcaaatgaaactctacctcca

aatgattttgatgatgttgaattaagtggaaatgacgatgatgatgttgttcaacatactcctacttctg

gaaataagaggaagcgtaaagagaaggaaacaggagatgaaggtgatggaggtaatagttcaaagaaaaa

gaagacaagtagtcgatcctatgtttgggatcacttcagtagaaagaaaggtaatccaaacaagtgcaat

tgtcattactgtgggaaagagttggcttgtccttccaagtctggcacctctactttgaaaaaacatttag

aattgtcctgcaaagcattcaaggcatggaaaagtactaatacagaccaaactcaaactgttattggtcg

tgatggtggtgatggtagtcttaccatgtacaaggtctctgaattagtcattagagaagcttctattgag

atgctgattttaggcgaattaccattatctttcatagagagtgtggcttggagacatttctgttctaaag

ctaaatcgtataagcctgtttctcggagaacaactacaagagaaattgtgatgctttatgtcaagaagaa

agtggctatgaagaagattctagggaagagccaggagaggatgtcactgactacagacatttgggtttca

aacaatactggagagagttatatggtaataacagctcattttgttgatgttgattggaagttaaagaaga

tgataatcggattcaagcatgttactgatcataattgtggaactatttgcaaggttcttctggaatgttt

ggctgagtgggatataaggagaatcttttgtatcacagtggataatgctacagctaacaacactgcattg

actaaattcaagaaaacgatgaagctgattggggatgatgcattggtactgaaaggggaatatatgcaca

tgcggtgtgatgctcacatcctgaatttagttgtaaaggaaggcttgactgaagtggatgcaagtgtgac

tagtattcgcaatgggattcagtttgtgaggtcttcaacaaatagactcaaatcatttgatttgcgttgt

gatgctggaacgattagtagaggtagcttccctttagatgtgaagacgagatggaactctacttatctca

tgcttgaacaagctgtgaagtttaggattgcttttgagaaaatggaggctgaagataaactatataatga

ctattatctggaaaaggtggatggagaaaaaaagattggaccgcctatgtcaagtgattgggatgcagct

gagaggttaattcaaatccttgctatattttacaagtcgacattggtgctttcaggttcaacttatgtta

cttctcataagatgtacaatgagataatcaacatggctagaaacctaactaccttaaacacagacacttt

ttttgatgagcaactaaagaagaaagcgatagcaatgttaggaaagctgaagaaatattgggatccgttt

ggtgaaggagtagaaatgaataggttggtgatggttgctactgtttttgaccctagaaagaagatgaaat

ttgttgagctttgctttggaaagatgtatggtttaggtagtgtggaggttgttcttctttctgattcagt

gattcaaatcttgaaggatttgtatgatgagtatagcagggcaaatttgttgagaataaatggaggcagc

gattcaatgccatcttctcagtcacaaggatcatggtctcagtctcaagagcaagatagaagtggagctt

atgagagaacaatcaacaaaactggtattcaattagaagatatggagaacttatttgatgagattgtgaa

agaaacatgtattcataaatcatctaatgagttggatttgtatttgaatgaggctgtcgagactccacat

cttctaatgggaatagaatctgatgtgttagattggtggaagctcaatagtgggaagtttctagttctgt

cgctaatagctaaggatatatttgctatgcaagtatcgtcggtggcatcagaatctgcatttagcactag

tggtagagtgttagatccatttagaagctgcttaacacattacatgatagaagtcttgatgtgcacagag

caatggttaaagtctgagatcagtatcaatgaaaaaggattgtcaaccattcatgaattgcttgcagatc

aagtggacgaagatgagcttatgagaggtaatctattccctttaaactctctttaaattctggtttacca

attatagcttacaagtttgcttatagtccgtgtttcttaaattgttcatgtgatgtagagtttaaacctg

aattccacatccatggttttgagtaggccgagttatcgtttcagccttcaggagacaaaaatgcaaaaca

aagtcatcacctcatgtcctcatcaactatcgtttaaactttaaacttctacttctatgtttttcatttt

cggattgttgttttgcttttcaattactcttgtttgagatttgagaacgttctatttgtcatatttgtgt

tcttgtttgtggtttgccaattaggcttggatttggatgctagttttgttgctttttcattctgtttttt

tttctcctcatttgttacacatgattagaatatcggtttttcgggtttatgggtaacttttacccaataa

tacccgaaccgaaggaaacccgtttttttccgggtatttaccggttcttatattgattacccaaaccgac

ccgaacccgggtgtacccgaaccggaccaaacccaaaaatttcagtttaccctattgggtaataatttca

ttacccattcaacccgaacccgaacgggttctacccgaacccgaacggattacccatatgcccaggccta

>At_ATHAT8 Arabidopsis thaliana

taggcctgggcataaaaaccgtacccgaatacccgaaccggaaccgaaccgaaaaaaccggttcggggcg

gatacaggtacatcgattttacactgatgggtcttttattagtgtacccgtgggtatcggttcggttcta

ggtattacccgatacccgaaagggtaaaccgaaaactcgaaccaaatctcaatacccttaatcccttata

gcattaggtttactggtttagtcgattttaggttctttagggcaatccaattaggcttttgtcgatttag

cttctccctattactcgtttcccgattctagatctctactctttcctttatcaatcatctgtttttattt

cgaatcaatcctggaaagtcaagtgaatctcctcacaccgtctttggtcacttctccaccgtcggagttg

tctagaaacaagtctgaggtaattttttgtgtcttttgtgattttctgtagattgatgttgatggaggct

gtgtcttttgtgattttctgtgtcttttgtgattctagatctttactttgtgtcttttgtgattttctgt

aaattgattagaattactcataagagtcaaaatataaatgatgttgaattgtcaatactattgatggaag

cataagaatcaaaatatatactcaatagtcaatggaagcacaccgtgagctaacatgtttgtcaaatcaa

ttgttatatgtttgtcaaatagaggtttgctttcaatcggctgaaggtgttaagtctgtgaatgtctgct

atgttaaataagttcgtgaaagtctgctatttaaggctgtgaatgcctgttagtttatgtttggttttac

tcattcgtttaagtttgaacttattagtggttgtattgaatttgtgcttgaattgaatcttgttgcagat

ggcgtcttgtgatgaaccaattcctgagaatgatgaaatgacagaagaagagattgaaacaccacttctg

gaaaatcaaacaagaggaaagataatggaggggtaggtggaagtggttctaagtcaagcaagaagggtag

gtcttgggtttgggatcatttcaccaaactgccaaatgatgcagaccattgcaattgcaattactgtggg

aaagagttggcgtgttcaaccaagtccggaacctccactttaaagaagcatcataaatacttgcaaggca

tattaggtatggaaagctgctaatgttcagaatactcaaaccgttttaactcctgatggtcctagtgggt

gtatgactattagtaaggtcagtgaatctgtttggaaagaagcaactaacgagatgttagttttagctga

gttaccattagcttttgtagactgtttagcttggagacatttttgctccaaagttcaaatgtataagcct

cattctcggagaaaagctagaagagagattgtagagacttatgttagtaagaaagctgcgatgaagaaga

ttatagagaacaataggcagaggttgtcattgacgacagatatatgggtttgtcccaatactgcagctag

ttatatggtaataacagctcactttgttgatgcttactggcagttgaggaaaatgataataggattcaag

catgtctgtgatcataagggtcaaacaatttgtgatactcttctagcttgtttagcggagtggggtataa

agagagtattttgcattactgtagataatgctacagccaatacttcagctttgactaagttcaagaaaga

aatgatgaggctgaatggaaatgatgcattgatattaaaaggagagtacttgcatatgaggtgtgctgct

cacattttgaatctggttgtaaaggaaggattaacagaggttaatagcagtgtggaggctattcgcaatg

gaatccagtatgtgaggtcttcgacaccaagatgtcaagaccatatggaattcgacttatctcatgttag

accaagctttaaagttcagattggcatttgagaagatggaggctgaagataaaccctataatgattactt

tgcagagaaagaggatggaaataaaaggattggaccagctgtgacaaaagactgggagaatgttgacagg

ttagttcagattcttgagattttctataagtctacattggttctttcagcttcaaactatgttgcatctc

ataaactctacaatgagatagtctccattactataaacttaggtgcattgacgtatgatgatgatggttt

aaaggacaaagccgaagctatgttagctaagttggctaagtatagggatgcatttggggaaaaagtggaa

tttaataggcttgtgatagtggcaagtgtgtttgacccaagaaagaaaatgaaatttgctgagttatgtt

ttgaaaggttgtatggtcaaggcagtacaaaggctactcagctccaagattcagtttataacatcttgtc

tgatttgtttgatgagtatacaagaaacaatttattgaacaaaagtagtactggtactactgggtcatct

actcaatcggaatggagtcaagatcaagttgtgaatgaagattcagagagaccagttctgcgaaatggat

ttttatatgagaatatgaaatatgaatttgatgagattgtgaaggagacaggattgcataatacagctaa

tgaggtggataggtatttgaaagagcctgtggagaaaccaatcattctaaaaggtacagaatatgatttg

ctaaatttttggaggatcaataatgggaagtatccggttctctcactcattgctaaagatatacttgcaa

tgcaagtgacattcgtagcatcagaatctgcatttagcacaagtggtagagttgtgaatccattcagaag

ttgtctctcacattacatggttgaagtgttaatgtgtcttgagcaatggctaaaatgtgagatccatctc

aatgaaagaggcgtctccacaataaaacaactactttcggagatcactttagaagatcatcttatgagaa

gtaagttcagattctcttctctcttcttagttataacatcatgatttatgattctaattcatgttctttg

gttttgtgtagagcatgaactaggtgaaacagatttcgatttcaacgtggaatgaaatacaaaggttagt

gagttagtcagtagtgattcaatgagatattggtttacttgtgagttgtgatttgtgattgtgacgttgg

tgttggtttacttgtgagttgtgatttgtgatgttggtgttggtttacttgtgagttgtgatttgtgacg

ttggtttacttgtataggtttgtcaaggggtcacgatttcacggagagggtaggagggagtgaaagacat

tatttatgctatctagtttttttctttgttggacttgtattttgttcaagtgagttgtgacttgaaattt

ggaactgttgtatgcattttatgtctttaacagtttaaatttggatgattggatttggatgttttactct

agttaattgcttcacgttatgttgttttttgaaatgctaagtaactttggttcaagtttttatttttggg

tataatgtagtgtaaacagggtaaaactttggttcaatggtgtattctggtataaatatccgaacccgaa

ccgaaaccagcaaaaaccgattggatttcgggtttttagcggttctcaaaactgatacccgaaccgaccc

gaacccgaagagacctgaaccgaagctgaaccgaggttttttatttaccctattgggtcctagaattgta

tatccgacatacccgtacgcgaccggatattatccgaacccgacccgcatacacgaatgcccaggtcta

>At_ATHAT9 Arabidopsis thaliana

ggatgttaatatgggcttataatttagggctggcccgaatccgttaaaaaaaatataagccctaaccctc

ataaaattaaaaaaaagattttgggctaagataaagctatacttaaatttttatgggctaaaatagggtt

ggccctattacacttttattattctctgtcctttttttttcttttctctttctcccaattcccaaacgcg

tcttcttcaactcttcttcttcaatctcttcttctttgatttcttcttctttgatttcttctctgaaacc

ataagagtggaagatttgatctgaatatatagagaaacaaaagcttggtgaaatagcaacaaccctaatt

tttcaatccctaaaggtgaagtttttgttcttctttttctttgattctactctttttcgtcacaatagca

aaatatttgatctgggttggcattttgattgaagaattttggtgggttcgtgttagattttggtgggttt

aggttggtgttagattttggtgggtttaggttggtatgacaaatatcgtgtattgctatctcatgaaaat

acagctttatgttgatgtaattaaatttttttatgtagtatggatcaacaatcactggaaagaattgctc

ttctagaagctgaaaatgaacgcacggagatggagatgaatgctgaagttgaaactcaagggcagaacca

aacagaaagtgtcactcaaccatgtcaacgagctaaacggttacgtaaaaaacagagagctttgtgttgg

gatgaatttacatccgtaggaatagaggaagatggaaaagaaagggctagatgtcaccattgtggtatta

agttggtagtagaaaagtcatatggaacatcgactatgaaccgccatctaacactttgccccgaaagacc

tcagcctgaaactagacctaagtatgatcataaagttgatcgagagatgacttctgagataataatatat

catgatatgccttttcgatatgttgaatatgaaaaggtgagggctagagataaatttttaaacccggact

gtaagcctatatgtagacaaacagctgcactcgatgtgtttaagagatttgagatagagaaagctaagtt

gatagatgttttcgctaaacacaacggtcaggtgtgtttaacagcagatttatggtcgtctcgaagtaca

gtgacaggatatatttgtgtaacttcacactatattgatgaatcatggaggctaaacaataagatattag

ctttttgtgatcttaagcctccacacaatggtgaggaaatagctaagaaagtttatgattgcttgaaaga

atggggtttggagaaaaagatattaacaatcacgctggataatgcttctgcgaacactagtatgcaaact

attcttaagcatcgacttcagagtggtaacgggctattgtgtggaggaaattttttgcatgtgaggtgtt

gtgcacatatattgaatctgattgtgcaagccgggttagaattagcgagtggtctgttggagaatatcac

agagagtgtaaagtttgtaaaagcaagtgagtcaaggaaagattcatttgcaacttgcttagagtgcgta

gggatcaagagtggagctgggttgtctctagatgtctccactcgttggaattcaacctatgaaatgctag

caagagctttaaaatttagaaaagcatttgctattttgaatttgtatgaaagaggttattgttctttgcc

tacagaagaagagtgcgatcgtggagagaaaatatgtgatctcctgaagccttttaataccattaccaca

tacttttcaggagtgaaatatccgacagccaatatttatttcattcaagtgtggaaaattgagttgttgt

tgatgaaatatgctaattgtgatgatgttgatgtaagagaaatggctaagaagatgcaaaagaaatttgc

aaagtattggaatgaatatagtgttattctagctatgggagctgccttagatccaagactaaagttgcag

atacttaggtcagcttataacaaagtggatcccgtcacggccgagggaaaggttgatattgtgaggaata

atttgattttgctttatgaagaatacaagactaaatctgcaagttcttcgaactcttcaaccacacttac

tccacacgagcttctcaatgaatcgccacttgaagcagatgtgaatgatgtaagtataataatatatttg

aatatctttcactagttcttgtttttgtttaactaattgtgtgtgtatttgtgtatttacaggatctttt

tgagcttgaaagtagtctcatatctgcttcaaaaagtaccaagtcaactttggaaatctatttggatgat

gaaccaagattagagatgaagactttctctgacatggaaattttaagcttttggaaagagaatcaacata

ggtatggtgatttagcttcaatggcatctgatttacttagtatcccaatcaccacagtagcatctgagtc

tgctttcagcgttggaggacgggtgttaaatccttttaggaatcgtcttcttccccaaaatgtgcaagca

ttaatttgcacccgcaattggcttcttggatatgcagatcttgaaggtaaaattgtaatttgaagttata

aaacttatataaatcattgtcttaatttaacttgtatcattaaattgcaggagacatcgaagaacttttt

gctgaagaagataatgatgctacaaagatgacaagtagttcaggagttggagattctaacatatgatgag

aagttttttttgctcagtttttttttggacaaatatcaaacaatgttttatatttcagcaaaatgacaaa

aaacaatgttttatattttgtggtgttatagtgtttccatttgataataaacaatagtattattatctta

taattgtttttgtttgaaaaaaccttctctttaaataaataaaaaaacttaagggctacaaagggccaaa

aaagggctcaaccctattttaaatttttttagggcttaaaaagggctaggattcacttagggctgggctt

aaaataaatagggctgggttgggctggaaaaccctttttaacatcc

>At_ATHATN1 Arabidopsis thaliana

taggcctgggcaaataaaccgaaccgaatgtaccgaaccgaaaccgaagcaaaaataaccgaaccgaacc

gaaaattggaaaataaccgaatggatactaaattctataaccgaaagaaccgaaaccgaatggataccaa

accgaaatattttgggtaaccgaaaatatccgaaatataaatatattttcaaaaatattagttatttcta

gactaaataattaaaaatacttgaaaatatatataaaatagtaaaaatactcgaaaataaccaaaaaaaa

tatccgaaaatatccaaaaataaccgaaatatccgaaaatattcaaagcaaaataaccgaatggatacta

aattttaaaaccgaaagaaccgaaaccgaaccgaaaccgaaccaaactttcaaaataaccgaatggatac

taaactttagaaccgaaaaaaccgataaccgaatggatacgaaccgaaaccgaatggataaccgaacgcc

caggacta

>At_ATHATN10 Arabidopsis thaliana

taggcatgaccaaaatctaaccgtaaccgtatttcggaccgtaaccggaccgtttattaaccgtaaccca

accgtaaccgtataaaacggttaataaccgtaaccgttaaccgtgaaaatagatcagttacggttaaaaa

aatatactaaccgtaaccgttaattaaccgtaaccgtatcaaaaccgtcggttaaccgygcggttaaccg

tattgttaaaacyttaaaaatatagttgtaattgtgtataattctaaataaataattatttcactaaaat

agtaccttaaatcgtttaatatgcccactactacgatcgaagttactccactactctttttaccataaat

caaatcataagtagttcatattattattatttatactcttaaaattgtttcttaatgctcacaacatctt

ttctttagtattgtttttgtttggaaaatgtaattaattcaatcattataattatgaccattaatttgtt

tattcattggtttatttaagaaaaatagatagctgatttactataaatatttgaaacacataatcacgta

aaataaatattatttgatatatcactatttatttatttattttattataaagattaaaaaatataatatt

ttgtctcatatattaactatatcataattatcaaataaccgtaaccgtatttaaccataaccgtatttaa

ccgtaaccgtatttaaccgtaaccgttttaaccgtatatataacggttaaggttaagrttaaaaaaaatt

tataaccgtaaccggtggttaataaaccgtaaccgtaataaccgtaaccgtggtcatgccta

>At_ATHATN2 Arabidopsis thaliana

taggcctgggcataatacccggaaccgaatacccgaaccgaaaaacccgaaaaataaccgaaccgaaccc

gaacccgatttttagaattacccgaatgggtcctcgacttcactacccgaaaaaacccgaaccgaacccg

aaccgaatctaatacccgtgggtacccgaatacaaatgtaaacctaaaaatattagttatatatatatat

ttgtaatacctcaaacacccaaaattaaaattataaatccgaatatactagatntttttagtaattcata

tacatttggatatatttgggataaaagtaaccaatgttatagttttcttttagataatttaagtattttg

gttacatttggttatatttggataaaaagtaaccaatatatttacgggtaatcgggtatttcgggtagat

ttgggtatatatgagtaaaaaacccgaaycyaacccgaacccaaaaatattttgggtatttttggttatt

tttacatttttactaaaataaccgaaccgacccgaacccgaaccgaaaccgaaccgaatttttagaatac

ccgaatggttctcaacctcctacccgaatagacccgatacccgaaaaaaaagaaccgaacccgaacgggt

acccgaatgcccacgccta

>At_ATHATN3 Arabidopsis thaliana

taggcctgggcaaaatacccgaacccgaagaaccgaaccgaacccgacccaaaaaacccgatccgaatcc

gaacccaaaattttaaaatacccgaacggatcctaaacttcaaaacccgaacccgaacccgatccgaacc

tgaacccgatcaagtacccgaacatatccgaaatatatatatatatacatagaaataatttattagtaat

aatcatatttacaacaacttaaatgtctaaaatatttggattttaagatattttgtatattttagatatt

ttacctacttttagatatattcagatagaaaatactcgtgttgttttagaaatttttgagtattttcaag

taatttgaatatattttgatacaaaattttgagttttggatactccggataaacccgaatccgaacccaa

aaaacccgatccgaacccgacccgaacccgaagtcttaaaatacccgaacgggtcttagatttctaaatc

cgaaaaacccgaacccgaaatacccgacccgaacccgatccgaatacccgaacgcccaggccta

>At_ATHATN3A Arabidopsis thaliana

taggcctgggcataatacccgaacccgaagaaccgaactgaacccgacccgaaatacccgaacccgttct

acttcggttccggctaattacccgatcgggttctgttttcaaataactgtgggtatcgggtattacccga

accgaacccgatactcgtgggtactttttattacccgaacatatatatacatatgaggacatatatgcac

taataacttgaatttgtgacttgtttaatgatttgagttatggaacaagctcaaaacaaggtttacttct

tgttttatattattgtcttaaggaaaaacttgttataatttcagatttttcgggttttatggatattttt

atgtatgtttgggtatatttggacaaagtttgggtaagtagtactcaaacccgaaatagttttggttatt

tatgggtttttaaccaatacccgaattatccggacccgaagtgaacccgaaccgaacccgacccgaaatt

gttgatataaccatacgggtccaaaatttctaaacccgaaaaattcgaacccgaaaaatccgacccgacc

cgacccgaatacccacatgcccagggcta

>At_ATHATN4 Arabidopsis thaliana

tagggctgggcaaaaaaaccggaaccgattatccgaaccgraaccgacccgaaaaattggacccgaaacc

gaacccgaacccgaagatttaccctattgggtcctaaattcctctacccgaaaaaccgaatccgaacccg

aaaatacccgaatattttattcraaaatctaaattttacccgaaacccaaaattttacccgaaaatccgg

atatttacccraaaaatctggatatttacccgaaaattcgggtatcggttccaaaaaagggcctaaaaat

ctatatccgaaccgacccgaacccgattggacccgaaccgaaaccgaaccgatattttagatttacccta

ttggatcctaaacttctctacccgaaaaacccgtacccgatcggattttacccgaacccgacccgcttac

ccgaatgcccaccccta

>At_ATHATN5 Arabidopsis thaliana

tagggctgggcataaaatccgaaatccaaaaaccgatccgaaaaatccggctccgaaaccgaaccgaata

gtcacaaaatccgaacagataatgaattctcctaycygaattayccgaaccgaatccgatccgaaccgaa

aaccgaacggatatcygctaatccgaaataaaattatatatctaaaaatattagttatatttagttataa

tttatacccaaaaaatactaaataacaaaaatatccgaaataacyttaatatccraatttnttttagtta

tatttgttataattatacaatgttttgcaaaattaaaccaaattttagtaaaattaaacgatttttgatt

aaaattraacaattttttagtttttttttctcaaaaactgatccgaaaccgatcygaatccaacaccatc

caaaccgaaaccaaaccgaatagtcatataatccgaacggataataaactccctcatccgaattatccga

atcaaaaaaattcgaaccgaatccgatccgaaatcygaatgtccagaccta

>At_ATHATN6 Arabidopsis thaliana

taggcctgggcaaaaaaaccgctaaccgattaaccgaaccgaaccgaaccgaaaaacatggttcggttcg

ggtttggatagcttaaaataaccggctggatattatatgtcttagatttcggtttcggtttcggttaggt

tagtaaccgataaccaaaggataaccaaagcatatatgtatgatagataatatatcacatatattgtatg

tattgacacttaactatgttcttctctttattagtttgcaagttgaatttcgtgagttgtgaatcaggtg

ttttaatattatggagagtatggaccatatttgcatctttgtgagttgtgaatatgtctttttttacgta

cgttctatatcggttattggtcaatttttttggttgtttcggtttttagtcggttatttcggttttggtt

tatttatggttaccaagtttaatatccgaaccgaaccgaaccgaaagatatccgaaccgaaccgaaccaa

aattcctcaaatatccaaatggttcttatattactataaccgaaataaccgaaaccatgcgcaaccgaac

cgaaaccgaatggttaaccgaatgcccaggccta

>At_ATHATN7 Arabidopsis thaliana

tagggctgggcaaaaaacccggacccgatacccaacctgatacccgaaccgaaaaaatcgggttggggcg

ggttagggttttaagaaattacttcattgggtaaagttttagtaaacccgtgggtatcaggtcggtccgg

ggattatccgttacccaaataggatacccgagaacccgaacataaatttaatatcaaaaatattaattat

atttaatcttaatataatgtaattatctaaaattataattataaatctaaatatatcaattaattttaga

tctaaatacccaaaacaactaaactatttaaaaattatttgttatgtttgtagtaaaatatattcaaatc

taattaaatttattaaaaatttgttaatatttaaggtttgtttgttgggtacccgctatctacacataaa

atacccgtttccatttgtttgggtatttttagggcttgaaactctttacccaaaccgacccgaacccgag

tgtacccgaaccagaccgaaaccgaaaattctattttaccctattgggtagtaaatccattacccattta

acccgaacccgaacgggttacccatatgcccagcccta

>At_ATHATN8 Arabidopsis thaliana

caggcccggcccaaggctaaagctcatgaagctggtgctttaggcatcacaaatatattgtaaaagttga

gggcatcattccaaatttaacttgtggcatagtggtataactgtactaaaacgtccacgagatgactggt

tcgagttccttcttttcctttttttttggatttttcatttaagttttgtttgttagtgggcttaaaatta

tttttgctttaggcatggaaattgtttgggccggccctg

>At_ATHATN9 Arabidopsis thaliana

caggcccggcccagagctaaagatggtgaagctagggctttaggcatcacattttagtaacaaaatttgg

ggcatcaaattgtaaaattgttggtggtatagtggtcaacgntttgaataaccttcttctatgtgtctag

ttcgagtctaacttttgtcagaatttgttttttttttcaatacctcattattaatgggccaaacacattt

ttgctttaagcaacaaaaagacttgggccggccctg

>At_DRL1 Arabidopsis thaliana

aagcttgaattgaaatagtcttgtattgattccatttttgtaggcagtgtgtttaaaaaccaatggcgct

agttgtgatttgtgggcaaccttgtagtggtaagtcaatagctgcagtaactttagctgaaacattgaaa

gagtctgaaacgaaacagagtgttaggatcatcgatgaggcttcgtttcatctagaccgcaaccaaaact

atgctaacgtgcctgctgagaagaatctgagaggaagctt

>At_SIMPLEHAT2 Arabidopsis thaliana

taggggtgtcaaaatgggtcaaaattcatgggtcaactcaactcaactcaacccatgaaccctaatgagt

tgaaaattttgactcaaatgagttgatgggtcaaatgagttgatgagtcaattggtttgatgagtaaaat

gagttgggttgtaatggttaatggtttcaatggtttacccaattaacccatcaagttttgtaaaattgaa

ttaaaccaactaaaatctctaaaccaatgccaatttaagtttaaccaacacatctaaaccaatttaataa

aattaatatttttccaaatttcttaaatatacaagcgatgaaattgagaaaaagtaaactcgtaattttt

ccaccaaaaaacataaacccgtgattttcccgccaaaaacgtaaacccgtgattttcccgccaaaaacgt

aaacccgtgattttcccgccaaaaacgtaaacccgtgattttcccgccaaaaacgtaaacccgtgatttt

cccgccaaaaacgtaaacccgtgattttcccgccaaaaacgtaaacccgtgattttcccgccaaaaacgt

aaacccgtgattttcccgccaaaaacgtaaacccgtaattttcccgccaaaaacgtaaacccgtgatttt

cccgccaaaaacgtaaacccgtgattttcccgccaaaaacgtaaacccgtgattttcccgccaaaaacgt

aaacccgtgattttcccgccaaaaacgtaaacccgtgattttcccgccaaaaacgtaaacccgtgatttt

cccgccaaaaaacgtaaacccgtgattttcccgccaaaaacgtaaacccgtgattttcccgccaaaaacg

taaacccgtaattttcccgccaaaaacgtaaacccgtaattttcccgccaaaaacgtaaacccgtgattt

tcccgccaaaaacgtaaacccgtaaaaagtggaatccgtaaatatcctaagtttgatgataatgaattaa

taattattaataatgataattatttattattgttttataataataattaattaaattattacttaaatgg

gttaacccatttaacaactcaacccatcaaattaaatgagttatgggttgacccaacccatttaacaaaa

tgagttgggtcaacccataactcatttaaccctaaactcatttgattatgagttgagttgagttgggtta

cccattttgacaccccta

>At_TAG1 Arabidopsis thaliana

caatgttttcacgcccgacccgaaccgcccggtccaaccggttaaaccacgacccgagcacttctccggt

ttgggtttagtgctaaaacccactatctataaaaccaaaaaaacccataaaaacccgctattaacccacg

acccattgaaccggttgaaccgactgggtgtgggttttaaaaactatagttgattttggaacgaatatag

ggtttttggaacgaatatagggctttttcatagttcataatttcttctcaaacggcgactcaatctctcg

tctacactagccaaacaaaccagttagtttttctatctctcgttttaagtttgaaaatcctatttgtctt

tatttgtgagcatcatgatcattttatattgtatattgatatagtgatttctatactctcgtggcctctt

tgtcttggctccactgaatcaaatataatattcaaagtgtttttgcatcaaaataataattcagcttcat

gtttgatgtgcatgtctcacagatatttgtttgtttttttgtgtgaatttaataggtaatggaaacagaa

cacgatgaaaattatgaagatatagctgctgcaaaccgttcaatccgaggaaaaagtgatattgcttggt

cctatgtaattcaatcaaaagacgaaaaaggaaaaacggttttggaatgtgcattttgtcataaaaaaaa

aagaggaggtggaattaataggatgaaacaccatcttgctggcgtgaaagggaatacagatgcatgtttg

aagatttctgcagatattagatttaagatagtgaacgcattgaaagaagctgagaacaagaagaagcaaa

atattgttttggatgatacgaacttagacggtccagaaattgaagatgttgatggagatgatatccgagt

tgatgttcggccaagtcaaaagaggaagaaacaaggtattgatctacatgattattttaaaagaggtgtg

catgatcagacccaaccttctataaaagcatgcatgcaaagcaaagagaggaatacatgctgtggacatg

tctgttgcattgtggttttatgacgcttgtattccgatgaatgcggtgaattctccacttttcagcctat

gatgagtatggtagcaagtatgggacatggatatgtgggtccttcataccatgctctgcgtgttggattg

ttgcgtgatgctaagttgcaagtttctttgatcattgataagtttaaaagtagttgggctagtactggat

gtactcttatggcagatggatggaaggacacaagacaaagaccactgattaacttcttagtttattgtcc

caagggaattacatttcttaagtcagttgatgcttcagatatctatgcaagtgctgagaatttatgcaac

ttgtttgctgaacttgtgggaataattggttcagaaaatattgttcattttgtgactgatagtgcaccca

actacaaagctgctggaaagcttcttgttgaaaagtttcccaccattgcttggtctccttgttcagctca

ctgtatcaatcttattttggaagatgtggcaaaactaccgcatgttcaccatatagtgcgccgtatgtct

aaggtaaccatttttgttttacaaccataagccggctttaaattgggtgagaaaaagatcgggttggaga

gagatcattcgtcctggagaaacacgttttgctacaactttctcatagctctacaaagtctctatcaaca

taaagaagatctacaagctttggttacaagtgcagatcccgaactcaagcagcttttcaaaacatctaaa

gcaaaagtagctaaatcggttatcttggatgagcgaatgtggaatgactgcttgataattgtcaaggtta

tgactcctatcatccgtttgttacgtatttgtgatgctgatgagaagccttctttgccatatgtgtatga

agggatgtaccgagcaagattaggaattaaaaatattttccaagaaaaagaaaccctctacaagccctat

acaaacatcattgatagaagatgggatcgtatgttgcgccacgatcttcatgctgcagcgtactatttaa

acccggctttcatgtatgatcaacctacattttgtgagaagcctgaggttatgagtgggttgatgaactt

atttgagaagcaaaaaaatgacagcaaaacaaaactttttcaagaacttagggtgtatagagaacgtgaa

ggaagtttttctcttgatatggctttaacttgcagcaaaacctctcagccaggtaaattattaaacttga

gttaaacttgcattttattatctatatatataatataattttatttctatttagatgaatggtggagata

ttttggtcatgacgctcctaatttgcaaaagatggcaatacgaattcttagtcaaaccgcttcttcatct

ggatgtgagcgcaattggtgtgtatttgagcggattcataccaagaagcggaatagactagagcatcaac

gacttaacgatctcgtctttgttcactacaatttacgtttgcaacataggtgattatcattattattttt

tgtttcttaatttagcttagtttataacaagttccatgtttttaatgtacaggtcaaaaagaaaaagatc

atatgatcctgttgactacgaatctattgataagacagagttttgggtcgttgaagaagaagaagcaggt

gagcttgaatatgatgaattagagaatgctcttgctgaggaatatcccaaagatcttgaagacacaaatc

ctgaaacatcgaatggtatcttaaattattgattaaacttttcgaattgacttttagttatagataatat

gttgactaactaaacgatatgtctttgatgttttagattttgatgaagacttcacattgccgcctgaaga

ggatgtatggaatgatgaggaagacaatcgagattagttgttttgaatttgcgatgttttattactttat

ttctgttattgacttattggtgatctactagacatattgttattttagtattttaaaggttttatggatt

taaattttttaaactttatgtattgttatacttcttattgttattaaacatggtattattattattttta

tatataagttatataacataaactatataatctctatatatatcaaatgcattaaatcagttgacccgtg

gtctaacccggttgacccaatgacccggtgacccagaaggtagtccggttcactgtccgggtcgggcgtg

aaaacattg

>At_TAG2 Arabidopsis thaliana

tagaaccgtcaattgggcatctgaccatgggctaagcccagtccacaacattatggacagtccatggtcg

gcccgtaaagaaaacgggttaaatggtctaaagaccaattatgtccacgggtttaatgggctagcccaca

cggtctatggttggcccatcaagtttctcgacacatcaattttcttctcttctctctccctcggcgacga

agctccctctctctctttctcgcttcgacctctctctcaactcctactccggttagttctactagaatat

gaatccgttgattagaataagttctctaagtccttatgcaatttcatcgctttgttcttttctattcctt

aattgtgattctgcatatgctttctttgatttcagcttgtgggtttgtccaaatttcacttgtttgctat

tctttgttattctctctctcccttcctttgatgccaaccatgtgtttgatggaatgcctcacagaatatg

tagagttttgttttggtagagcttgtaattacttgtgtaaagcttggtgtttatgtatggagctaagtgt

atgaacgtttagtgtgtgtttgctaatgttaatgaacgtttagagcttgtaattaagtaatcgtttctca

ccacaatccatcacttgtttgaccaaacgtgcaaatattgtgagcagacttatcacttgaatctgagacg

aaatggtactaataccatgaatcgtcatatgagatcttgtgaaaagactcctggaagtacacctagaatt

agcagaaaggtggatatgatggtctttagagaaatgattgcagtggctttagttcaacataatcttccat

attcgtttgtcgaatatgaaagaattagagaagcctttacatatgctaacccttctatagaattttggag

cagaaatactgcagcttttgatgtctacaagatttatgaaagagagaaaattaagcttaaggaaaagtta

gctataatcccgggtagaatatgcttaacaacagatttgtggagagctttaactgttgaaagttatattt

gcttaacagctcattatgtagatgttgatggtgttctgaaaacgaagatcttatctttctgtgctttccc

gcctccacattctggagtagctatagcaatgaagcttagtgagctgttgaaagattggggaattgagaag

aaagtttttactttaactgtggataatgcttctgcaaatgatactatgcaaagcattttaaagagaaagc

tacaaaaagatcttgtctgcagcggagaattctttcatgtgagatgttcagcacatatcctaaacttgat

agttcaagatggtttggaagtgatatctggagctttggagaaaattagagagactgtgaagtatgtgaaa

ggatctgagactcgagaaaatttatttcagaactgtatggatacaattggaattcaaacagaggcaaatt

tagttttagatgtctcaacccgctggaactccacttatcatatgctctcacgagcaattcagtttaagga

tgtattgcgtagtcttgcagaagtagataggggttataagagtttcccatctgctgtggagtgggaaaga

gcagaactgatatgtgaccttttgaaaccatttgctgagattacaaagctgatttcgggttcgtcttatc

cgacagcaaacgtgtattttatgcaagtatgggcgattaaatgttggctgggagatcatgatgattctca

tgatagggttattcgtgaaatggtggaagatatgacggagaaatatgataaatattgggaagatttcagc

gacattcttgcgatggctgcagttcttgatccaagattgaaattctcagctttagaatattgttataata

ttctgaatccattgaccagtaaggaaaacttgactcatgtgcgtgataagatggttcaactatttggagc

ttataaaagaaccacatgcaacgttgcagcaagcacttctcaatcttcacgaaaggatatcccatttgga

tatgatgtaagttgctaattttgtgtgtttacagtattgttcttgtatgtttgtttgttgtgatagttag

tggctatgaaaatgcattcttatacttagtgagaatgggttgtctgttgtgattgttgaactatacggtt

ggtcatattttcttttgttggtcatatttcaggggttttactcttacttttctcaaagaaatggaactgg

aaaatcaccattagatatgtacttggaagagcctgtgttagatatggtctcttttagagatatggatgtt

attgcatattggaaaaacaatgtctcgcgttttaaagaattgtcatcgatggcatgtgatatcttaagca

ttccgatcactaccgtggcatcggaatcagcttttagtataggtagtcgtgtgcttaacaaatatagaag

ctgccttttacctactaatgtgcaagccctcctatgtactagaaactggtttcgtggatttcaagaagtt

ggtaatattcacatcttgaactcgtttataatagtaaacttatatggcttttggactgcagcttttagat

taatagctcggtttttcttattgtagaaactgatgagatccaaggtcaagaggacacaaacaccatcctt

gcatgaaagtttcaagactgggaagaaacaggtacatggctgttctcttaaactctgagcctatggttat

gtgtttattattctcagtctgtgacttagaagcttatgtaatcgttttttcttttctttgtttctcataa

acaaaggtttttgcttaagaaacaaggaaaaatagaaccatgtaagacacaaacaaagctccaaacagta

acgcagagacgaaatgcaagttgtgccacgtcaaaataaacttctttaagtgtagttgaaaaatcatggg

ctgaccatgggctgaccattggacaatggtccttaatggtcttggaaaaattttcagaccatgtccatta

aagaccaaccataaaaacccatgaaagatgatggtccaagtggacatgaccattgggctttggacagccc

aattgacacttcta

>At_TAG3N1 Arabidopsis thaliana

cagtgttttcacgcccgacccgaaccgtccggtccgaccggttaacccgtgacccgaacacttttccggt

ttgggtttagtgctaaaacccaacaagttcaaaaccgaaaaaacccacaaaaactcgcaattaacccgtg

acccggtgaaccggttgaaccggccgggtgtgggttttaaaaagttctcttgattttacaataaaatatg

ggattttagactttattcaattatttttaaaagttttttcagttcttaaattcatttgatgttttagatt

ttggtgaagactttacaataccgccttaaaattgaatcgaatggtgagagagacaaccgagattagttgt

ttgaattcacgatgttttattaatttatttctgtcattgatgatctcctaatatatttgttacggtgtta

gacatttagttggatttcaaatttttaaccactttattattattgtttgattttttctatatatatatat

atatatatttgggtgtaaatgattttatattttgttaaacataatataattttatatttgattaaatatg

atatgattatttttgttttatatttataaatactacaawttttttttttataaaatactctaaaatttct

attaatattaaatttattaaataaattttaactaattaacccgtggtccaacccggttgacccgatgacc

cggtgacccagaaggtagtccggttcatcgtccgggtcgggtttcaaaacattg

>Hv_HAT-1 Hordeum vulgare

tagggacgggcatataacccgaaaaccgatttaccgaaccgaattaaccggaaccggatccaaattaacc

gaaaccaaaaactttggtattattatcggttcactactccagaaaaccgaatttagttcggtcaaatcgg

gtttaccccgcgggtacccgaaaggaccgagtaaataagaaccagccaaccagtctacagcgatagcatc

agtccttatcttctcaagtaggcaagtagccaaagtttgctcaatctatccctcctctaccgccgtgagc

tttgttgtttgattctctgcaatctgcatcgacgcttcccaatcccatcgcctcgcatcccctaatttcc

gacccttcccaattcccatcgttcccgtcgcagtatataaaggatgcggcggccgccccggcagcgattt

actcaccgaggaccaggatctccaaaccctagcaggttcgccggagtgggatccatcacagcgctgctgg

tagctcaagcagattgctgtgcagctatcctactgattcgttgcacctgaacaacaagaacagttcaacg

ccgcccaccaagacgctcctgcaaggctgcaacgtgagtccctgttcccactataatttttattcctaat

ttaattgttttaacttcacccaatttgatggtgtgttctgcttagaaaacagtaaataactgatttaatc

attcacgtattgacgtaaggtcacttgattacattttttgctgaacttttctgtacctgagttctgaatt

ttgtataagtgtgaaagcctgcaatcaaacactacagggatcttatgttattcacatgtcttggttgtct

tttgatccggatagcaagagctagttgtcgttgggtacgacttacctgtttatcatggaaaaaacagggg

agaaaacagattatcggttcagaaaaatagatgagcacacacagtctagtttgaggcgtctattcagatg

ttcagctcttattgttttccatcttcgtaatcgagaagaggtttattagttaagtatcaactacagaact

agcatgccatgaatttgtactcactgtatttataggaccttgctcttgtgaatcattaatctcatgtcag

ttcagtccataaataattttaaagaatgtgttgtaggcctaaaataattgttgagtatgtttgcacctga

ctgataaacttgaccttactgtttactgccaccctcttttatgttttcatgagggaatccttaatagttg

atgcaactttgttatgtcgtagatggcctccgagcctcaggagatcatggggatagaagcttctgcgcct

gtgcctacttcaactctgatagttgctggtgcagtacttacgaacatggagaaggctagtgttgatggtg

agctgcaagcaggggaaacagagaaaagggatggcgggaaggctcaagttgtggaggttgaaaagaaaga

tggtcgaaggcagatggtttcgagatcagagatgtggaaccactttgaaaaggtcatgatggatggtgtc

ttgcagaagggaaaatgcaactactgcaagagtgacatcagtgctcatcccgtaatcaatggtacatcag

ctttacgcaaacattttaatatttgcaagcgtaatccccataggaatattggtgatgataagcaagccgt

gctgcaaattaatcagggtgatagtgtacatgcttggaaatttgatcctgatgctattagggctgctttt

gctgaaatgataatagaagatgaactcccatttgcattcggtgaaaaatctgggtttaaaaagttcatgt

ctaaagcctgcccacgtttctcagttccatccaggagaacatgcactagggatgttgttcgcgcattttt

tgaacagaaggccaaactgaaacttttctttaaaggacaatgtgaaagagtgtgtctcacaactgatggt

tggacatctcaacaacaagacagttacatggctgttactgcccacttcatttgtaatgaatggaagcttc

acaagaaagtcattagctttttcaaggttaatggtcataaaggagatgatattgggaaatacttgcagaa

aactttgatcgattgggggatagagaaggtcatgaccattactgttgataatgctagtaataatgatggt

ggtattggttacatgaaaaaggaactgaataaagctaagacttgcatagctggaggcaaatacctgcaca

tgaggtgcgctgcccacattgtcaacttaatagttaccgatggtctaaaagaacttgatgtttcaatcaa

gcgtgtgcgtgcagctgttagatatgtgaaaaatagcccaaatagattgactaagttcaaagaatgtgct

gaccttgagaagcttgacacaaaggccttcttgattcttgatgtgagcactaggtggaactccacatttt

ttatgctgaaggctgctatttgctatgagaaagtttttgcaagatacgcagatgaagatccttactttgc

agttgatcttcttagtgataaaggtaaggacaagggtgttggagttccagatgagcaggactgggagaat

gtcaagaagatggcagattttcttggacattttgcggagctaacaactcgtgtttctgcttcattgcatg

tgactgctaataagttttttcatgagattggtgaggtgaatctgttagtgagaagttggatggaaagtga

ggatggtttgcaaattgcaatggcagagaggatgaaggataagtttgacaaatactggggcaattggcat

gagctcgataccagcaaaaaagggaaggaaaaggagaacttcaacttgctgattttcattgcaaccacac

ttgatccaaggtaaaatactaaaaaatatcataatataatttgtcttcaatatgtaatgctttatttata

ttatagcaccacaaattgaagttggtactttgttattgcatctcaggtacaagctttcagaatatacaca

gtttgccattttagaaatgtatggggaggacaaaggacctaaggtgtgcactgcagttagtacttgtctc

cgagatttgtttgaagaatatagggtgatgtatggtccaaatattccatcatatgaaactagtgcttctc

atgaagttcaatcaagtggagggcgtgtaagcatgatgaagtccttgattgctaagaaaatgagactgaa

cagtggtggtagtagcagcagcaagtctgagctggacaagttccttgccgaggagcctgaagatgatggc

ccaaaatttgacatattggattggtggaagattaactcatccaggtttcctatattggcatgcttggctc

gggatgttttggcagtccccatctcgactgttgcatctgagtctgcattctctaccagtggacgtattct

cgatgacttcaggacttctctgactccattcatggttgaagcccttgtgtgcacacaagattggctacga

tatggtacttacatcaacatagcagagaacactgaagaattgaccaagcttgaacaaggtaacttgattt

aattaatctatccttatgtaatatgtgttcatttgctgctatataatccttcttccaccattttgttttt

tcagaattgattgcagaatataaggagaaagccaagctccaaagcaatgcgaagcaacccaccactgaaa

agcctaaggattggtgagcttccacttctgagctataggaattcatcttgctatttcagttcatattgaa

atcataatctatgtatgattttatctacccatatgaaccactaaagtgaattatcgttgatattacagat

gttcagattggttgtcctggttgcggttctgaataactactgctggttgttagagcagatgcactaaagc

ttgaagttaagaagttggggcatcgcagcgatactgctcttgtacgctgctcgttgctgtttatttcatg

tttttaaaatttagtcgaaccacccgtttctgtcgtcaaacttaatgttatgtcaggtcttgagactttg

gctttgtgtgagtgtgcactaaactactgttggatgctatgttctgtatcaatatatgcttacgtccaat

ctgtgttaactctacatcatgatattacattttgtgattgattgttgttcctgtttattaagaaaggaca

gaaaataggagatgttttgtcaaaagaaatagtagaccatttgtttttacacaagaaaagtgcagattag

tgtagagattccagttattattaaatgattggggttgcacactagtgggttgtttctactattttttaat

gattggggttccagactagtgggtggttcctcctattattaaatgaaatatgggtctgttcggtcagaac

ccttgaccgaaccgaactcttggtgtacaaaagcatcggttagtgcttctcctgttaaccgatcggtcat

tgtttttgaacaaccgaaattttaaaacaccgaagtaccgacccgaatggttcgggggaaccgaatgccc

aggccga

>Hv_TREP43 Hordeum vulgare gcgcctctactcgcaaaacaaattccgtgttctctgcatacgctcaaggtatatattagaaaaacagtag

caatagcattagcattactaattggttgtagattgggaagcatcaatattgactgtagaataatacgaaa

atctgtttataacagggttgaaaagaaaagctgagcctctctagtcggattcagaatgtacgtgcacgtg

cgcgtgggcatggatgtggccgcacatcaccaccaccaccaacagctgagcgggccacccgcccagagtg

taggcagtagcagcgc

>Mt_HAT1 Medicago truncatula

taaagctgtcaaaatgggccgggccgtaagggccggcccgaaagcccgaataaaatgtagggtttgggcc

gaataattggagcccgaaatttgaatagggctttttagcccggcccgtaaaagcccttggcccgttaggg

ctagcccgtccgggctccgggctgcccgaaagcccgcacaaaatacaacagtttttttttttttttttgt

ggtatgtgctgtgtgtgagtgtgagtcagccagcccgccccaatttggaaatccgacccggcccggccca

agtgaattgaaaagagaacaattgaacaaaccctaattcactcaacactctcctctctctcactcactct

ctcttacgtaccgtacggtgccgccgctcctccatttctctcaagtctcaacactctcctctcactctct

ctttgttacggccgccgctccatttctctctaatctcagctcaacactctcctctcactctcctcgccgc

tccatttcacactctcctctcactctcatttttacggccgccggcgccgctccatttctctctaatctca

gccctcaggtcaggtcggaggtcgcacaagctcaccatcaggtctcacttctcagccctcaggtcgcatc

gcagccatcaggtcggaggtcgcatctcgtctcaatctgaggtcgcatcaggtcagagctcaccatcctt

tcttcgttttattggttttgttagtttctttgtttcttcgttttttgcagtttctgcgatcctcatcatt

ttcttaatttctaattcaggctcagcaggcagcagctattgtttctaattcaggcttaacagctccattt

ctaattcaggcttaacagctccatttctaattcaggttcgtatttcttttgttattgaagtgaactgtgt

gaagtataatatacttggctaaaattgaagtaacagcaacatcgtgtcgtgttacaattacaatgtattt

cttttcttaatcttcatcgatgaagttattacaatgtatgtaaattgaagtataacattaatagctgctg

aaatataatatacttatgtaatcatatttaatgaactgatatagagtagttattctatatgtaatcttta

atgattgaatttctggtttagaagtcgttattctacatttaattaggcttataatttggcatataatgtt

gcaggaattacaatctgtttgtgcttcaaatatgagggataatgatattgatagtagtggaagtcatgaa

aatatgtgcgttgatggggaaattgagagccaagctcgggaaattccattggtaccaccaattgatcaaa

atgcacaagaagtgtcttctgatccaaaggataagaagcgtaagggcaaggctaaggctaaggacaagag

caagggtaaagccctaacatctgatgtgtggctatatcttgtgaaagttggtattgtagatggagtagag

aaatgtagatgcaaggcgtgtcacaaattattaacttgtgaatcaggaagcggaactagtcatttaaagc

gtcatgtacgtagttgtagcaagactataaaaaatcatgatgtgggtgaaatgatgattgatgttgaagg

aaaattgagaaagaaaaagtttgaccctatggctaatcgagaatttttagctagaatcatgattacacat

ggtgcaccatttaatatggttgagtggaaggtgtttagagaataccagaagtttttgaatgatgattgtg

ttttcgttagtagaaatacaatagccaaagagattttgaatgtttatcgtgatgagaaacaaaagctgaa

atcacagttagctcaaattcgagggagagtttgcttgacttctgattgttggacggcatgcagcaatgaa

ggttatatttctttaactgctcattatgttaatgtgaattggaagttagaaagtaagattttagcatttg

ctcacatggaacccccacatagtgggcgagatttagctttgaaggttttagaaatgttagatgattgggg

cattgaaaagaaaattttttccatcactttagataatgcttctgcaaacaatagtatggctaactttttg

aaagagcatctaagtttatcaaatagtttgttgcttgatggagaatttttccatataagatgctcagctc

acatcttgaacctcattgttcaagatggactgaaggtagttagtgatgctttgcataagattagacaaag

tgtggcttatgtgagggtaacagaaggtagaacactacttttttccgaatgtgttagaattgttggtgac

attgatacaaccataggattgagattagattgtgttacccgatggaattccacttatataatgctacaga

gtgcgcttgtttatcgtcgtgcattttatagcttaagtttacgggattcaaattttaagtgttgtcctac

aagtgaggagtggagaagggctgaaataatgtgtgagattttgaagccatttttcactattacaaacttg

atatctggctcttcatatcctacatcaaatctgtactttggtgaaatatggaagattgagtgcctcataa

gatcttatctgacaagtgaagatcttttaattcaaaaaatggctgaaaatatgaaggtgaaatttgataa

gtattggagtgactataatgttgttttggcagttggggctgttcttgatccaaccaaaaagtttaacttt

ttgaaatttgcttatgaaaaacttgacccgctcacaagtgaggagaagttgaaaaaagttaagatgactt

tggggaagcttttttccgagtacatcaagaatggaattccttctaatctaagctcttcacaagtccagcc

tagctatggtggaggaactcgaattacatcatcttcatatgatgtaagttcatacattttttctttattt

tattcttttattaattatttgtaacttggttaggttggatgatttgtaggaatttgaagaatatgaaagc

caatcaagtaacaacaccggaaaatcagaacttgatacttatttagatgagttgcggatgcctctatctc

aagaatttgatgtcttagctttttggaaggaaagaagtcgtagaagtccaaatcttgcaaggatggcttg

cgatatattgagtattccaataacaacggtggcatcagaatctgcgtttagtattggcgcccgagttgtg

aataggtatagaagttcaatgaaagatgattctgttcaggctctcttgtgcgcacgtagctggttacatg

gttttgaaggtattagttctatcatttttacaattgttttatagtttatattattgtcaatagctggtta

caattgttttatggtttttttttaaaacatttgcaacatggtttctgcaaaattgtattcagttcccctt

actggtttgatagaatgctaattatgtttgattcctttgcattttttccatgttctggtgtttgaaaaaa

ctataggttgataagttctagaatagtctcgtgaatttattgcttcttagttccttatcaatttctgctt

tggttttctgtttggtgtgcaagatattgtctctaaattattgaagatgttggtgaaagattagtatgca

atattcttggagtttcatgattgatttttgttcaatatttaacaatcaaatttctcacagaattgattta

tttttagttagtatgctagattaggtagttagtaatagtatgcaatattctcacagaattaatttatttt

tagttatcaatactgaagtgattttttaatctctatatttaacaatcaaatttcgtaatttgtagaatta

tatgatgacaacaatgatgttcaagaagatgaaactcatggaagtggacaagcatcaaatagtaccgtgg

atgttgtgaaccttgaagaagattaagtgactgactttgttgggagtgactttttggattaaaacttttg

ttatgttattgttattacttattctggataattagtaatttgaatttagaatcttcattctggataatta

gcttgatggaagcctattttgtcatcctagttgaaggattgaacaatagatacttctactgttttacatg

atgcctgtctctgttaattacatacagttttggattttagggtacattaaccaacatgagttggcatgga

gaaaatgaaacaccagcccggtaatatcaagcttacctccaggaaaaatggaaaatgaacataaacttgt

gtataagtatatttaagttattataatttatgttcaatcattaaagaatgggaagaaaaagaaaaaaaaa

cagatacgggcccgggctggcccgttagcccggtatatttatgtatttcgtacttaaaagaatggaaaag

ggaaggaaaaaaatacgggccagggctggcccgttagcccgggagcccgtatagggccgggctcgggcct

gtttttagcagcccatattcaaaccgggctttttagcccggcccttaaaagcccttggcccggccgggcc

gggcctaaacgggccgggccgcccgttttgacagctcta

>Mt_HAT2 Medicago truncatula

taggggtgtacatgggttgggttaatccggaaaacccggtcaaacccacccaaagaaaaccaaaaaaatg

ggttgggccgggtaaatgggtgaatgtggttttaaaaattgatatacccataaaataaaatgggttccgg

gtaaaaccggacccaaacccaaaaacccattttaacccggaagatgagcaatcaatacatatattccaat

ttttctcaaacgttaatactgaaatttttttttcttctcaaggagcattgatactatacacattccaatt

tcataagttcagtttccaaatagtgttcaccgatacatatttcattttgccaattttcaattcaatgttc

aattcaatatataaagagaccaacattaaccaaccagtaaacccatctaccaaaatcaaactcattcgtt

ccagcaataacatactatttatgaaagattcatttcacacattgcataacaataatttctcgttccagca

taccatttttcttaatccatagttgttttgcatcatcaaagtaagcttgcttcttgtggctaaattgaca

gtaccatatctaactcatgttcatttctttttttagtttgcacaaaaggtgcttgatgaaatgtctactt

catatcaatattttttaatgtttatttatagtatctacttataagttagaattgaaaaatactttaatag

tttgatttttcgattctcttttattttaatactgttgtacatgattgttaatgcaggagaacatggatgt

tgatgatgttcctacaaatgctgtggaaaatccaattgatgttgattctgatcatgacttacaaggtgaa

gcggcaggtgacgtcggaaaaaatcgtaagcgttctcgtgcttgggatcattttgtaccagacggtaaaa

gggcaaagtgtatttactgtaatatgacgtatgctgctgaaggtaatacacatggaaccacaaatctcaa

taaacactataagaaatgccccaagaatccaaacagggtaattgacaaggcacaaaaaacccttgtactt

gggaagcaagtagagggtgatagtaatgtctcttttaagcttgttgaatttaatcaattagagtgtagaa

tggagcttgctaagatgattatcattgatgaacttccctttaaacatgttgaaggtgttggatttaaagg

ttttatgagtcgtgctcaacctcgtttgaaaattcctagtcgtgtcactgttgctaaagattgtatggaa

ttatataaggaagaaaaagtgatcttaagatctttgttgtctctcaaccagcaaatggtgtcattgacca

ctgatacgtggacctcaattcagaacatgaattacatgtgtgtgacaggtcatttcattgatgaagggtg

ggaattacaaaaaaagattttaggctttgggttaattgctaaccataggggtgacaccataggtaaagcc

ttagaaaaatgcttgaaagattggggaattactaaactgtgcacagttacagtagataatgctagctcta

ataatgttgctctatcttatttgactagaaatatgagtgcttggaatggaaataccttgcttaaagggga

gtatatgcatctgcggtgttgtgcccatatcttgaatttgattgtttttgatggattatctcttatagat

tcatccatttctaagatcagggctgcttgcaagcatgttaagtcatctccttctagattggcattgttta

aagtgtgtgtgaaagatgcaaacatttctagctcacaaaaggtagtaattgatgtagcaacaagatggaa

taccacatatttaatgttggaggtagcttctaaatatgaagaggcattcaaccgcttggagggtgaagat

ccttcatatgtgtctgaacttgaggtcgtaggaggaactccaactgtgtatgattggaaccgtgcccgtg

tgtttatcaattttctgaagatattttatgatgctaccctaactttttcctcttctttacatataagtgc

taactgtttctttagaaagttggtgaagattcatactgcactgtcatcatggattcaaggtgaagatgtt

gtgttgaaaaacatggcattgactatgaaagctaaatttgaaaagtattggagtgatgagaatatcaatt

atctgttgtttgtagctgtttatcttgatcctaggtacaagatggagtatcttgatttttgttttggttg

gatgtatggggtggaaaaggctaaaatgataattgctaaactgcatgaactcataggcaaattgtttgat

tattacaagtcagtgcagcctattggttttgtttctagtgattcctcctctagctccactaatacaatgc

aaagtgatgttgttactttcggtgttggaggtaatgtggatatggatgctttgcaccgaagaagggttaa

aagaaggcaaagtgaacataactcaagtgagttggtaagatacttagaagatgaggttgaagatgattat

gaaggatttgaacatttgaaatggtggaaaagtaagagcacaaagtacattgttctttctctcatagcta

gggatatattatcgattccgatatctactgtttcttctgagtctgcctttagcactggaggtcgtgttat

tgatccataccgtagttctttgaaagctgaaacagttgaggctttgatctgtactcaaaattggatcaag

cctgtcacaaggtctgttttagatgaaagtaaagcatttgatgtgctggaatttgaaacaggtattaatt

gtttcttagcttttatctttttattttatcttttagttttattcttacattgtttatttttttatgtcat

tagaaatgtcatatttgaatggtctcgaggagtttgcagctgaatcccttgacgagtaactaggtaaaaa

tctaataccattgtttcatgtttatactaattttgtcttgtgtgattggtgttcctgttgttgatggggt

agtctatttcctgttgttgatgaagtttacgttttttagttgcatactactactatgtgtttttcttatg

tgccttcaattagagcctgacttacatattaatgaaggttgtttgtcactgtcatgatgtcctggcaggt

ttagcaacttgggttatattgagtgatcttttgaattgggattacacatgtttgtcttaatttttcatta

acatttcagatttttcttgctcattattatgtaatgtagtgctgagattttcacaatggataaatgtata

tgtttcttgctgcatacttttgaactccaatctatgacataattatcacttatgtagtatttatgacatg

catacttttgaactccaaattggtagtctatgaactaatgctttaatttttattatcacagtttggtgga

gatggagaatataaacgatgaagtcaggactcttttgaattgtggggaatgttaatgatgacatcaagac

tcgtttcttgttatgttttgggctcttttttgtatttgatgagacatgattttggtttattttgacttgg

ttgttgcaatttgtctcttgaacatttgttgttttcttttgccaagactttagctaatttaagatgatgc

actctttgagtatttttattctaatgcaattttatgttatgcaatgtctttaaatttttgagttattttt

atgaaaaaattatgatgacttgtcatttggtttaatactacaaaaaaaaagaagcaaaaattaattcggg

taacccactacccaacccaatccaacccggaaattagtgggtttacccaaaccggcccaatagtttaacg

gatggacattttatctcacccaaaccggagtaccttatatggtttgggttttggatttggccaaacccaa

cctaaaccggcccacttacaccccta

>Mt_HAT3 Medicago truncatula

tagggttgggaataggctaggccggcctacaggggcctacgacctggcctatttaagcctggcctggcct

ggcctatttattaaaaaggccaggttaaggcttttttaaaagcctatttgatcaaataggccaggcttag

gcttttaaaaaagcctgttaagcctgataggccggcctatttacacccataagttaagcctaataggcct

aattattattatattaatatatatcaataaaaaataattcccatcccaattcccattatcagctaaaaat

atgaaacggcaaattgtatttcccaatttccattcaatatcattcccaattaatggcttctcttgaattg

cttccttcttttgaatcgccgccgctgctagtaggtttgaatcgctgctgcctgctgccttctcttgaat

cgcagcctgccaccttatcaccgccgccgccgtatcaccgtcgccgtatcaggttttcttctttcttctt

tcttttcttttttttgaatcgctgcctgctgccttcttttcatttgaatcgctgataccttttcttcttt

cttttcttttttttttttttttttatctcttcatagtgttcttcttatattcgttatttggattgaaact

tcattttttcagctaaagattgaatctttgagatttttctgttattgggtttttgtttaatgttctctga

cttgattttagaaacccatttttttcagctaaagattttgggtttcaactcataatctctgttacttgga

agtttaatgttctctattattgggtttctgtttaatgttctcttcttcatcgtgttctacctttcactgt

ttaaggttctagaatggacaactacaggttgcaacctagaaatggagaaaaggaatataggaaaaccaaa

ttttttagttttatggatcagaggcatttaataaaattgattaatagttttctacttaatataacacatt

ttcattaatgtttttaccatttacagcaaatgtcttctcaagcaaatgaagaaccaactcaagcaggagc

aaacacaactcaagcagaagcaaatgcaacccaagaagaagaaactcaagctgaaccaattgttgggaga

aagaggaaaaaaacaagtgtaatttggaaagatttcgatgaaaaggaaattactaaaggtgtgtttagag

ctgtttgtaaacactgcaaagcacaatatactactgggacggtaggatccagcacgagtcaaatgaaacg

acatcttgtaagttgcactgccaagaaattgcaagatgctactgaaaagagacaagctgctattccattt

aagcgtgtgagttcaggtaacccctttcttacttctggtgttggatactctaatgaaaggatgagggaaa

taattgcaactgctgtaatggttcatgaatatccttttaatgttgttgaggatgatgtttggatgtgggc

attcgaatatgcaaatcctgagtttcgcaaggttactcataaaacaacaagaagtgattgtttgaaacta

ttcgagaatgagaaaaaaatcttaaagaaacagttggaaagtgtgagcaagattagtttaacaacagata

tgtggaaatctagccatcaagtggttgaatatatggttatcacaggacatttcattgatgcgggatggaa

tcttcagaaaagagttttgagttttgtgaaagtgcctgcaccaagacgtggtattgacgtggctgatgct

attcataaatgtttgaaaacttggaggattgaaagtaaaatatttacagtatctgttgataatgctgctt

acaatgatttgtgcttgaaatatcttaaggataatatatcgatgagtagaaagttaatccttaatggtga

tttgtttcatgttaggtgctgtgcgcatattttgaatttgttagtgcaggacggccttagtaaaattaag

gatatcatttttaatattcgtgagagtgtcaaatatgttaaccacaatgatgcaaggctaaagaacttct

gtgatgtggttgagcaaaaaggtttgaaagaaaggaaactcgtcatcgattgtcctacaagatggaattc

aaccttcaatatggcgtcaaccgctttgaaatttaaaattgtattttcagcctacaaagaaagagagcct

cactatgatcatgccccttcatttgaagaatgggacaaagttgagaaagtgtgtaaattgctagaagtgt

tcaattctgctactcatgtgatctcaggtagtttctcaactatttaattgttgtactttattactcttag

aaatgtttaatataactagtgtttaatataaatatttttgtactttattactcttaaaaatgtttaatat

aactagtgtttaatattactattttaaaaatgtttaatataactatcgtttaatataactattttgaatg

taggtagtgagtatccaactgcaaatttgtatttgccagaggtctggagggtgaagcaagtacttgatat

ggcggatgaagatgaagatctctttatgagagaaatggcaaaaccaatgaaaaagaagtttgacaaatac

tggggggagagtaatttgttgatggctatagctagtgttttggatcctaggtgcaaatttcacagtgttt

gtatatgttttcccaagatatataaatccaaggaagtttctgatgagaatatagagaaggttaggcgttc

cttggaattattatatgatgagtatgtggccttatctttggaagagtcttatttgatgcctgctgttaat

ttggataattcatcttcctctcaaacaaatgttaaaaatgccactggaattgatgacttgttacaaacca

ttcgggagcaacaagccatttctcccacgaagtcagaattgcaagattatcttgatcaaggtgttcatgt

tgttcctaactctgaatcctttagtgctttggaatggtggaggaacaacagcatgaagtataagatcttg

tctaagatggctgctgatatactagctattccaatctcaactgttgcatcagagtccacattcagtgctg

gaggtagagttatcgatgaatttcgctctagattaaatgaagaatctgttgaagctctcatttgtggtgg

tgattggttccgtcataaatatggtgtgaagaacaaatcaaaggttattctttgtcatgatattttacta

atatttattcatatattcatatggtattgtttagtccaataactaaagtatttttttcttttggttccca

aggttgataaagatgagatacaaatcaacttgaagatttgatatcattttggttttttgggctggtttgt

gtacatgaagctgatgagtgatttgttgacttgtgtgatatgttgaattatgctgacttttgctacacct

tgaagatttgatatcattttggttttttgggtggtctctgtacatggagctgactatgagtgatatgttg

acatgctgacttttgctacacttgagttttgactaagaccatgtgactatgagtctatgactaatgagtg

tcactctatcactcatttctttttttgttttctcatatcaagcactttatgaaatcatatgattttctat

tttttggtgaattgtactcaaatggttgataaacattgatgagtactaacgaaagattattattattagc

tctaaattcattgtgatttgttgttttgttgttataggcttttaaagagcttgttatgttgttaaatttt

gtttttagtaaaataggcttaaaggcctgtttagcctatttagtacgtaaaatgaactatttgatgacca

taatgttaaataggcttcaaattaggctttcaggccaggccaggctttttaataggccaggccaggccaa

gaaaaacggcctatgataggccataggccaggcttaggcttgtatattttttcgtaggccaggctcaggc

ctttcaaagcctggcctggcctggcctattcccaacccta

>Mt_METRAHAT Medicago truncatula

ggggtggacaacaatagtcgacccgacccgaacccggcaaacccgttggaaaattgaaagtctgggtggg

tagcgggtcggtttccgggttgggcggattgaaggaatggtttcagatgggtttagatgggcgggctcgg

attgaattttcaggcccatcgaaacccaaatccgaccgagcccaactaatattcaatcagtattttttta

cccctaaggccctattcttgttaagttgctggacttttgcttggatcaaatttaagcccaattctttaga

tccattgacaacaaactagggttatatcaaaacacacactcatacttatccattcattgagagaaagaga

gagaacgagttctggaagttgaaggaaagccagtttcactgcgccaccgtcgtaaacatattgaccacca

cttcctccctctctctcgatcgatttcattttgaaacccaaagaagatgtctgtccattttcgttttcgt

tttcgtaagtgttcattttcgtaagttcattttcgttttcgttttcttcacttaatctcttgatttttct

gttatccatctaattaagttcatcttttgcggatgtgtttgacagatgtttggtactagtagatcgattt

tcacaaaggtacgtgttgtatcgtattcactattttgttggctagggtttgcattttttcatcatagaag

aaatcaaagtttacttatccatctaaccaagttttcttttttactttgtatgttcattttctcttatctg

aagaacaaatcaaagctcaacttaaagaaatagaaggtatatgactatatgcttgtgttcattatttttc

ttttttggtttgtttttacttttttggtttgttcttactttatttctaataaattcaaactgggttttaa

tttgtaatttttgttagaaatgtaaaaaaaactttttctaaactaaatcaaacaagtttttgtaattctt

ttttgttttggtagtttctttactgttttctttcaaagtaaacgcaaggactgtcatgataatcttgaat

caaagagttgctgaagcatgctttattttcaatgttcaattcaagctcaacaaatatattcgtgcaaaaa

tgatttaacaataaatttgagagtaaaaaacaattctagaaacaagctttactaacaattatccttttta

tttttgaaggttattggctttttggtggtatttaagtttgtagtgattattgattaagctgttgaaaagg

taagttttttaatccatcctttttgaacatgtaaatataaagctatgaaatatttaagttgatgtggttg

acgttttttttacttaatcttcatgttttcagtaatggagagtataagtcaaaatttgtcttctacctta

acatctggaagcgctaatgttgctgagcctactgaacaagtagcagttgctactcaagttcctgttgtcg

ggcttcctcctctcccttgtcttgcaaaaaggaggaagcctaatgctggtggtcctaggagaacctctcc

agcctgggaccacttcattaagttacctgatgaaccagaacctactgctgcatgcatacactgtcataaa

agatatttatgtgatccaaaaactcatggaacttctaacttgctggcccactcaaaagtatgctttaaaa

atccacaaaatgatcctacacaagcttctcttatgttttctaatggggagggtggtactttagttgctgc

tagccaaagatttaatcctgcagcttgtaggaaggctatagctttatttgtacttctagatgaacatgct

tttagagtagttgaaggggaaggttttaagctcttatgtagacagttgcaacctctcttaaccattccat

ctaggaggactgtggctagggactgtttccagctctttcttgatgaaaatctgagattaaaaacatattt

caaatcagactgtgttagggtagctttaaccactgattgttggacatctggtcaaaactttagctatatg

accctcactgcccatttcattaacaatgactggaagtatgaaaaaagaatcttgagtttttgcacagtcc

ccaatcataagggtgacacaattggtaggaaagtggaagagattttgaaagagtgggggataaggaatgt

gtctacaataacagtggacaatgcatcttctaatgatgtagctgttgcttacttgaagaaaagaattaat

aacatgggaggtttaatgggtgatggttctttctttcatcttcgttgttgcgctcatatcctaaaccttg

tggtgggggatggtttaaaacaaaatgagctctctatttcttcaattagaaatgctgttaggtttgtgag

atcgtcaccccaaagatctgcaaaatttaaagagtgcattgagtttgctagaattacttgtaagaagatg

ttgtgtcttgatgtccaaacaaggtggaacacagcgtatttaatgctagatggtgctgagaaattccaac

cagcctttgagaagttggagggtgaagattctgggtatttggagttttttggggaagctggtcctcctag

tattcatgactgggagaatgttaggtgttttgtcaggtttctaaaaattttctatgatgctaccaaggag

ttttcctcatctcaggaagtgtctttgcacaaggcattccaccaactagcttctgttcattgtgagttga

aaagatcagccatgaacctgaacacagttttagcttcaatggggtctgacatgaagcagaaatacaacaa

gtattggggtaaaattgaaaacatcaacaagcttatttactttggtgtgattcttgaccctcgatacaag

tttagctatgtggagtggtgcttcaatgacatgtatggtgaccaacctacattttttactgatctgattg

ctgtgatccatactcagctgttcaaactgttcaattggtacaaggatgcctatgaccagcaacataattc

tggtcatccatctgcaagtccatccgagtctagttatgttagcgagaatgtgatcccagctgaagtccca

tctcatttggcaagggctgaagcttttaaggagcatcttaaactgaaggaatcaatagttaaaaaaaatg

agcttgaaaggtacctagatgaggagcgtgcggaggatgtcaactttgagatccttctttggtggaaaca

aaactcttgtcgttatcctgttttgtcatccatggtgagggatgttttagctacaccagtttcaactgtg

gcttctgaaagtgcctttagcactggaggaagagttttagatacatatagaagttcattgaacccacaaa

tggcagaggcattgatctgtgcacaaaattggttgaaacccactttgaatcaattcaaagacctcaacat

aaatgaggaatttgagctgtctgccactgttgtatcaggtatttaatatgtttattttaacttttaaatg

tgtggcatttattcttgtttaaatgccattaactaatcatattgcttggtccttgtatttgtatatagaa

tttgatggaccatcagctaatggatcaacatcttgtggtgttagagcagctgttgtacaaggagatgcat

catcatctcaatcacagccaatgagctgtgattgaaaaaactagtgttagtgattatattatttttcatt

atttgtgtttatgttattatttattttattaagttatgccattgtttattttattaatgtagttattgtt

tttatttacaggttcttaacccttttttggtgtcattaatcataatgattaatgactttgcttagtgcat

tttttgggagaatctactttttgtggaatatacacaacaactctttttggaagcatctggaggatgatga

aggattttaatccaacttgtatgtttgaagacattttgaaatgtagatgcttgcttgcttggctccatgg

tctcacatttttgcttgattgatagcatatacactgctgtttaaacgatgtttaatgcttatgctaaggt

atgttaggcttagtaagtgttacttgataatgcttgtagttatcatggttttaaatcccttactgtttgt

tttattcttctttttttcaagtcagtgatgcagttataaatctagaggtcagctctagtgatggtaatgt

aggagagttcactgtttttatcccaaactgttgcacaaaggtacaataatgtaggatgcataatgatttt

attgtgatgattttactgtgatgaaatgtgttggtaacacattgcttttgattttcattattcaagcaga

ttattgatgttaacttgggtgtggctactcttgtacttgctgaaatgatgagatgtgatattaatttgaa

cacaatcaaggttagttttagtttatttattgaatttagtcttgattctaacttcatctataaaaaaata

caaaatttatatcttataacattttgtccctcttgttgtgcagctatatttgtgtgaccatgttgcagca

tcagcaactgcaagcatctcctgccaatgtatttgtagaattatggatgattgtaattatggcattgtaa

agtttaagttgttggttgtgttgtgcagtcttatcaactctaaacgtattgcacagtcgtacttttattt

atttgaagtttgtaatattatgatacaggaataccggatacctatcacatggatgatgaattatcatgaa

tgatgtagttgggcttctttaaaccaactaaattcatgtttatttcctattacagggctgaatcatgaat

gatcaaatgatgtaattaggcttttctaagccaacttaactcttgtatgatatgacaagaaaatatgctt

aatttatttgtatttcattggcaacttagattgagaggggatggaatcatgaactgatccaatttggaaa

ttatggttaaaaatatttgaaaaacagcgatgggccaaaaatccatcaccggcccaatgttggcctcatc

cgacataacccacccataacatccgctgaaaccgattaaccgagacccgccataaccgttgcttcatttg

ggtgaaattgggccttgaattctcaaccgtggattgtttcggattgagctttctgagcccgaacccaccc

atacccgaccgttgtccacccc

>Mt_Murb Medicago truncatula

gggttgggaataggccaggccggcctacaggggcctatggcctggcctgtttaagcctggcctggcctgg

cctgtttattaaaaaggctaggcttaggctttttaaaaagcctatttaagtaaataggccaggcttaggc

tatcaaaaaagcctatgaagcctaataggccggcctgtttatgcatgttaggcttcatagtggacttttt

aaataggctttaaagctttatagtgaaataggcttttaaggccttatagtagtgatagacaagtcttaca

ctgaaataggctttgaggcctattaagcctatttaaaagtagataaaatggaatgtttagtgactttaat

agtaagtaggcctgtaaataggctttcaggccaggccagacttttaaataggccaggccaggccaaaaaa

ataggcctatgaaaggccataggccaggctcaggcctgcaaattttttcgtaggccaggctcaggcctat

caaagcctggcctggcctggcctattcccaaccc

>Mt_RAHAT Medicago truncatula

cagtggcggatccagggggtggcaagggggagccacagccaccccattttttttgtgaaattacgaagat

gcccttgcttattaataatattttccaactgatatttcatttggtcagcatatttttctctcttcagaac

aaaaaaacgtgacttctcttgttcagaacaaacacacgcgacttctcttcttcttctccaaattccatcg

ctgatcactccatccaattccaaactcttcttcttccgcggccgccactgctccagtcgcaaccgccgct

gcaaccgcctccttctcgccactgcgtcactgcgaccaccttcttctccaaattccaatcgctgatcact

ccatccaaactcttcttcttccgcggccgccactgctcgaatcgcaaccgccgctgcaaccacctccttc

tccaatctccatgactccatccatcttctctgttgtctaaagtctccacaaaccctaattcttgtaaatt

gcaaggtaataagaaattgaatctattttccttcaaatatgattattctctgatgattacatgttcttga

atgctctttaactgcttgttggttttgaaaaagtatagctttttgttgattttatttaagttatctgtta

gttgtgataacttgttctatattttcgttggttttaatcacttttagttacctctgattgttcaatttat

tggttttttcaagtgttggttcactttgagttcaattatcaattttagagactctgttagttgtgattct

gttaaaatagactttggaatattatgttcccttaatccctttgatctgtttcaaactattgttgattgta

atcttattgttggtttgattcttgctgttaacaggtggaaaagttaagatgaagtttcaaagaatcgatt

ctattttcaagaggaaggccgttgatattcaaaaagatgaagttataatttcttcatccgaacctgaaca

agttcatgagaatccaagaatcgaagaaaatgagtcccgtccttcaaagattaatagagttgatccggat

gacattgaaaattctttagaaagggatccaggaaaatgtattccaatttaccaatatccaccaaatcaaa

aggatgcaatacgaagagcctatctaaaatggggtccttatcaatcaaacttagaaaactatcccatgtc

cggtatcgggaaagcacaaaggaggtttcaaaacagttggtttagcttgttttcttcgtggctagaatat

tcgccgtcggaagatgctgcctattgcttacaatgttatctatttagcaacaaaccaagtggacgtctcg

gatcagaagtattcatttctactggctttagaagttggaggaaagttaggaatggagagaattgttcctt

tcttaaacatatagggaaggatcctcgctcaccacacaacaatgcaatgaaagcttgccaagacttcttg

aatcaagatgggcatcttaggaatgttattgaagtgcaaagttcgagtcaaattctgaataatcgactat

gtctcaagacttcaattgacactgttcgttggttaacaattcaagcttgtgcttttaggggtcaccgtga

aggaaacaaatcgagaaatcaaggtaattttcttgaattgttaaaacttttagcatcctacaatgatgaa

gttgcaaaagttgtgttgaaaaatgctccagaaaaatgcaattatacttcacatcaaatccaaaaagaga

tattgcaaattctttctagtagggtgagaaaacatattcgtgaagaaattggtgattctaaattttgtat

cgtcgttgatgaagctcgtgatgagtcaaaaaagaaacaaatggctcttgtgttaaggtttgttgataaa

gctggtttgatacaagagagattttttgatgtggcacgtgttaatgacactgcttccttaactcttaagg

aagcagtatgtggtatactttctcgacataaccttgatgtttctaacattcgtggtcaagggtatgacgg

tgctagcaatatgagaggagaatggaatggtttacaagcactttttatgaaagattgtccttatgcttac

tatgtccactgttttgctcatcggttgcaacttgctttagttactgcatcaagagaagttgcatcaattc

ataaattctttgagaagctgacttttgttgtcaatgttgttggttcttctactaagcgccatgatgagtt

acaagctgcccaagcaaaagaaatcgaaaatttgttagagactggggagattgtaactggtaaaggtaaa

aaccaagttggaactgtgaaaagagatggagatactcgttggggatcacatttcaactctatttgtagct

tgataagtatgtatgaagcaacttgtacagttttgaaaatcattgcaaaagatgcaaaaaaatttgccca

acgtgcggatgctgatagttcttacaatcacctaaagtcttttgattttatatttatcttgcatttgatg

aaagaaattatggggacaacagatttgctttgtcaagccttgcaaaaacaatctcaggatgttgttaacg

ctgtaattttggttcgttcaacaaaagctcttattcaagatttgagagaaaatggttgggataagttgtt

tgccaatgtcgtgtctttttgtgaaaaacatgatattgaggttcctgacctcaatgattgtcattcaaca

acaagatttgggcgttctcgccttgaagagaatcaggtaacaatagaacattatttcagagttgaatttc

tttttactaccattgacaaacaattgcaagagttgaatagcagatttagtgagcaagcaatggatttgtt

gactttaagttgtgctttgtctccggaggatggatataaagcttttgacattaacactatatgtactctt

gttgaaaaatattatcccatggattttagtgaccaggagaagattaatttgccatttcatcttaaacatt

tcctttttgaggctcgtgaatcatcaactttgaaaaatttatcaactattcaagaattatgctcatgttt

ggctgctgccgttcctgccaatggacaacccaaaaaacacttgttgcttgataggttgttgcgtcttgtt

atgactcttccggtttctacagccacaactgaaagatctttttcagcaatgaaaattatcaaatctaagt

tgagaaacaagatggaacatgggtttttagcaaatagcatgtcagtttacatcgaaagggatattagtga

gtgtattagttctgaatcaattattgatgatttcaagtcactccgaaagcgtaaagtgcgtctttaggta

tgtaatgatcgactttatatattatgtagtttaaattttgaatgattggtttttggtttattttaatgat

ttatatattatattttagtttattttgatggcgggatgacggccaccccaaacatttttgtctggctccg

ccactg

>Mt_SHATAG Medicago truncatula

ccatagttttcagactcggctcggaccggccggtcggaccggtcggaccgtgaaccggtgggtaggccgg

ttcgagccatcatttggatcggccatgcagataacccggtcaaaaccggtgactcggccgagtcgtgggt

cagaccggttcaatttttttttttttttttttgcttaccaaaacgacgtcgttttggatttgttaattga

aaaaaaaaaaaaaaaccggaattagaagggtcagagaaactaaaacgaaacctcatactctgttcttagt

tttcacgtacagagagaagagaagctaaaacgaaaccggtccactccacgccattctccgtcgccgccgc

gaaaactccaggtcgccgtcgccctcgccatcgccgcttccgcttccgcttatctcacgtacgtacaccg

tcgttgcagagaaccctttttttttttagattgttattctttgcttctttttgatttgttgttttgattt

cttgtttaatgtttaatttcatgtagattctgattgtttaatgtttaatttcatgtttaattatgaatct

ctgattgtttatatgttgattcctcattccatgtttaatgttgactcctctgttttatacttattccatg

ttttatactgactcctctcctctcctctgttttatactcctcattccacgtacgttgttgattctgataa

actgattgttaaatggttcatttcatgttaatgtttaattctgattgtttcataattccatgtaaatgta

atgtaaatgtctctgatatatcatatgtatttgtgattagttatggcctcttctgaagcaccaacacaac

catcagcagaagcttcgactcaagaatctcaaactcaaaggaatgttagggcaaaaaccgatatagcttg

gggtcacgctaaaattgtcctagacggtgacaaagaaaaaccacaatgtatctattgtaacaaagttatg

aagggaggtggaattaatagattaaagttacacttggctggagaaactggacaagtcgaagcatgcagcc

aagctcctgaagaagtccgctttaagatgaaacaaaatcgtgaagagcaaacacaaagagaacaacctag

gtcagtggttgttgcatctcaaaagggaacgaacaatggaagctttgataattactttttgcctagaaca

actcctggatcacagcctactataaaaagtgttttgcaaaccaaggaagttgtagaaaagtgtgatcttg

cacttgcaaaatggttcattgctgcatctattcccttcaatgcagcaaattcaccatattttcagtctgc

ggtcgatgctctttgttgcatgggagccggatataaagctccttctatacatgatttgcgtggtcctttg

ctaaataagtgggttgatgaaacaaagaaaaagatagagaaataccgtgagatttggaagaatactggtt

gtactcttatggcagatgggtggactgacggggttaggagaactctgataaactttttagtttattgccc

taaaggaactgtttttatcaaatctgttgatgcttcaggtgcttcaaaaactggtgagatgttgtttaag

cttttcaaggaagtagtgttatatattggctctgaaaatgttgttcagatagtgacagataatgctgcaa

actatgttgctgctggtaggttattgatgaaaaagagttccctggcctgtattggactccttgtgcagct

cattgcattaacttgatgtttcaagacattggaaaattacctgaagttaaagaggcagtttcacatgcca

caaatgttaccaagtatatatataatcattgctatccattgtatttgatgaggaaatttactcatggaag

agagatacttcgtcctgctccaactcgctttgccactaatttcattgctttgcagagtattttgtctcag

aaaaatgcacttagagccatggtaacatctcaagaatggacaacttctgcttatgcaaaagaagccaagg

ccaaacaatttgtggaacaagtcttgaacactaacttttggactgcttgtgctgacatagtgaaactcac

agaaccacttgtatgtgtgttgcgtctcgtggacagtgaagataaacctgctatgggttttctttacaga

aatatgtataaggctagagaggagatggtgaagaggtttcaaagaaataagacaaaagtggagccttact

tgaagatcatagatgatcgatgggattcacaacttcgaaaaaatcttcatgctgctggttattggttaaa

tccatcttgtagattcagtcctgagtttgagaaacacaagtccaccacatctggtcttatagatgtcatt

gaaaagtatgctcgtaataatcatgagttgcgagcaaagttaaatactgagacaagtatatttagaaatt

ccgagggcgactttggaaggaaatctgctgtagaagctcgaaattcaccatttccaggtatcttacattt

cattgtatcatatacacttacgatgatccatttacaaatatcttacctttcattgtatcatataaactta

cggtaatactgtattaattgatgaaattttgcaattttatagatgaatggtgggaactttacgggtgtca

agcaccacatttgcaaaaattggcaattcgggttctaagtcaaacttgtagctcttctggttgcgagaga

aactggagtgtgtttgagcatattcactcaaaaaaaagaaataggttggagcatcaaaggcttaacgatc

tagtctttgttcgttacaacttaatgctagaaaataggtatgtatttctactatattttagttatattaa

tccattgataaaatagtgtttgtttctttgcataatgggattgttcacaataaaccttatatgttcatca

ttttttatttttacatttagatagaaatctttatatttttataatgaattataggaacaacaaaattcga

aactatgaccccatcaatgatgaattacttgatgatcatcatgataattgggtgttggaggattcaccgc

catttttaacagttgaggagttggaatcattacgcaatgatcttgccaatatgaccatccaacctatttc

aaatgatattggtatgtatgtgttttcttataaggttaaaatttcataatttcactatatggttatatac

caattgttaatatttatttaaattgatgttttcatagatggattgaatttggatgaggatgatgattatg

gcaatgatgcacctgacactaatgcagaaaacatggatcaaagtaatgtttttgatgaagctgctggaga

agatgttgaattccttgatgagcttcaaattcaatcaatattgactccttggaattaagatgttattgat

gataatacttgtgttggaattatattttcttgggatctttttgaatttttataacaggggcaatctcttt

ttttaatttatgttggaatcatacttttttgggatctatttggatttttactaccaagatgtattttgag

ttatgactatgttttgaatttgagcaattagctatgaaattattattgttggatagtttttttcttattt

tataggggtcattattttagtttctaaagtgaccgagtcaccgattaaatccgagttaatccgagttaat

ccggttatataactatataaatttaatctaaggaccgagttatctaaccgagtcatccgagtggttccgg

ttcagtcatgcggttcgaccaatgactcactggttcgaccattgacccattgacccagtacccccgccga

gtcgatgaccgagccgattctgaaaactatgg

>Nt_SLIDE Nicotiana tabacum

taatgctgctcggtgggccgggcctgaaccggaccggaccgggcccgcggtcctaacgggcctggtgggc

ctggtgggccggtcctgggtgggccggtcttggtgggcctctgagcccgtaacgggctggtctccatggt

tgagcccacgagcccgggaccgtttggcccgggaccggcaggcgggcctgggccggtcctggcgggcctg

gcgggcccaacggctatttaaaaaaaaaaaaaaaaaaaaaaaaaaaaaaaatcaaacggccatatttaaa

atctagccgtttgggctgaaaatatgaccgttttttaagttaaaaaaatggccatttggcccccaaactt

tattttaaccccaaactttatataattacacttttccccatttctcaactataaataccccctcattctt

tcatttttattcaccaattcatcaatatctctcaatatctctcaatctctctcaatctctctactacaat

tacttaatttattgttgaaatttcgtgaaaaattgtgaagttgttgaattgaagttttcaagtgttcaac

gattttcaattttcaagaagttgttcggcaatccggtaaactcgtttcaactcttacgtttttataatat

atttttgtgtggtttagtttgcataattataattaatatggcatttactttgaaaaaaatgtttggtaaa

ggaaaagataaaaccggtgaaagtagtggccaaccaactacccttcccccggctccccgacctagaaaag

ataagcaagttgaaagtagtcgccaacctagacgtcctcctccttccgtaattcttgatagtgatcaccc

ttgttttcaatttaccgatagtgaaatttatcataatgttgcaccaggtgatagattagatgatgaaatt

atgaatgctctttatcctaatgaaaccatcttagaaaataatgaggaaaatgaggatgatgatgaaactc

aaacaccggatttagatgatacacctactagtcctcttaataacccaagtgatgcaccggtcgacccacc

tgtagaaactcctacttttaatagagaacctgctaaacgcttagaaacatcattagtttggaattttttt

actcaagtaaggagaaaaaataaggctaagtgtaaaacttgtgggaaattaatgtcgcataaatatgtag

gagaccgtagcggcacaggtagtttgactaggcacataaaaacacaccctagagataaggctagattttt

tcaaatgaaagcgccatctagaggggacaagtgtagattctgcgattaaccctagtacaggttcaaatct

agttcaaccaggaattaacactgtcactggaggtattttatattacgatccaaatagagatcgtgaagaa

ttagcaaagatgattactgttatgtgcttaccttatacttttgcttctaatcctaattgggttcattata

ttagaagagtgtttaatcctacttataaaggttggcctcgcgcaacagttaagagtgatatttataaatt

caaacatgaatatgaacaatatttgcgttatttatttactcatatacctaatcggatttctattactact

gatattggtagaagtggtaatgattgtgattacctaactgttacaagtcattggatagatgaagaatgga

taatgcaaaatcgcataattgcatatagaataattaattcgcgtcacacaggtaaatttatagctaacac

tgttgcagatatttgtaacaaatgcatttcctgggcaatattatcctactatttcaaactgtttagttta

tattgcagcactatctgatttgtttgttgaatttagtgagggtggggatatttatgaacttgctataaat

gaaatgaaacaaaagtttaaaaaatatttttttcctatccctcctatttatggtcttgctgcaatgctaa

atcctacaatgaaattgggaggtcctcatttttggtattcaaatatttataaggctttagatctttcaaa

tgaggaaattgcgacacttgcagatgcaaaagcttcaattaagattaacgctcaaacagtttataatgct

tatcaacttgccttagagcatgctaggccaactattccaacccctacttcgtctagctcacaatcctcta

aaagagttgcgggcttaaaagctcttaaatcttggacggagttcagggggtctcaaggtgaaaattatga

tgaaacttcacatctaaatgagcttcaagtttatttgtctcagggacttgaaaaggagaatccagacggc

tcttttgatcttttggaatggtggaaggcaagggaaaaacattttcctgttcttgcaaggatggctcggg

atattttatcaattcaagcttcaactgttgcatcagagagcgctttcagtcaagcaagactgcaaatagg

tgatcatagagcgtctatgagggatagcttggaaaaatcagtattgtttagagattggatccgctcggaa

agaagaaactttggaattgcagaagcacaaccggcgatagatgaagcttatgaagaaatgatagcggaac

ttacggaggattcgcttcgcccggaagtggtgatgaacaagcttcttttccaccaccaccaacgcaacct

cctccgaaccttgaaggatttatgagatttgttagagataatacatagaataatatgtaacttgtatttt

ggcacatcttccttagtttttttccttctaatggtggtattagtaccttgttgtgctcattccattgggg

gaaggatgactaagaaagatatgtcattttttggtaataaaatttattgcttctacccatgagcttcttt

tcgcaatatttctttgtctatacttagaattatttatatgctacaatatatacataatatacaatatata

tactacaaggaaaatatatttataagctacaatatatacataatatacaatatatatactacaagaaaat

atataagagaatatatatacataagatacaatataatatactacaaggaaatatattatgctacaatata

tacataagatacaatatatatactacaaggaaatatattatgcgaatatatatacataagatacaatata

tatactacaagaaatatataagagaatatatatcataagatacaatataatatactacaaggaaatatat

tatgctacaatatatacataatatacaatatatatactacaaggaaatatatttataagctacaatatat

acataagatacaatatatatactaaaaggaaatatataagagaatatatatacataagatacaatataat

atcaagaagtgatatttatgcatgacaatttagtgttttactattgttttgttattttctttttcgtcaa

gcactttaataattagatatacatatatactacatattaatatagccatgatactacaagaaattgtctt

taaaaaaaaaaaaaaaaaaacccgctaggcccgcgaagcccacgagcccggcccgttaagcacaggacca

tgtgggcttaggcccgtcacgggccggttccacccattaggcccacgaagaccgggaccgccaggcccgg

gaccgccagagcccgggaccacgaagcccgggaccgcgaggcccggcccgttaggcccactaaggcccgg

gcccgggacaaaatacagcatta

>Os_CRATA Oryza sativa

tagagatggcaatgggtacccggtccccggtccccggtcccccgtggagaattctcccattaggtgatgg

tgatggtattaattgtgtctccatggggaatcaaatggagagaaagtattctccgtcgggtctggcgggg

gcgggtttggttccccgtcccccgtccccgccctctgtggaggcccgatataaaagtatgtgtgtagtaa

tatcctgtgtatttgacccatatggcccataaaaaccaggcccaaagcatagagtatataaagcaattgt

gtaaccctaaccattccaatccttatcccctccgtccccattccctagtccacgccgccgtccgtcggcc

acagcgactgagcggcgccggctcccgatctcgcgcccgcgcccgcgcgtgcaggcgtccgtgcgctcag

ggccgccgcgcccgccgtgtcggcgtccgcgcccgcgccgggccgccacgcccgccgtgtcgccgtccgc

tccagcgccaggccgccgcgccaggtcccagcgccacgccacgccgtcccgttcccttcactcccagcct

cccaggtccgaggacgccgtggactcggcaccggccaccggacgtcggcctcctcctccaccctccgtca

atatctgatccgtcatgtaggcttatttgttttgctgatggtggatgcaggtgaaggcttcggtggcaag

aaaggggaaaacgacgagtccacacgacgaccagccgctcgagtccgccatggcttcgccttcaccaccg

ctaagctctgacgatgatgcaaggtgagcacatctagctgtcctactcccaactcagtacccctccctag

acgcctacctatagtaccatatccccctccccctccactaaattcctcttccatggctgcatcattatct

cgttgagtcactaactcacgttcggcgatccacaataagggacgccaatgcggagttggaccagccatcg

catgctataagcgctgcagctgcagctgcagccacacccgctgtcgtgcctgttgctggatctgccattg

ttcatgtgggcagatctggttcctccaactccggtgcaaaggcctgctgtcggccgccgcaaccacctat

aaaaaggaccaccactaggtcaaaaagccgtcttgcgattgcaggagcaccttctgcttcgactaaggca

ggagctccttctgcccaagaactccagaagcttcatcttccgccaacggcctgcgcttattcaaatccat

ggggttcgatttcacgctaaccctttatacattgtagcttcactttcgtaatcctaagttcctaacaatt

tagcatgtgcaatttattaatttgcagcttgcttgattttcttagatattgaatgttgtatttagagtat

ctgtagaaaattagatatctttacatgtatattgtatggttgcacattgaatgttgttaagttataaata

tgttatgaacttaagtttttgaaattaaaggaatccgattctgtgtagtgtagatcataagaaatttatt

attctataggctgtagcagatttcagtatgatttatgtgtagagattcttcatttcaagtttgccaagat

tattctataggctgaaagatatattgatctatatttatttgtcatttgtcagatttaccacgaattcaac

tgctggtactggttgtgggagtagcagccagcaagaatcgcagtcacttccacacaagcagagtctgtta

atgattctgagccaatacaagttgaagatgatgagccagaagatgattctgagaattttggcacaaagag

gaagcttacttctgttgtttggaaggatttcaagaaagtcaaagtctgtggtgatgtcaaggctgaatgc

cttcattgccacaagcggcttggagggaagagcagcaatgggacttcacatctacatgaccatttgaaaa

tttgcacattgagaaagatcaagatggggccaaagacacttgcacaatcttcattgaggttcaattccgt

agagggagggaaagtttctgtggacacatacacatttgatccagcagtggctaggagagagcttgctgcc

atgataatattacatgagtatcctttatctattgttgaccatattggctttcgaagatttgttagtgcac

ttcagccattattcaagatggtgactcggaacactattaggtaatttcagttcatctattatttatttgc

tggttttgcatttctgaaatttgattagttagtgagtttattgattaatgtttgaatataggaaggacat

tatggatacatatgaggaagaaaagaaaagggcattagcatatatggctggagctaagtctagagtgggt

attactactgacttgtggacctctgataatcaaaaaagaggctacatggctatcacagcccatttcatcg

atgattcatggacacttagaagcattatcatgaggtacatgattcacaaatgattatcctcttgtcacca

tcttaaattcaacattttaatcttgctgcaatattccatttgtagattcatatatgtgcccgctccacac

acagctgaagttatttgtgaacatctttatgaagcgttggttgaatggaatcttgatgagaagatatcaa

ctttgactcttgataactgcaccacaaatgataaggtgatttctgaactcatcaaaaagatcggcaagcg

taagcttatgctagaggggaaacttttgcatatgcgttgtgctgcccatattcttaacttgattgtgagg

gatggtttagatgtgataaaagattcaattgcaaaggttcgtgaaagtgttgccttttggacagccacac

ctaaaagagtagaaaagtttgaggaaattgcaaagcatgtgaaagttaagatggaaaacaagttaggcct

tgattgcaaaactaggtggaattctacctataggatgctcagtattgctttaccttgtgcacgtgctttt

gaccgtgcaacacgggttgagaagctatttgattgtgctcctagtgaagaagaatgggcttttgctagtg

aggtagttgataggctcaagttgtttaatgacatcactgcagtattttctggcacaaattatgtgacttc

caatattcaactacttaaaatctgtgaggctaaagagcaaattaggaagtgggctgtttgtggtgattct

actatagaggaaatgtcagttgaaatgattcaaaagtttgataagtactggaaagatattcaagggccaa

tggggttggccaccattcttgatcctaggtttaagattgactacctccttgggttcattgaaaccatcac

tggtcagtcaagtgaagagtgtgcaacgaaagttgctgaggtgaaagacactctatatgatttgatgaag

gagtatgaagtggaagatgatgaggataacacggaatcttcagctcctccacttgtcaattctgatttat

tgtcttcaatcacagcacgtgttactagtaggagaccagcagcaatacgcgttaaatcagagctagacag

gtacttagaggatgagttggtttcaattaatacagaaaactttaagattcttgattggtggaaagtggct

gggacaagctttcctacattgaggaaggtagcacgagatatatttgctattcctgttagcactgttgcat

ctgaatctgcatttagcacaagtggaagggttcttagtgagcatcgtagccggcttactccagaactctt

ggaggccttaatgtgctcacaagattggctgcgaaacaagtatcgaggtacatatgatgttttattcata

catcatgcttagcatagattttacaatgcaatggatgcaaaataattgttataattgttatcttgtagct

gataatgaagaagcagcaagcttttggagttgccttcaagatatccaagatcggattgaggtattctttc

aacaacttgaaattagcaatcaaactcctattgccattatttgttatatccttataaaatacttgactaa

ttgaatatcttttgctacttctttaatttagggacttgctcttgtttgaagacatgaatgtatggacatg

cactgttcttgcaatgattttggatgtttgtaacccctgttatgtgtcatgataaacttgtaaaccatgg

caatgcttttggatatttggcacaaaagacctactccgaactatggttttacgtatgcatggacaacatg

tgttgttgctgcaatgtggtgataactaatgagtgatgactgatgaatgatgaatgatcatgtattattg

tgttgttgcttaatctgggaatatgatggatattgtattcttgggcacacttttgttgcactgcttgttg

ctgcactgctgttgtaagtgatgaattgatgatgtgcatttgtgaactgatgatgtgcatttgcgaattt

gtgatctgaatctgtgaacttgtgttcttgtagttgttgttcatgtgctgaaattgatggctggaattgc

tattttgagtgttaatcttttattgtttgatatgagaaatgctacatgttgactgttgagtaagttgata

gcggggatggaggctccacggggataaattacccgcacggaggcggtgatggggaaagatgttgccccgt

tgcatttcacggtgatggtgatggggaaaattccccaccgcggaggcgggtatggtggagtaacctccga

cggggaattccccgttgccatctcta

>Os_CRATA-2 Oryza sativa

tagagatggcaatgggggcccgttcaccatttccccgcggtgaattcacctattaggtgacaggggatgg

ttaatatccactcaccatggggaatgaaatggttaaaaacctctcaccatcggggatgacgggggcgggg

acggtgaaccattccccgtccccgccctccactgtgacccgactgtactgtacaagggaggaattctaga

ctttttagatgtattggcccataatattgaagcccatttgacaggcatatatatgatctaaccctaagtc

aacaggtcaaactcccacccatccagatccacaccacaccagcggccaaacaccacccgacccgagtcac

gcgcgccgccattcgccagccaccaccggcgccgtttgccagccaccacgcgcgcctccactcgctgcct

tctctcgccgggagactgaagctgccgtcgccatggactgaagccaccgttgccagctgccttcgtcaac

gcgccgccgggccggcgcggcgcctgcagccgtcttcaccgggagccgccggcaacctccgttgccggga

gccgccgtcgccgccggcagcctccttcttcaccaggagcagcctcctccttctgtaatttgctgatttg

ttgagtggagcattttggatatatttgatataatgtgactgaatatgttgtttaaccggggacgggttca

ccacaaggaaaaattcaccgcgtggtgaacggggctggagaaattatagccccgtcgtggttgatggagg

cggggacggtgaaatattttcactgcggtgacggggacgcgggcatgacccccgacggtgaattcaccat

tgccatctcta

>Os_DEBOAT Oryza sativa

taggggtgcaagtgggtcaaccgcgaacccacttatagatcaaaataaacgagcttgtgggttagtttag

cctataaatgggttttcgcgggtagccacttgcaccctta

>Os_DELAY Oryza sativa

tagggatgcaagcgggccgacccgcaagttcacttataggtcaaataaatgataattcatgggttttcgc

gtcgtggtccggtttgccgcatctagatatgcaagtggatcgatccataaatccatttataggtcaaatt

aataggtaacccgctggtcaaacccgcgggttacgtactaattttgcctataagcgggttcgcggacaag

cccacttgcaccccta

>Os_DS-RICE2N Oryza sativa

taggggtgaaaacggtaacggtaattaccggccgaccggagttcattttcgactttctaccggccgagcc

atatggaaatggtaatcgaccgaaacaaaaatagaaatggtaaaaaatatggaaatgaaaacggaaatgg

ttttgctgttataccgatcgtttccgtatttaccgtattcttgcggaaattaccgtttcttataatatgg

taattaccgtattctaaatatgtcaatattataggacatgtttatacttgacccacagcttatagattga

atgactcttcaataaaatctctaacttttgtacatggctaaaatgaagttaatttataatttatatagta

taagcttgaatttatgtatatatataacatacttatgtaaagttaaatatatgtttttatagtttaatgt

ttccgtatttgttaccggtttccgatctgtaccgacatgtttccgtccgtattgttccgtttccggtttt

ccgatatttccgatatcgttttcatttccgactttaccgtttccgatttcgtttccgagaaaaatatggt

tacggaaatggttgaggctgttttccgatcgtttccgaccgttttcatcccta

>Os_DS-RICE3N Oryza sativa

taggggtggaaacgagccgagccgagcccggctcggctcggcttgcattggctcgcaacaagctaggctt

ggcttggctcggctcggttagggaaacgagctaaaacttgagctcggctcggctcgtttcctggctcgag

ccagctcgagctggcttgcgagccttcctgttgaaacacctcttcatatatattttgtaattattaaaaa

aagtaataacaaatcatcaacatgtaaaatttaaataagtttatatattcaattcttcaaaatcttaatg

ataaatttcaagttgacaaataataacttcataaaaaataaaataatcgatataaacacatgatggccta

ggctcacgagccaaaacaagcggctcgcgagccagctcgggctcggctcgtttcagaaacgagctaaaaa

ggaggcttgggcttggctcgtttggctttcgagccgagccgagctaagcgagctcgagccaagcccgagc

tgagctcacgagcccgagctttttttccaccccta

>Os_DS-RICE4N Oryza sativa

taggggtgaaaacggtacggaaactttccggattccggacctattttcgaaaacggaatctgtcggtcgg

aatttttcggaaacggaaacgaattcggaaatattttctcggaaacggaatcgaaaatgataagggcagt

ttccgtcggaactcggaatcggtcggaaactttccggaaattttctcggaatttttggaatttttctcgg

aatttccagaaattttgtaactgaaataccctggattttttttttaagcactgaatagtgaataccatgg

tgttttgctgttatttttttttataaaaatatttgttatgcaaatctaaaattacataagaatatttttt

tcctgcattgggatttatcaacatcattactcttttaaatatagataatttatttcatagattgtgtttt

gtgatgtattattaatacttaaaaattagaaatatatgattttgttttataatgagttattcttcgttga

gatttgagaagcggatttatcagtttgaggggtttttttatattccgataaatattcgttaccgtattcg

tttcggttcgtattcgctccgtatttgtattcgataatattcgattccgttttcgtatccggggtttccg

attccgatttcgattccgaaaaaaaatatgaaaacgaatatgataaagctagtttccgtccgtttccgat

ccgttttcatcccta

>Os_F118 Oryza sativa

cagtggtggattcagaaaatcgattcgttagtgtcataacgtgtctatcggtgtcatagtatgttagtta

tgcttagatcatagtgttataacatgtggataaacagtttcgctatagattttgctgaaagtcgtcggtg

tcgcctgacaccgctcatactactgtagatccgcccctg

>Os_hAT-10 Oryza sativa

tagggatgcaagtggggcgggcttttttttcggcccgcagtcccgccccacaaaaaagcccgcaaatttg

cgggctggccatcccgcgtggaccttgcaaaattgtttggcccatcccgcacagcccgttgggcgccacg

gcattagggcacagactgggagacgcgagacgcagccgcagcggcgcagccgagacgggagacgggagac

ggcggcggcgagacggcgccgcagcggccgctactctcgtcttcctcgtcgcccatccatccaaatccag

cagccgacgccctccagtctcctgatccagcgaaatagcgaatccagtgcatttcatacttcagttcttg

atagttcttgatagatccatccaatccaaatcctgcagcttgcgacgctgcgctgccgcctccagatctc

tacacactaattaaggtacaagatcaaactaacctttcatgtgtttcttgtgtatttatgtgaatcaacg

aattgcttctccaagaatggcagcaggttgtctcactcactctatttgtttttgtatatgttaaactttg

agtgtgattggtgatctagccattgttatactggacatcagcgtctgcaaatgatctattgcatcttttt

tttatctaattaatgtggtgcatgatcagctaccactggtttgacataacaagattgataggtgctcatt

cagagaagtttccttcagactaatttcttaattattagcttcagatttttttttcttgttctatagtata

taaagtacagttacatgtagtagtaatattcaccccagatgttacactggataattattttttcatgttt

tccctgcatctgcattagttattcagaatttcatcttggtgaaataaacagaacacttacacaactgttg

gcccaatcagactatcagagtactgttctaatagaggaccaatcaggcacatgtgctgatagtagagtgc

tacgcattctttttattatttgcccttcatgatatatagtctatttgttttgatttttgaagtgctatat

agttcacaagtgctcgcaattttactcttttcataagtgctcacaattttactatgctcacaattttgct

cttttcagcacaaattttcttccatgtcgaccccatcatcgtttgaagctactgaagaggcaaccctttt

aggggcaaagcggaagtcttctttagatcctccttcagccaagcgaccacggtcacctattttgccatta

gtaaggtccccaggggtgagacacatgatgtcccaagtgagctctccatctgttagatctccaatggcaa

ttatcaattcttcaaggcaaagaactccatcaccagccccaacttctaatgatatcgatatgacaaatca

gacaagagcgactccttgtgggagtcacggtaagaaattgctgacttttctttgaaattatttgttctga

tttttgtcgttatctatccatgtctatgaattattatttttacattaattgattgcatgcatgtactgca

gttatataatatgtatgcaatagccattaattgattgttacagtaatctattcatgtctatgaattaatt

tatattaattgattgtgtgcatgtactgcaattgcacaatatgtataaactgaacatagagtacttgtcg

aaatgtcgctggtatacttataatattcgtataatacttgtcagtgcagtatttttactttacttgtcac

tagtgtatttatagcaatatttttgtgcttagccacagggagagcaagaaaactgaaatctacaatgtgg

aaagaatttgatccaatacgtactaatgataaactatcacatgctaaatgtatccactgcaataaggttt

tcgtggctagccgatcttctgggactagtcagtgccttagacatttgaaagtttgtaaggtcaggcttag

aatgcaccatttgattgagcatatgcatgccaacttgtcccctaccgctgatgttatgaagaattggaaa

tttgaccaagaagtgtcacggaaagaacttcttaggatgattgtgttgcaagagttaccctttagcattg

ttgagcatgttggatttaggagatttgttgcaagtttaaatccatattttaaggtgatatcaagaaccac

actgagaaatgattgtatggctgcttatgaggatcataagttagctttatttgatgtcttgaagagctca

aattctcgagtatctcttactgccgacatgtggacttccatccagaacttaggctatttgtgtgttacct

gtcattacattgataatgaatggaagttgcaaaagagaattataaagtttgctctagtgccgaccccaca

tgatggcatcactatgtttagtgaaatgctaaaggccattcaggagtggcatatcgaaaataagttattc

agtgttaccttagacaatgcaagtgtgaatgatacaatgatgactcatctgaaaaccaatcttgttggta

agacaatgttgccttgtgatggagtgttgttgcatttccgctgtgctgcccatatattcaatctaattgt

ccaagatgggctaaaaaccatgagcaacgccattaacagtataagagaaagtgttaaatatgttcggagt

tcgcaatcacgtggtcaaaggtttgaagagatgattgctcaagtgggaataaaaactaacagacgaccat

cgcttgatgtatcaacgagatggaattcgacatacttaatgctcgagtcatctttattggtcagaatggc

ctttgaagccttggatcggcatgacataaattatttgcatcaacccttcgattatcaatggacaatggct

gagaagctttgtgctttgttgaaagttttctatgaagctactgttgcagtatctggtacgttgtacccaa

cttcaacatgctattttcatgaactttggaagataaagatggttttggacaaggaggctacaaatgaaga

tgtcaccattgcatctattgtcaaggaaatgaaagaaaaatttaagaagtactgggacgctcaatacttg

caaatatgtttcccggttatttttgatccaaggtataagtacaaattcattgagtttcgtttgaagtctg

catttggagctgctgcaactccttaccttaaggaaatcaagagtaatatgcagaaattgtttgatgaata

ttctgccaagtatgggggctcaaacaacatcaattctcagcccgaaacaagtgttgagcagaatgttgat

gcaagtaatcaattcgctgattggagacagtttctacatgacaaaagcaggagcaaagtaaagagtgagc

ttagtcgatatcttgctgatatgcctcaggaaggtgatttccaagatgggcatgattttgatatcttgaa

ctggtggatggtaaacaaaacaaagtaccctgtgatttcacgaatggcacgtgatgttttggctattcca

gcaacatcagtggcatcagaggcagcattttcaactggagaaaggatcattagtgactatagaagtagac

tttcgagcagtactgtagaggcattgatttgccttcaagattggatgagggcagaaggtttgggtgattt

ctttgcacgtgatcttgctgagagtgatgaccaaaatgtgcaacattcaggtatttaaattgagattcgt

ttgaattaacttggctattctatatttttgtatagttgtatactacaccactaacatcattgtttctttt

ttattaacatccaacagtccagcatgtactcttttttattaactccattgtttcttcctatacaggtgaa

aatgcctatacacctctagtaacatgtagtaccagcacaacaaccagcagcaggttatatatctcactat

cctcgataactgcttgttattgtcaattatgtcctttaattcatattttggatgcattcttaattgatga

aacttagtacatatttactccattctacaatatggaataacgttgtgatcagtcggttgatgaattcatt

tctaattttcgattagtagtttctccactctaactttattaaatttaatgtgttgaaggtaatatagaaa

aagaccggaagagctcatggagttggaagcagttgtttgtcactttgtcaattgggacagttggtttatc

atgaagagaaaatgcatcatcatcactctaactttagtcatgatgttgaaggaaaagaatggtgttctta

ttcagtactatacttgttatgtcattgaaaaagaatactttcacatgaaaataatacagttttcatgtgc

taaatttgggatatggagtagttatgtgcttgtgtgattgatgaacctaattaaacagagatgtgcacat

tgtgggctgtccagcggtgcccgtcgggctgagcaatcccgccaagccgtgcggggcgggctgccgtttg

cccgcacatgcagtttgcggctggtgtggggcggggcgggacggcccacaacccatcccacttgcaatcc

ta

>Os_hAT-11 Oryza sativa

ttacccaggcccaatttgttaccgatttcagaatccctctttccccgtgggtggtggtgtgctggctacg

ttgttgggttagggattcttttcggggggaagcgcggcctgcctggaaatcgttggagctcgctgagatc

tcgagttgtttggcatgaatttcgccgtggggctggatccgctgggagatccggagagggcgacttgatt

cgttcgttttggaggagatatgtgatgttggacttgctgcgcttagataattgaatctgaatgtgtgctt

gtgtggtcgtgcagagaaatttatctttctttgtccaaagaaagctgtaccactagctaccttcttagcc

tcttcagctaggttcttgctactttgtactagcttcttagcatcttaagttaggttcctgccgctgttta

gctaactaggtgagcttgattttcaagtcctgtataagtgtcccgccaactcaagcaatacatagttttc

tcccttctcaccgattattttgattattttgagttggtgcccacgaggaacaagaatgagttgacttgca

ttgcatgagaataattagcaagatctgaattatgcactcctaggttgacttgcattgcatgaggataata

agcaagatcttagttatagggagtaaagttagcaactaagtttgcacgagtcattctggtccaattagct

ttgcatgtgatcccttcttttcttgaaatttgtcagcgaaacaaacgtagcacatggttctgctaatgaa

gtttgcattttcggtggcaaataaaatttaatttaaattgtgtactttaagcatttttactgaggagtga

gaagagatggttacccattctaagtttccatatattcctggatgacatctataattttgttgtttcacct

gtgctccagttactatatataacctggtttgctaatttgttatcaagaaatcgtgttgtctttcttttat

cttcctgttttgttaaataggccactgtagtgatactggtttgttattgaaacaaccgtgtataagtcaa

atttgattatttttttggccaaatgattgttattctgacttctgctgtcaccatgttactatttgacaga

ctttgtgagttctagctactagccaagaagcatggctgaggaaactggcaacgacaaccaggtggttcaa

ggtaatgagattgtcccaagcaatgaggaagctcaagctgaggaagttcagggtgatgaattggtccctg

ctgaggacttaactcaaggtgacgaggtccaaggaaatgaattggtcagtgctgagatcagtacccctcc

aacattaaggcgccgtaggaagaagtctctagtgtgggagcacttcactattgaagctgtctctggaggg

gctacacgggcgtgctgcaaactgtgcaagcaaacttttgcttacagctctggttcaaaaattgcgggta

ctagccatctcaagaggcacattacattgggttcatgccctaaaattaagaaccaagagcacaagctagc

actgactccagctggagggactgacaatgatggtgagggtactgtggagcgcccatctaagaggcgttac

agatatactggttatgcaaatgctgcttttgatcaagaccgcagttgctcatatctggcaaagatgataa

ttttgcatgactacccacttcacattgttcaacagccagcgttcactacctttattgacagtctgcagcc

acgtttcagggttgtagatgttgagacaatggagggggaggtgtatgctgtttaccagaaagaaaaggaa

aacctcacgcaagcattcagcactatgcctggaaggatcagcctcaccattggattgtggacaactagcc

aaactcttggctatgtttcactttctgggcagtttattgactctgagtggaagatacatcgaagaatgct

aaacttcatgatggtgtcttctcctcattcagagaatgcacttagtgaagctattagtgcaagcctttcg

gactggaatatgaaggacaaactgttcaccatcacattggacaatgattgctcatcacatgatatataca

gtgcaaatctgagggattatctctccaacaagaacaacctcatgctcaagggccaactgtttgttgtaag

gtgttatgcccatatcctgaatgcagttgctcaggatgtcattgcttcaatccatggtgtcatctacaat

atccgtgaaagcatcaagttcataaaagcttctcctacccgtgaggagaagtttgcagagattgctctgc

agctagagatcccaagtaccaagaccctttgtctggatgttacgactcagtggaacactacctatctcat

gctgctggctgccttggattataagcaggccttttctactctagagacaagtgatgacaactacaacgag

gcaccgtccgctgaggactggaaaaaagttgaggctgcctgcaattacttgaagctattgtatgactcag

cacatagcatcatggctgcagcaaatccaacttcaaatctctttttccatgaggcatggaaacttcaact

agagctgtcaaatgctacagggcatgaagaccctgttttcagcagcattgccaaggatatgcacgagagg

tttgacaagtactggaaagattgcaaccttgtgttagctattgctgttgtgatggatccacgcttcaaga

tgaagcttgttgagttcagctactcgaaaatttatggtgttgaagctgcgaagtatgttaaggtggtgga

tgacgctgttcatgagctttacaaggagtatgttgcacagcccctccccttgacgccggcctatgttgag

caaggggaaggtaataatgcacctgctagcgagaatagtactcaaacaactgctccttcgaccggcgatg

gacttgtggactttgatatgtacctttctgagatagctacaagccagccaacaaaatctgaactggaaca

gtaccttgatgagtccctcactccgcgcatccaggaatttgacattctgaactggtggaagctcaacact

ctcaagttccctactctctcaaggatggcccgggatatcttggccattccgatgtcgatggtgagcagcg

gcaactctattttctctgctggaacaggaactcgcatgcttgatgactacagaagctcattgcgtccaga

aattgtggaggcgctcgtctgcgccaaagactggcttcagtatttaccagctaccccggaggcgccgagt

accacgcttgtcaaggtggatgcaccataggctcgccgtgatccccccccctcccccccttcctcttgtg

aagcaacagcatcctatccgtattttaggcagggactatgaactgattgagatccgagcttatcttccta

gggtgtaatgtgctgtatcgtactctgttaactccttcatgttattatgtacgctttgtcttgtagtatg

ccatcggagttacctgacattacgtgtagtgtgtgcgctggctgcataatgtgtatcagttgatgcctta

tgtctggacttcccttcattatcatccattgtagcatgtgattgttctgtgccgttgagatatgttcatt

tgattgtcaatcaatgcaacgtgtcaattttactcgtgtgtt

>Os_hAT-12B Oryza sativa

tatagatggccataaggcccggggcccgacggcccggcccaaggcacgacaaattggcccggcccaggca

cggcacggcccgactggggtcgtgcccgtgccggcccggcccaacagccgggccgtgcttgggccgctcc

ctcggcacggtgggccggcacggcccggcacgaaagaggtcggcacggtgggccggcacggcccggcacg

gtccaacggcaggcacggcccgaccgaggcctgttatgccgggtgggtataaataccccaaaccgcatca

aaccgacaaaccctaaccctagccacatcagtctggagtctcgaccgatccgccgctcgcccgctccgcc

tccacctccaggctccgacccctcagccctccacgcctccatccctcttcgtcggcgtctccgatggatg

cctcgccggatctagcaccgagggtcagcgggctcccgctgggggcttgagggtcgtctcttctctatcc

ttttctttcatctctttggctgatctgtgaccgtgagctcttcgtctccgcctctccggtcaccggatcg

acggtcgcattgtcgatttcctctccattctgtacttttttttgcaaccctcatcgtttctcttcgtcag

atactcagatccactcatccaccccccaaatctagtctctcctcttgtcttctcgtagatctgtggatct

ctatcatcaacggcaactccggtgctccggatgcgcaggtacaagctttttttttagtttattttctcta

ttcgatcagatccgtaactactctctgctctcgtcgttgtttcatttgttcttgcaggtcgctgagcggc

aggcggcagccaccgttgaggaaccggagtgcctgcgatggatggtgatccaacctcatgcaactacgag

gcgagggtgatggggacgcagggtgatgacgaagatgacttggaggaggaacgcatcgaggtgtttggca

acactgcttcacctctgagagatctgtcgcagccggagccacacgatgatggtaccggtgccgatggcaa

cggtgctccgtctggctcaagtgctagcaacaagaggtccaggtctgaggtatgggacgactttgaagaa

ctctttgaagagcgcaatggcgctcaggttcgcgtttctgctaaatgcaattactgtcataaaacattgt

ctgctcgttcaactggtggtactggacatttgcttaggcatattaaatcgtgcaaacctagaaatgttgg

tgctttgtctcagtccatgcttaggtttaatgctgatggttctgttagtcaatgggaatataaaccggat

gtggctagaactgagttggttaggttgattgctagagaggatcttccacttacttttggtcaatctgctg

cttttgaggagtatattcagaatgctcacaaccctagatttagtgttgtctctaggcaaactatcagtag

ggacgtttttaaggtcttcgacaagagacgtgctatgcttattgatactttgaagtctgttagctctgtt

gctttgacatctgatatatggtctggtaatgctaaggaggattatttgagtgttgttgcccattttgtta

attctaattggcaattagagaagaggattttgggactggtgctaattgatgtgtcacacaatgctgaaaa

tatttctgaacgtgtgctttctgttgttcaagaatatggtttgactaataagatcttctctattaccctg

gacaatgcctctgctaattctaaagcaatggatagtcttaaacctgctctgtctggttatattggtgatt

tatatttgcatcagcgttgtgcatgtcatatcatcaatttaattgttaaggctggtctagaaatcttcaa

acccatgcttcaagattttagaactgctatttcatttataaatgcatctaatcagcgcattgctttatac

aaaaacttctgcattgctaaaggtattcgtcctcgtaaatttggtttggacatggatgttagatggaatg

caacttatctaatgcttaagcatctcttgccacatagggtcattttttcagtgtttattgcatctcacca

tccaatggctgatggtcagccattactgactgatttacactggacaattgctgaaactgttcttttattc

cttgaacaattttatgattcaactgttattttgtctggtgtttattatccaacatctccattaattatgc

atcacattcttgagattgctggacatctaaatacttatgagaatgattggaaccttagaaatgttgttgt

tcccatgaaaaataagttcttgtcatactggtctgagattccatttctgtattcatttgcttttatcttg

gatcctagggccaagatcaggggttttagcaatgttctccaaattatgggacaacttattagtggtgatt

actctgcatatttaaatgaggttagggctgcattgtctgacatgtatgctaagtatgaaagtaaatttgg

tgctgtgagattacaaagggcaaccccaagctcttcttctggtaagaaaaagacagcttgggggaaaatt

tttggtgctgctgcttgtggttccagtcttggtgctggacttggtgctggtaatgctggtgctagtgcca

gtccaggttctggatttggtgctggtgccagtccaggttctggatttggtgctagtgccagtccaggttc

tggacttggtgatgctggtgccagtgcaggatctggacttggtgctggtgcattttctaggagaacttct

gctaccgctttgattcaggctgtttcttctactgctaacatgaatgcatctgagttgtttgcctatcttg

acagtgacactgtcaaccaatacgatgatgacttcaatatccttaactggtggcatgagcacaaccacac

ctatcctattctttccatattagctagggatgttttgactgttcctgtttctactatatcatcggaatct

gcttttagcttaactggcaggatcatcgaggagcgacgacggcgtctagctcctgacatggtgcaagctt

tggcactaatcaaagattgggagcaagcagatgcaaagatgcaacataccatggagaatttagagcttat

aaactcttttgacaatttgtttcttgatgaagtaaccactgccactgggactggatgattgtattggact

tggactttattatgaacatttgattaatgctgtgggctgcactcttttttcctttctagggttttctcac

gaggtgtgagtttttacctagaaaggtttttaacgaggcagccattgcactacacagctactttattcat

tgaaatacttgtgtcaagtttttgggtgaaagtttatgattttgggtaatttttgacttagtgaaacttt

tgaatcttagaactgtgatttctgtgaattgtccatgtcatctgtgatctaagtcaagttttttgggtga

aagtttgggtgaaaatttgtgattttgggtaatttgacttggtgaaatttttgaatctacaactgtgagt

ctgtgatttttgaaactcttctgtgaattgtccatgtcatctgtgatctaagtcaagttttttgggtgaa

agtttgggtgaaaatttgtgattttgggtaatttgacttggtgaaatttttgaatctacaactgtgagtc

tgtgatttttgaaactcttctgtgaattgtccatgtcatctgtgatctaagtcaagttttttgggtgaaa

gtttgggtgaaaatttgtgattttgggtaatttgacttggtgaaatttttgaatcacaactgtgagtctg

tgatttttgaaactcttctgtgaattgtccatgtgatatgtgatcttagtcaagtgatctatgaattttt

gtcaatgtgatctgtgaatctttgatctgtgatctgtgaatttttgtaaaggtgatatgtgaatctagaa

ctgtgaattcaactaaggcatgttcaaatttgaatttgtcaactaagttgtgtaagttgtgttatttgaa

tttgttaaccaaggcatgttcaaatttatatttgtcaactaagttgtgtaatttaaatttgttaactaag

gcatgttcaaatttgaatttgtcaagtaagttgtgaaatttgaatttgttaactaaggcatgtttaaatt

taaattttgcatgttcaatccattgatccattgaatttgtcaatccactgaattactggtccactgatcc

actggtcctaagccgtgcctgggctagcccaggcccaatgggctgatcgtgccttcgtgtcggcccggca

cggcccgattgctactgggccgtgcctgggcctgacgtgcggcccatgggcaggcacggcacggcacgaa

attccaatcgggccgtgccggcccgatcgctctcgggccgtgcctatccgtgcctgggccgggccgtgcc

gggcgggccttatggccatctata

>Os_hAT-12 Oryza sativa

tatagatggccatatggcccgcagcccgacggcccggcccaaggcacgggcgtttggcccggcccaagca

cggcccggcccgtcagtgttcgtgcccgtgccggcccggcccgacagccgggccgtgcctgggctcccgc

agcagcacgctgggccggcacggcacgacggggccggcacggcccaggcctgtataacggtaggcacggc

ccgacgaggcccatcgggagccgccgccgcgcgcccgcgcctcatatataaaggccgccgcgtcctctcg

cctccgccagtccgcctccctcacaaccctaaccctaaccctagccgcctccgccgatccacgcacgcct

ccgcctccgcctccgccttgaccgctccgcctccgcttccgcctccgtcgatccacgcctccacctccac

gcgaccacgcctccatctctccatccctctccgtcgccgtctctgatgaacgcctcgccggatctaccac

cgagggtcgccgcctcgccggatccgtgaccttgtctcgtctccgcctctccagtcttcggatcgtcggc

cgtgctctcgatttcctctttctttttcattttttttgccactctcgcccgctcgccgtttctccgtgag

ctctttgtcasatcactccccaaacccagtctctgctmtcttcttctcgtagatccgtcgatctctatca

tcaacggcaactccggtgctccggatgcgcaggtacaccgtacacacctatctctgctcctctctcctct

cttggttttctacttctctccggtccctaactgctctcttctctcattctgtgtgtcttgcaggtcgctg

agcggtagccgccgttgaggaaccggagtgccgacgatggacgatccatcctcttgcaaccacgagttga

ggttgatggggatgcggggtgatgacgaagatgacttggaggaggaacgcgtgcaggtgttcggaatcac

ttcgtcacctcctcacttcgatgggtcgcagccggagacggagccggacgtggacggtaccggtggcggt

gacggtgatggtggcwcaggcgcgccgtccggcaccgactccagtgctaccaacaagcggccaagatcat

ccaaggtatgggatgacttcgaagaactctttgaatcacgcaatggcgcgmaggttcgcgtttctgctaa

atgcaatcactgcaaaaaaaactttgtctgctcattcatctggtggcactggtcatttgcttaggcatct

caagtcatgcaaacctagaactgctagtaatttgtctcagtctatgcttaagttcaatgctgatggtact

gttcgtccatgggaatatgatcctgattatgctagaactgagttggttaaattgattgctgtagaggatc

tgccacttaattttggtcaatctcctgcttttgaggagtacattcagaatgctcataaccctagattcca

wgctgtctctaggcaaactattagtagagatgtttttaagtactttgacaagagtcgtgctatgcttatt

gagaggttcaagtctgttaactctgttgctttgacatctgatatatggtctggtaatgctaaggaggatt

atttaagtgttgttgctcattttgttaattctgattggcaattagagaaaaggattttgggtctggtgct

gattgatgtgaagcacactgctgagaatatttctgaacgtgtgctttctgttgttgaagaatatggtttg

actgataaggttttttctattactctagacaatgcctcttctaatactaaggcaatggattttctaaaac

ctaagttgtctgcwtatgttggtgatttatatttgcatcagcgctgtgcatgtcatataatcaacttgat

tgttaaggctggtctagaagttttcaaacccatgcttcaagattttagaacagccatctcatttgtgaat

gcatctaatcagcgcattgctttatacaaaaactggtgtattgctaagggtgttcgacctcgtaagtttg

gtttggacatggatgttagatggaatgcaacttatctaatgcttaagcatctcttcccacataaggagct

tttttcattgtttattgaaactcattatcctagggaaaatggtagactattgcttactgatttgcactgg

acaattgctgaaactgtgcttttattccttgaacaattttatgattcaactgttattttgtctggtgttt

attatccaacatctccattaattatgcatcacattcttgagattgctggacatcttaacacttatgagaa

tgatawtaactttagaaatgttgttgttcccatgaaaagtaagttcttggcatactggtctgagattcca

tttctgtattcatttgcttttatcttggatcctagggctaagatcagaggttttagcaatgttctacaaa

ttatgtcacaaattttgacctctgattactctacttatttaactgaggttagggctgcattgtctgacat

tttctctaagtatgaaagtaagtttggtgctgtgagattgcaaagaacaaccccaggcagtactgctggt

aagaaaaagatagcctggggaaagatttttggtgccagtgatgcacttggacatggtgctggtgccagtc

caggttctggacttggtgctggacttggtgctagtgccagtccaggttctggacttggtgctggtccatt

ttccaggagaacttctgctactgctttgattcaggctgtttcttctaatgctaacttgaatgcctctgag

ttgtctgcctatcttgatagtgacactgtcaaccaattcgacgatgacttcaatatcctaaactggtggc

atgagcacaaacacacatatcctgttctgtccatattagctagagatgtcttgactgttcctgtttctac

tatatcgtcggagtctgcttttagcttaactggcaggatcatcgaggagcgacgacggcgtctaggtcct

gacatggtgcaagctttggcattgatcaaggactgggtgcaggcagacaagaagttgcagcacactgctg

agaatgtagagcttataaaatcgtttgagaatttgtgtcttgatgatgtaaccagtggcagtgccactgg

aactggatgattgtactggacttggactttaatatgatagttagatgaaagctgtgggctgcactctttt

ttcctttctagggttttctcacgaggtgtgagtttttacctagaaaggtttttaatgaggcagccattgc

actacacagctactattatttttgaaagtgtttgtgacttgatcacttgagtttgaactgtgatttgtga

actgttagacttagtatttgaaagtgttttgtgattgcggatttgtgacttgagttgtgaactgtgagac

ttagwagttgagttagtgaattgaattgtsatattgcacatactatataacttgtgaattgtgatattgt

aatctgagacttgtgatattgtgatctgttaacttgtgaattggatggttttgtgctgatttttgtgatg

gacatkgcattttcgatatgtatgtgcaaaactgaagtgaacaagtggcagttagtgtttggcgtgcctg

ggctggcccgggcacggtgggctcakcgtgccgtcgggccggcacggcccggcacgactgctgtcgggcc

gtgcctgggcctgaggttcagcccgtgggcaggcacggcacggcacgaagtgccaggcgtgccgtgccgg

cccgatcgcctcgggccgtgcctaggccgggcctgggccgggccgtgccgggcgggccatatggccatct

ata

>Os_hAT-13 Oryza sativa

cagcctgcaagtggggcgggtcgacccgttaggtccgtggcccgaacctaaatttgtggcaccaataggt

tttcgggccggcccatattttcttaggtgacaatgggtccaggagtcagttgaccctaggggtttttggg

tcggccttgttcgagcgctccgcttgctcgcgttcacatggcggcggcggctttagggctcccggcctga

ggcggtgcggctccaccgcggatcgagccggcggcggtggctcccggcggaggcggcagcggtggcggcg

cagctccatcacggatcgaggtggcggcggcggctcccggcggaggcggggcggtggcagcttcgacttc

gcggtgtgctccaccgcgttttgaggcggcggcggctgggcgtgctccttcagtcctcctcccgtctcca

actccccccaagactggtactgtggttcacatcatagcaactagcaacggattctttggttcttgatttt

ctttttcttttctttggttcttgattctttggttcctcttctatgcagttccttctttcggcggcggatg

gaggcaccggcgggcggcggctacctgcttggtggccaaccggcgaggtgctccggaggacactccaccg

gcagcaacggcgagggtttcggaggacacccggccacatcgccaccttcgtccagctgggtttctctccc

tcgtgagcacaggtatgatttcctccatgaaccctgtcttgttcatctgtcttgctcggttttggtttga

tttgtatgtatagtgtctcattcaggagaaagtttcagtgactgttatagctaggctttcatgccatttt

gctaggcccagatttgtatgccaggaaaaagtttcattttgttttcatgccattcaggagaaagtttcag

ttactgctaatttaaagtttcctactgccttgtgtaaaatagatggtcttttactttgtctctgtataac

ctggattttctcaatattcaggatatactgaacgaaacactgaataagtagcatttttgcatgcttctga

caacatgctgttgtgcagatttttacaccaaaatttcctttccatacattatgtgatagaaaattatttt

agatggcaaatgatacatttgtgttgttcgttgctttgtttcaagtgaggaaatgcggacacccaacgca

ttttttggacttggcactaacagtggtgaagatattgagatgggtgatggtgatagtgactctgaacaag

ttggtcctgtagatgagcatgtgaatccagtaatacaagctttaacaaggaaattcagattgaggcttgg

aaagagtttgtgccgatactcattgataatgaagttggtgcaggaaaatgtaagcactgtgatacagaga

tccgtgcaaagcgtggagcgggaacaagttcattgaggaagcatttgacaagatgcaagaagcgaattag

tgctcttaagattgtgggaaatcttgactttacacttatgtctcctaatagtgtaaggttaaagaattgg

agctttgatcctgaagtttctagaaaagagctcatgcgaatgattgtattacatgagttgcccttccagt

ttgtggagtatgatggatttagaagttttgctgctagtcttaacccctacttcaagattatttcgagaac

aactataaggaatgattgcatcgctgcttttaaagagcaaaagttagcaatgaaggacatgttcaagggt

gcaaattgtaggttctcattgactgcagatatgtggacttcgaaccaaacaatgggatacatgtgtgtga

catgtcatttcattgacacagattggagagtacagaagaggattataaagttctttggagtaaagacacc

tcatactggagtgcagatgttcaatgctatgctcagctgcattcaagattggaatatcgcagacaagata

ttcagtgttactttggatatgcttcagccaatgattcaatggctaagttgttaaagtgcaatttgaaagc

taagaaaactataccagcaggagggaagctacttcacaaccgatgtgcagcacatgtcatcaatctcata

gccaaagatgggttgaaggttattgattctattgtttgcaacatccgtgaaagtgtgaagtacagggata

attccctatctcgcaaagaaaagtttgaggagatcattgcccaagaagggatcacatgcgagttgcatcc

cactgtggatgtatgtactcgttggaactccacatatctaatgctcaatgcagccttccctttcatgagg

gcttatgcttcattggtgttcaagaaaaaaattacaaatatgcacctctcctgatcagtgggaaagatca

accatagtctctggaattttgaaggtgctttatgatgcaactatggtggtgtctggatctttatatccaa

catcaaacctttatttccatgagatgtggaaaatcaaattggtcttggacaaggaacgctctaacaatga

cactgaagtggcaagtatggttaaaagatgaaggacaaatttgacaagtattggcttaagtcttacaaat

atctgtgtattcctgtcatttttgacccaagattcaaattcaatttgtggaatttcggcttgggcaagcc

tttggtgaaaatgctaaagagaggattgacaaagttaagaagagaatgaacatgttgtttaaggagtact

ctgataaactcaaggatagcaatgctaacccactgcgccaagctgaacatgtgatggctatctctgaaaa

tgatcctatggctgattgggttcaacacatctctgaacaatgaagtgagcaagttgatactgaattggat

atctacctcaaagagaatcctattcaggaatttgggaacaaatttgacattctaaattggtggaagacca

atcgctcaaagtatccaactctcgcttgcatagcacaggatgtgtagcatggcctgcttcaacagttgca

tctgagtccgcattcagcacaggatctagagtgatcagcgatttcagatgtagccttacaatggattcag

ttgaggcgcttatctgcctccaagattggtttcgagcttctgccggtatacacgactgccctcgttcatt

tacttctagtatttatcttgctaaaactaatttatttttatgttttgattgctaggtcctaacataaatg

tgagctcagtaaatgaaatcaactacagtgataattttgtggttagtaaattgcttgtgctcttcttatc

atctgtctatttaattttctgaaagagaaagatgtctatttattcagtatttgcttgattgcttgcaaga

tctcttccagcttttctctttctatttatccagtattgagcaatgcctatatgggtttgctgtcctactt

gctagctttctgtaaaattggaatatgaagtttcaatgtctctatcatgtattcatctttatctatagaa

cctggacctggaggattccatggatggacaggatggtggaagcttgtgaatgcccaaacaaagggtctgg

atattgatggttagtaatctatcatgaacttgtattatactatttataatctgagctagttcaactcttt

acaacatatgagaagctctagatgtacaaaaggaataagcctcatttctggtattgcaatgctctgcttt

ctgaaataaaacgatagcctaaaaatagactgtactgcactactgctagataaatgaagaaaattagtaa

aatctgcaaatttgtgtagcacctgttagatgattcttgtattccttaatctctgattgtgaatactatt

gggtgactgcagattacttctgtttttcaagcatgttttatatgtcaaatactcaaatgcttagaacacc

tactgccttgattcctctgtctgtctgcatatgctcatgtcttgattcagttgttgcccataaaacataa

atgcaccatttctttttctgagagatattgcaggttattacctttttaaaacaattattgtatattttct

tcctgtcattataccatgatcattcgatctctccatcatgttaacctgcagaatctggacctgggtgatt

caacgaacagtgcaagctagggaatgccaaatgatgcccactttgatcttggaaggctgcattttgtttg

atctcaattaatgaaccggatatttgggttgttcaaatcgatttttgtgaatgatatgagtacatgcatg

tacttagttttaattgaactgggtgccagtacccagtgattgatgtttgtacttttgaatgcaaaagatt

aagtcatttcggaatgtttgcattgggtatacagtgtgttctggtatggtttttgattatttatttattt

tgctgtagtgctgttgatttgcttcgcacaaccgaaatttctagtatgttagtatgtgttcagatatcat

gctagtgtatgtgttttttttccttatttcacttgatatctgacttatataaaattagtatagtacaaaa

atgatatcagtttaatataaaatgattgttcatattatttttgaatcttgtataattgaattgacgtata

gctacttgctttaatggttcactgataacacattaacacagccagtcaaccattcactggacacggtggc

tcagacgctcagtgcatccggcccgcggctacggtgggtcggccacgaacctaacgggccggctccactt

ccaccagagacggggcgagctcaggcaccaagatagatagggtcagagggtataggggatgggtcggccc

gatccgccccctttgcaggctg

>Os_hAT-14 Oryza sativa

tagggatggcaatttcccccgcggggccgggtcccctgcggggacccgcccctacgggggcgggggcggg

gccgattttcgacccgcgggtgcgtcggggacggggccccgcatttgggcggggaggggacgggggactg

atttcacccgcgggtgacccgcggggccccggggagtacatatagacctcaaaatatagatcaaagaaag

cccaaaaagcccagcccactaaaaaaatccttaaccctagcagcactgcagcacagcagcagtgcagcac

atccctcagtctctctcctccccacatccattcccctcccctcccgtcatccactcatcccgtgcggctg

tggcgtgagccgcgtgacctaggcggcggcgcgcgctcgtgggcggctcgccggctcgcctctggatctc

gccctctcggcgtcgccggcgcctgccagccgccaggccaccatctcctctcccctcctctccagtctcc

cctccggcgtccggcggctcgagctcgtcctctccctccggagtcagcggcggcggcacacagggcggcg

ccgcggcgggccggcggcggcgcacaggcgcggcggggaggcgacaccggcagcaagggcgcggccggcg

gcgcacagggcggcggcggcggcgcacagggcgcggccggcggcgaggttgcagcacatcacaaagagtg

agaggtattgctccatctccaatactccaaatgtgccttgtgatttgtgaatgaaattatgaaacagaag

attgtgatctatttcagtgtatatttcagtgtatatttggtagatttggtagtacatagcacatagcagc

agattgtgatctattattgaattatgttatttgtggacagaatgtcgaaccctgctgccaccgttgagtc

acaagttgcattgacaccatcttccacttccactcccactccaaccaatgctccaagcgtcgaaataaat

gattcacaaggtacagaagtagaacatgataataagagactgaaatctgcagcatggcaagattttgtta

agaagaaaataaatggtgcgtggaaagctgaatgcaaatggtgtcacaacaagcttggagctgaaagtag

aaatgggactaaacatttgttagaccatattaaaacttgcaagtctaggcakgctagaaaaggtctaaca

caatccaatttgaagatgggaatagatgcagaagggagggttaccgtagggaaatatgtctttgatcaag

aggttgctaggaaagaacttgcactgatgatatgcctccatgagtacccactttctattgtagaccatgt

tggttttcgtaggttttgtggtgcattgcaacctttgttcaaggttatgaccagaaacacaattaggaag

gatattattgacttgtttggggttaacaagatttctatcagtaattattttcacaagcttcagagccgtg

tggccattactactgatttatggacagcaactcatcaaaagaaaggatacatggcaatcaccgctcactt

catcgatgatgaatggaaattgaagagttttcttttgaggtatattgaatatgtgctgcacattatgcta

tatcatgcttcttacatttgtttaactctatactaatgttctcttgtggtgttttatgttttgtaggttt

atttatgtaccagcacctcataccgctgatcttataagtgaaattatttatgaggttcttgctgattgga

acttggagagtaggctgtctactatcacacttgacaactgcagcaccaatgacaagttaatggagaacct

attggggacaatgctagataagttgcctgctgatactcttatgttaaatggttcattgctgcatatgcgt

tgttgtgcacatattttgaatttgattgtgaaagatggaatgactatcctagataaaatcattgagaagg

ttcgtgagagtgtttcattttggacagcaacaccaaagagacatgagaaatttgaaaaacaggcccaaca

aataaatgttaagtatgagaaagtaattgcccttgattgcaaaactagatggaattctacttatctcatg

cttagcactgcagttttgtatcaagatgtctttacaaaacttggaactcgtgaaaaaatgtatactcctt

attgtccatcaaatgatgattggaagtttgctagggagctttgtgatagattgaagattttttatgatgc

aacagaggctttttctggtagtaagtattgtactgcaaatcttttctttcctaaggcttgtggtatttat

ttggctatgagaaaatggtcaactagtgctgatccaaacattataacaatgactaaattgatgtcagcca

aatatgagaagtattggaaggatgtgcatggtattttggctattgctagtgttttggatcctagatataa

acttcatatgttgaatgctatgtttatacaaatctatggagaagaggtggcactaagaaaagttaatgct

gtgaaagaagacttgtataagttagtgctacaataccaaaatcatgttgaggagggtgttggaacttcag

atggagttaatgcttcttcaagtgttgcccctccagggggatttgatttggtggatgatatatttgatca

gtatatgtctggacaaactgttgcctcttcctcacagattcgcactgagttagatttgtatttggaagag

aaaccgttgcctaggacacaagattttgatattatcaattggtggaagtttgggggcattaggtacccaa

ctttgagacagattgcccgggacattttggcaatccctattacaacagtagcatctgaatcagcatttag

tactggtgggagggtgataactccaaatcgtaatcagcttaaacctgatcttgtggaggcacttatgtgt

gtgcaagcatggggtcgtgctgatatgttaggtaatttcatttttgcttttgtatcgttcaagtttaatt

tgtcctttcatgtttcttccatttcatctaattgcaaatcacattatatgttgatagctgaaattgctaa

caagaccaatgcattgaacacggtacttgacgatgaatcagaatcggtatgtcctacactcccactttat

tgtttttattactataacatacaatgcttagttgcttcctctaattttgatttcaggaagcatctactgt

cactgaagcttgaggtggagatttcataagtgaaggatgggctagaaagcctgcaattgcaaactatcta

cctatatattttattatgttggcaatgtaatagtatattaagacttctatatttctgtgagactgaattt

gcttgctgagttgctgtgtgtgatgtgttgaacaattcaactgttgaactttgaaggcttgattcgcttg

aacagttgaactcatgtgtgttagtgtgtaactgtgtgaaatgatggatatcatcaatgtgttcttgctg

aatttttatgatgaaattgaagtattcatgctatttttgatggtgtgttaattatgtacaatatttcttt

ttgatgaaatgatgattgtgttgtattttttgagctgattggatggagatgctacccaatgcggggcccc

ggtcgggttgcggggccccgcaggggtcgggggcgggggatgttttcaacccgcatcactggtcggggcc

gggggcggggggtgaagtcgggggtcggggtcgggggcgttccagcccaacccggccccgccccgccccg

ttgccatcccta

>Os_hAT-15 Oryza sativa

caggggcggatttactgtgggggcgggggggtctcgagaccccactaccgctcccgggacagtggagacc

cccctaagaccccactaaaattttttatgggatgtgggggctggaggaagagaaattcgtgatttgggcc

aaaaatataaaggaaagacagcccacgaccacccctgaggtcattatttccattttggcccaggcgccca

gcccattggaagaaattttggcccactcggccactaatccagtctccaaccgggcaaccgtccattccaa

tccttgcatccccaaatccccaatcctccctgctccctgcgaccgtgcgtagtgggcggcgctgggcggc

gcggcggcgcgcggagggcgacggctgacgggggcgacgggaggcggccggcgagtggaggcgagtggat

ccggcgagtggagacggcgcggcgacaccggcgtgccgcaggcggcggcgcggcgccgcggcggggtcgg

gacaggaggcgggcgcggccgcgcgggccagccggcgggaaagcgccgccgcgccgtcggggaggcggcg

cgccggcgactcgactgccagcgcccagcgaggccgcaaggcgggcgggcagggcggacaggaggcgggc

gcggccgcgcgggccagccggcgggaaagcgccgccgcgccgtaggggaggcggcgcgccggcgactcga

ctgccagctgcccagctcccagcgaggccgcaaggcgggcgggcaggcggcaggccgcaagcggccagcg

gcgccggcaacgccatgggcagttgggcacagccacagcacaggggcagccgggcaggccactgctagtc

tgctagactgctactttgcattggccattgggtaaactactaacatctgattctctgaatagaaattgtg

tcaagagttcaatttcagattttcagtgacttcaatttcagtgagttagtgattctctgacagaaactta

taattgtttcctatacctttttactttttagattgtcaacatgtcaaaaagaacactactcacgtattat

tctagttcaagcaacacagatccttcacctagcaccgagaatttaagtcagccgaagaggcctagggcag

agtttagtcaatcagatataattggtgatccagggcttcgcaaaccaattgaagcttatccacctgaaat

tagagatcaagtaaagagggcatatgcattgagtggcccaactcaacctaatattactatatttcctcgt

aaatggcaaggtggtgagtggagatcctttcaaaaaacttggttcaatgagtttgattggctagagtata

gtgtttcaaaggatgcagcttattgcttgtattgttacattttctttgaaccgggaaaacctgaaaagtt

tgggagtgctgtctttgctaaggaaggttatgtcaattggaagaaaggtaaagataggcttaccgtacat

agtaattgcaagactcacaatgatgctaggaataaatgtgaagactttatgaaccaaagaacaagtgtat

ctaaaaaaattgagattgttagcaaggaggaagaaattagatacaagattcggttgacatcttctttaga

tgttgtaaggtttctcattgaacaaggtgatgcttttcgtggacatgatgagtctgatacttcccttagc

aagggtaaatttaaagagatggttgattggtacaaagacaaggttcctgaagtgaaggatgcatatgaaa

agggccttaaaaattgccaaatggtatcacatcatattcagaaggatcttactaaagcttgtgcagaaaa

agtgacggcagttattatggatgagattggaaatagaaatttctctgtacttattgatgagtctcgagat

gtatcaataaaggagcaaatgggtgtcatcttgaggttagtagttcacatatgtagttttatatgtttga

tttttcagattctattttgagtactaacttagaaataattattgttgtgcaggtttgtgaatgatgaagg

aaaggtgatggagaggtttcttggtcttcaacatattgagagatgtacggctattgctttgaaggaagct

ttatttggtatgatctctagtcataagttaaccatctctaagattcggggccaaggttatgacggagctt

ctaacatgagaggggaatttaatggtgtgcaaaaattgattcgggatgaaaatccttatgctttttatgt

ccattgttttgcccatcagctacaactagtagttgttgctgtttcaacttctactccagccattgcagat

ttttttaactatgtgcctttaatagtcaacactgtgggtgcatcttgtatgcgaaaggatgccttgcttg

caaagcatcacgatgtattgttagaaaaggtggagaatggtgagattaccactggaagaggcctgaacca

agaaagtagcctagctaggcctggagatactagatggggttcacatcttaaaactttgcttcgtattcta

gtaatgtgggaggctatcatagatgtgctcgagattgttaagaaagattcaaccaaaccaacatttaatg

gtggagcatttggtttgatgggtaaaatgcaaagctttgattttgtattcatcatgcatttgatgataga

catgttgagcattacagatgacttgtcacgtgctttgcaaagaaaggaccaagatattgttgaggcaatg

agtttgctcatagatgtgaaagagctcttgcaggacatgagggagaatggatgggagccactattgaata

gggtgatttcattctgtaataaacatgagattaaagtgccaaaaatggacaaggaagttaatgaaagagg

gacatctacacatcgaaggcacaaggtaacaaataagcattactatcatgttgagatctatcttgcagcc

attgatgccattttggttgaaatgaaccatcgctttagtgaagttagttcagagttattagtatgcatgt

catctcttaatccacggaattccttctccaattttgatgtggataagcttgtgaggcttgctgaaattta

tgctgaagattttttggttggtgatcttatgctactacgaactcaacttggaaattttattagcaatgtt

aggagaagtaaagaatttcttggatgtaaagatcttgcaaaggttgctgaattaatggttcagaccggaa

agaatagaacttatcacttggtgtataggctcattgagttatcattgatacttccggtggcaacggcttc

agttgagagggtcttctcagctatgtctcttataaagacagatttgcgaaacaaaatgggggatgaatgg

ctcaatgacttgatgatttgctacactgagaagcagatttttagaagtattagtgatgaaaaaatcatcc

aacactttgaagagatgaaaaagcgtcgaatgctagtgcctcaacagaaattagtggtatgctccatgcc

tccattctctccctaagtctctaaattatttgaattcaaaataagtcattttcgattaatcgttcatttg

ccttttttttctttgtacagattactaatgaagaataataagtgactggcagtgaaacatcatctattgg

atattggtttggtatggacaaaattctctacttatcattaatttgacgtgtctactttgaactatacatg

tagccaatttatatgtgagatgtttcgatttatgctggcttagttagttcagcccccactactttttttt

cctggatccgccactg

>Os_hAT-16 Oryza sativa

tagggctgtcaaaaaagctcggagctcgcgagcagctcgagctcggctcgtcgcaagctcggctcgagtt

cgactcggcaaggattcgagccgagctcgagcttgtctcgaagctcgcgagctctaccgagccgagctcg

agctattcggcagaagcttgaaattttcactcccgctacccatcccaatttctcccgcactccaattcca

aaatcctaccaccaacagcagtccatcacctaaccatttatttcccttatatccccatcccgtatccaaa

ccctagctcatttgctctcttcctcgaaacatttctcttctagatctacaatcctaggctgctctagtgc

tccactgaccactgctgtggatctagccaagcaccgtccatccttcgcctctcctgtccaggaagaaccc

catggaggacagcagcagcccaccgcctccgccgtgagtgacgaaccgacgactcggatctgagtaagtt

tcctcttctttctttgttttacatttttatttcttttgcacccacatgtatctagggccctacatgtatt

aactgtgtagtgtttagttgcttgatggatgttattgatttacattgaattttatggactaggtggagac

atggagtagtacttatcttgctgtgctcctcttctgaagaaagactgaaggttgcagaacaccgtgtgtt

tgccatttgccaaggtaaattgatggcaatgcttttcttgttgtacttgtttgattccttcccttgtgca

gaatatatattgatagcagttggtatttttaatttttgtagatgtctgtaaacctgcaaagacagctatc

tatagagatcgagtcaccgagcaatagggatggcatagcagcaacagaagcacaaccgaatcagcagcta

gtaccaggtgaagtaaggggagaagagggccaagagggaaacccaactaaagccaccaggaaaaagaaat

ctccattgtgggatttctttgaagaatgcactgtgccatcaaagaagaagaaaggagaaatggaaaataa

ggtcaaatgtaaggcatgtgaaactctgctcaccaagaatagtagtggcactacaacacattggcggagg

catttagagcagtgtgattatcacaagcttcaacaaaaatccataaaacaacagaatatcaatttcccta

gccttgatgaaggtgatgttgatcttgatgctccttgtgtttctgtccctggttattatgatcctacaaa

gatacgtgagcttatttgcaaaatgatcattgtccatgagttgccattttgccttgtggagtatacatgg

tttaatgttttgctaaagaggctaaacccttcttacaaaaaggtctctagaaataccattagaagtgatt

gtatgaggctttatgaatctgaaaaagaaaaactgaagaggactttcaaagatgttagaaagataagcct

cacatgtgatctttggacctctaaccaaacaatttgctacatgagccttgttgcccactacatagatgct

gattggtcaatgcattgtcgtgttattaactttcttgagttggagcctccacatactggtgtagttattg

ctaatgccatttctgattgtcttgcttcatggagaattgaggacaagatagctagcatcacatttgacaa

tgcctcaagtaatgacagtgctgctaacctgttgcttgccaagtttacaaaacgtggttcactttggttt

tatggaaaattccttcatatccgctgttgtgctcacattctgaacttaattgtccaagatggtctggctg

tgattaagcatataattgacaaagttagggacactatcaagtacatcaagaaatcaaacaatcgagcata

caagttcagtgctgatattgattctctcaaccttaaaagtgacatgggtttggctattgattcctgcaca

aggtggggctcaaccttcaaaatgctgcaatctgcatttttttataggtctgccttggatgtatatgctg

caggagatgcaaactataggtggctgccaacaccagaggaatggaatctatattgtgaagtcaatgatgc

cctttctgtcattcatgcagccactgaggagttctcaggttcaacctacccaacatctaaccttttttat

tctcacattgtcgatattaagagagtactaggtcagttgttgaagtccaaagacccaattcttaaakaaa

tggcaaatgcaatgcttgaaaaatttgagaagtactggggcccagaatgcaatactttgtttgcagttgc

tttggttctagacccaaggtttaagatgggtatgattaactacacattccctgctttgtatgaagaaact

gtacttcctaagaaacttgcaaatgtsgaatccacattgaaatctttgcatgcatcatatgaaagtgaac

tccagagcacatcaaaggaaaatgatgccaccactcaatctactagtacatctctaggcactactagtag

tcatttctctgctgctagccaatttcatgagtatatgaaatcaaagaatgctgctagtcttcctaagtca

gatttaaagaggtacttagatgatcctgttgaggatattcctgcaaaaagtttcaatctacttcagtggt

ggaggatgaatgaattaaagtatccaattgtggcaaagttggccaaggatattcttactattcccatcac

ctcagtctcatctgaatcagctttcagcacagggggtcgagtgattagtgattatcgcagctctttactc

ccatcaacagtacaagctttggtgtgtacatctagttggattagaggaggccaccacaaaagcacagatt

tagtaagttcttatattttcatgtttctaaccaattaatagagatacacaatctaataatctgtgtttac

ttgtttgttgcatactaggaggatgatactaattctattatccttgtcccaactcaagcttcaactgcaa

attcggttgtgggcagcaattagactcgtttgtcaggtatatgtttgaggttatataaattatctattgc

taccatatcaattcctcatcactgatgaaacattacctataatctttgcacatggtttcagatttaactt

tacgaaacattaatgcaggcatatagcatagcatcaacaattagtttgaagaaagtgcttggaggtttca

cattggcgtactgccattgccaatagtggagtaaatattttgtgacagttgaaatttgaagtacgctgtg

ttagtagtataagtaatatttagatattagagcttttagatggaacatatgttgggaacttccggtgggc

tacttttgatgttaattgatgctctatgttctgtcaagtcattgacacatgatattgtaatgtttcttat

ggtgttatacacaccggtctattataaagtagtaatcctattatgacttatgcactactatgaatttgta

atctttctagtatatacctgtcgagaagctattatattctattatgaatttgtaatcctactttggcttg

ttaagaagcttttattgggagataagttcagattgctgaaggaaacagtcttaacgagcttctacgaatc

gagctcgagtcgagccgagccttgatcgagctcgaaccgagcctaccttcaagctcgaaggtaatgctgg

gctcggctcgagccgctcgagcctgtaccgagctcgagcttaggtaggtaagctcgctcgagctcggctc

gttgacagcccta

>Os_hAT-1 Oryza sativa

tagagctgggacttgggccgtgctttctcgggccggcccaaggcacgatggcccagcacgagggaaagca

cggcccagcacgacacggctatgggccgggctggcacggcccgtagttgggccgtgcctgggccgtaagt

tgaggcacgctgggcggcacggcaggccattgggccatgcctgggctagcccagcacgaaaaaggcccga

aggcccgaaggccggcccagcacgaaaaaggcccgaaggctggcccgagctctagcctagccgagcgcgc

gggagtcaggagtgcgtgcaattcgggccggccgggaggcgatcaggaggcaggagcgccgagccgccga

gtgcgtgcgtgcgtggtgagacttcagcgacgcgttcagaatgggcgtaaccatgagacgtggtcagtcg

gcggccgtgctaaatactgatcagaaacataggagccgtgctttcagtctttcagaattcagagagccgt

gcatgcatggaaagcatgatttttttggctaattaattgtgggatcacttaccattggcaattagtatcg

tgtatatccactgtctagtcaattaactttttgttcatgtttttgaactttttaaatggctaatgacccg

cgtccttgttcactgcaacggtgcatgtatggaattatggaaagcatgttcattgcgtggaagttcagag

ttccgacgcatcgagcggtcgaggcgtcgactgatgactgatcattggtcgatcagtatttagcgcggag

gagtgttcagcacgtgtcatggcacggcacggccattggtcgatcggtcatcagtcgaggcgtcgagccg

tcgaggagtcgttcagagttcagacgcgtcgccggccggtaaccgaggaggcaagggagaggagaagagc

ctggcgcccggccctggcataccctatataaaccatacccattttttttgatttcaacacacttcttctt

ctttcaacacatctccccaccgtatctatttttcatagaaaatatgcggaggtccaaaaagcgtcaacac

gagtcgacaaccggtccatcccagaggtcggtcgatgcccggcagctatccccgggcatggacgactacg

ggatggatgatcgccacatggatggtctggagggcgacgacgatgatcagccatctcctggcgaggccaa

cttctccgtcgacgccgacgccacgacgttgcccacaccaaattccgataaccagccatctgccagaaga

gcggagtgttggcggcacttcgaagtattcaccgagatggttgacggtaaaagtatacctcgtgctcaat

gcaaatattgcgacaaaattttgtctgcagctacttcttcagggacaggccatctcaaccggcattactt

ggcgcatctcaagaacaaggcgccggcaggagcaagacagacacaattgtccttcggccctgacggttca

gtaagtacatggacctatgatccaaaaatagctagagaagagattgctaagtttattgttgctgaggatc

taccaattagaatgggggaaagcaaacattttgagagaatgatccaaaaagctttttgcccacagtacaa

aaaggtttctaggaaaaccaccaagaacgatattacagctatatatcgttctaagttgagtgtgttgaag

caaactttcagtactactagtttttcatttgctgtgacatcggacatctggacatcccaacaccaaagaa

catcttaccttagtgttgttcttcattatcttgacaacaatcggtcattaaataagagagtcatagggtt

caaactcatgacgtcacacacgggagatgccatagccacgacaatcctagaggtattgagagaattcaat

ttacaatctagggttgtttccattactcttgacaatgcatcagcaaacacaacggccatgtcgattttgg

agccggatctacgaagttatgttggaggcttcgttattcatcagagatgcatttgtcacatcatcaatct

catcgttcaaccaggtactaatgttattaataatttattgattttagtgtatgcggcatattgcttattt

attttaatgtatgcgacctatttgttttgcaggcatgattgttctggataaattgttaaacaaaattcgc

cgttctgttcgtatcataggaggtaacacagtggtcaaagcgaggttccaggactactgtaaagcgaaga

agaaacctggacggatgtttggcattgatgtcaaacataggtggaacacaacatacctgttgctgcgaca

gttaaaagggtatgaggagttaatttctgttttcatcaattcgatgcatgtcagaatgaaggataccgat

gacgacggcgatggtgagattttgattctcaccgatcaggactgggagatcgctacacgtgtgcgtaaat

ttctgaagcccttttataatgctacagttcaattatcaggaatttactatcctacatcttgtttggtgct

tgagtggatttggaagctggcacttgtttttgatgaaaaccggtctgatcgtattctgtcttccattgtt

aaacccatggaagaaaaatttctcaaatacttcaccgctatacctcacctttattgctttgctttgatac

ttgatcctcgcaaaaagttggaaatcgccaaggttgccatgaactcaataggtgatgcagtggggcttga

ttattctgaagcttttcaacatgtcaatgatgagctctatagggttttccgtctgtaccgaaccaagctt

ggtggtactccccgagttcctgagcagacctcacaaaagaaagcaagcaagtcaagtgcagtaaatttgt

ggaagcagtatataggcaacgaccaagcctctccatcctcagagaacaaatcaacatggaacccggattc

agagcttaaccactacctcgtcaccaatcatactgagcatgatccgacgcttgatggcgacgatgtcgat

ctccttgggtggtggaaagagaaagagcgaactcttcctgtgttagctcactttgctcgagatattcttc

tggttccggcctcctccgtctcttccgagcaagcattcagcgttactggaagaattattgaagaacgaag

gtcatgcctgacacccgagacggttgagtcaatmttttgtcttaaagactggatggaagcagatgaacga

acgcaacaccgtctasatgatcaagagcttgccgatgcagtggaagacgcattggcggaaatttgcctta

ccaaagatgatggtggtgttgatccggagtaattgatgctataatgtaaaatagttgttgtactcttttt

tccttacgggggaaccctactctagggcgtaaggtttttaacgaggcaacaactatatattcaaattatt

aatatatatattttcccccaattttattgtctcaagttatttatgatattatgtcaattctaattgtgtt

cattaagccatatacattattatctcattgttttttcattttccctctcttattttttccctcccttatt

tcagtttccctctcattttccctatttttcccctttactatttcacttatcgagcagcatcgggcagagc

acaggtcagaggcaaagaccctaggcgccggggcgtcggcagccggccctcggcggcggcggctctcggc

gtcggcggccctcggcgcgggccgcgcggcgcggcggcggcggctatgcgcggctgcgcggcgtcggtcc

tcggcggcggcggcggctctcggcgtggagccctcggcgcgggccgcgcggcgcgcagctatgcggggcc

gcgcggcgkcggtcctcggcgttgcggcggctctcggcgtcggcggctggcgcgggcagcgccgcaggga

gcaggcggcggcggcggcggccggcgcggccgcgcgggccccggcgaaaaccctaggcacggcacggctc

ctcaggtaatcctgggccgtgcttgggccggcacggcccgaagaggcccgacatgtctttgggccgtgct

tgggccttgtcttctccacctcggcctggcacggcccggcacggcccgttctagcacgaagcacgacggg

ccgggccgtgctagcccggcacggcccaagtcccagctcta

>Os_hAT-2 Oryza sativa

tatagttggccatatggcccgaggcccgatggcccggcccacggcacgggaatttggcccggcccaagca

cggcacggcccggtagcagtcgggcccgtgccggcccggcacgactattgggccgtgcctgggcctatgg

ctgggcacggtgggccggcccggcacggcacggaccgaggtcggcacgatgggccggcccggcacggcac

ggcccaacggtcggcacgaccacggcccgtaccaacggtaggcacgagcacggcccagcccaacagacag

ccaggccgcgccgcgtgcgcccgcatatataagccagcgaggccgccgcccctcccctccccagtcccct

ctcgcctctcgctctccgcctctccgcctctccgcctaaccctaaccctagccgccaccgctcaaccgcc

tccgccgatccgcctccgacgctccaccgcccaccagatctcctcctccaccgtccgccgctcactcgct

cagggcctcaggcgcgcaccggtgagactcaccgagtcgcctcccttccactcttccagatccagccttc

ctcttcgtctccgatcgatgcctcgcctgactcgcatgaggggcgcatgaggatgggggccaccggccac

gcatggtcgcctgagtcatcgtccttccactcttccagatccagccttcctcttcgtctctttccctttt

gttcttcttcgccagatcccttggtcttctcgtagatccgtgagcctatcgtcttcaacggcaactccgg

tgctccggctgcgcaggtacaagcatggaacccatcgtttttcttttttttttctccatttccgacccta

accttgagtgttgttggctgcttgcaggtcgcaagcccgttgcttagcggcagccgccgttgagggacca

gagtgcctgcgatggacgacgatcctacctctgtcaactacgagttgaggactatggggatgcgcggtga

tgacgatgatgatgtggaggaggatcgtgtggaggtgttcggcaatacctccgacatcccgatccatgtg

aatgtcgatgacgacgacccgccggtcgacgacagtggcaatgggacgccgactggctctagtgcgactt

gtaccaacaagaagaccaaaacctccaaggtatgggatgactttgaggaactttatgaaacgaccaatgg

taatagggttcgagtctctgctaaatgcaattactgtcataaaactttgagtgctcgttcttctgctggc

actggacatttgcttaggcatattaagtcatgcaaacctagaaagcttgggtctaatgctttgcctcagt

ccatgcttagatttagtgcagatggttctgttattccatgggaatatagtcctgaagttgctaggtttga

attgtgtagattgattgctagagaggatcttccaatcagttttggtcaatctcctgcttttgtgaactat

attaaggctgctcataaccctagatttgttcctgtctctagacaaactactaccagagatttttataagt

tgtttaaggatcgtcgttctgttattattgatcgtctcaattctgctagctctattgctttgacatctga

tatatggtctggtcatgctaaggaagattatttgagtgttgtagctcactttgttaattctgattggcaa

ttagaaaagagggtcttgggtcttaggcttattgatgagtcacatactggtgctaatattgctgagcgtg

tgattgctgttgctgaagaatatggtattactgataaggttttctctattacattagacaatgcttctgc

taattctaaagctatggacacccttactcctgctctgtctggttatattggtgatttatttttgcatcaa

cgctgtgcatgtcatatcatcaatttgattgttaaggctggacttgacaagttcaagcccatgctaaatg

acattagagctgccatttcattcttgaatgcatctaatcagcgcattgctacatataaaaatgtttgcat

tgctgcgggttatcgtcctcgtatgtttggtttggacatggatgttagatggaattcaacttatctaatg

cttaagcatctcataccacatagggaaccattcacagtatttattagtacacaacatccttttgttaatg

accatccattactcacagacttacattgggcatgtgctgaatctgttttgtgtttccttgaacaatttta

tgattcaactgttgttttgtctggtgtttattatccaacatctccattaatcatgcatcacattttagag

attgctggacatctaaacacatatggcaatgttcaaaaccttgcaaatgttgttggtcccatgaaaacta

agtttatgaactactggtctaaaattccaattctttattcatttgcatttatcttggaccctagggccaa

gattagggggtttagtaaagtgcttcagattatggctcaactcattggtgatgattactctgcttattta

acaactgttagagcttcattgtctgatacttttgctaagtatgaaagaaagtttggttctgttagattgc

atagttccactattccaggcccttccactggtaagaaaaggactgcatgggggaagatttttggttctgt

tgttgctgctgggcttggtgctggtaatgcaggtgccagtccaggtgctggtaatgctggtgccagtcca

ggtgctgggcttggtgctggtaatgctggtgccagtccaggtgctgggcttggtgctggttcattatcca

ggatgacatctgctactgctttgcttcaggcagcttcttcaactgctaatctcaattcttctgaactgtc

tgcatatcttgacagtgacactgtcaaccaatatgatgatgacttcaatatccttagttggtggcaacag

cacaaactaacttatcctgttctttcaatcttagctaaagatgttatgactgttcctgtttctactatat

cgtccgagtctacttttagcttaactggcaggatcatcgaggaccgtcggcggcgtctaaaccctaggct

ggtggaaattctggcagtgatcaaggactgggagctagcagatgcaaaaagtcagcatactactgagaat

gtggagcttcaaaatgcttatgagaatatgtatcttgatgatgaaattgatgtaaacccttgaactggat

gattgtaattgatatgaacattttagctatgggctgtactcttttcctttctagggttttctcacgaggt

gtgagtttttacctagaaaggtttttaatgaggcagccattgcacaatcacagcttatataaaccccttt

tatattctaactttctgaggtttgttttgatttgtgatttgtgattctgagatggacttgatggaaaata

gtagatgacttgatgtatgcatttgatgaattgatttgaactgattgtaaatctgtgagactgtgacttg

atgaattggtgacttgaagaattgttcatttgtttgtaaatctgtgcttgtaaatctgtgacttgatgta

tgcatgatttgataaactggtgacttgatgaattgtttgtgattctttggaggtgagtttacttatttga

tgaattacatgtgaatttcatgaatttgatgtgttatgtcaatttgttgtgttgaattgaacctttgaag

gggtttttttgccagggcgtgcctgggccggcacgggcacgatgggctagccgggccgtcgggccagccc

ggcacggcccgaggctacttgggccgtgcctgggccgtggttgtagcccgtgggctggcacggcacggca

cggcttgttcgtcgggccgtgccggcccgactgacctcggcccaggcccggtcgtgcctgggccgtgccg

tgccgggcggcccatatggccaactata

>Os_hAT-3 Oryza sativa

caaagttttaaatctccggctatagctgccgctatagccggagatagctgctgggaacggacggagctaa

gagatgcagtttttagcgctaagaatagcgggacgctaatagcgccatttagccggcaatagccgctaaa

ctggccgtaatttagcgtagctaaattgtaaaacgcggccggtctggcccaagaggaaaccggcccaact

agaccagagtcgagaggtgggtaaagtgctgcaaaccctagctcgtaagctcccagcagcacactatcca

ttccaacttcgaagcgctcactctccagctgcaagaaaagtggaggcggccggcggcggcggcgcagagg

cgtccggcggcggcggtggcttcaagcggatcgaccaacgcaggcggctcgtggaggcgaccggcgccgg

ctgctgcttcgagcgggcgtccaaccagcggcggcggctttgaaggcagctgagcagcggcgcaagtccg

acgaggaggtccgtcagtgcaacgtctccgaagcactgaagcagcagcaccaaaggtgacggcatggcac

aacaatccggtcagttctcacatcctcctctcttttttgcttgtatgttgcagatcttacaatatgtgaa

caagttgtccatatttgttacctctaatttcagatacttgccttatgatgtagatcttactatatgtgaa

ttattttcttactccctgagatctctcgtttgcttgtttcgcctgtttaggtggtaatgataatacgtgc

tatatcagtttgcattattttgatgcttaggcagctttgacgctagcattaatgtgggctaagtttgctt

tttgtataggtaatccgtccagtgctgcctctgtctcagtagaagctggaccaaaacaagctggtatcaa

ctcagatgatcctgcatgggctcactgtttctgcccagacataaccaagaaacatcacctacggtgcaag

tattgtgacaaggtctgcactgctggaattacaagaattaagtaccatcttgctggaattaaaggtttca

atactactaagtgtcagaaagttccaagtccagtgcagcaagagatgtttgatttgcttaccaagaagac

tagtgaaaaggaacagaaaaacaaagaaaaggaagtggccagagctgaagttgacatagaaaattcagat

tgtgaaagtggcagtgaaggttcagatcatggcaataatgttcttgtggtgaagccaaaggagacaacag

gatctagtagctctagatctgtagcaggtggtcatactattgacaagtactacaagcctccttctataga

agaatctgccagtatgacacaaagagtaattaagctaagcaataaggttcaaacagcattgacaactcag

aaaagagaagagaggaggaatagaacttgtgagtacatatgtcagtggttctatgaagctagtattccac

acaacacagtaacccttcctagctttgctcatatgttagaggccattggacaatttggtagaagtctgaa

agggcctagtccctatgagatgagtggatcgttcttacagaaaaggaaggaaaaggtgatggatggattc

aaggagcacaaggaatcatgggagctcacaggttgttctatcatgacagatgcatggacagataggaagg

gtaggggagtgatgaatttagttgtgcatagtgctcatggggtactcttcttagattcagtggaatgctc

aggtgacaggaaagatggcaaatatatctttgaacttgtggacaggtacatagaagagataggggaacaa

catgttgtccaagtggtgactgataatgctagcgtcaacacaactgcagcaagtctattgacagcaaaaa

gaccatcaatattttggaatggatgtgctgctcattgcttggatctcatgctcgaggatattgggaagct

tggaccagttgaggaaaccattgctaatgcaagacaagtgactgttttcttgtatgctcatactagggtg

ttggatttgatgagaaagtttcttaacagagacttggttcgctctggggttacacgatttgccacagctt

atttgaatctaaaaagcttgttagacaacaaaaaaagagttagtaagactatttaaatcagatgagatgg

agcaattgggttacttgaagcaggccaaggggaagaaagccagcaaagtgatcagatctgaaaccttttg

gaaaaatgttgacattgcagttaattactttgagccattggctaacgtgttgagaagaatggacagcgat

gtaccatcaatgggattcttccatggtttaatgcttgaggcgaagaaagaaatttctcagagattcgata

atgataagagccgcttcatagaagtttgggatatcattgataaaagatgggacaacaagctcaagactcc

actacacctggctgggtactatttgaacccctactactactacccaaataagcaagagatcgagagtgat

ggatcatttagagcaggtgtgatttcctgtattgacaagttggttgatgatgaagatatccaagacaaaa

taattgaagaactcaacttgtatcaagatcagcatgggagttttggacacgaaattgccgtaaggcagcg

aaagaacaaaaatttcaatccaggttcagatctcaacatgtgaacttaattttattgctgaatatcattt

ttgttgtaatttgtgtaatgtttatgcagcaaaatggtggctgaaccatggcacaagcacaccaaatctt

aggaagttggctgcaaggattttgagtttgacctgcagctcctcagcttgtgagaggaactggtcagttt

ttgaacaggtaaaaagaaatttgtgacctgtagctttatgtgcatgctgtctattttgaattataatgct

tatttatggaatttagatctgacctgcagctttgtgttcactacttggcttataggttcatacaaagaag

cgcaacaggctacttcatgaaaggatgcgagatctggtgtttgttaaatttaactccaagttaagaaata

agagagagaacaagggtagagatcctatagagaaggaagtggatgatgttgtggcagatggtgacaatga

attcattactggtgttgtgccttcatcaagtgaaatggatcaacaatgtgcaaaagagtcacagcagcac

acaacaccacaagcaccagcaccagctaaaaggaaaaggcctgtgcacgctaagaagaggaaagtcagaa

gcctacagtctttgatgcgcaatgccccagtgcatcctgaagctccatcatccgactcagaagattgtga

tgatggtattccaatgcagacttctgattctgataagtcaccctctccctctccctatgtctctgagacc

gatgattgatgactagaagaaatgaagattgcaatattgcctcatattagtaatttagtatccactatcc

agaattcttgatgacctttgtgttgtactgttgtctttattccagaatgctgtacttttgtgaaactgat

gtttcagtattctgttaaccacttagcctgtgatgtaatgtactcctatgaacctgtaatgtactcattt

gaactggttatgtatcaattggtaatggttatgaagttatgccaagatcatgtcttttatttatttattt

atttgcaaaaacagctaaatggcttagctgcagctatctccagctatagctgtgcttagctgttgagaag

gtcagcagctaagaggcttagccggagatttaaaactttg

>Os_hAT-4 Oryza sativa

cagggttcactttctcgccggtaaccgcgcggtaaccgcggttaccgggcttaccgtggggggtcggtaa

cgaaaaccggcggtagggtttgggcaaattttgctcaaattcaaaaatttgaaaaataatcgaaaaaaat

aaaaaaaatcatggatgtaaagcttgttttgtatgttgttatggtgaaaaaatttcatcaaaaagatgca

ggtaaattcaaatttgagcgcaaaacctattgtgaattataaaaaagaaaaaagattaaatggaatgggc

caagtatgcactgttcacgaggcccaataaggcccacaatgggaggaaaatatctttggaagcctctgct

tcgtttctctggcgaatcctaccgtttttcccctctctcgcgtagctgcgctcaggaccgaaggggcgac

ggcgtgcgcccgcgccagatccgccgcctccgcgccgccggcgaggccggttcccgcgcggtaagcggcg

gtaaccgcgcggtacgcgacggtaccgcctgggtcgacgggcaagaggtaatccctccgttttgaaggtt

ttcgcgcggttaggagcggtaaccgtgcggttttgtacggttaccgtcggcttcgtaatctatgagacga

tatacgtatgtgcggtaatcgtgtgaaaaccgtggtaaccgtccaccctaccgtggttaccgtggcttga

accgtggtatggtattttttactgtgatatgcatggacatatttgggatttaggtcaatagaacatattt

ttaccaatatgcatgtgaaatacattgggaatatgagtttatttcggcatttggttgcccgtgcttggct

aaaaccatgcatgtgaagtatatttgattgctttggtctatgtacatataggttaaattaaatacgtcat

agtatggaagaatttggatgcatgcttaccagtgtacatataattacgtcgtattattgcataatgggag

gtatgtttataattagcagtgataatttctcatgcatttgatacagggtggagatgtcagatagtaggga

ccctgtgtgggagcacggggagaatatcccacctggatggcgctgtaagtattgccatacaaagaggggc

ggtggtggggcaacaagactaaagcaacacttggctgcaagagggaagggggttacatattgcaattcag

tgcctccggatgtccgtgagttcttctgccgtgagttggacaggataaaagatgcaggtgaccagcgtaa

gagtgatagcggaagaagggttgaagcagcaagggtcaactattatgacctaacaggtgatgccgacgag

gaggaacaaatggaagcagccattgcagcctcacgacaagacgaaaactttaggagggatgttgaggagc

gtggtggcacttatgagcatggcggtgggagtggatccgctcagccggaggcacggaaaggtagaagtaa

cccaattaccaatatgctacgaagggctacgtctcatagggagtcacctgctgtgagagattacaaccta

gcatctgccaaggcccctgtgcagccacgcattgacacaggattcttcacaaagaaggggaagcaagcta

ggcaagccattggtgagtcatgggcaaggttcttctttaccgccggcattcctggtagaaacgccgataa

tccatactttgtgagcgccgttcgggagacacaaaagtggggtacgtatgcatctattaaggaacttctt

tttttctaactcattgcgcactacttatttcatacgtcaattgtaggtgaaagtgtaccttctccaactg

gtaacgagatagatggaaaatatcttgattctacagagaaggatgtgaagaagcaatttgataggttcaa

gaaggattgggatgagtacggtgttactataatgtgtgattcttggacaggtccgacgagtatgtcggtc

atcaattttctgatatactgcaatggaataatgtttttccacaagtccattgatgcgaccgggcaaagcc

aggatgctaactttgtgctgaaggtatgtatggttcatgcaacaaattacttcatcacctgttatcgtca

gtgttaggggcatggactaatttttattatgttatctaccaatgcaggagataaggaaggtggtacgcga

aataggctcggagcatgttgttcaaattatcactgacaatggctctaactacaagaaggcgtgcagacta

ctacggcaagaatacaagacaattgtgtggcaaccttgtgtggcacacacagttaacttgatgcttaagg

aggttgggaaaatgccagaccacgaaatggtgattgagagtgctaggaaaatttgcagatggctatacaa

tcataacaagttgcatgctatgatggtcttagctataggtggtgaactggttaagtggaatgctacaagg

ttcggaacaaactacatgtttctccagagtttccttaggaagcgagatcttttcatgcaatggatggctt

cctctgtcttcatgcaaagcaaatttagtgggactttggaaggtagatatgcacatgcatgtctatctag

tctgtcttggtgggagaacttagaggcagtagtgaattctgtccaacctatgtactcattccttcggttt

gctgacgaggacaagaaccccaatttgagtgaggtacttttgagatatcagttgctcaaaatggagtacg

acagtctttttgcgaatcaaagggacaagtttgaagcatacatggagatcgtgaacagaaggatgcacga

cctaaccaatgagactctcatcaatgccggtaaatattggtcataacataatccacatggcatattatgt

aactcttacttaacctgtgccatgttttgtagctgccgcattgaaccctaggacgcactacgcgtattct

ccaagtgctactgtcttccaagacctccgacaggcattcgagtggatgacggatatcgatacggctgccg

ccgctttgctggaggttgagatgtaccgacgtaagacgggtgaatttgggagggcactagcaagaaggat

ggccatagatgggaaaacttcacctggtatgtttctaatcatggaactatgatatatctaattgatcttg

atgtattcagaattatgaaatgacatgttacttggcttttgcagcacaatggtggtctatgttcgcctca

gacacgccgaatttgaagaagcttgcgttgcgtttggttggtcaatgttgctcctccagtggatgtgaaa

ggaattggagcacattcgcattcgtacatacgaaggtccgtaatagacttacccacaagaagctcaacaa

gctagtgtacgtgaactacaaccttcgccttcgaattcagcaagcaaatgcccaaattagggtggaggac

gatgatccccttcaaagattagcggacctctcattctatgaaactaacaatcatatcagtgcctggatgg

acaatgctcgctctaatgcttgccccgaactagatgaagattcagctgagagtgacgctcccctgcctag

tcaacttgtgtctgacctagtcaacttggatgacctgcgaaggaccacgggagcttctagtattgctgag

tgggctgatacgaatgtaggtgacactcatattgggaaaaggaagactcgaaagccgccaaaagcacgtc

catctaaaaaggtaaagggcaagggtccacgatcgactagtgttgatagcgatgaagagacccaaggaag

cccagagtaccaagagtccaatgatagtagctcaagaactgaaacagatgacggcgacgacgatggtcaa

ggaggccaaggcactactaatgtgcctcctaggggtcacacccaacaatcagatcaccacagccccgttc

aattcactggtgtgatataattcagttgcaatttcaaccttcatcttccaattcagtactggataatttt

cttcaattttttgatttgcaggtgagggcgacttcactcatgctactcaggaccaggatcatggtgctcc

aagctctcaacgaactaccattgcaccgggagtccgacaacagcaacaattcagtgacatacaacaggat

agctctagctcattcagtgcctctagctttgaaagtggctatccaacgtatgtgtacaaccgtccccaag

cttctgattatcctgctactacatgggtgtatgaatggcaagagcctcagtggtatgctcaattgtacgc

tcagtggcaaacaacatcatcatggacaggtcaaagttgggaggaatacaaggcggggttgctacattca

catggcttgatgctcatgtccacagaggagtacaatatggcatataatcagtggaatgcgtagctatgca

tgtccttgtttatttcctttcattcgaactagaattgtaatcgtggacattatactgtggactctattgt

tattgtggactatattgttattctggactgtattgttattctggacctacatgtggacatcgttatgctt

tgaaatgtgagattgatgattgttattgttgttataatgttatgaattattgtattgatttgaactccac

aatatagagtaggtcatgccgaatttttctgttgtataaccacatcttaattaactaaaatactcacatt

ttagtttttttctattatttttttcatttttcttatttcagttccaacatatcgcggttaccgtcctctt

accgcggttaccgtaccaaaccgtgcggttaccgtgtccataccgtggattttgaattcaaaaatttgga

aacgaaatttgtgcggtttttcgcggtaaccgcacggttaccgcggtaaccaccataccgcggggggtcg

gtaacccccccccccccaaaacggtaaggtgaaccctg

>Os_hAT-5 Oryza sativa

taggggtgaaaacggagcggatagtttccgtccgctccggtcaaaaacggatacggatagtgttcggagc

ggatttttttcggatagttcgaatacggatacgaatacggatattttttctccggatacggatacgaata

tggtaacgcagtttccggcgaatacgaatacggagccgaatatccggagattaagatgcgcggatatccg

cgtaacgcttgatgtctctccagcctccagccccatcccttcaatttcagcacatagttttctctcggct

atctaaaccctagctgccagatgggcgaccggcggcggcggcggtgacgacggcagcggcaagccggcga

cagtggcggcggctgtcttagatctccttggctccttgctggagttgctgggcattggccttcaatcccc

ctagtgcttgtttgtgcttcctatctccctaaatcgtgggacagtgggaggctaggagccgtcaccacgc

cgccggcggcaaggcctcttgcatgcactaatctcttaggttagaatccattgatttgttcttctcctgt

aacctttgttttgtgcctttcatgagatcgataaactgatgtagatcttggatgtgcttttttttacttt

gtgtctagattggtgatggagtctggtagtgcaagtagtgcaagtgcagctgcaagtaccactgcatcat

ctccagtctttgcaactcccagcacctccaagccatgctacggcagctacgcaggacacttctcctactg

ctggtgccttggaacacaatgatgcaattgatttgacaatcaatgatgattctgaggttttacctcctcc

tggtaagaagcaaaagaagtgtagttctgaagtttggcagcactacactaaatataaggtaagcaagaaa

gggtcagatgacactgtgattgtagaggagtatgctaaatgcaacaagtgctcatataaacgtcggtgtg

agagtaattgtggcacttctgttttctggaatcacttgaataacaagcacaacatcaagtcaggtcaaca

acaactacagatgaaaaagagtgaagatggtactgaaggagctgttgagacatacaggtatgatgagacg

gttagcctgaaaaagttctatatggcaatcatcatgcatgagtatccctttaacattgttgagcatgatt

actttgtggattttattaagtctttacgccctacttttccaattaagagtcgcattactgtgagaaagga

tattctaaacatatatgaagaggaaaagaagaaattgtggcagcatttgcttacactctcatgccgtttt

agcacaaccatggacatgtggacatctaaccaaaataaaagttatatgtgcatcacagttcattggatag

atgacacttggaagatgcaaaagaggatcattaagtttatgcatgtagaaggacatcattcaggaagtaa

tatgtgcaaagagttttatgacagcatagttgattggaatcttgatagaaggcttgttggacttactttg

gacaatgcatcgtctaatgatgtttgtgttaagggtgttattttaaaactccgtaaaatctctcctttaa

tttgcgatggcatcttctttcatgtgagatgtttcaatcatatcttgaaccttgttgcacaagatggtct

gaaacaaatcacaggagctgtattaaagatcagaaatactatttcactgaattctaaagcactcgccact

gcagtttgaggcatctattcagaagtgtgccttggaagttagtttggataacacaaagggcctttccatg

gatactcctacaagatggaactccacatttctcatgttgaagaatgctatctactacagaaatgcttttg

ataggttgtttcttcggcatggtagaaagtatgcaaagtgtgctcccaccaaggtagactggtctatggc

aatagcactatgcaagtgcttgaagccatttcatgaagcaactgaactattctctggtactacatatcca

acagcaaatcattttttcggtaagttttgttccatcaagctttatattgctgattggtgtaacagtacag

atggaacaataaaaaccatggcaaacgccatgcaaactaaatatgataagtactgggagaagtcgaatat

ggctcttgctgttgcatgcttccttgatccacgttacaaaacaagttcaatagagtactatggtatgaag

atttatggtttagaagctgcagaaaaatttgatgagttcaatggggttatcaaaaaattgtttgatgttt

atgctttgactagtgcgtgtgcaacatcaaagaagaaaggtgctgaaatgcatgtgcatcaacttcaaat

tcaatctgatcctgttcataatactgatgagtttgatgatatctttaatgagaatgacagtagccatgat

catgagcaacattttcaaagatttttgcttgagagaagtcagccaatttgtagtgataaaaccgagttac

aaatttacatggaacaaccactgttactatggacttccaaagatccatttgatattttgtcttggtggaa

gctcaaacaagcagaatttcctatcctctgcaaacttgctcgtgattttcttatgtattcaagtgtcaac

tgttgcttctgagtcggcatttagtgctggtggccgtgttgttgatccatttcgcacccgtcttgatcca

gaggctgtgcaagctttagtttgcaccaaagattggattaaagcagctaacaatggtacactttgtgtct

gtgttcattgcttatttggccattccttcttgtatatatcaatatatgtatagctgtatccatgatctga

tctggcatcatcccgggcattcttaggttataagactcaggctatcatcaatgagttggacattgtgaca

aagcgtagagaggcatccctggcaaacttattgatagtggtgagctattttcttattttacctctatgaa

tatttttcttaatataacaacaatattattcgactcctgtaggaaaaagaggagaataatgatgacatgg

aacaggatgatgttctgatggatgaaatggatgaataatgatgttgcagttttcatgcatcggactgcaa

gtgtactgaaaattgctttgggctaattactattttgatcttatctgtgttacctatagaactcgaactc

aatatgtgttgaacttttcaacgtgtttcgatttgaacacgatacactttgtttaatctgtgatgcgtcc

tacaacgatgatatttatattaaattagataaatatatgtggtgcatttgtatctacgagcagtttgagt

ggtctctatattccgagaaatattcgttttcgtattcgtgtccgatcatattcgatccttattcgtatcc

gcgataattcgtattcgtttccgtatccgagttattcgtattcgtttccgtatccgcctaacaatatgaa

aacggatacggtaagagcattatccgtccgtattcgctccgttttcaccccta

>Os_hAT-6N1 Oryza sativa

taggggtgaaaacggggcgggtatttcccgcccgcccgcccgaccgtattatttcgggacaatttcgggt

aaaaaattcgggtacccggaagtttttacgggtagcgggttcgaatacgggtattttctatcggatatga

gatcgggttcgggaggacggtatccgacggatacggattatccggcaaaaatacgggtattacccggtta

aatatccggataatacccggataacatgcctagcccaataaagcactagtggccaaaaggcccactagta

caatgaccaccatcaaccagtaaaccatgcctaacaacccaacaaccaaaaatactaattactgttcagt

gttcactaatcccactaagcaatgacgccgagactacgagcgccaccggtgctgcggtgcagcacccgtc

gcaccgccacctgctgcatccgctgagccgcggccaccgccgctacatgcctttgctgccagagccgatc

ctcatctcttcaggaacaggaagtcagcagctcatcacaagtcaggccgagtttagttccaaactttttc

ttcaaactttcaatttttctatcacataaaaacttactaacacacacaaacttccaacttttccatcaca

tcgttccaatttcaaccaaacttccaatttttccagttaaaaatcaattgttcattgtcgtattgcccgt

acatcttaagccctaataatatagtcgcattatggttatatggggttggttgaatatatagctattgtct

atatagtatagatgatcgttgctttgacttgatatccgaaaagatattcataaccgatagtggtcgtatc

cgtcttggtacgtattcgtctcatatttgtgcccgatattatccgtgcttgtatccgtattcgatactat

ccgtatccaacccgattccgattataaaatgtgggttaggatatgggaagagtgagatccgaccgaaccc

gacccgatttcaccccta

>Os_hAT-6 Oryza sativa

taggactgaaaacggggcgggtatttcccgaccgcccgcccggccacattatttcgggtcaaattcgggt

cgggaattcgggtacccggacccttacacggatacagggttcgaatacgggtaattttttgcggatatga

gatcgggttcgggagagcggtatccgacggatacggattatccgggaaattatgcgggtattacccgata

atctatccgtatattacccggatagatggacagcagcccaacaggcaagcgcccaacaggccaacacaac

aggcaacagccagcccacatccaaaaggcccaccaaacaaagtgccaagcacaaaccctagccttcactt

cacagttcagatcagggcagaacgcaggcaggcaaacagccaggcagcaccgcgccgcacgccgcatgcg

tgcatccaggctccagcaccgccgccgtgccttcgcagttcgccgaccaccaagtggctacgcggccacg

cccaccgaccaccggccgctgtcgcacgtcgccgcgtgggtgtgcgacaagccgacgaccgtcagactgc

cgccgcgccgaccaccaccgcaaggccccgcctggccccttgcctcttttggctcctcaacgccgagagc

cgaaggacgtcgcgccacctcgtcatcttcttgggtagtcaagtcaacactcagcgactcatccagccat

ccaggttagccaaagtttcagtttttcattgccctttctcaacatcacgttagaacttggaaggattttt

ttttcttattttacttttgctgtttggctgtaggattaggatagaatggctagtggtgatgcaaacacta

gtatgtgtgcaagcacaggtgcatctccggctggtaccgtagctgttggtactggtcactcggcattggc

accaaatgcattggtactaaatgatgcaaccacagccactggaggtggaggtggaagttcagaacctgtt

gttattcccgatgaagaagatggagaattcacaggagaagttgaaccggtagcaaagaggcagaagaagt

gtacctcgaaggtttgggactatttcattaagtacactgacaaggccaagaacaaagttactggtgtgga

agagcaacaacgtaaagcaaaatgcaaaaaatgtggtagagtgtttggagcagagacagtaaatggcaca

aaacacttatggaatcatttgaatcgtattcactcattgaaacaaggccagcaagaacttcaggtgaagg

gtgaagttcagactttcaggtatgatcctgaagtaagcttggaaaagtggtatatagctgttattatgca

tgaatatccattttctatggttgatcatgtgtatttcaatgagttcatccactctcttcggcctagtttt

gaattcaagtgtcgcattaccactagtaaagctatattagagatatttgaagttagaaagagaattttgt

atgatgagctgaaatctgtttctagtcgaatttctacaaccatggatatgtggacatcaaatcagaacaa

agcttatatgtgcatcactgctcattggattgatgaaaattggctgatgcaaaagagaattcttaagttc

attcatatagatgggaaacatacaggaacaaggctagcaaatgcatttgtcaagggagtaatgtctatga

atattgagaaaaaattatttgctctcacactagacaatgcttcatcaaatgataaatgtgctagagaagt

tgtgatagagcttaacaagctgtttaacatcagtaaggtccctccattgatgtgtgatggtgctttcttt

catgtgagatgtttatgtcacatattaaatcttgtggctcaagatggattgaaaatcattgcacatacaa

ttcagaatattaggactactattggtattgtgaagaattctacgttgcaatgggaggaatttcaaaagtg

tgctgtagaatgtgacttgaacaacaattctgggctaccccttgatgtgcctacaaggtggaactctaca

tatgacatgttgaaacaagctatttactacaggggtgcttttgaaaggcttttattcttagatgaggata

gatatcaaaggtgtgcatcaagtgctgaagaatggggtatggcagaatcactatgcaattgtttggagaa

attcaatgatgccacattgttattttccggttgtctatatccaactgcaaacttgttttggtggaagttt

tgtgagattaagttagcacttcgagaatggtgtgcaagtgctgatgtctccattgcttctatggctgtgg

ccatgcaactcaaatatgacaaatattgggacaagtccaacctagctcttgctgtggcttgctttcttga

tcctagatacaagcaaaagctagtgattttttttctacagaagatttaccctgacaagtatgaagaggaa

tttaagcgtgtcttggctgctatcgataaatttttccgggcttacaagagttgtgttgcaaggtcttcga

aacctaccgctgcaggatcaagtgaaaattctcagccacatggtaatacttctttgggtcacaatgaaat

cgagaaattcttgtatgatgatgcagctgcaaataaagaagacgatataaatgaattggatgtctacatg

aaagagaagccaattcgttgggttgatccaacaggtgaaggtgtagaatttgacatactggcatggtgga

agaacaaccaaatgacctttcctatcctctcaacactcgcgcgtgatgtaatggctgttcaaatctctac

cgttgcttcagagtcagcgtttagtgctggtggtcgtgttgttggtccttttcgcagttctctacatcct

gagatgattgaggctctagtttgtaccaaggattggataagagcatctagaaaaggtaactgcttgttta

tcttcatttcgctatttatataatctgccatttctagactgtgtttatattttgtcaattttatttacag

gaccatttaaggacaagatagcaacaattcttgatgagctggatgtacagatagaggataaagatggtgc

taaaactgacaatgatgaggaggttaattacatatttatgtgttgcatttaaaaatatattttggacctg

caatctttaaatgaacaatctgttggttccactttggattttgagagagttaaggagcaccacattgtat

taactgaatgttatgttctttttagggtcaggcaacgaatagtgtttgaagctgagcatgttgctgattg

gagagctggagtttttggtgattgaatctgaacatgctggcctttgggatgactgtattggtaatatgtg

atcagtagtcatataagtgtatcttttggcttgattgtataccttatgtgaactactttggtactactta

gtagttagtactattacttatggagttatggtgatgctcttagtatttactagtatggtactacttatta

tggtgaactatagtgtacagcattgagtctgaagctatcaagctatgaatgtctacttttgctactactt

tggtacagttttgctgataggcattacgatgccataagcccctaatatattcataatagacatataggaa

atggctgaatggctattgtctatgtggtatatttggttgttgctttgacttgatatctgaataaatattc

gtaaccgatagagttcgtatccgtcttggtacgtattcgttccgtatttatgcccgatattatccgtgct

tctattcgtatccgataatatccgtgcccgacccgattccgattataaaatgtgggttaggatatgggaa

ggatgagatccgaccgaacccgacccgatttcaccccta

>Os_hAT-7N1 Oryza sativa

taagcctgccaatgggctggattgaaatgggcccaatggattggtttcagctttgggtgtagtttggatg

gatggaaatggattgaggagtttaatgggctggactggttagggtggaggtggaaacggtttggattgga

ttggtttttggtttctgatttttggatccaaattggattcagtccatggatttctttggatgcactagac

agtccagtactctccaaacctagcgtcatcagtcgtccacggctccactccttccgccgccgccgctccc

tccccttgcttcgcctcctccacctctccgccaccgtccgcgaatccgccgccaagccgtcgccggtcag

attccctcccccaccaccattgcttttctttttccctggtttttctcggtctttgatatatcctggttgt

tttgtttgtgtttttttttgggtctcttcctgatcttggtggttgcaggtgcaggtgaagcagaagaaga

ggaaggtgcgtgcgccgccgggcgctggcgaggatgtcctcctccggcggtccggccgcgtcgccaatct

ccctgagaagcccaagtaccacgacgtatgtgaacagtgtgattggtgtgatagtttctataagattttg

ttttttttgttggttttactgaggagttaacttgctcaagattacaatcttcgatgggtttatgtgcagg

agttccaagatttcaagaagaagataaggaggtatagtgaatgcaggttcccttcatctcccaatgcaaa

ttatttaacatatcattacagattgagtcctgatatgattttgagatgcaaattaattctatggtcaatc

gaattttagtgtgcaatttttagattgtagcacgtgtacaatgtgtgaaaaaaataattatagaccatgt

tctttcaactatcttgattttagatttcttcctctatgcagacaattgaacaaactcaataataaatatg

tccatacaaatgtcagagagttcaggtactaatagtccaaataataatcaatcttcaagaacacgtaaga

gagctaaagtatgggaacactttcagcaagaggttgtaatgatagatggtgtccccaagactcaatgcaa

atattgtagcttaagattgacagccaccaaaaagtcaggaacaagtcaccttattaatcacatagccgaa

tcatgtccagctattgatggtgatgctagaatcaacttccttgcaacaattaagaaacagactggggaag

gtttcgtgtttgttcccaagagaagccgggagctaatggtcagattcttcattcatgcagaggtgccatt

caagaaaattgaggatccttattttttggagtgggtggagtccatgcagccaactttcaaagttgtgggg

cgtcaaacacttcgtgatgatgcctttaacttgtatgagcgaatgagggaagacttgcatgctgaattac

ataatcttgattcgcacgtatgtctaacatccgatatgtggacttcaattcaaaatataggctacatggt

tgtcacagcacactatattgatagagagtttaacattaagaagaaaattataagctttaaggaattgaag

tatccccacacgggattcgccattgaagaagctataatgagttgcttgacatattggggcattagaagca

aactgtttactataactttagataatgccagtaataacaattctgcatgtcaagagttgataaagaatca

caagaatgcattgatgtttgagggtcagcatttgcatgttagatgttgtgcccatatcctaaatattttg

gttcaggatggtatgaagataattaaacctacaatacacaagctacgtgagttgttgaagcatttagatt

cttcagtatcaagaatgcaagatttcaattctatggcaaactcgaaaaatcttccttcgaagttgagttt

ttcttttgatacaccgactagatggaattccacctataaaatgattgtagaaggattaatgtacagatct

gtcctggatagctatgcaaatcaacatggtgaagttgcacctactgaactagaatggcagaaagttgaat

caatttgtgcttttcttaaagcttttgaagaagccacattatcggtgtcagcagataggaaaccaacagc

acataggttcttgcccttggtgctacaaattcgtcatgcattgaatgaccctgattggcagactagtgat

attttaaaggtattggctgcagctatgctctcaaagtttgtcaaatattgggacagtgggtttaactctg

cacttgtcattgccacaatgttagatccaagaagaaaaggagattatttgaatttcttttatgagaaaac

atctaacagtgtgacagagattgtcgaaaaagtgggttctgctgaagattggttgaaggattattatgaa

aaatacgaagggtttgtaagaagaaatgatgaacacatgctttcacattctcgtgaaggcagcagcagtg

ttggatcacctgttcttgggaaaagaaaactcgaggaagagtttgctttatacaagtctcgaaggaggac

tgcacggcaaacgaaatcagaatttgccatatatttagaagaagatgttgaggaggatagtgagagtttc

gatgttttggactggtggaagagacatgctcaaaaattcccggtattgtcatctatggccagagatttcc

tagcaatacccctcagcactgtatcttccgaatcagcattcagttgtgggggcaggatacttggtgacac

acgaagttcgttaacacctgaaatgcttgaagctctcatttgtgcgaaagattggttaattaaagccaac

gatcctttgatcaatattccaggtgctgaaggtataatagtgataaatgattcctccataaatgttctgt

gtatttcaatcatgaagtagtgatcatatatcatttacaggtggatggaatttgtcctgatttattcagg

tggtgaagtgaagaagagtttttggaaaggtgactttccatatacatatatgcaactttaaattcaactt

ttcctttggcgtattggaaatttggaatggaatacttctcttgaacctttcatgcttgaacaaaaaccca

agcatttttccaaaattgagtttaccactttgttgcctataaatctgtttgtatataaaatgttactatt

cattatttaccaattgaatcaatttggcacattgttttgataacactgcaaaggtcacattatttaaatt

gttattgtaattttgtatatttgctttcttaagcaacgaagtcacaattttctagttattcctggatata

aatttttgatgacataaataaatctacttctttcaggttactgtcatctgcaagaatgtgtggctgagaa

ttggaagggtttttggttttggttatcttagaaggtaatctaaagattgagacatgaagaaattgttggt

gttcagttatacatggttctgaggcttgtaatgcttgatagctttttttgttggagattttgtctcttga

gcctgttgctctagtagaatctctagtgataattgtaatatttagttgccaaaacttgttggatggcact

acttaaagcatcaaaagttctaagtatgctagtagcgttgattgattatttttgcatcttgcttaacttg

ttatttgtaagtatgctagtagctgtatgcagtgtgagtgttcgccggctgctctgctctgctctgcttc

ctgccgtggattgggtatgggttggaaaatggattccacccgtggattgtgttgaatttggactgagcaa

ctttgtgggttgggttgggtggggttagctcaaggaattcttggtctggattggagtgacaattttgcta

tagtcactttgggttgggttggattcggggtggatgccaacccattggcaggctta

>Os_hAT-7 Oryza sativa

taagcctgccagtgggctggattttattgggcgaaatggattggatgttgatttggatggtggtggttgg

attcaaatgggctgatattaattttggattggagcgggttggcctgtgaagaattgcggtttgggctgga

gtggatgcgggttggcctgtgaagactatccatggattcagcccatgggttagcctccaaaacaaaccga

actatccccactccactccaaaccctacactccccgcacgcgcctgtgcgcggtgcgccgccgcgcactg

ccgccgccgccgccacaaccgcgcgccgtcaccgccgcgtgccgtcaccgccgttcgctgccaccgccga

gcgccgtcaccgccgttcgccgccacagtcgccggactcactcaagaaggtgactcatctttcatgtttc

ttgaagtggcgttctctctctgcttggtcgagctccgctgatatatgttcttgctgtgggcagggaggtg

gcagatctgcggttgcgtgacctccatcttggatctcggtgagctggggggactgttcttggaagtggtt

cttgggagcatcctggtatggtaccttggctatttttgtgctttttctttgtttcgatttttagataatg

tgggttggttcgattggagatgctgaagagtggtgggtttctgttgggtttaatgccgcagctggtgtcc

catcttagttgtctcatgtgatacatactctgctggattgtagtatgtgtgccatcagacatcaggatta

gtcacaataattaggtcttaactcttgaaaggtaaagacttatgaagctattgccaaattagcagtacca

atgtctcatctaaccttgagaagtcatctagatctctggtatactgcaatgctactgaaaaagaaaaaaa

aaagatgttggagatttgaacaaattggaattacacaaattgattgctagcaatgttctgttattcgcaa

ttaattctgtcagatggttgtttataatatatcagtgcaattgcaccctttcccaagtcccaacctctaa

gtcatgtttattaagcatgcagcttttttgctgcttaatggtgactgttgagcgcatctagaaaatccag

cacttaaacttttaagctgttggtcaaaggataaaacattttgaatgtcagttcattcagttccatataa

ctactaatctgttgatttaggtggctgtctttcactgtagtattaagtagctaactattttatgtgttga

catgacatgaggcctgtctttcttgttcaaagatgtgatttgatattcattatgttattagtggagtttc

aaaaggtgaaaaaaaagtgcacaagaatatttatgcacggttctttgaaaagaaacatgtccagccccca

aatgctagatgcatgattacggtgtataacaggttgctgttgggtatgcttatcatttcactagttttta

gcacaagtagtaaatgtaaatattaattctgcacaagtagcaaatgtagatattaattttctattcttgt

caaacacaggtttattaaacatgtctatgcaaaattctgaaagttcatctgccaatagttctagtcatga

gctgccttcacaaacacgtaagagagcaaaagtgtgggaacattttgagcaggagcttgtcatgatagat

ggtcttccaaaagcacaatgcaaatattgtggcttaatgttgtcagcgactagaaaatcaggaactagcc

atctcataaaccatatttgcgaatcttgtccactagttgatggtgaagttagaaataggtttatatcaac

agttaggaaacaacccgtggaaaattttgtgtttgatcctaaaaagactgaagagttgatgataaaatat

ttcatccatgctgagataccattccataaaattgaggatccctacttggatgattggatggcatccatgc

agccaacttttaaacttgtgggtcgtcaatcaatccgcgacaagatttacaactattacaataggctgaa

acaagaattgcatgctgaattagagaatcttgattcgcgtgtatgcctaacatcggacatgtggacctca

aatcaaaacctaggatacatggttgtcacagcccactatgttgatgctgaattcaaaatgaaaaagaaaa

ttataagtttgaagcctgtgaagtatccacacactagctttgcaattgaagaagccatgatgaggtgttt

gactgagtggggcttgagtagcaaactgttcaccttaactttagacaatgctagtaacaacaccgctgct

tgtcaagaattagtgaaaactctcaaggatgagttggttttggaaggcaaacattttcatgtgaggtgtt

gtgcacacatcctcaatttgttggttcaagatgggatgagggtgattcgtgcagcaatagataagatccg

tgagatcctaaagtacattgagcattcaccctcacgcattcaagcgtttaattcaattgcttctagtaaa

agtcttcctccaaaatctggtttcactcttgatgtaccaacccgttggaactcgaccttcaagatgatta

gagaaatattgccttacaaggctatcctcaatagctatgcctctgaaaattgtgaacttctaccaactga

tgaagaatggttgcatgctgaatcaatttgtgaatttcttaaggcttttgaagaggctactagaggtgta

tcagctcataggacaccaactgcacacacattcttgccccatgttctttgtatccgtcatgctttgagtg

atcctgattggcaaaccagcgatcttttaaaacgattggctgctcctatgcacaccaagtttgcaaaata

ttgggatgaaaaattggcaaacaacttcaatttggcacttgtcattaccactgtgttagatccaagaagg

aaaagagactatttggatttcttttatgagaaggtgtctccccatggttctaatgttgaaagtaaagtgg

actctattatagaggagatgaaatcatactttcatgtatatgaaggaattgcaagaagaaggggtgtatc

ctacatgtcacagtctagtgagagagttagtgttgtaggctcacctgttttgggaaaaagaaagctagag

catgaattcaccctcttcaagtcaaaccggaaggttgcacgtactcagaagtcagagattgatacatatt

tagaagaagtatgtgaggatgatagtgaggattttgatgttttggcatggtggaagaaaaattccaaaaa

gtttcctgtgttggcaattatggctcgagactttcttgcaattccacttagcaccgtgccctcagagtcc

gctttcagcagtggaggaaggattcttggagacacaagaagctcattgacaccagagatgctcgaggcac

ttgtttgtgcaaaagattggctacatagagccaaaaaacaaggggattaaaggtataatatctcacgcct

tgtatttcgtctcagactttgtggtctgtgccatacaaatagtaattgtagatgtgttttcattttatat

tgtttgattagtcacaaaatctagattaatttgtctgagtgcccaccagtattactaatatttgtgccaa

attttctttgatctaggttcgtactgagtactgaccatatgatgttttgtgaagaaggtttggtatgatg

gctgttttttttttacaatggtggggtccctgtgctcagtaacttaatgttgttttttgttcatcagttg

gtgaaatgttaggcggaatttgtaacaccttgcaatgccttttatatctattagttgtatccttacttgt

atatattcttatttgagcagctagagcatgttcaggagatagatgagagcattgtgtatgtgaaggataa

gcaaggaaggtggcctctctcttctcccagaggggcaaaaaaggccccaaccaggactctgtcatcctct

gccaggtaacatcttcagctctaactctctgtctctgaatcttttctttctgtttgatgtgctcagctca

gatcaatccgctcaatttctcctcttgatgaggctgctcaggccaaatgttttgttttgtactcaaaagg

gttcagattagttcagggcttcagggtgttctctagtccatgatttgagcttccatgagctaattaggtc

agcagttagtagagtacttttttttcaggaagagttagtagagtacttggtagaaatagagaaaaatctt

ttctttctcttgcaaaattgtttgatatgatatgttgtatcctactatgttgattctggctgcagaattt

tggggctctcaaccttgttttcttcctctcttttttttttccatttcttgaaagtccttgtggttgtata

tagtagtatagatatttagttgaatagtggggatggatggattcattcttcattcttactagtgtgtcca

ctaactcatgctagtcatatcttatagaaataataatattcaacttgtaatctttcaatgttatttctga

aatataaatttctgatggcttctttctcttccagattatctacaactgcatatggatcaaggcatagatg

atgttttggtagtttttgttgaagatataaaccttattatggcccttattattgctgtagtagtgagcaa

ttgacttaattgtaattatatgacatcattagtcttctggtattcatttcagtgctggatcagctaacaa

acaatgcgatgcgtatcattctgagtattttttttgtttcattttcttgtttttgcccaattttcagtgt

ttcagcaagcaatgatgttcttttaatatttctgttttgaattaattgtctgcacgtgtgtctacactgc

tgctctctatactgtagctgagatccatggctaaagaagtaacgaggcctcctggctccaagctctctgt

agtgtagctgggatccatggattgaatccattccagtgaagtgggctgaacccattttagtgtaatggtt

tggggtgggttggattgaaatctgatgcggggtgggttggtttggattttggaggagaggaaatggggtg

ggttggtttggcatgccattccatgaggaggtgggctggggtggattcaaattggatctcagcccactgg

caggctaa

>Os_hAT-8 Oryza sativa

caggggcgaagcccaatagaggcccgtgggtgcagatgaaccccccttaaaaaaaattttagttattacc

cccatatctcggccctatatgcacccccctcggcccaatatagtgcacccacagcccaaacaacagtcca

gttaacgttgtaagcccatgaggagagtcgaatccagatcaatcttttctcttctcgacttctcgtatgc

cgcgtcccgtcgccgccaatcttgacgactccgcctcgcgtctgtttggcttccacgccgctgctctgca

atcagccaatcccagtctcccagacagcacttgcccatctcggcatcgacgcgccgccgtcggccgtcgc

cctcggcagctcgcgtcgccgtgcccctgcttgcttagcgcctgcttgctgctccagccgcaggagccgg

aaggcaggaagccggtcgccaggcaaccagctaaccaggagcaagcgtcagaccacactttcacaagcca

tgaagccaacaacgacatcatccatctccatcaactgatcagcacacagccacattgtgcgttagtagag

acaaggtaagaaagcatgcttcatctatttcttttttaatcaatttgatttattagatacaaaggcctaa

aatttttattggttgtttttttttacattgtagaattaggaggccagtagggttcaatttggaatccttc

agaattggaataaaattgcagattgggggaaaatttgagaatttgagttgaaggtgctatttggatgtaa

gtcatatcaattttcattctacttctgtacttgaatgaatgataaaatcagtatatatttacatcctcct

gaatcatctaatgataggttaattgatggaacgattttacaaaaggaaagtgccagagccgaatagtgct

aacaatgcatgtaattcatgtttggatgatataaactgggaagatgagattaaatatgatccaggattaa

gaaaacaaattgatgagtaccatcctaatctcagagagagggtgagaaggaaatacttggagaatggccc

ttgtcaacctcgcacatttgcttttcctatgacaggttcaagaaggtttgtcccggaatggtttgatgag

tttggaagttggcttgaatacagtgagtccaaaaatcgagcatattgttttttctatttcttgtttagag

aaaagaaggatggtggatatgaagcatttgttaaaaatggttggaatggttttcatagaaaagaaaggtt

gaaattgcatgtaggtgatgtcggtggttcacactatcaagcaatgaagaagtgtgatgatttattacaa

aaaagacagcacattgatgtagcttttcatagtgtgagagaaactggtaagagggactatcttactcgat

tgaatgggtccattgatgttgctaggatgctagtaaagctaggattgcctttccgaggtcacgatgagtc

aaaggagtcctacaacagaggcaacttcagggaatttcgtgattacacagcggagcaaaacccatcctta

agaaaggcaataggtacaaaaaaatcagataacagtcttttggttgctcctgaaatacagagggatattg

tgaaatgttttgcaaaggaagtgctacatgctattctagaagaaattgggcatgatgttttttgcttgct

agttgatgaatcaagagatgtttcttgcaaagaacaaatggcagtggtcttgagatatgttgataaatat

ggaattgtgagagagagatttgttggtcttgtccatgtgactgaaacaacttctgcctatctcaagtctt

ctattgatgctctatttgcagaactgaagctaagtctaaagcaagttagagggcaaggatatgacggtgc

tagcaatatgcgaggtgagttcaatggtttgcaatcattgatcatgagggaaaatagttcagcttattat

gttcactgttttgctcaccaactccaattggttcttgtggctattgtgagaaaacataaaggcgttagtg

atttttttactaagatttccatcttactgaatgtggtgggtggatcatctaagagaagggatttgattag

agatattaatgttaaagaaatgagtaaggcattgggttgtgggcaacttcaaactggaaccgggttgaat

caagagcaatgtcttcagagacctggggatactcgatggagttcccactataaaactctcaaaagtttgg

ttggcatgtttgctacaatagtcaaagtgctagaaattgtggaaaaagacaaaaatgattggaaaattag

agatcaagcatcgaatcttttggagtacttccaatcttttgattttgtcttctatttgcacctcatgttg

actatattaacaatcacaaacagcttgtcactagcattgcaacggaaggatcaagacatagtgaatgcta

tgaaatgtgtaaagtcaactaggctcaatttggatgagcttagaagagaaaagtgggagaaagttctaga

tgaagtctctgacttttgtgacaagtatgatattgtcaaattggaaatggaagatacatatattgatccc

aagaagcataggcacaaatctggaattacaaacaagcattactatcaagtggattgttttaatgatgtta

ttgattggatacttcaagagcttgacaatcgcttcagtgagacaagctctcaattgcttatttgctcatc

ggcttttagtccaagagactcatttcatgatttcaatctagagaatttgatgagtttagcaaaattgtat

cctagtgattttaattctggaaatttgagggaccttagccaccaacttggtctctacatcgctgatgtga

gggatgatggtagattctccaacatacaaactattgccgagctttctcaaataatggtggagacaagaaa

acatctttgttatccattggtttatcaacttttaaaacttgtacttgtattgcccgtcgctactgccaca

gttgagaggtgcttttcagctatgaagaatgtgaaaacatatttgcgcaataaaattggtgatgagtact

taagtgatagccttatttgttatgtggagaaagaagaaatgaagaaagtcaccaatgaggccgttgttcg

tcgcttcatgaaaatgcaaggacgcagatttgatgatgattgaaggtaatgattgtatctaagtgcgctg

gattctactttttatttgaaaaatgagaactgattttgatgttaacttgttttaggttatcaaatcatcg

tcgaggatgttgagataatgaaatagaacggtgtttgcttatgtttgcatatgcagatcatcttttgctc

atgggttagtaaccaattctcaattatcaatgttattagtggatttcttaattttgaaaaacaatgtttg

tcactttttttattttattgatgcatgtgtgtctctactgttgatgtaaaaaattattaatcaacggttc

tatcgaaaatgtagcttgaagttatatatattgatatacttgtggcaaaatctcgaacccccctccattt

tgctctagcttcgccactg

>Os_hAT-9 Oryza sativa

cagggtcggtctctccacggcagggcggctccagtgggtagtccgggtggtccctggaccaccctggatt

ttggcctaggaggaccccccccccccgacggcccaaaccaaaaaagcccaccacaggctagggttaggat

gacagaggagaggcgcaccgcgaggcacgcccgccgctccamtccgcctctgtctcgtctccgcctctcc

cgagtcccgactccccgctcctcgcctcctcctcctctcgctctgcggctgtgcaggcgccagcgccggc

ggccatcgcctgacgcctgcaggagcaggctgcaggmggcggscgwcagcccgtcggccgcccgggcccg

gcgcgggcgtgcggcaccatcccggccggcggcggccagccggccacgattttgccccgccgcccggccg

cccctcacggcggcgacgagcaccggagtcgggtccctctcctccctcctgctcgacgagtcgacggctt

tgctgctccggcctccagtgccctgcccccctgctgctgcctgcccctccgtgacaccggagagtgaaga

ccgagatggtgagttactcattttagtctttagtatttactttgaatagatcggtcatcgatgaatgaaa

aatgtggatgaaaaaatgtgattgaaaactgaaacttagattttttttttagcaacaacttagttagtag

tcaatataacataacttagtgtctcattaatttgtagtgcaaattgcagtgagacatgaaaaggaatgga

gacattgcatcgctttttcggaaacatgaagcaaaggcgaagaagagggcagatcttgttgatgaggaag

ctcaaattgaagaagaggagccaccactagttgagccacagcctgagccaactctagttgttgaggcaac

aaatgaagagcaagatgcacctccagagtatgatgctgatcatcttcaatatgatccaggactgaggtcc

cctattgcgagctatgatgtcaatgaacaagacgcagttcagcgtgcatatattctgaaaggtccaaacc

aatgttatccacatgatttcccagtcagggaaatttatggtaagaagcgccacttcaattttgtttggtt

tcataagtaccaatggcttgaatatagtgtagcaaaggatgctgggttttgcatggtatgttacttgttt

ggtwgtggaactagtaattttgttaaggatggttggagaaattggaacaaagccgatgcacttgacaaac

atgtggggggtataacaagtgctcacaataaagctcaagagaaatacaacctatttgtaagtggtggtcc

ttcaatagataatgtcattgtgaaggtgtcaaatgagagtgaaattcggtataaggctaggttgacttat

tcacttagatgtttgagatatcttttaaatcaaggattggcatttcgtggacatgatgaaagtgaagaat

ctagtaacagagggaattttattgaacttttgaaatggcttgctgaaaataataaagaagttgatcggtt

ggtgttgaaaaatgctcctggaaattgcatcttgacttgttctagtatacaaagggaaattatccattgt

tgtgctgatgagactactaagcgcatcattgaagaacttggtgatgatcattatgcaattcttgctgatg

agtgtagtgacctttcacataaggaacaacttgctctttgtgtgcggtatattgacaaacttggaagggt

atgtgaacggttccttggagtagtccatgtagctagtaccacttctgctgcacttaagaaggcaattcta

actttgcttagtgatcatcacttgactccttctcaaattcgtggtcaaggatatgatggggctagtaaca

tgaaaggaagattaatgggctgaaaacattgatcatgaaagagtctccttcgcttattacatccactgtt

ttgcacatcaactccaactagttcttgtttccgttgccaagggaaatgatgattgtgtatggttttttac

tcaagtttctcacttgctgaatattgttggtacttcttgtaagcgtcatgacatgcttagagatgttaga

gctcaaaagattatggaagcacttgaattgggtgaaatagaaagtggggttggattaaatcaagagatgg

gattggctaggccaggtgatactagatggggttctcattacaaaacaatattgcatattattggtatgta

ccccacaattcatgaggtactcataactcttggaaaagatcccacacaaagagatgattggccaagaata

catgctgtggttggagcttttgaatcatttgattttgttttcagtgcacatttgatgcttgtcattcttg

gatatacaaatgagttgtgtttgtgtttgcaaaagagagaccaagatattgttaatgcaatgtcacttgt

aactttggccaaggaaagaatgcaaaaattaagatctgaaggatgggaagaattttttcaaggaactgtt

gtttcattttgcaataaacatagtatccaagttccaaccttagatggaaaatatgtgccacatggaagat

ctccacggttttatccggaccaaacaaatgatgatcattttagaagagaagtgtacattggtgtgattga

caaaattagccaagagctsaacagtaggtttgatgaggttaatatggaattgctcatttgcatgtcagca

ttgaatccattcaattcatttgcctcttatgatgcacaacaagtacttaagcttgccaagttttatccaa

aagacttttcacccatggatttgatcagacttgaattgcaacttgggacttttattgatgatatgagaaa

agatgaaaggttcaaaggcctagaaactcttgctgagctctctattaaacttgttgaaacaaacaagcat

gttctttatgattgggtctatttacttctcaagttggtattgatattacctgtagcaacagcaagtgttg

agagggtgttttctgcattgagtgtagtgaagagcaagctgagaaatagtatgtgtgacaaattattgaa

tgattgtttaatcacatttattgagcgggatgtcttctctcaagttagcgaagaagacattataaagaat

ttcatgtctatgaagacccggagagtagaaaagaagtagtgtattataatcttctacctatctattgtaa

gatatttgaattatttatgttgtaactttcgtaacttcgaacttaaatgttggttttaatcaatttgtga

tgttctgagctatattgcaatatttgaattatatttccactgtatttgacatatttgacttgattttgta

acatcgtgacaatttgggacgccaagtaggaccacccaacgawtcgwtcctggagccgccactg

>Os_hAT-N10B Oryza sativa

tagggatggcagtcgggcacgacgggcacgggtagtgactacccatacccgtgcccgcgaggtaattcat

gcccgcgggcatacccgttactacatgacgggcaaggaacactacccatgcccgttgcccgcgggcgcca

tgtgcccgcgggcatgcccgtttacccgtcacaaactcacagttcaacatttcaaccacaaatctccaca

agcagagagacattatcacagtttaaagaaagggtacagttgtaccaagttcaacaatacaagcattaca

agttcatcaatactagatggtggcatttatactaatttgactgagggaccacatgtcagttggtttttgc

gggtaaacgggcgaagcgggcatgggtagtgactacccatgcccgtgcccgtatgcccgttgggtatagg

ttttgacccaataaaaaacccatgggtactaaatggagaacaaacccaactctattagggtttttaccca

cgggtaaacgggcaaacgggttaaattgccatcccta

>Os_hAT-N10C Oryza sativa

tagggatggcagtcgggcacgacgggcacgggtagtgactacccatgcccgtgcccgcgaggtaatttat

gcccgcgggcatgcccgttactacgtgacgggcaaggagcacttcccatgcccgttgcccgcgggcgcca

tgtgcccgcgggtatgcccgtttacccgtccaagatccagttcaactataattcccagtttcccacaagc

aaagacaatgtcacaatttaaagaatttaaagaaatggtacaatagtaccaagttcaatacaagcattac

aagttcattaatcacaacttcaaatgacatagtttagcacaatcaacaaacttcaaatgacatagttttg

aggcagccaaacaagccatcgcatgtaaagtttcttcttcttcttcctgttcagcatgttctgcagatgc

atccacaccaacaggaggagcagtttgtgaggcttgctcacggtcaggctggctgctgctgaccggagga

gtagcacgccggctgctactcgccggagtatttgtatgcttgctgctgcttgctggagttgccattagtt

caaatcactagcaatcaaatcacaaataatagatcaaaatcaggagagactagatcgaaaaaaatagaca

cctagatctagaggagagaaggggaatagaaggggaaaagaaggggaataccttgccggagcgactggag

gcggcggcgaccggagacggcggcgaccggggggaggggggaccacggctcggcgcagtggaggcaggaa

gcagcggcgacggcggccgcaaacggagggggccgtggatggaggacgaccggccggaggggtgctcggc

gcagacggtgaagcccctcccggaggcaaagcccctcccgtccccttctgcggccgtgaggcaaagcccc

tcccgtcctctctcgtcccctcacggcgacgactgcccggcggccggcggcgggagtgggggcgccggcg

gcgggggtgggggcgccggcggagaccggggtggctgggaggaggagtcgaggctgggaggcggctggga

ctgggagtgggaggagaggggtggcggctgggagtgggagtgggaggaggccggagggagagaaacccta

gagattttagatggtggtttttatactaagttgactgagggcccacatgtcagttggtttcgcgggtaaa

cgggcgaagcgggcatgggtagtgactacccatgcccgtgcccgtgtgcccgttgggtacakgttttgac

ccaawaaaatacccatgggtactaaatggagaacaaacccagctctattagggtttttacccgcgggtaa

acgggcaaacgggcaaaactgccatcccta

>Os_hAT-N10 Oryza sativa

tagggatggcaatcgggcgcgacgggcacgggtagtgcctacccatacccatgcccgtgagattatttgt

gcccgtgggtatacccattactacatgacgggtaaggatggttgcccatgcccattgcccgcgggcgcct

tatgcccgcgggcgtgcccgtttacccgccacaacaaaagcagtggaacaaaaagtctacaagctactaa

gttcgaatcgaacttaaaacatcaaatattctccgcctcggtgtcgtgtctctcctctggcggcgataga

gtaagaggagagaaataataagggaaataaaagggaaaccgtagaaaacaaagaacgtggcttatgtatt

ctagtggattttgctcctagcctacatgtaagttagatttcgcgggtaaacgggcaaagcgggcatgggt

agtgattacccatacccatgcccatttacccatcgggcatagcttttgacccaataagatacccatgggt

ataaaatggagaacaaacccaaccctaatagggtttttacccgcgggtaaacgggcaaacgggcacaatt

gccatcccta

>Os_hAT-N11B Oryza sativa

tatagatggccatatggcccgatggcccggcccaaggcccggtgatttggcccggcccgactcgggtcgt

gcccgtgccggcccggcccaacagccgtgccgtgcctgggctgctacctcggcacgctgggccggcacgg

cccggcmcgaagttcagggatgcgaaacagacttattctcaaccatatgtaaggcacggcacaaagaata

gggaggaaaaatagacgtattatatggcacggcccaaagagtagggacgtaaaatagacttattttagct

atatatatgtcacagcccaaagaggagggaggggaaatagacttattttaaagaaggcacgatgggccgc

ccgtgccttcgggccggcacggcacggcccgactactggtgggccgtgcctgggcctgaggtgcagcccg

tgggccggcacggcacggcccgaagtgccgaccgtgccgtgccggcccgatcgccttgggccgtgcctag

cccgtgcctgggccgggcgggccttatggccatctata

>Os_hAT-N11 Oryza sativa

tatagttggccataaggcccgaggcccgaaggcccggcccacggcacgcctttttggcccggcccaagca

cggcacggcccgacttgggtcgggcccgtgctggcccggcccgaccgtcgggccgtgcctgggcctcccc

accggcacgctgggctggcccggcacggtcgggccggcccggcacgatgggccggcaggccaggcccggt

acataaagtatggggactaaaaatagacatataaatgtaactgtctaaagaatagggaccttaaatagac

ttattttagctatttatataccccagcccaaagaagagggaggtaaaatagactttttgtagctgtacat

atgtcacagcccaataggagggaggaaatttagacttattctgaggaaaagcacgacggcccatcgtgcc

ttcgggccggcccggcacggcccgaggcgtgttgggccgtgcctgggccgtgagtgcggcacgtgggctg

gcacggcacggcccggtgcgtcggtcgggccgtgccggcccgacaagcctcggcccaggcacggccgggc

ctgggccgtgccgtgccgggcggcccacatggccaactata

>Os_hAT-N13B Oryza sativa

cagtggcggacgcaggtttaaaatacagatggggcggcagagctagccctaattaggtgatggcacaacg

cacacaaaccatagtcgctggttcacggcattggcgcaacagcgcacggcacgaacatcgagaaagttaa

caacgaagattggtagtcgaacaaacgtatatgacttatatgtagagagatgaaaaaaaatgagaaatgc

tgttagacaataaggggaaccactttcaatagttcgggggtataaaacgagatgaaaaattatttagggg

tttaagtgcttcgccaaaatagttcaagtgagtaaaatggatttttttatatatttaccaatttaactat

ttactgtagactgtattatgcgctaaagaaatagaaagaaaatcgaattttatgtatggccaaaatcaga

aacggtcaaaatggtactacttatacacgaattttcataaatgtttttgttcctacttaaaatacttgat

ttatctgattattcaatagtagactgatgactaatgtgttagaaataggactaaaatgaggtttgggggt

gtggggcgacacggaccgtagctgggccggccgacccaccctgcccaatgggtgtgtccgccactg

>Os_hAT-N13C Oryza sativa

cagtggcggacgcaggattaaaataaagatggggcgaagatgctcgtctcaataagaacaactaaacgca

aaaaataccatatttatgttgtaatcatataaacatatagtataatcaataaattaaggcaaacaaaaaa

acaaaaatgatattaaaataaagtaataatttttgcgccatacataattcatcatacataaaaagatact

agtaaacatatcaatttgcaaatatgagtcaattcataaagcatggttgatagatatcttttaaaaaata

aatatatgagaagatatcgcatacccagaataagatgattattattttgcttagaagggagagagagaga

aaaacattaggcacctaactatagtaggggacgggggtgagcagtgtcgaggaattgactcaggaaagca

tcacatacgcaggattaaaattggacacttgggctgcttatttatgggccaattgtgaattggatgtggt

ggtaggcggcccaagaaggatatggagggttggggcggcgcgggccgtagctgggccggccgcccaaccc

cgcctaatgggtggtaccgcccctg

>Os_hAT-N13 Oryza sativa

cagtggcggacgcagctttaaaatatagatggggcggagaacggagatgttcactcgataagagcaatcg

aacacaacacatatcgtatttatagttcaatcgcataactgcctcgatcattgtttgtggcgtgcgtgcg

ttgtgaagagacggactaatctgttgggcgttcacaccttgttatacctaattttgatggcccatgacta

ctataagggggtagggagggtcttgggggtgtggggcgacacgggtcgtagctgggtcggtcgacccacc

ctgcctaatgggtagatccgcccctg

>Os_hAT-N14B Oryza sativa

tagggctgtcaaaaaagctcgaggctcgcgagcagctcgagcttggctcgtcctaggctcgattcgagct

cggctcgagctccaaacgagccgaacccgagcctgagcaaaagctcgcgagctttaccgagccgagctcg

agcttctaccgagcctggccgagcttgactgtgtatagtttatattgcatgttcaccttattaatataat

aaactaatagataaatatatatattagtatctatttacacaataaattagcatgtgttagcacaatatat

ttattttaatcattttctactcttttattagtgtgattaactagaaaataattatacttatcaagagatt

atcattaaaatatatattatactacttatggccgagctcggcagccgagctcgagctcgaacgagctcga

accgagcttgcctaaaagctcgaaggtgcaataggctcggctcgagctcggctcgagcttgtgtcgagct

cgagcttgggcagacgagctcgctcgagctcggctcgttgacagcccta

>Os_hAT-N14C Oryza sativa

tagggctgtcaaaaaagctcggagctcgcaagctgctcgagcttgattcgttacaggcttgattcgagct

cggctcgaatactagacaagccaagctcaagcctaggctgaggctcgcaagcctagcaagctcgagctcg

aatagctcggcagggcttgattagcataagatgcattgcatgtaggaagatattcttgatgggtcaatgt

atacttgtcatcgtgcttgacctgctcaagtctcaagacctcatcaatgcttctcaataaagcaattatc

cggctgctagtaatagtactactattcctattagatgtagtagctagcttttgttaattgcaacgtttca

aagccatgaattcatgagaaaagctgcagaggcttcattcaagacaagtagcacccattaattttgacta

ccgataatttgcattaatatctcttatacaagagtaagttctttggagtttcaccaagtcaagctgtcaa

gctcgagtaggctcgaagctcgctcgagctcgaactcgagctcactcccaagctcgatcagtatccaggc

tcggctcgagctcggctcgagttcatgtcaagctcaagcctacatagccaaactcgctcgagctcggctc

gttgacagcccta

>Os_hAT-N14D Oryza sativa

tagggctgtcaaaaaagctcgagactcgcaagctactcgaactcgactcgttataggctcggctcgagct

cggcttgagcctaaaacgaaccgagctcgagcttgaaatgaggctcgtgcactttaacgagccgagctcg

aatagctcggtaaagactcgagtactagttagtaactgtcatgtctcatgctttaccttggtctccaata

aaaaatcatcgatgcttttccttggtctgctactactccaacattggtctatgtgaagtacagaagcaat

caattcttgaacactactcccaaagctagtagtagtagcaaagcagactactcctgtgctagtactagta

gtacagtacactagtgctcccgtatggccgtatctctacacaacgtgcccagctgcagctttctgtcagc

ttctctactttttgaaatatgtacacatgcttgtgttgcgttgatgcgagctcgattaggctcgaggctc

gtccgagccgagcccgagcttgactgcaagctcgaaggttagtccaggctcggctcgagctcgactcgag

ttcataccgagctcgagcctgaggatctaagctcgctcgagctcggctcgttgacagcccta

>Os_hAT-N14 Oryza sativa

tagggctgtcaaaaaagctcgaggctcgcgagctgctcgagctcgactcgtcctaggctcgattcgagct

cggctcgagctccaaacgagccgagcccgagcctctcccaaagctcgcgagctttgtcgagccgagctcg

agcttctaacgagcctagtaatatatgatttttttgttgttatttaataaattaggagatcaaatatgtt

atttgtatgccttatattgacatcatatatttattttattcttttttcctcttctattaatatgatgaac

cagaaattaattattttttaaaagattatctttgaaatatatattgttctaattaaaaatcaagctcggt

agtcgagctcgagcttgatcgagctcgaaccgagcctgtctgaaagctcgaacattttataggctcggct

cgagctcggctcgagcttgtatcgagctcgagcctaggcagacgagctcgctcgagctcggctcgttgac

agcccta

>Os_hAT-N15 Oryza sativa

cagggccggccctggccctatgcagccgaggcgaccgccgggggcccatgatggtggggggccacataat

ggtggggggccaacacctaatactagtagtctagtacctcatccgatgttgagatcaatgatttttattc

agaattaaaggtgttgcaagtgagtttgccagattcttcgatgtccgcgcctgagattctgaagtttgtt

atggatgcagatttctatccaaatgtttcagttgcctatcgaattctcttaaccgtacctatgacggtag

cttcagctgaaagaagtttctcaaaattgaagttgttgaagaactatttgagatcaactatgtcgcaaga

aaggttaaatggcttggctatgtgctcaatcgagaaggatatcttggacactattgatcttaataccgtt

cttgatgattttgcatcaagaaatgcccgaagaagtatcttttcataggaagcaatggatatgatggggt

tctattcttcattaagttaatgaataatgatattagttccaagttacaaatatattgttattttggtcga

ctttatttcttgataattaccagacatgttaaatttaatatatggatgtatttgttttcgttttgcaagt

aaaattagcgtctatatatatcataaaattttatatatagcttagagggcccatcgcgatgagttcgcct

agggccctcaaaatcataggaccggccctg

>Os_hAT-N16 Oryza sativa

caggggcgtgcctaggttatactacccggtgtcacaggacaccgggtagattattcaaatcctatataaa

ttacactatattaattaatatattagtcaataattagataattagtatgtattggacccccggtaacttt

tgttctgggttcgccactg

>Os_hAT-N17B Oryza sativa

caggggcggatctagaaaaaaaatagtagtgggggctgaataaactacatcagcataaatcgaacctcca

gtccccacatcctatggatctatggaaaaaaatttagtgggggcttaggggggtctccactgtcccgggg

gcggtagtggggtctcaagacccctccgcccccattgtgaatccgcccctg

>Os_hAT-N17C Oryza sativa

caggggcggatccaggaaagattaacaggagggtctgaacaaactatacaaaattatagccccctcttct

aatagatgtatgacagaaaattttagtggggtcttcatggggtctcccatggttacaatacgggtagtgg

ggtctcgagaccccagagaccccatggtggatccgccactg

>Os_hAT-N17D Oryza sativa

caggggcggatccaggaaaaaatagtagtgggggctgaacaaattacgccagcataaatcgaaacatgtc

tcacatgtaaattggctaaatgtcagttcaaggtacacattgaattaatgatatgtcgagaattttgtcc

ataccaaaccaatatcgaatagatgacgtgtcacacccaatcgtctattattcttcatgagtagtaattt

atacaaagaaaaaggcaaattaacgaataatcgaaacatacttattttggattcaaataatttagagatt

tagggaggaatggaggcatagagcataccactaatttctgttgaggtactaacatctgacgcgctttcat

atcttcaaattgttggatgattttttcatccgattgaaaaaaccctgacttgggtttctttggctaactt

aaacactcattactggatggaggatcactttactggatggaggatcactgccatttgtgttgcttaaact

aaaataatacgtgagcaatgttctttttgacatgttgacaatctaaaaatgtatagaaaataattataag

tttcaagcacaagtgcacaacctcattaagacacaattttagactatctattagtattagaggttactag

tttaccgatttctaacttcaaatcttcaatcacagtcacagtaggctgcgactgcgtcgttgactcgttg

ctgccttgccggccactgtgccgctgacctgctgcctgcctgcctcggcgccttgcgccctcgcggcacc

tcgccagcgagcggctcgccaccttcccggcggcgtggcggcgcaggcgctggagtgctggaccaccgcc

tccgcctggcctgcctcctgtctggcccatcgccggctcactggacgccgccacggcaccttgccgctcg

gcgctcgccagcttgcagctcaccggaggtcgccacctgctccgggcatgcactggaccaccggggcacc

gcctggcacgcctcccgtctggcccctcgccggacgccaccgcctgctcggccgccgcccgccgcccgcg

cctattcgcctagggttagggatttaggagaggaggaggtggccagtgggattggggaattggggaggcg

ggaggccgggagggggagtagccaaatgggccggctggaatctgccaccgtgggcctttgcttggagtgg

gctagacaaaacttgaagactgcgctcgtggtcgtgggctgctcttcgaaacaagcccaaatcgcgaaat

aacatcgtcttcaacctccagccttccctccagccaacctgacgaagctccagccaccacatctcgtgaa

aaattttagtgggggcttaggggggtctccatggaccctggggcggtagtggggtctcaagacccccccc

ccccccgcccccatggcaaatccgcccctg

>Os_hAT-N17 Oryza sativa

cagtggcggatccagaaataaatagcagtggggtctgagcaaactagatagagttacagtcccccctccc

atctacatatggtaaaaaattttagtggggtcttcgtggggtctctcatagtcttcacgccagtagtggg

ggctcgagaccccgccgcccccatgctggatccgccactg

>Os_hAT-N18 Oryza sativa

caggggcgaagccaggatttgagattagggggtgtggggtgaagataaaccatgagaccgaataactaat

attgatagcaaaggaggtcaaatagtcaccgtcaataacaatcaatgcgtaaactagtcctttaagaaaa

acttgaattttagctctttaataatatcattgaaagtttgaaactagtctttattgctaaattcttttaa

tgtaaactattgaataatctcacaaataagtatcatctattttactttgaaaaagatgagaaatcacaaa

ctaattttgtagatcatgtttcttgctaatatttttagacttgctaactaactaaactgataatactttc

aagctacacaaaataaactagataaatacttatcttagctaaccttaatatagagtaaaaacatgggggt

gtagtggtggtagtgtttggataggggggtgttggcaccctttggcaccccccttagctgcgccactg

>Os_hAT-N19 Oryza sativa

cagtggcgtagctagctaggtggcactgtggtccatggaccacctaaaaatttgatcgagattaaaacta

tagtatatgtgctagttaatataataaaaattgtatagtggaccacccttttaattgacctattacatat

agatggaccacctttactctaaatcctagctacgccactg

>Os_hAT-N1B Oryza sativa

taggggtgaaaacggtcacggaaattcccgatcgaccgggcgccgtttccgtttaaggggtaccgtttcc

gaccgtaccgtttttggctatttttaaattttgttttttctaatttctttctgcatcgtattaatgtcga

tactgagtagtttaaatgataaatatggtcaattaccggccgatcgagcatcgtttccgaagagatgcta

ccgattccgaccgtttccgatcgttttcaccccta

>Os_hAT-N1C Oryza sativa

taggggtgaaaacggtatggaaatttcccgaccgtaccgataccgtttccgcaaaactaatataataata

atatttttaccgatggttaccgttttcaaaatttagtttttttaatttctgtcagcgtggcattgaagtt

gacactaaatagatgaattcataaatatggaaatttcccgaccgtttccgttcgttttcgaaaaaaataa

taaaataatgataccgtttccgaccatttccgaccgttttcatcccta

>Os_hAT-N1 Oryza sativa

taggggtgaaaacggtacggaaatttccgagaattccggctaccgtttccggtttgcaccgaccgtttca

atcggtatcggtaattgtcggtatcggaaatggaaacggtaatctctttcggaaatggtagcggaaatga

tatggggttttttccgaccgttttgtcggtaaccgttttttatcggtaatttccgtcaaatttccgacaa

aattccgaagaaggaagtcggctgggcctggctagcaggaggaggagcccattaggcattaaggagcagc

ccattccgaataaagaagccaactaggcctgaccgcatcgtcgtcttcatcggcgctggggcaccgccgc

gccatgttgggcgccgccaccaggtcgccgacgccggccgcaacctccttcgccccgacggcctccgtcg

cagccggccaccttccctcccccatgctccaccgccggcttccgtgccaccgagccaagcgccgagcgcc

gccgcaccgccccagcctcctcctccccaccgatgccgcccctttctcctctctccttcccccaccgccg

ccctgcgccaggccgccatctccggcgcctctctcctctcgggcgcgcgtgaaggagccaaaggcagaga

agagaacgagagagaaaacagagaagaaatcgagagagcaggaaaaggaaccgagagaagggaaggataa

gagagagagaaaaaataaaagaaagagaggaaaagaaagactgtgggatccactggcgggccccaccatc

ttgactcctctctgtgatcgacctggaaataccaaaaagtctagtttagcatgaaacttaattacaacgg

tagttatacagtgtcaacataaaagttcttatgcaatgtgccactgtaggacgacgatatagctatgtca

attgatgatgaaataatgaatgatgtttgacaaggagctgatcagctgaagccatgccagcttgtgagct

tggtgttctcgaagacttcaaatctatatcctaaaaaaagtcggacaatattagttatgctgtaatccta

tcatgtaagccacattgtatgctttagccacattgatgttgaagtatcattgaactttatttatattatt

tgtcaatcttattgtcaacttattaatcatcatataacggtgttgtgtggcagcgtaatgatgcaagggt

gaaattataaggtcatcttcatacatagttaaatgcgggatgataccacagttcgctgtttccgacatga

attttcgacttccgtccgatatcggtatattcccgttccgacggttccgtttccgagataccgatataac

cgatatcgttaccgtttccgagtataccgttccgattccattttcgagaaaataaaatgaaaatggaaat

ggtagagggtttttccgaccgtttccgaccgttttcatcccta

>Os_hAT-N20B Oryza sativa

tagggatgaaactggttcggatagtttccgtccgtccggaccattttttggattcggatagtttcggttg

gaattatccggaaattctcggattcggaaacgaattcggatatttttttctcggaaacgaaaacgaatac

agtaagggtactatccgtcggaatcggaaaacggtcggaaactatccggaatttttttcggatatccgca

gacattgagcaaattttagaaaaaacacgtatatatgcataactttttcatacggaatcagatgaagaca

aactttatatcaacattgtagagctcgatgagatctacaactttattattgactattttattatttgagg

tcatttaagggtctaaatattcattataatataccatattaatttttacaaaatctcagatctacaattc

aaacgacatctgatggagatgtgttccatatcaaaattgtagagctcaacgggatctaccactttgtagt

ttataacatttgtatttgaaagcatttaaagtataatacaaacattacaagtttcgaagatgtgaagtaa

aataaataacatctccaatttatacaatggtaagtaagttatacatctagtaaaaggtcttgggaataga

aaatgataatcatatttgcatatggatctcttagaggaacaaccatgaaaatcgatttttcacatacagt

tgtgggaccgtctacaaaattgacgatataaatgttggagatgttgaactaaaataataagcgatatctc

ttcctttatcaagaaactcaatttgaggggttttttatataccggtaaatattcgctaccgtattcgttc

cgtctcgtattcgctccgtatctgtattcgataatattcgatttcatttccgtatccgagttttcgattc

cgattccgattccgaaaaaaaatatgaaaacgaatatgataaaactagtttccgtccgttttcgatccgt

tttcatcccta

>Os_hAT-N20 Oryza sativa

tagggatgaaactggttcggatagtttccgtccgtccggaccaattttcggattcggaaaaattcggtcg

gaactatccggaatttctcggattcggaaacgaattcggattttttttctcggatacgaaaacgaataca

gtaagggtgatatccgacggaatcggaaaacggtcggaaactatccgggatttttcatcggatatccggt

tcatgcaggatatttttcagcaaaaaaaaaaaaccgaaaccctagccggctacgcccggccgcccccggc

cgcggtggccggcgccagatccccgccgctgcgccgcccgccgccggccgccgccgccgccgaaccccgc

acgggccgccaccagagccgcgccgccgccgcctgccgctggagcccaccggctccgcccaccgaagccg

tcgccggcagccgccgccgccgagcccgcccgccgccgcccggcggaaccgcgtccgcccgccgccgccg

cccgtcgccgcccgccgcgcggcgcgccgctcgaggccgccgtcgccgcctgccccgccccgcgccgctc

tcggccggccgccgcccgccctgccctgcgctgtggatgagaggaagtgagagagagagagagagtagaa

agtgtgtgtgagagagaggagaggaagagtcagagaagataaggttggggtgaaaaaaatggtggatttt

ttttcaatgtaaaaatcaattttttttttatatgtggctctttaaaacgaatttaaaggcactaaatcaa

ctcatatgaaaaaagttgttaaaaacaaagttgtataacttattgagatctatcatttttattttggtca

tttctccatccgagtttgattaaactatataaaatttgaatttaaaaatataagaaattcaaataatttt

tcgggtagtaaatgatttcaaatgaaaaaattgtcaacaacaaagttgtataacacatcaacatctacaa

cttttgttttggtcatttttctatatgactttgtttgaaccgtttgaatttgaatttcaaaatatgacaa

cttcaaacaatattttgaaatactaaatgatttcaactgaaaaagtcatcaacaacaaagttgtataact

catcaagatctataacttttattttggtcatttcttcatccgacaaagtgatagtaacattgttcacaaa

atttacatctatctcatttggttctataaactataagagagatatgtaaactttgtgaacaatattacta

tcactttatcggatgaagaaatgaccaaaataaaagttgtatgtcttgatgagctctacaacttttatgt

tcatgactttttcagctgaaattaattacagcttcaaaatattatttgaagttttgaaattcaaattttt

aattgataaaacaaagtcacaagaaaaaaatggccaaaataatagcagtaagaacacaataacatgatag

agcatgattttagaaacatttaggaaaaagaatcatccaatttggagttcatatgagtgagataaattag

tttcaaattttcaaattttatttttgcatacggcttcttaagtcacccgtatggaaaaattgatttttcc

atgcgggtccttaagtggtccacatacaaacaaatgaaaaaaattcaattctgaaaaataaccactcaaa

tttttaaaaactttcaattgaaatgttcaacccaaaatttaaaatattcaaccaattttcaaaaatttca

atgtgcctacgaaaatttcagtgatacacattaatttatttgtttcatgataacataaatttgtatggaa

agtaaacataacattttctatctcccccttgcccttataaatagagataaaatttgaaaatactgatgtt

ttgtttttgaaaactaatggctacatctcttcctcgatttaagaagcttattttgaggggtcttgatatt

ttgataattattcgttaccgtattcgtttcgattcgtattcgctccgtatctgtattcgataatattcga

ttccgttttcgtatccgggtttccgattccgatttcgattccgagaaaaaaaatatgaaaacgaatatga

tagagctagtttccgaccgtattcgatccgttttcatcccta

>Os_hAT-N21 Oryza sativa

cagtggcgaagccaggatttagagatggggggtgccatctacgagacgtggtaactcacttaaggttcag

tgaacagtgatattgtagcgttgatgtatgatcgctaactcgacatcatacatgttatagcgattagcta

caagagagaatgagaacccatagatcatgatttgccaatttaaaattcagtaatatttttaatatatgga

tgattgaacagtttgttccatgattttaattggagtcttgcaatcacttctaaaatgataaatcagttag

ctaagtcatttcataagtcctatatatatagctaggcagttgtcctatatagttagtctcatctgcaact

aatgatgtaattaatggctagattataagggctaatcatccttgggagaagagaggggaggggaggtcac

ataggggttaggggggtgttggccctccctggcccctacggtggcttcgccactg

>Os_hAT-N22B Oryza sativa

cagtggcggagccaggataaaattatagtgggggctcctcagccttttgggccttctaacaatctcagac

aaagtctattttagcccccttgcaatagatatatggatttaaattttagggggtcttaatgggggctcta

atgattcattagggggtagggggggggtctaaagaccccccagcccccacgctgcctccgccactg

>Os_hAT-N22 Oryza sativa

cagtggcggagccaggataaaattatagcggggctaaaccaaaatgaccaagagcaactaagagagattt

agggttgggttaggattttttgggccttcttagccttttgggccttctaacaacctcaaacaaagtctat

attagcccctcatctataaatatatggattagaattttaggggggtattatgggggctctaatggtatat

tagggggtagggggtctaaagaccccccggcccccacgctgtctccgcccctg

>Os_hAT-N23B Oryza sativa

caggggcggagacaggagtagggcagctagggctgcagccctactcctgatcctaaatattcattgaaat

tccattttaaaacttcaaaaactagaagataaaggcaaaattatatacattcaactaattgagccctagg

tttggaaaaattctggctccgccactg

>Os_hAT-N23C Oryza sativa

caggggcggaggcaggcctagggcagctagggcttgagccctaggcctgcctccaaaaacttgcttaaat

tttgttgattcaccatgtaaatttttcagaaaactaatactccattagtcgagccctaccttagccctag

tcttatataatttctggctccgccactg

>Os_hAT-N23 Oryza sativa

caggggcggaaacaggcatggggcagctagggcttgagccccatgcctggctccatgatccccattataa

tcttgaaaaataccatgtaatttctaaaaatatttgaactcccgttagttcagccctacgcgtgtagatt

ttctggctccgccactg

>Os_hAT-N24 Oryza sativa

tatacctggccaaatgggccgtgccaagccgggccggcccgagcacggcagtactgtagccggcccaggc

ccggcacggcacgccagcctgtgggccgtgccggcacggcccgttatcccgtgccgtgtttgggccgatg

cctcagcccgtgggctggcacggcacggcacgtttactgtagcgtgccggcacggcacggcccaggcatg

gcacggcccaccggcccatttatttttcctacgattttctaatttttcccatatatttctcatatttttt

atttttttccacattttatcctatttgtaccatatattttatgatatatttcctaaattttcctcacatt

tatcctatatttatcattttttcctttatattatgttttttctgccttttttctttttttcccctcaaat

ttcaatttttatgggccacatgtactagtgggccggctaccagcccacgtgccactacagtagccgtgcc

ggcccggcacggcccagcaccttcttgggccgtgcttgggccgtggccttggcacgtgggccggcacggc

acggcccgctacaggagccgtgcctaacgggccgtgccctagtgggccgtgccgtcggcgtgccgtgtcg

tgctgggccgggccgcccgtttggcctgctata

>Os_hAT-N25 Oryza sativa

tagggatggcaatgggtcgggtcgggcacgggtagagcaaaaccatgcccgacccgatgcccgacattgc

ccgcccaaaccctacccactagagttagcgggcaaaactccgtgcccgtacccatgcccggcgggcgcgg

gtatgtccggcgggtacccaatggatctcacatgaataataacttgaaagatatgtaatttaacaaagag

aaataaaatcaataatttaccaaattatcggttatcaacaataatttgaattacatcacaacttagtatc

agttagcatcacaaatgaaccgagagaaatggtataagaattaagaaatgagtcaaaattagctaataca

tatgtatcgggtcgggcatgggttacccacgggtaaaaaatgaaacccgcctattgcccataacgtcttc

gggtctcgggcgggcgtgcccatgggtaaaaagtcacacccacgcccgtgcccatcgggtcgggtatcca

cggatacccggacccatgggtaaaattgccatcccta

>Os_hAT-N26 Oryza sativa

tagagatggcaatggggacccataacccgagacccgacgggtttttactctattaggagctaatcgtgtt

ctaactcttatacccatgggtctactaacgggcgaaaactcttacccattgggtacgtgggtatgggtcc

gttctttacccacccgtacccaccaacccgtgggtagaaaaaacccgttgattgagcctaaaactatgag

caatataagtctcaacgactactaaatcatagcataaaactcctttcacatcttaaagcattgctctagt

ctagttatatgaaattattatttgatttcttgattatgtaatgtggttgtataatttttttactcgcctt

tgaacactaatattaatagcattgttgctagagaaaatattgatggtttacaactctaatatggctaatt

atagtacaaaactataaatgctacaagtcaattatatgggtatgggtacccgttggatacccgttacccg

catgggtgtgggtatggggacgattttgtacccgcgacgggtgtgggtatttaagtgggagtatttagac

atcgtgggtgtgggtatggggcaagagaacccgatgggtatgcacccgttgccatctcta

>Os_hAT-N27 Oryza sativa

cagtgttctaaaaatcggccctaggcgccgcctaggcgcccattaggcgctaggcgccgggtagccgccg

cgattaggcattcgatggtgcattaggcggttaaattacttgggtagtgttttattgctattttagtgtt

atatagtctaatttctatgcaatatgcctgctaatttcttggatttgactatttgacccgacctaggcgc

ccgctaggcgccgcctaggcggggcctaggcgccaattaggcgctaggcgccccctatccgcccaactag

cgcctagcgccttcttgaaccatg

>Os_hAT-N28B Oryza sativa

cagtgttgtggatgacggaatacggatgtcggacggagagggtaccgctaaacgattaatcggagtacgg

acgaataatcggaatttgacggaaatttaacgttttggacaaatatatgtacataactcataatagctta

ttatttttgacagataaatgcatataactacatatatacctactattcttaaataaaaataaggataaca

tgacattaactcgacatatagtaaaatataggtctgaacactcatagttgtgcataaatagtgataggtt

tggttcgtaatgcataaaggaaataccaaaagcaggtaacactatataagtgacactaaaacaagtccct

gagagaggttagatgaaaaaactaacctagactgcagttgtagcggcaggaatgagctgcgagggttttg

ggattgcaaacgacgttctgttagctatatagtgctaggttacacaactaatgggccagtatagttatcg

tgggctggctgggcagggccttcggccgtcacgtgattaggcccgtttattcggccgattagtcggatac

ggccgattaatcgctaatgacggtgattatacgtcctgaaacgactaatgtgcaccgttttgctaaaacg

tgacggagaggtcggcgccaccgttttgacgtccgaatatacggccgtaccaaccgttatccacaacact

g

>Os_hAT-N28C Oryza sativa

cagtgttgtggaagacggaatacggttgtcggacggaaagggtaccgttcaccgattaatcggaatacgg

acgattaatcggaattatacggaaatttaacattcgtgaatatatgtacaaattttataatattatatac

ttatttgaagatttagatgtgtacaactatataaatcaaatatgttcatgttgttttttaattaaacaga

taagtaaaacattaactcttctaacaccggatttatatatcacatggtggtagaattggttgcattaaat

aagtaaaacataaaaatatatgttacaaaacaaaaacaattctaaattaagtaaaaaaaactactctaaa

tcatcatcaaatacaacgtacagacgattattcgggtgattaaacggaaaaacgaccaactaatcgctaa

aaacagagttttaacgttcatgaacgataaatgtacaccgtatcacgttaacggaacggttttactaccg

ttaacgtatatacggccgattaatcggtcggtacggccgttttccacaacactg

>Os_hAT-N28 Oryza sativa

cagtgttgtggaagacggtatacggatatcggacggagagggtaccgctaaacgattaatcggaatgcgg

acgattaatcggaatttggcggaaatataacgttttgaaaatatatgtacatagttgataatagttttcc

ctttttgataaacaaatgcatataactaaatatattccttctatatatccttaaatagaataaccatata

tgatatttaaataagttcataatggatagctgttactgtatatctcaattctgacgtagtgatgggtttt

gttcataatgcatgaaatgaattatcaagagaatgtagtacatagcataaatgaccgtaatcaaggtccc

aaataacatatgtatcgcaagagcttcatcaaatacaaactccaataacctgatcatcgtcaaaatcaag

gaactcatgttcaaactttagaggaagtcagttccttgtcattttacatcttgctgccttgtgagaattt

cggaagagcaaacttctagtcaatagtagtaaagggcacagctaatgagctggattgtggaatcgacctt

tgtcaccacgccgattatacggctgattagacggagacggacgatttaatcggtaatgatcggatttatg

cgttcgttaccgactaatgatgaccgcatcggcgtgacgtgacggactgatctccgtatccgtattatcg

gacgtattaacggccgttaacaccgttttccacaacactg

>Os_hAT-N29 Oryza sativa

cagacccatccctaggcacgtcgcggagatgtgcgaccgtacaggaaaaattaggatcccaaaaatttat

gtgtttcaatttaagccaatacgttccgatatatttaggatctctagattccattttttttttcaaattt

cactctttctgcttcactgaatcggctagaacgagttgttttcttctcaactccgacaagtgttcgatgg

taatcataactacatcaaagaactgtatttcgtgacgacgatgtgaacgtgcgttctaagcaataaggat

tccatttttttccatttagtaagaaaaaactgaattgttgcaacgattcttcttctacttagccggaacg

ccactacatatgtaggtgggtgagcttctttacatagctctagttgtcattggttgcaaacaattcttgc

ttgtttgatagcaggacatagctaaagaaaggagtattagattgatgctagctttcctaacatggttgga

aggcaattggtaagacataaaaaaaaaagcagtggtaatagagacccatcccaatgtattataaacaata

tcatatagccctgatctattatacaattagtattttctaaagataaggctttgttttatattttgcacat

ggccctcaaattgttagggatggccctg

>Os_hAT-N2B Oryza sativa

tagggttggcaacggggcgggttgggggcgggttgggctggaacgcccccgcccccgctccccgacttct

ccccccgaccccgaccccgcagaggtggtcgggtggaaaacgtcacccgcccccgtccccgcaggggacc

cggcgggtgagcggggccccgcactacccagctggaagacgacgaggcgagccgcggtggacgagacgag

gcgaggcccgcactacctagccgtcgatggacaacggcgacgaggcgagccgccgccgcccgcgcttggc

ctggccttcgccgcccgcgcctggccktcgmmgccgccgcccgtgccctggccgacgccgtcgcccgcca

ggtcgcgcctggccgtcgatgccgccgcccgcgcctggccgtcgccgccgccgcgcctcgccgcccgcgc

ctggtcgccgccgccgccgcgcctggccgccgccgccgccgcccgcgccttggccgccgacgccgccgtc

tgcgccttggccgcccaccgcccgtgcctggccgtcgacgccgccgccgcctgtggtgtgtgggtgacgg

ggagaggagattggggagagggtgtggaggggtgggaagattagggtttggctcttttatktttatatat

gataggcctttttagattgggctcttttgggcttacaacattatattttaaagtctaatatacatcctcc

ggggctccgcgggtcacccgcgggtgaaacgcctcccccgtcccctccccgcccaaatacggggccccga

ccccgactcacccgcgggtcaaaaattgcacccgcccccgcccccgtaggggcgggtccccgcaggggac

ccgccccgcgggggaaattgccatcccta

>Os_hAT-N2C Oryza sativa

taggggtggcaacggggcggggcggggccgggttgggttggaatgcccccgcccccgccctcatgcgggt

atccccggccccggccccgcaaaggaagtcgggtgaaaaacatcacccgtccccgcccccacaggggacc

cggcgggtgaccggggccccgcatcacccggatggagctaatacttcataaaaatattgcaatacactaa

ataatattatgataaaatttcaatagcccccggttgtgtgggttggttgaagaaaggaaagggattacgg

ttgaggggaactagggtgttgagcttttatatgtcaattttgaatgggtttgttgggtctatattattgc

aatgagctacttttagagaagtaactcacatccgggtggggcccctccgggtcacccgcgggttaattcc

gatccccgcccccatccccggaagtttgcggggccccgtccccactcacccgcgggtgagaattctcccc

cgcccccgtccccgtaggggcgggtccccgccggatccccggccccaacggggaaattgccaccccta

>Os_hAT-N2 Oryza sativa

tagggatggcaatttcccccgcggggccgggtcccctgcggggacccgcccctacgggggcgggggcggg

tgcgattttcgacccgcgggggcgtcggggmcggggccccgcatttgggcggggaggggacgggggagac

atttcacccgcgggtgacccgcgggccccgggggggtgtatatgaaccctcaaaatatagcccatagaag

gcccaaaaagcccaacccamtaaaaaaatcctgaaccctagcagcatatccctcagtctctcccmtcccc

atatccttcccttcccctcccgtgcggctgaggcgtgacctaggcggcggcgcgcgctcgtgggcggctc

gcctctggatctcgccctctcggcgtcgccggcgcctgccgcccgccggccaccatctcccctcccctcc

cctccgtctcccctccggcgtccggcggctccagctcccctctccctccggagtccgcggcggcggcgca

cagggcggcgccgcggcgggccggcggcggcgcaagggcgcggcggggaggcagcaccggcagcaagggc

acggccggcggcacacagggcagggcggcggcgcgcagggcggcggcggtggcgcgtagggcggcaagat

cacagcgatggatatcatgatgtgtcttgctgaattttgtgatgaaattgaagtattcatgctatttttg

atggtgtgttaattatgtacaatatttgtttttgctgaattgatgattgtgttgtattttttgtgctgat

tggatggagatgctacccaatgcggggccccggtcgggttgcggggccccgcaggggtcgggggcggggg

tgatttggccccgcatcaccggtcggggccgggggcggggggtgaagtcgggggtcgggggcgggggcgt

tccagcccaacccggccccgccccgccccgttgccatcccta

>Os_hAT-N30 Oryza sativa

cagggccggccctagggggggtcaggccgtgcggccgcccgaggcccccaaaatctaggggccccctata

cacatatagtatatatactacgtactgtataattctagtccacgtgccatgggtctatattaagttagac

agctgaatagcccaatacagaaaatgaattttagtccatcgttaggcctgttttagcgaagccgaagcga

tcttcatctgactcccgtcgcttcgcctcgatcacatcacgccactcgcgtcgtgccgcctcgatcacgt

cacgccgctcgctgctcctgcgctctcggtgacttctcggcgtctcgcaaacggaaacagcaagtcggca

aatcgcggctcgccagtagccacgctcgtgtcgatcgtccgccggtcgccttctctgctcgctactgcta

tttatttctctttgaagttttaaaatgttatatatagataattttgtcacatcgaatattaaatggtgtg

tgtttataagactatataaatatatawattagtttatgtatatatgtaggggcctccatataagtttttg

ccctggggccccaaaaccccaggaccggccctg

>Os_hAT-N31 Oryza sativa

caaggtaataaatagcgtatagcgggttggtagcggattagtgtctgggtagcggattgcggatagcggg

cgctattgcggacgctaagttccaatagcagaattaagaaaaataaccataaatttagattgtatatcaa

tattaacatcaagtttcacttaaaatttaaaatttagactgcatgtttaattactcctatatttcataac

ataaaaaattatgaaataacatataaatatataagataaagaagataaacaatgctaagttattctatcc

atatgtgaagaacaccgtttaagcattatagcgatccaacaaagtcaatagcgggctgttatagcggtcg

cgaagcgctatagcaggctgctatagcggtagcgaagcgctatagcggccgctaagttccaatatgctat

ctgcagtccgctaccttgtagcggtagcggtagaggtccgctaccgctatagggccgctattgcgggcgc

tacgaacgctatttattaccttg

>Os_hAT-N32 Oryza sativa

catagttattaggaccggaccggaccggcggtcggaccggaaaaaaccggaaccagagcctccgccggtc

cggtccagttaaaagaccggtgtgcaatcgacccggctataaaccggttggaccgcccggttttgagaaa

aaccgctataaaccggggaaaaaccgctttgaaccggctgctcgcggagcgcgtgcatggccgcatgggc

acggctggtgggggagaaaaatgagggaaaaggcgcttgggtgggattcgaacccaggaccacatgggag

gaagcaaccaccaaccaccaggctaccatgctccttgtgtttggatggaatgagtaatatatttaaccta

ttgaaccgctttagaccaacggttcaaccggttgaaccagtgaaccattgaaccgagggcctcaccggtt

cgattaccggtccggtctcaataactatg

>Os_hAT-N33 Oryza sativa

cagggccgtcgatgagggcgtgcgaaatgagcgaccgaacagggccccaaaaattaggggccccaawttt

tttatattttaatctatgccaatgcgttccaatatatttgaaggcctctcgttccatttttataaagttt

cactcttactcatttgttgatttggctagaacgggcttgtttttttagctccgataagtgttcgatggca

atcatggccaactcaaagagctatagctcatgacgatgatatgaacgtgcgttctaggcattaaagattc

catttttctatttaataaaaataaattactacatcatgacataatatttttctaacatttattttaaaaa

tcttcttccgactgagatagagctccactgctaatttttttgtctttcctatttttatgggcgttaaatt

gtgtttcgcaccggggcctcgaattcstaggtacggccctg

>Os_hAT-N34 Oryza sativa

cagtggcggattcaaaaattttcatgagcctgggcaagcctaacaacaaatgaagtatatacaagtctat

ataattcatagatcattttagcatgagcatttgctttaaagtttgtgttaagcacgtttgctgctgtgct

agttgcatcatacgcctgccggtgactaagcaaggtgattagcaaggtaattaggtattaatctactggt

gcattgctgcatgcagctggcggagcccgggccattgcccgtgstagccgggccctggctccgccactg

>Os_hAT-N35 Oryza sativa

caatgttttaaaaagcggtatgcggtttcgtgtggtcaccccaccttgtagcgcttatgcggcataatcg

gacgcttatgcggtataatcggccgcttatgcggcttaagcggtacaaggttggggcacctgttccttgt

atgcggccttgtaacttttttttaaaacactg

>Os_hAT-N36 Oryza sativa

caggggcggatctagagtgtaacaagcgggggccttggcccccactcaattttgcagcgagtgggggcaa

atgcccccttgacactttttacttccttagtctacctaagcggtagatccttagcccccactcatgctat

tttctgcctccgcccctg

>Os_hAT-N37 Oryza sativa

caggggcggatttaatgtagaaatgatgggggccttggaccccagtcaaaattacactccttaggtatat

ccctagtagtatgacctatgggcccccactttaattgggaggaaggaccccagtccccaagttttcgttc

ctttcatcgcaatcgcgcgtgcgtggaggggtcgaacggccgctccctttctctgattctctcagattcc

atcaactggcaaatccgcaaattagggattagggaagggaacgggaagcgaacccaccatcgtctcaccc

catccgtcgcgcttccccgcatgccacagttcatcttcaccggaatcgctgcttcctgccttcctcgacc

gctcgtcatcgtcagcgcctcagcggtgctgcagcggctgctgctcgttctctcctctctgcctccctgg

tamgtactcatctccttgaccctctcctccctttcatgctgtcaatagttatagtggatggatccatagg

tctccctcctcatgtgctctatctaattagccttcccatgatttggtaggtgagataatgaacaaktttt

tttattaaaaatccagcagctatgaacaccaccggaggaagttctagtgcaaatgcatctagaccaatac

tgagaaaaatctggagtgcctcaaccaattgatatggacaagtttcctcggatacagtaaaaggaakaga

atggcagttatcatcccaacatgaagacataagaaaatattagacacaatcactaatgacgtgattgtcg

atcgtttccataaaatgaaacaccgtcgtgaaaagggtaaaaaggatgccatgtactatatttataaacc

ttatttcttatctatatatactactttcagtaatatttatatggcttgtgttaactaataattatgttgt

ttctatagacaatgacattatttgaaaggcatatctatatattactattaaagataatgtaatatctcag

agtttttagaagctgcaataaggtgagtttcacttggctttctaactgttctatgagttatatagatgca

tgcccccagtcattttttgtcctgcgtccgcccctg

>Os_hAT-N38 Oryza sativa

tagggatgcaagtgggtagtcccgctacccgcataaaaacccgcttgctagttcatttctcacatggtag

tacaaaatttagaagaaaaaatgaactagaagtgagataagcgggctaaaaaaacccgcttgcccgcccc

gcttgcatcccta

>Os_hAT-N39 Oryza sativa

taggggtggtaatgggcccaacattttagcctacaaaatttgagggctgggtctaaaaagggttgggctg

aaatttaatagatttttgagctaaaaatatttaagggctaagttgggttgtgaaagaacccatgggcctt

gacccattaccaccccta

>Os_hAT-N3B Oryza sativa

taggggtgaaaacggagcggattcggacggataatagctataccatatcctaaaccattttttttaagcg

gatgcggagcgggtgcggatgacgtgcggatgcggatgcggagcggattatttcggacgtcggattcggt

gcggagtcggtgcggactcggttcggaaacggacaaaaagttgtcggatgtcaagtttgtattcaatcca

tacaacaagagggcggcaatacaattagccgcaatacgatggcaaaacaacatatgaacatgatctcagc

tctctaggaaaaaccaaccaaaacacatcaataatataattttgcttaatataacacatccatcaaaaac

ataatataagtgttcagttcagcagttcactgcgttgcttaaaatttaaaaatatagtgttcagttcagt

agttcactcatcagtacgacaggcatgaggcaatgaggcatacagcaattaggcatacagcacacagcca

cacaacacagtagcacactgcatacagcacacaacacactagttccatacagcacactgctacagcaaca

gcacacaacacactgtcggcggactagaaggaggagcggcggcggtggcgccagcaggctgcagcggcgg

cgcggcgcacctatggtgccatgcggcggcgcgtgtgggagcaggagaagggaccgagagaagagtcaca

gagagagagatttaggggaggattggattaggttacactagatgggctagatgggccgcccgatgtaaaa

tgggctaaatagagttgttgtgtggcctgaaagttgcggattatccgccaaacagtacatcggataatcc

gattaaaatatcggataatccgcatccgtcggatatcgttgataccatatccgcatccgcatccgcatcc

ggccggatatctaaaaatccttaccatatcctcacatcggacggattcggagcggatcggatcggataat

atccgctccgttttcaccccta

>Os_hAT-N3C Oryza sativa

taggggtgaaaacggagcggattcggacggataatggtcataccatatcctataccattttttgtaaagc

ggatgcggagcgggtgcggatgacatgcagatgcggatacggagcggattatttcggacgtcggattcgg

tgtggagttggcgcggagtcggttcggaaacggaataatattatcggatgttaagtttgtattcaatcca

tacaacaaaagggcagcaatacaatgagcagcaatacgatggcaaaacaacatctaaacatgatgtcaac

tctctaggaaaatacatccaaaacacatccataatataaacaaccaaaaacataatataagtcctcactt

cactgcattgcttaaaaataaaaggcaaatgccataacatcaccatcaggagtaaagacccaacaaatta

acatgttcaatagtccaagatcaagatgacagtacgctaaatctcatctctaaattaatcaaattagaca

ttgctactgacttaaattacctaaagcaaagctcatctctaaatttctaaatcccaaatctcatctctaa

atttctaaatcccaaaattgaaaattcaaattagagaaagaagtcaaagaacacaatacttaatatggag

ccggcgggaggcgacggtggctagcaacggggagtccgggaggtagaggcgctggccgctggagagtggc

gacggtggatggcacacagcaagcgggaggatggggacgacaaggactggatctggaggagaggcgacgt

cgcaggaggcactggccgctggcgagtggcaatggtcgctggatgcgctggcgctggccgctggagagac

ggcgtcacaggaggcgctggccgctggaggagaggcggcgtcgcaggatggcacatggcaggcaggaggc

gctgcgacgggcagttgatggggtagtcgcggcgctgcggcggtggcgtcgcgcatgtgcggcgccggga

cggggactagcctgtgcggcgcacgtgtgcgtcgggactgtggcgcaatggtggcgccgacgcgcgtgag

agtgggaggagagccggcgtccgacagtgggggattaggattaggtcaacactcaacataaatgggctag

atgggctggcctattaaaatgggctcaattgtgtagctttttggcctgaaacatgcggattatccgccaa

ctcatcattcggataatccgattaaaaaatcggataatacgcatccaccggatatcgatgataccatatc

cgcatccgcatctacatgataaaatccgcatctgacggatatctgaaaactcttaccatatcctcactcc

agtcagattcggagcggatcggatcggataatatccgctccgttttcaccccta

>Os_hAT-N3D Oryza sativa

taggggtgaaaacggatcggattcggacggatagtagtcataccatatcctataccatattttttaatcg

gattcggaggcggtgcggataatgtgcggatgcggatgcggagcggattatttcggatgtcggaaatggt

gcggagtcggtgcggactcggctcggaaacggatgaaaataatcggatgtgatatgtatatacaatcgat

ataacatctgatcatctgtacaatgagcaacaatgaggtaccaatatcacaattcacatgactcatgaaa

gcaccgatcacatcataatataatgtcaagttgttaacattgacttgtcttctgtccaaaagcatgtaat

cgacacatacagtattgacatcacgatatatcgcattcacataacacgtaagacatagaagttccacaaa

tgttagaaaaaaacagtatcacatgacataggtgatagaacattggaggtccacaaatgatgtccaattc

tcatacagtactactaataagttgcatattgcatatgacataataacacatatgcagctaaggtagtcag

ggcgtcaggcggcggcgtgggaggactggaagaagagaagtctcgatgaaaaaattggttgacgcgacgc

cgacggagtcacggactgaactcctggatagattgatggataaggtggacctggatgggctgaatcttta

cacgatgaaaatgctcatgtcaaatgggccaaattgagtggttatatgggctgtgaaattcggattatcc

gccaaaacgtttatcggataatccgcttaaaatagcggataatccgtatccgccggatattaccgatacc

atatccgcatccgcatccgcatgcaactatccgcatccgcatccgcatccggccggatatctaaaaaccc

ttaccatatcctcacttcggacggattcggagcggatcggatcggataatatccgctccgttttcacccc

ta

>Os_hAT-N3E Oryza sativa

taggggtgaaaacggagcggattcggacggataatagctataccatatcctaaaccatattttttaagcg

gatgcggagcgggtgcggatgacgtgcggatgcggatgcggagcggattatttcggacgtcggattcggt

gcggagtcggtgcggactcggttcggaaacggacaaaaagttatcggatgtcaagtttgtattcaatcca

tacaacaagagggcggcaatacaattagccccaatacgatggcaaaacatcattagttcactgcgttcag

ttcaatagtaatagtataactataagtgaataagtgttcggttcaatagttcactagtcaaaataatcca

agatacatccatgatccatcaaaaacataatataagtgttcagttcaatagttcaccggtcactacgttg

cttaaaatgtaaaaataaaagtcatcggcatgaggcaagccatacaggcatacagcaacagcaagcatag

agcagtagagcacccctatgcagtgctacacgcagcatactgcatacaggcatacagcagtagtagtaca

ggcgtacagcacaaaacacacagcagtgagcggcggcgctagcagcctcctgcagccggcggcggcgcta

gcagcctgcagccggcggcggcgagccgaggaggagcggctggcgcgtgttggggaggaggatcggcctg

gcgcgtgttggggaggagcggccaccgcgcagggtcgccgtcgcgccgtgccgccgcgctactctctccg

actcctcgactgacggctgagggacggcacagagagagagccagagacggatctgggggatgaggagagg

attaggttaggccgggttgggctattgggctagctaggtgggcctgaaagttgttgtgtggcctgaaatt

tgcggattatccgccaaacagtacatcggataatccgattaaaatatcggataatccgcatccgtcggat

atcgttcataccacatccgcatcccatccgcatgacaaaatccgcatccgcatccgcatccggccggata

tctaaaaacccttaccatatcctcacatcggacggattcggagcggatcggatcggataatatccgctcc

gttttcaccccta

>Os_hAT-N3 Oryza sativa

taggggtgaaaacggagcggattcggacggatagtggtcataccatatcctaaaccatattttttaagcg

gattcggagcgggtgcggatagtgtacggatgcggatgcggagcggattgtttcggatgtcggaaatggt

gcggagtcggtgcggaatcggttcggaaacggattaaatttatcggatgttacgtgtatatgcaatcaat

gcaacatcgagttagcaatccaaggaacaataatacaataatcaataaatgacccatcgtaataattata

tgattaagtgctaaacaacatgaatacatgatgtccataatataaaaaatatagctacgtgagtgtgaca

tgagacgataaaagccttagactaaaaaatctatgttaattgatgaattagggcattggatgtaccaact

tatgttatatgcaatagatagagtggttatatatgttgataatttcggattatccggcaaacggcttatc

ggataatccgacaaaaatgtcggataatccgcatccgccggattttagagataccatatccgcatccgca

tccgcctcatactatccgcatccgcatccgtatccgccggatttctaaaagcccataccatatcctcgtt

tcggacggattcggagcggatcggatcggatattatccgatccgttttcaccccta

>Os_hAT-N40 Oryza sativa

ggggcggacccaggactggcccaaggcccgggccggcccatggagattttggcccaacacgatgtttaca

tggttgaaaggaggaatagacttttcctgtaaaatgcatatatctcaatgggcctccagtcctaggcccc

gggccgcggcccaggtggcccggggcctgggtccgcccc

>Os_hAT-N41B Oryza sativa

cagggccggctctgggctacggcaagaagtgcgatggcctagggcccaggaaaagtaggggcccagaaga

cctaggtatgtattaggtgtaccatatacatgtatacaagtttctgatcgaatcgtcgggcatacgcgac

cgtccgagttccagtcttctcgtctcctctcctctaccggcggcagtctccgccaatccgcctccacgcc

ctgcgcctccacggcgcctctgcaagttcgcccacgctgggccgccggcgatcccttcagcattcagccc

ttcacagctttgtgcctttgtctatcgactgttcgccctggacaccgtccgaccggcgaccgctgacaca

acgtgaatctgcctacttccagagtgtctgcaggtctcagtttacaggtacttgcgtactgctgtgctgc

aaccatattgtccatactccattgaattgaactagcgaagtattaatcagtcatagtaactagtaaatac

taattaaagatcagtttcatctatacgtttaggtttgcaatgttacctaaaaagcatttatccttttaac

tgtgcctgtgatagtagcctcagctgaaagaagcttctcaaaattaaagttattgaaaaactatttgaga

tcaaccatgttacaagaaagattaaatggcttggctatgtgttgcattgagaaaaatgtcttggacaata

ttgatctcgataccatcattaatgattttgcatcaagaaatgcccgaaggagtatttttttatgagaagc

aaatggatgtctgtcatcttatcttttgtcaagtattattaatgaagtccctggagcttgatccttagtc

aagatttcaaggtaaccatagtaattccattttaatgctcttttggtatttttagcaacatatatagagg

atattatcaatatttattttcagcgtgattagcggatgaatgctttaaaagtatactttaaaaatccaaa

gtgcatatgtgtatatatagagagagtgtatgcctaagggcccatgtcatggagttggcctagggcccta

aaatatgtagagccggccctg

>Os_hAT-N41C Oryza sativa

cagggccggctctggagcagggccataagggcgatggcctagggcccagccaaaatgggggccccgatcc

aggtatatatcctgtataggtatttagcccaatagcttagaattgatccttctcactgattagaattgat

gatccaatccatcgatccttctcactgatcaggtgatgatccaatccatgatctctgattagaattgatc

tgttcttctaatttattttttgttccccattaaattacccaagattagggacttagggttctaccgttct

gggttcaaccgttcaagggatcaatacaagtatacaaccattcagtgcaccataagtccataagctaatt

aaattaaggtatatggtggattagttttcattaggcctaggcccttgattatatagatttttccctcact

gattttaggtcatagtactttgtttctactgtgtttttttaagttgtcaatcatactatcgatattattt

atcgagaacaaattactatattgcaacttgcaaactgcttgatttattatttaccatgttagaaatgttt

atttmtgttactatagtataggtgctttatagagtatatattacaagttaaaaaaatagccttaacttct

gaagggcctatagtgtgaagttcgcctagggccctcgaaaatgttgagccggccctg

>Os_hAT-N41 Oryza sativa

caggcccggctctggggcagggcaaagagggcgacggcctagggcccgtcctggctaggggcccatgcct

aaatgtatgagtactgtatgagccaaagtggtattttaagattagtgaataattccatactatctactac

tcctggttatctactattatctacataatactgattagtgaattaataaagattctagattcaacaatca

aaattgtatgtatagatagattcttgttgataatatatatttcctttataattttaggtgctgaaaagtt

gtaattttactcaagaagcatttttatggtgctaagtaaatgggaaaaataaacaagatcagtttaagtg

ttgatatttttctatttctaagaacatatatatgaaatgttatcaatgttcatttctctctatgattaga

tttgaaaacacactttgaaaatgaaggggttgttcagattgtagccaaaataaatcttaccaaattttgg

caatgccaaaattttgacaatttagcaatattgccaagttttggcaggatttcttatgtatttactaaag

tttggtaaaaaaactaaatgaatgcacatctttgacaactttaccaaaaattgatatggtttgaaatgac

atcaatctgaacaacccaaaaaatgtgaatatacatataacacgtctctctgtaggggagagagggttaa

gggtcatgttgtcaagtttgccctagggccctcaaaattgtagagccggccctg

>Os_hAT-N42B Oryza sativa

caggtttcccttaccaccggtaaccgcgcttaccgcgggggtacggtaggggttaccggtggtagggttt

gatgaattttgcccaaattcaaaattctgaaaaaaaattaaaaaaatcatggatatagttcttgatttgt

agtggcagatggtgaagaaattttttgaaaaagagtaagataaattcaaatttgagggcaaaaccgaaag

tacattagaaaatgaaaaaaaaaaaataatgtaatgggccttagggttgcactttttatccggcccatta

aggcccataagtggagaaaatatctcctgggtcctctcatctctctctatcgctctctctttggcacatg

ctttcaaatgtgaggttgttgaattgttgttgttgaattattatgctacgaaatgttgaattgcttcgaa

gtccataatatagaaaaggtcataccgaatttttgtattttacacccaaaagttaggtttgcaagtatcc

tatatattcttctaattatccccaatttctacacacatttaagtgtttttttaaatttttttaagagtgt

ttctgttctgctacacaattggcggtaaccgcgcgaatatcgcggttaccgcgccgaaaccgcgaaaccg

ccgatttttgaattcaaatatttgaggacgaatttcacgtggttttcggcggttaccgcgtatgctcggg

gggggggggcaataatcccccctaaacggtaagggaaaccctg

>Os_hAT-N42 Oryza sativa

cagggtttcccttaccgccggtaactgcgcggttaccgcggggtacggtaatggttaccggcggtagggt

ttggggaattttgaccaaattcaaaatttccaaaaaaaaaaaaaaaaaaaaaaaatcatggatgtagttc

ttgatttttagtggcagatggtgaagaaattttttgaaaaagagtaagataaaatcaaatttgagggcaa

aaccgataatagtttagaaaatggaaaaaaaagaaaaaaagtattgggccttaagttgcactgtgcaacc

ggcccattaaggcccaatagaggagaaaatatctccataatatagaaaaggtcatgccaatttttgtatt

ttacatccaaaagttaggtttgcaagtatcctatatattcttctaattatccctaatttctacacacatt

taagtggtttttcaatttttttagcattgttctttgttcagctacacaactcacggttaccgcgcaaata

tcgcggttaccgcgtcaaaccgtgtggtaaccgcaccaaaccgcgaaaccgccaaatttttgaattcaaa

tgtttgccgacgaaattcacacggttttcagcggttaccgcggtaaccgccttaccgcgggggggtgcga

taacccccctccaaacggtaagggaaaccctg

>Os_hAT-N43B Oryza sativa

ttaaaactagctaggactatttctttatataactggtgacccacgtatcatcaatacttaagaaaaaaat

taagagattgtgtcgacaacaaattcctcataccaccagcaactgctaactgccactctacgcaccgcca

tcaccgctctttctccatgagccaccatcgttcctcctctttgcctttacatgcgcagccttaacctcta

gtaaccgacaagtctttttctcctcccctatcgtgatgcacactattgttatggttgcagttgaggtcgg

ggatgtcgggcttgcagggactttgggaggcaggagctgacccaacaaagagcgaaggagctatatccgg

gctccttcttgctagatctatcggttatccaactatcagaggaaaaaagaaaatggagagagagagagag

agttaatgtgcggagtgattgctgagtcaccggacttggagatggatagaataaggtggtccaaattgaa

atgtaaagttagctattgtgtaaatgaaaagaatggttaaagaataacattatatgaaaaattttaagaa

tttttaagaatacatagtatggactgtaatttactccctttgtcccacaaaaaacgaatctagaactggt

gtggcatattctagtacaacaaatctgaacatatgcatgtctagattcgttttactaggatgtgtcacat

ccagtcctaggttggttttttatgggacggagggagtactttttttttctagcacaattaaagataattg

tccattacctgatagtctcgtgatatgctaagtatgatattttttctctagcacaattaaagataattgt

ccattacccgatagtcttgtgatatgctaagtatggttgggccggtgtacttagctataactaataatct

catgatatgttgatgcatgacgatacaaacttctactaacacgggctgattaattatggttagatagatt

cctctaaaagatgataaaaataataccatcatttttgctcaataataaagcattcgactatggtcttctt

ttctcattgcattatatatgatgactacattagtacgtatttccgtaattctttcatattgttttttatt

ttctacatatatgttaatttggactaatatttttctcataattaaatagataataaatcatttttccata

tttctcctataaaatgagaatagggcattttataatttgaaaattatattataagtgtatgtaatttaat

gtgtaagatatggacttatagtataagatttaacttttttctaaaaatgccgcatcatagtagataggta

gttaaccgaacaaccataaaaaacagagacaatccattgtgtgtcactgattttgtccttgctctacaat

atgtcattgggtttgtcaagttctaatatggcatcgtattttgcttaacttctataatataccattgtcg

tctagttagcctccgttagcattgtacaattttatcatacagattgtaaaacttacaaagtttgagatgg

gcaacttctctgcctagtttataatatgatatttcggaattttcggccaaattttggaaattttttgtcc

tagggatatttcaatcattaggacaaaattatacagtactaacggaatctaaatggacggcgatggtata

ttatagaatttaagcaaaatacaatgacatactgtagaacttaacaaactcagtggcatattatagaaca

aggacaaagtcagcggcacacgatggattctctcttaaaaaataatctagttaaattgtttcttttttat

tcaaaacaaaacatcttttacctaaaatatgttcagagaacttcgaaagaagtaagaaaaagaggaggtt

agtcactagcccaaacacatgagggtggttgctcacattcatggtatggtatatactcacattaatcgat

aatatactaatattattaaaattgttctttgtagtaaatggtgtgaaactaaaaactttaaccttatatt

ctattcttctgttgttccatcttcttcttgttttctcattcttatttatttttaataataatattgctac

atacaaaccagcactattacttgcacatgaatactactccctccgtccctaaatataagagattttggtt

agatgtgacacattctagtacaatgaatctagacataaagcctgtccagattcgctgtactaggatacgt

cacatccaaccaaaatcccttatatttaggaacggagggagtagcagctagctcatgtgtgtgtgctccg

taatatatattatctatgtttttacatgaggaaataataactattatctttgttaatgatattgtattta

taaaaggtacctgactagttttttttaagagaaagatatttttgctaaacataatctagaatgctataaa

agttaataatattaaacgatatagtcatataattttttttggatgaactagtgatcaacacttgttatga

atccaatgatcataatgcactatatatattgttcaagtgagttgtgacattcactatttactagttacat

accccattcctaattgatcatcatatgcattacaataagatctacaaataaaaataagatttagtttatt

tcatttagtctggttgtttatgatgtattggattattttacattgatatctttaaaaataatattgtata

acaattaaataatttattgttttataaaaaaattagtttatcgtacacccatttataaaatcctacatcc

gacactg

>Os_hAT-N43 Oryza sativa

caggggcggatccaacttgggacatgggggttcagttgaacccccaaacttttgggtgaacaaacaaact

gttattacctgaattatttaagagaggtttaaggctatcaaattaaggaggaggagaagatctagaagag

aagctagtagggttcacgaacgcaagcaaattccagtcccggtggggtgggagagagagagagagagaga

tgaatcagaaagaggacgcaccaggatcccctcccctccgctgcgatggagatcgccgcccggccctccc

ctgttcagccgcgactcgtcttggcggcggcgcaaggaggcaaggagcggaggagacaacgagaggtact

cgactactctgcccttatctcaatatttttacttaccactccctccgtcctaattaagtatagttttgca

aggttcacatccaacgtttaacattttatcttatttaaaaatttgaaaacttttcaaagacggacggtca

aaagtttaacacggattttcacggctgcacttatttgggacaaggtagtattttaaaactagctagtgtt

atttctttatataactggtgacccatgtatcatcaatacttaggaaaaaattaaacgattgtgtcgacaa

caaattcctcataccaccagcaaccgctaactgccactctacgcaccgccatcactgctctttctccatg

aaccaccatcgttcctctcctttgcctttacatgcgcagccttaacctctagtaaccgacaagtcttttt

ctcctcccctatcgtgatgcgcactattgttatcgttgccattgaggtcggagatgtcgggcttgcaggg

actttggaaggcaggagctgacccgacgaagagcgaaggcgctatatccgggctctttcttgctagatct

atcggttatccaactgtcgtatggataaagaaaatggagagagagaaaaagagagagagagttaatgtgc

gaagtgattactgagtcaccggacttggagatggatagaatatggtggtccaaattaaaatgtaaagtta

gctattgtgtaaatgaaaatggttaaaggataccattatatgaaaacttttaagaatttttaagaataca

tagtatggattgtaatttacttcctttgtcaaaaaaaaacgaatgtagaactggtgtggcacattctagt

acaacaaatctgaacatatgtatgtctagattcgttttactaggatgtgtcacatccaatcctaggttag

ttttttatgggacgaagggagtacttttttttttctctagcacaattaaagataattgtccattacccga

tagtctcgtgatatgctaagtatgattacaccgctgtacttctctataactaataatctcatgatatgtt

gatacatgacgatacagacttctactaacacaggctgattaactatggttagatagattccgctaaaaga

tgataaaaataataccatcatttttgctcaataataaagcattcgactcttttctcattgtattatactg

cctctgttttttaatagatgatgccgttgactttttctcacatgtttgactattcgtcatattcaaaaat

tttatgcgaatgtataagatataaatcacacttaaagtactatgagtgataaaacaactcataacaaaat

taattataattatgtaaattttttgaataagacgagagatcaaacatgtgagaaaaagtcaacggcgtca

tctattaaaaaacggaggtagtatatgataactacattagtacgtatttctgtaattctttcatattgtt

ttttattttctacatatatgttaatttggactaatatatttctcataattaaatagataatacatcattt

ttccatatttctcctataaaatagaatagggcattttataatttgaaaattatattataagtgtatgtaa

tttaatgtgtaagatatggacttatagtataagatttaactttttctaaaaatgaatgccgcatcatagt

agataggtagttaactgaacaaccataaaaaatagagacaatccattgtgtgtcactgattttgtccctg

ctctacaatatgtcattgggtttgtcaagttctaatatggcatcgtattttgcttaacttctataatata

ccatcgtcgtctagttagcctccgttagcattgtacaattttatcatacagattgtaaaacttaaagagt

ttgagatgggcaacttctctgcctagtttataatatgatatttcggaattttcggccaaattttggaaat

ttttgtcctagggatatttcaatcattaggacaaaattatacagtactaacggaatctaaatggacggcg

atggtatattatagaatttaagcaaaatacaatgacatgctgtagaacttaacaaactcagtggcatatt

atagaacaaggacaaagtcagcggcacacaatggattctctctaaaaataatctagttaaattgtttctt

ttttatccaaaacaaaacatcttttacctaaatatgttcagagaacttcgaaagaagtaagaaaaagagg

aggttagtcactagcccaaacacatgagggtggttgctctcacatccatggtatggtatatactcacatt

aatcgataatatactaatattattaaaattgttctttgtagtgaatggtgtgaaactaaaaactttaacc

ttttatattcttctgttgttccatcttcttctttttttctcattcttatttatttttgataataatattg

ctacagacaacactattacttgcacatgaatactaagcagctagctcatgtgtgtgtgctccgtaatata

tattacctatgtttttacatgaggaataataactattatctttgttaatgatattgtatttataaaaggt

acctgactagttttttttaagagaaagatatttttgctaaatataatctagaatgctataaaggttaata

atattaaacgatatagtcatataattttttttggataaactagtgatcaacacttgttatgaatccaatg

atcataatgcactatatatattgttcaagtgagttgtgacattcactatttactagttacatgcctatgc

attacaatgagatctacatataaaaataagatttagtttatttctagtctggtttttatgatgtattgga

tttttttacatttctttagaaataatattgtataacaattaaataatttattgttttataaataaattag

tttatcgtacccccatctataaaatcctacgtccgccactg

>Os_hAT-N44 Oryza sativa

cagtggcggagctagaaagatttttgagcccaggcaaacttcaatataactagtatttgctatagaaatt

tcatcatcaatttactcatcaatattaaaatttaatggagttttcgccggcgagcccaggcggccgcccg

ggctagccgggcggtggctccgcccctg

>Os_hAT-N45 Oryza sativa

catagtacaaaaaaaccggaccggaccggcggttgaaccagaaaaaaccggaaccggcgaccttggcggc

tcggttcaacaaaaagaccgcccctgcaatcgaaccggtaaaaaaccggtgaaccgggcggtttttatgg

gaaccggtgaaccggtagcggttttttaagaatcgaaccggtttttaatttgtccatggcagtatgacat

tatgaatttattatgttaccacactacatttatttttttttatgttgtgaccatataataatagatatac

tagtttatttatgcatattttcaccaatttgtgatgattctatatttaaaataatgcaaatcaaatataa

tattaataaataaaaaaaccggcggttcgaccggtgacggaaccggtgaaccgacggccagagcgggtcg

acctccggtccggttttttttactatg

>Os_hAT-N46B Oryza sativa

caggggcgaagccaggataaatggtcaccggggtcagtattaaccagggtttatgtttttgaccaaaacc

aaccaaaaatcatgtaattttgatgtttcgacgtgggacgaaaagtatattctgagtgaaattttgaatg

ttttttgagaattcacggcaaaactatatcaaaacttattaaaatttggtgaaaaatgtcatatcgctac

attggaatccaacactaaatcggaaatgtcatctagtttgtaattaatgatctaagactatacataatac

aagacaaaattaatgacaatacattatgataaaatgaaatgtctacaaatttgttaaaaactatacatta

ttaatttgtaaaagacaccaaatgcaccaaagattatcaaaaccactaatttcttgcctttcgttctgct

agaaataagatgatataagatgtcaagatgtgcaacgccggtgcgggacaaagtgctcgaaggaatacga

tgacgcaacaagagaatttgtggagttatttttggacccaaatctcaatcgtatatgtatattgggctga

cgtataaatcataagtgctagctaattttctatttgttgtttgttagtatcgttcctagtcgctagttgg

gctttgacggaacaatcagcaatatcaccggggtctagtttttctcttcgctggggtcatagatggtaaa

ataaagaggggtacgagaggttcgcaaaacatcgtcggggtcaactgacccctatgaatggctgtagctt

cgcccctg

>Os_hAT-N46 Oryza sativa

caggggcgaagccaggataaatgatcgtcggggtcattactaaccagggtctatgttttcaaccaaaacg

gattgaaaacaacgcaatttcgatgttccaacttgggacaaaagtagattttgagtgaaatttggaatag

tttttcaagaattcaaatttgaaattgtaaaattaaaccaaattaataaaaaaaaatatcaaattttggt

aaaaaaaaagccatattgctatatttggaatctaattttaaatcgaattggtgaaccctgatacaatctg

ggtgtgttcagacctagctacgggatctgaacccttccctcccagcacgcaaaatagagcgaggttacca

catgattaattaagcattattagcttataaaaaacttgaaagacaatagattaatatggtttttaaaaca

atttcctatagaaattattttgctagggaatttgaaaagttatgagccgctggccttatgcatggtctca

aatctcaaaaactcaaagtatatgcatattcggctgacgtataatcataaatgctaggtaattttctatt

tgctgctgctagtatcgtttctagttgtcaattggaatttaacacaacaatcaacaattaatgtcaccgg

ggtctaatatttgttttcaccggggtcatagctggtaaaataaagaggatatgagaggttaaaataaaat

catcggggtctactgaccccggtaattgactgtaccttcgcccctg

>Os_hAT-N47 Oryza sativa

aggggcggatccagcgtgggtgctggggggactcgagtcccccctaccccgctggatcaccattgataga

aggggagatgaaaggggaagaatagggagaaaatgaagagtaggggggctgaagaaagaaggagatgagt

ccccccttaaatcttgttctggatccgcccct

>Os_hAT-N48 Oryza sativa

cagtggcgaagccaggatttagtgataggggtgctggccacgagatgtaagaactcgccagcgcctcggt

agacaatggtgtggatcttgatggctcagttcatgctgagtggcagattggcctcacctcacctcttgtt

cgctctaatatgcgtagtcgcccctcgtataattatattttgaattttgtggttaatttgcactaatatg

taaataaattgattgttgatatgactttggatatatttcaacacattcttctagattcatgtaaaaaact

gaacttattttaggatggagggtgtatgtccgaatgcacattaatataacataaaagaatcattttttaa

aattaggcaactaaatagcaccaaatttactgaatacatgaatgacaagacatcatttctattcatccgt

tttagttttggagacttgcaaccacatcgcttagttacattaattaactcaggtaatcacgtaaagctaa

aatctattggtcagttatgtataggctcaacttgcaactaactaatgagttaattgtatggattagatgg

ctaatcaccggggggttaatttgcagcgcatagggatggggggtgccaggcccaccccggcacccccggt

ggcttcgccactg

>Os_hAT-N49 Oryza sativa

caggggcgaagccaggatttatggttaggggggtgtgggcagggcgaacaagtcataagtcagagtaagc

aataccgacgataaaacaagacatcatgtgttctgccataaaaaagcttgaatttagctctttagttgta

tcattggatgtttgacagtttgagacttgagacaccatgctcgacagcgagacgcaatgggaggacggag

agacgtgtgaagccgaagcaatgaagacgaggagttgatttgcgttgtctctccgtctctttcttattaa

ttaattttctatatcatgtttattgataatggactaattaacatgctaatgcgctagttagtacagcagg

gagggggtcggataggggggtgttggccccctccggcaccccccgtagctgcgcccctg

>Os_hAT-N4 Oryza sativa

cagtggcgaagctatattgtgtttggggggtgcacatgaacccccaaattcctaatttctcacttaaaaa

cactagaatgtacagtgttgtactgaaattttctagattctcaatggtagtgcaccccccttgttttgat

gctagcttcgcccctg

>Os_hAT-N50 Oryza sativa

caggggcgggcccaggtttttcaacttgggtattcgaaattgtaagagagagaaaaaattttccaaccca

aggtctaatagttgtataatatgtaacggtataaacatgatttcataaagtcataaatggatagaattga

ccataaattgaatgcgttaagcatattagagtacaatactaaactattaattgacaaaacatatcaaatt

tatagagcatacaaaatggcaaaatgataagaaatcttacaatttagagaagaactttccgtttcttgat

attttgaaaatgattgatgatgtctttattctcaacttgcaagaagaaaactctttaaatgaatgtaacc

aagtaaccatgggaccctttttttctcttcatgtttcaaacctacaaacaacaatatcaaagacaaagag

caattgagcaaagcagcacaggttcagtggttcctaaatcctaatctagtaatctagatagtcattcact

tcacaaactatgaagctaaaataatcaatacacttcaaacaaaaagagttcaccgagaataagaatatta

gtctcggcgactcaccgttgcagtgcacggttgctgtgatgctgaggctcgaggacgggaggccgaggcc

gcgaggcgcgcaggcggccgccgggcgcggggcgctgccgcgccggccaggcgcggcggggaggcgccga

ggcggcgacggggcggcgacacgggcagccggcgcgacgcggagagcgggcggtggcctgtgggccgccg

gcgaggcgccgccgcggccgcgccgaccgggcggcgggcgccggcgacggcgaggcgcgcgcggcgcgcg

caggcggctagcgggctgggcagcgcgaccggcgacggcgaggcgcgcgggcaggcggctgggctggcgc

ggcgacggcgcgcgcgcgtgcgtcggcctgggggctgcggcggctggcgctggcggctggggggaatggg

atgggaagggaggtgaggagaagagatagggttagaaaggaagtgggcttttttttgctattgggccaat

gggcctgaaatggtgggtggtgggagggttcagtttgggcttcttttgagttttgaatgggtataagtat

tttttttttttttcctatatatacatatggaatatatacttcatatatgtattttttgttcaaaaatctt

gggtattcatttgaatacccatgaattgaagtgggcccgcccctg

>Os_hAT-N51 Oryza sativa

cagggtttgcactttcgatttcggccaaaatttcggtgatttcggtaggaccaaaatttacgaaatttcg

tgattttcggcacttttcggaccgaaattttttgaaattttaacatattttgactggatttgaataaact

ttgaccaaattcaacaaacatttgagaaaatccgaaaatttcggtcgatatattgatttgccggtggggt

ccgaaatttcgaaccgaaattgcaatcactg

>Os_hAT-N52 Oryza sativa

cagggccgtccctaggggcgtgcggccagcgcgcccgcacagggcccccaattttcaggggcccctaggc

ccaaaacttcaacactagatatacaactcaataagtaaattggtagcctaactagtcccgaaaatctgtt

atttttcttgtcgtttcggcttcgattacgcctttgccggttcgcacagttgcgccggttcgcaatcttg

aatcgcctcccactgtggacctcgccaatcgccaatcgcaacgaacgagaggagtaaacgacgcctcacc

tctttctctcgtgcggtcgtgccgcctgacgcctgccgcgcccctgacctaattctttctggtaatttct

aatccggtaatccctaattttggattatcattttaggccattctagattgctatgaaatatattacggag

tataaattattttcatattatatcatcaattaagtttttatccttcaactttgcacagggcccccgaatt

tgacgggacggccctg

>Os_hAT-N53 Oryza sativa

cagggccgtgcacaggggcgtgcggcccgtgcgaccgtacagggcctccaaatttccagggcctcaaaaa

atcagcccaaccgaagaaggcccatgtattcgtgtgaggccagagcgccagaaccccgttcgagtccgcg

cgtatctccgcgtggcgacttcgcgtgcgtggcgcttcgtattctcaactccgagccccgaggccccacg

gcgttttgtattctctttcgttttcgtatctgtgacttattcaattcgtctcgagtctcgacgtctcgtc

tcctccgagcaccaggccgccagccggctcatccaatttcatattttcattcaaccagcgaatagcaact

tagcaagcaaccaacaagcaaggcactaggcagcaagccagcaacagcaacgaacattctccgttttcag

tttgacagtttctgattatgcaaaacaagagttaaaaaatatactattcttgatatatcgcatttgctct

gcagtatatatattattttagctatatttggtacttgatttaggcctcacttagagccttgtacagggcc

tacaaatttgacggcacggccctg

>Os_hAT-N54 Oryza sativa

taggggtggaaacgagccgagccgagccaggctcggctcggctcgtggaagctcgcgaaatgccaggctc

ggcttggctcggctcggttctccaaccgagctgtacattaggctcggctcggctcgtggtctggctcgag

ccggctcgagctggctcgtgagctttgcaatagcagcagttgtattgccagcagcagttgtgttgctttg

caatgacttggttattcagataataactctcaagtccctacaaagataattaggtatattgtatgcagcc

aacatataacaaattgatgggcttttttggttttcggccagaagtctttctattcaaaccgagcctacta

agctcgtgagctgctcgcgagctggctcgggctcggctcggttcacaaacgagctaaaacccaggctcgg

gctcggctcgttcaggattcgagccgagccgagccaggcgagcccgagccgagcttgagccgagttcacg

agcccgagctatttgtccagcccta

>Os_hAT-N55B Oryza sativa

taggggtgaaaacagtacggaatttttccggattccggtcatgttttcggaaacggaatcggccggtcgg

aattttttcggaatttctcggaatcggaatcgaaaacagaaatattttctcggaaacggaatcgaatgat

gataatagcagtttccgtcggaacccggaaacgatcggaaacattccggaaaaattgtcggaatttagtg

aaggttttctggatccgaagttaaaaaatcttgagggcccatagattcaatagtccataattcatcccta

gcttcccagcccaacccaaatcgcggcggcatctacctacttagatctaaccggctaaccctataggaga

gagaccgtcagactcagaactcagacgcgactctcgcgagttggaggcggagcggcggctgcgtgcgtcc

agatcgcggcatcgcggcgccggcgtgccctcacgcccaccgcccgtggcgccggcaccaatcaccaccg

taatcccactgctggtcgcctggccggctggcccccgccatcctcgcccccaccatctcgcgccgccctt

gctcttcgtctgccgcggtggcttccatctcgccagtgccggccggtcgttgttgtctgtttcgccgccg

gccgccgccaccatctcgcacctccgccggcctgctcggtccccgccatcgtcaaggcgccgtcccaccg

cgtctcgccgtttcaccgtcgccaccaccgttcaattgtgccgccgacgacggaacaaccaccacaccga

cgaggcgacgacgacaccagcaccactgcagtctcttcctcaccgccaccaacgccgacggctgaaattc

ctcgcctcgcagcagccagcagcccagcggcagcatctgagcaaccagcagcagcaggtgtatatagtga

ttgactttgcacttttactaatgctctatcatttttttttgaaggctaatggaacggaacaagagacaga

gacagtagaagcggaatttgtctttttgttctaatgaggatggcattgcgccaatcggatgtttgaagaa

aatgttggtcaatttaattttcaagttacatctaatttcaagtttccatttcaaattagtgtactcctgc

tgcacaagtggatgagatatgtttgaaccatgtactgatgtgttttgtgattgaactatgttgttttgtg

atgaactatgtatttatagggtgatgtgtttcttttgtgattgaactaagtactgaaatcactgatggac

tgatgtgcgttgcaactattgctgttctgtgatgattggaagatgaactattgctgttctgtgatgattg

atgaactaagtgttgatggactgatgttctgttgttgttctcaaacccatttcctggagtgataggctga

tagcagtatagcacctgaattgtgtataccttataggcttataccattctgttttgctgtattcttttag

aatatttgttatgcaaattatatgaagttatagaagaatatattttgctgttgaactaaacaattagctt

gatataattgtgttttatgatgaattgttgactgttgagacttggaattggacttgacaagctgtgaggc

tcagtttgaggggtttatgtattccgataaattttcgttaccgtatccgcgccggttcgttttcgctccg

ttttcggtttcgataatattcgtttccgttttcgtttccggggtttccgatttcgattccgattccgaaa

aaaaatatgaaaacgaaaacgataaaggttgtttccgtccgtttccgtaccgttttcaccccta

>Os_hAT-N55 Oryza sativa

tagggatgaaaacggtacggaaactttccggattccggacctattttcgaaaacggaatctgtcggtcgg

aatttttcggaaacggaaacgaattcggaaatattttctcggaaacggaatcgaatatgataagggcagt

ttccgtcggaactcggaatcggtcggaaactttccggaaaatttctcggaatttccggaattataaaggc

ccataccatagtctataattcctccccagcccaacccaattcagcatcctaattcctaagtcactcaatt

cagcccaacccaatacacccaattcaaacttgagaattggacttgacatgattgtgttttgtgatgtaat

gttgaaatttgagacttgagaattggatttgacatagtttaaagcttagtttgaggggtttttgtattcc

gataaattttcgttaccgtatcagcgccggtccgttttcgctccgttttcggtttcgacaatatttgatt

ccgttttcgtatccggggtttccgattccgaaaaaaaatgaaaacgaaaacgataaaggtggtttccgtc

cgttttcgtaccgttttcatcccta

>Os_hAT-N56B Oryza sativa

cagtggcggagccaggatttcagcaaagggtattcaaagttgaaaaaaaatcttacagaactcaaaaaat

tggactggcaaaatttaaagtataaataatagcataacaacatagtggcaatgacatgtttaaaggtaca

aacagtagcacaaaagtaaagatatctaaattataaatggtttgaatgaattacatgacaaatgaaataa

attgtgcaaatcctttgtatctaattttgtgaaatcctttggataaaattgtgcaagcttaaccaagttt

tccttgtcataagcagcaaatgaatcaataggattgaaggacgccatgcaaagaagcaactttgtgttta

cctcatcaaatctatcattaaactctcgaagttgtctatcaatgatgcttataaacataaaacatgtcaa

catggaagcgagggaaattcttgacctttttgaataatcttttagatcttccaaccggcttgtagaagcc

atccatatcaacaactttgatgccatgcttcacacaaaaagaagtggcatatctttcagaaaagattacc

attcatcttcatcatcctcacgcaatagctgcaattgcatctttgtattaattagttcaattgcattacc

aatatcttgatccctcagctgctcagcgtcggcagtcgccgtcgaggcctcaggtgtgaggcgacgaccg

gacgacgcctcggccgctcggcggcgcggcggcggcagcggacggcctcggcggctgggcttcggcgatg

gcgatgctgcgtcgaatcagggacagggagtcagggagatgaggccggtggtgacgcgctaagctgccga

gcgctgagccctcgatgcgccatcggccgtcgcgacgtcgcccgtcgttgcggagtggaggcgtggattg

ggaggagtgagaactggactggggacaccgggacagggagccagcagccagggagatgaggccggtggcg

acgcgccgtcgccgaactgccgagcgccgagccctcaatgcgccgtcgcccgtcggcggggagtggaggc

atggattgggatttgggtggagtgtggacttgggactagggtttagtctctttttgggccagtagcagtg

ggcttattcgctggggcaaggtggggaggaatttacgggctgctgctaataagtgggctttttttcattt

gggctatacatgctgcactttcctctaactttttacgtatatatgcataacatacctatatatataattt

ttttttggtaaaattctatgggcattcacgtgaatacccatgaatcacatgggatccgcccctg

>Os_hAT-N56 Oryza sativa

cagtggcggatccaggatttgagcaaagggtattcaaagttgaaaaaaaaaatcatacagaactcaaaaa

attggactgggaaaatcatacaggagtggtaatgacatgtttaaagccttaaaggtacaaacagtagcac

aatagtcaagatgtctatctccaactgcacacttggggacatatcactatcatgggcattgggtgcttca

agtagtagctgaataggactagaaggggcattttctgggatctgaacttcactctcaatggttaatggtt

ttggatcagaggtggaagctactttctgagacttggttgacctttcccacaaagacttcaaatctattgt

cttcttcttcttcatttttcaaatttcaagacctacagttcaaatttctgactaatttaacttctaagca

atagcaattaagcaatggccaatcgcttggggaatttgacaagaggatttattttaccagtgccagtcca

ctgtccagctgtctagggaccaaggggacgcgccgacgcgggggtgcgtcgccgcaaccggctgccgccg

tgcgccgcagcgccgccgccgtccgtgcgaacctcagctgctcagcgtcgcccgtccccgaggaggcggc

ggcgtcgtcgcaggccacgccgcctccgcagcagcaggcccagggaccagggtccagggagatgaggctg

gtggcgattctggcgacgcgccgagccggcggccggcgccgccgagcgtcgagctctcgatgccccgtcg

cccgtcggtggggatgagtggaggcgcggcggattggggctgggacgacagaaggggtggggactggctg

accgggggatagggactagggttttgttcgtttttgggccagtgggcctattcgttgggccaagatgggg

aggaatgtatggtctgctgcgaataaatgggcttcttttcatttgggctatacatgctgtacttccttct

aatttctcatgtatatatgcataacatacctatatatagttatttttttataaattctatgggtattcag

gtgaatacccatgaatcacatgggatccgcccctg

>Os_hAT-N57 Oryza sativa

cagtggcggatccactgttggggctggttggtgcgccagccccacctctaatccctgcccctaattagtc

attaacaagtctagttagtcattagtgaagaaatttcccggatggcaaggatgcaggctgcctcactgct

tctactgcccaaactgataaaacagaacatactccgagtatacataacaatatattcacagatatttgtg

aatattttaatatagccacaaatcaaaatagagaaagttcttttgatcatatgcgcatttggttttcatt

ggaaaatactttagttgatatatctttatttgttttaatcatattatatttttacactagcaccccctca

atttacatcctggattcgccactg

>Os_hAT-N58B Oryza sativa

cagtggcggacctaggatttgaacccagggtaggcctagccaaaaaaaatccgaatgcaatacaatacaa

tatgattcgatataaaatgtggtaaacgatacggtatgcaactctaaatgattcaatacaagtagaaagt

aacaaataagtttgaatcttacctaaatgacaatgtaaataggatattacaatacttaatactagtcaag

actaagctttacgcttcttcatggtcacgaaagagtgaattatgtcttcctcacttacattggagaagat

atcacgctcaatctcctgacaaccatcaaaccatgcaaaattgtcatatgaaattgaaggggtagtacac

tagtagtatggtttgctcggtctttcgcctctatttcagaaataggaaaatagctgattctacttcatgt

ataactaatcgttccaagaatctctccaaacatgtttgttgatattgttcaatgcctaaatgatagttgt

cattctgtacatctatggctaatacggccaaaaggtaatgacaaaatcagctagtaggtttggaattgct

caccggagtccggagaggcggagagccggagcggagaggcagtggcgcctgggcggctgccggctgggcg

ctggagccgccggccggagagggcgcgctagccggccaggcctggggcacgccggtgactcggcgagggg

cggagggagtcgtggagagtgcgcggccgcggcgcgggcgccggcgcggcgcgtgaaggcagcggcgccg

ccgccacggctgggcgcgcgcctggcgggagccgggaggccgccgcccgccggccggacgcggcgggagg

aacggcggcgcgagtagcggcctcgcgggacggcgggagcgccggagtcgcggctcgcgggagcgtcgtc

gccgtcggtcggtcgggactcgggagcagtggcggaaagggaggggagattgcacgggagagaggagaag

agattagggttttttttgtttgatgggccaggcttttttcgtgggccaagaaaaatgaataccgggtctt

ctctaggccaaattctagggtagtcctaggcctatctggactaccccttgggcccgcccctg

>Os_hAT-N58C Oryza sativa

cagtggcggacccaggatttataccctgggtgtgcccacaaatacaagccctacacattggtctagagca

tatacacaaaaatataggcaaaccgagcattttatacaccaaaatatggagccctgtacatggcctagac

aaagcataatctgaattttctaatgccttgctaaaagaagacaatgcccttgttcctcttcgtattaact

gatttcagaattttcaatttttcaaccaatctctagctctcctttaaaagaaaattcagggagacaaaga

aactaaaaatagtgagagactaaaagtcccaaagagtatatctgtatctgaagttttagatagatgatgc

gaagctggacctgcttgcttgccttggtcaatgtggttgctgctggctgcattccttgcgttgctcacag

actcaccgggcgcgcatcgacgttgcttcgccatgtctctaaatgctagtggctgcggctctcgtctcca

ccgtcgacgctccacactcgccgtccagccaatatcggtgctctggcctgttgactgtcgtgctcttcgg

tgtcaccgccgccgctcgctgaatgtggaacaacgcctgatgccctgaccctagctacctttcacacgat

gccaagtggatacgcgaatgcgaggtgaagcggggaacttgcacgatgcaaagcgcacaacacatcacac

aggcatctatgatgggctggctcatgcgttcatactcattcaaacttttgccccaaactaatacatgcta

tatactataagtttttcgtttttttgggtgtgccggtgcatacccggcacaccccctgggtccgcccctg

>Os_hAT-N58 Oryza sativa

cagtggcggacccaggatttgaacccagggtaggcctagccaaaaaaaattcgaatgcaatacaatatga

ttcgatatataatatggtaaacgatacggtatataactctaaatggttcaatacaagtagaaaataacaa

ataagtttaaatcttacctaaatcaaaatgtaagtttttttttgcaagaagttcacaagcattttttttg

cttcatgcttttgaaaaagcaacgcaatgcttgacttctccatgcctcaatctcctggactcctaacaac

catgaaccatcaaacaatgcaaaattgttatataaaggggtagtacactagtgctgcctgttggtctact

actagaactctagtctctagattctagaagggggagagggactgtggaattgctcaccggagtccggaga

ggcgaagagccggagatggagtggcgccggccgccggccggcctgggcggctgccgactgggcgctgctg

cgctagccgctggcagcgtcggccgtcgggctcgggcagggcgggagccgggagcgggacgccgggacga

cgggagcaggactgtggcctgcgggaggccgccaggcgacggccggacggcgcggcgggacggagagagg

acgcggccgcgcgggcgccggcgcggcgccgccgccacggctgggcgcgtgcgcctggcgggaccgcggg

agccggccagccgggaggagcggcgggggcaggagcgccggagtcgcgccggcggccggcctgccggctc

gcgtcgcgggagcgccgtcgccgtgacgccgtcgagtgggaggggagagagcgsacgccgcacgggggga

gacggccagactggagaggagttagggttttctttgtttgatgggccaggcttttttcgtgggccaaaaa

aatgaatacctggggktctctgggccaaattttagggtagtcctgggcctacctggactaccccttgggc

ccgcccctg

>Os_hAT-N59 Oryza sativa

acccgctacttgttgccccaaaacattgacattgctggtctagtaatttaaattatctatctatgattcg

tattcataggtccatacaaagaggcgcaacaagctacttcatgataggatgagagatttgtctttgtaaa

ttaactccaaactagaaacaagagagacagcaaagatagggatcctttagagaaggaagtaatgatgctg

tgtctaataatgagaatgagttcattaatggtattgtacctctttctaatgagcttgctgaacatgatcc

acaagatggagcatcacagggagaatcatcacaagctcaaggaaaaaggaagaggcatgtgcgccctaga

aagaagaaaatcaagagcgtgcactctttgatgaatgatgttacaatgcagcctggaccttcatcatctg

actcagaagatggtgatggcaatggcgctggagacgtttcagtgggatcctccgattctgataagtctca

tggtgtctctgaatctgattagtggaatgcaatattctaatctacgaacttgttgttcttattttgttaa

ttaatggtcatgttcttgacttcttgtgatgaactatttattatttgtgtcaagaactcaagtggcatgt

ttactgtgaactatgaactgttaaatttataagtatgaactcaagtgccctgtatctgtatgatctagta

tgaactatgaaagtatgagttaggaactatgaactccatgtatgaagtatgaattggtgatgagctttga

attggtgatgaactctatgtgcaatgaaataagtgatatgtcaatttttagctcaaattctatcaaattt

ttattttttctcatattttagaaaacgctaaatgctttagctgtagctatagccggttatagctgctctt

agctgttggagagcacaaccgctaagagccttagcc

>Os_hAT-N5B Oryza sativa

caggggcggatccagaaacaaaaagttggggggactcaacataccgagtacaccgatataaacacataat

ctcagtattaaactatgctaaaatgctattatgagacctttagcacctcctatcttatcaactatgatga

aaaaattgaagggggggcttaggggggtatccatgggttttgtgggtgtaggggggacttgagtcccccc

tgcacccacgttggatccgcccctg

>Os_hAT-N5 Oryza sativa

caggggcggatccaggaacaaaaaattggggggctcaattacacagtttcttcaacctccagccatttag

ggacctttagtttatcattcatggtgaaaaaattgaagggggtgctccgggggtatccatgggtcttgtg

ggtgtaggggggactcgagtcccccctgcacccacgttggatccgcccctg

>Os_hAT-N60 Oryza sativa

caggggcggagctgcagtactatcattggggtcagatgaccccgatggcattcgatcaatcaagtgaatt

gttcagtattttgtagctaatgaccccagcagcaaaagcttaatgaccccggccagcccgctaaactgct

ggagctcaggaaagctgaaacaattgtacagcttctttctttggaagcacaatttgttagctgtgttatg

ctactgtcaatccactactaatgctgcaattagacatatttgatctattaaccaactatttattttagct

atgtgctatgtatatcaatgcattaggcctgtagttttgctaatcaactactagatgttagtgtgttttt

ttaagctactcactccgtctcaaaatatgttacttctaagattcaaatttgctttaaaatatagctaatt

ttctatgaaccatcccatctcaaccaatcacaatattccccgatttaatttttttcacctactttttttt

ccaaccaatcataattatttcacaccctaacattaatctcttaaagagacgtaggtagttgtattttgag

acagatggaataataaacaactttcagaacatacttttatgtctgaaaaaacacatggttgttatgcccg

gttgtatatagtcttaacttttaaatttttggaccttaaaaacctcataaattcttatagaatagacttt

aaacatttatttttagcaaaatgaaacaatcccaacttactcatagctctagctccaaaagaattttgag

ctaaaaatatctagcttcgagaataattagagtttcaaacacgacataaatacaagtaaaagccatgacc

ccagtgactcaaaatcctggctccgcccctg

>Os_hAT-N61 Oryza sativa

caggggcgtcccgggggcccgtgcgggccgtgcgaccgaacagggcccccaaattttaagggcccctaaa

atattaagtatgccctatatataataatattagaggctttaattttgttaatttgcagtccaaatagtag

caaacacaaggcccaaaacgctgcagaaggtgaatcgatccatcttccatcgtactcccgtccaggcgtc

cgcgtcgtcgcgtatcgccgaagtcccgatgccgcagccgcttcgtctcctctccccagttccctccggc

ctccgcttctgcttcatcgctgatgatcggctaatcgccacgcccgtttttaattttactataaaaaatt

tactaggctactagggcccattttaatatttcgcacggggacccgatttggccgggacgcccctg

>Os_hAT-N62 Oryza sativa

cagtggcgggcccagctttttaaacttgggtattcgaaatgggaaggggtaaaaaaaatgtccatacacc

aaaaagccaaaaaatgggcttgtatattatgatacactaataattataatttagagcgtatagaacataa

aattgatcattagttcacacagtgatagaattgatcatcactagaaattgtttaacttatagaaatacta

ataccaaacatatcaaagtgtcaaaatatggaatacaaaaatgatagaaaataataagttaggaagaaaa

cttacaatttagatattaacttttcgacgcttgatattttggaaataattgatgatgtccttgtccttag

cttgcaaaaagaattctctttcaatgaatgtaaccaaacaatcattcaaatactacaaatttgctaatat

ggtatgaactatgaactactgtccagtgtccaatatactctcaattactcaaatactcaaataggcaact

agtcaactacaatcctacaaactaaatgtctaaatcaagcaagcaagcaagcaagcaactacactaaaat

actaaatgtctaaatcaatgaatttttgtgcttgtcccgttgtccggagtttagtcactcactgtgctaa

tgtgcttgtccggaatttgcaggtcaggcagtcgccggcgagcaggatgccgcacggccgcagcggcagc

gcgcagccgccggctgcctcgggcctcggcgcgccggcgcggaacggcggacgccgacctgcaggctgca

gctgtgcagcgcctgctcctgctcggctgacggcctggagcagcctcctgcctggctgcctcctgggccc

ctgcaggctgccgcgccggctgccgcacgcggcacgccgtcgcccgtcggtcacggtgcgcctgccggtc

acgggcttggcgccttggccttggcggctcaccgcctcaccggtcgccgtcgccgccgccgcgccggcgc

cgcgcccgtcccccgcgccgcccgcgcgccgctgcacgccacggccgcgccacacgccgccacggctccc

gccgccggccgccgcacgctgcacgcagcccccgccggccgccgcacgtcacgccgccgcacgcctcctc

ccgtcgccgtgctgtactgctgtgttgtggtctagggtttgcaggtttgggtggccaaggcgccaggcgg

gtggggaattgggggaaaattgcaaatgggctgagatgtggcccaattagaccacaggtagagaggaaag

agggaggggagtggaaaatttaggcccaatagagaagaaatggggaaacaaggcccaatataagcagttt

aagctttctttttatttttttaatacctatatgaactatatagtatatataccttttttttttccaaaaa

tcttgggtatacatctgaatacccatgaatacatagtgggcccgcccctg

>Os_hAT-N63 Oryza sativa

cagtggcgggcccaggatttagaggttgggtattcgaaataggtaggggtaaaacaaaattctttttact

tccagaagtccaataattaattggtataatattacacattactaattgtataatattatagcatatagaa

cacaaaattggccataactttataaattgatagaattgatcataagtccaaattctttaacatatataag

tactaattgttcgacatatgaaagtggcaacatatataacatgtaaaggatagaaaatggtaaaatgata

aaaaaaacttacaatttagaggagaacttttcaatgcttgatattttaaaaatgagtgatgatgtcctca

tcctttaacttgtacaaaaaattctcttccaataaatgtagattgttgctcatcttgcttcttgttttta

tatagttcatcgaagaaaacaccctctctaaattctctaaattcctaacaacagctcatatatagcctca

tactatcatcatatatataaatttaacacactgaaaattaaatattatggttctaagttatctaaattcc

taatatttacagctttatactatcaatctgtcaataaatatacacttaacagttaacactctgaaaaata

aagaagacaggaactgaggaagttgaatccttcggtgctagtgaactattgaactactatagaacaaatc

aatgaaacaagccgtcgccaccggaaaacataactactattgaactacctagaacaaatcaatgcaatga

atcaatgaatcaagcccctcacgcaatccatgccaggccggcgagccgtggtcgttcggccgccgcatcg

cgtacctgtgtggtggcctggcggactggcggcctgcacgcctggccgagccccaacgccgcacggccgc

agccgtcgacgcgccgcctgcacgcctcggcgcctcctgccgccgacgcagccgcgcacgccgcagccgg

aagcctccgccgcagccgccgacgagcacgctgcagactagactgcagtcgccgccgcccgacgcgccgc

ctaggctcctacccgccttggcgccgccttccgccgacgcagccggaagtcgccgccgccgcactgccgc

acgtcctcgccgccacaccgcgcacgtcctcggtcctctggttagggtttggccgatttgggaattaggg

ctttggtttgctgtgcctaggcgaggaggggaaatgaactagttgtggcccaaactgattgtgggctgag

atgaaaggggagggaagtgggaaatttaggcccaatagagaagaaacgatgaaacagggcccaaaatgag

tagttttagcctttttctttaatttcttgaatacatatacgaggtatagagtacatatacttattttttt

tgtccaaaaatcttgggtatacgtctgaatacccttgaatcaatagtgggcccgcccctg

>Os_hAT-N64 Oryza sativa

cagtggcggatctagaaattttttttagcctgggcaaacctaacgatagaaaacaaattaacaagtaaac

acaaatacatatagcattttatcatggatagaagtaaaattgggctattagctaatgactaagtatgata

attagtgctaatctaatggtgcatgtatgcagctaggctaatggctaagcaccatagttagttctaatgc

atggatgcatgcggcggagcccgggcgcctgcccgtgctggccgggccttggctccgccgctg

>Os_hAT-N65 Oryza sativa

cagtggcgattctagcaggtaggctccatcaggctctagcctaccctccaatttttgcgaaactttttta

actagatagtcatcttacaaaccatcgggagaaatcatggcggcgttcgctggggaagaagatgaacagc

ggcagcgcttggcgtcgagaaagaaggccagcgaagcggtacgcacgcacaaaaatagttaggcccaatt

gtggcccagttgactcgtcacttcatggatagcccatctctccgctcgctgattccctcgatctctcgct

tcctcaatcacgcaagccgctaaaagccaccaacctagcgtctagcggcgtgaccggcgacggcgggccg

gcggcgacagcgagcagcatggcgcaggcagaggctgtgcacgggcaggcagaacaggcggcggcgcagc

gccgtagggcgcaccgggcgacgaggggcggcgagggcaagcagctagcagatctggccatcaggtcatc

catccatcaaagggcatcaggacgtcgcagcgggggcaagcagcagcagctagcagagcaagcgtcaagc

cgctagcggtgagtcctcacctcatctctgttttcaacagcagcagctagatgatgggatttacaagatt

tgagcttcatgattcatagttttgatttatccttaggtttgcagtggaggagaagaaaaaaacgttgtta

aattatgcaagcaaatcaatttaatagtacccatccaattccaagtgatgagagcacaaatcaaccaaag

aagcctcaagtacaatttgatcgtaatgaaatttttgctgatcccgcacaaagaatgcctatattttttc

tcctgacataagagatgaagtgaggatggcatatttatcgaatgctccaaatagagcacctgaccatatt

tttgtttggaagacatcagatggtaaggtttcaagtcatttcaacggaattcgaaaattagtgtccaaaa

ggatcctgtgttttgtttctattgttttatgtttaagaaatcagcacatccccgtaaattaactttagtt

cttcgatgttgagcctatcctctatttcagtcctggattcgccactg

>Os_hAT-N66 Oryza sativa

cagtggcggagctggacagaaaatcaaaggggggccagccggcttttgacagttcaaaaaattagcaatt

tctctttttttttcatgcattggcaatacataaaagcgaatagtttgcaaaagtcgacgacaaagatcag

ctacagatgtcagattttgaaatttaggattggtgggtacatcaagttcatagtgacgtaattgacatct

caaattggctctcttgttctaaaaaatcagcaggatagaactttgaagctagagagcacactttatcaat

gttgaatgaactgaatgaatcacttggatccaaagatgtacacaaagtgagaagctctgttgcttgttca

ctgaatctgctatgatatatgggggtaataaaaaattttgtttttattgggaggggccatggcccctgcc

agccccccctcagttccgccactg

>Os_hAT-N67 Oryza sativa

cagggccggccctgggggggggcgagcggtgccgccgatctgggcccccagatcgcggggccccccacgc

ccctccctcccgcgtccctggcccgcagatcgagcgcatgcatgcgcggaagccggaaagcgaagcgccc

cgcggcgaaaagaaaacagaaacgcgcgtgattgccgccgcccacatcccctcctctcctcttcacgcgc

acgcgctcagcgatcggtgaccgggcgatggcgaattggcgaaaagccaaaaatcgaaactgcatcgcgt

gtcgccggcggccggcgccgacgtaattcactccatggccatcaccagctgctcggcaccgacgcaatct

ccctggttgctgctgccagctgctcagcgacgacgcaatctactgcgtcgctgctgctcggcatctccta

gcctccatcgcctgccttcccctcggcaaggttagcaatcagcacaccaatacaccatctccgtagccca

attattaatttaattactgctgtccaatctctcgatatcaaaacataaacatatgtaggccatcccgacg

cacggcgtcgcgggcattagcgcttaattgtttgtttctgttcactcgatcgaatcgtcctcatgcccta

cgcatcacatctagacgcttgcgatcaaccaattggtaaaattttaaatttatatttgtaattttgtagt

gtaatttatatgagttaaactatcataatagtgttaattcttttaattatcaagccatgtcatcgataac

ttcacatctagaaattgttgattattgttttgtagtagtatgttaagggcccctacatttagttttgcac

tggggccctgaaaactcaggaccggccctg

>Os_hAT-N68 Oryza sativa

cagtgttcacctagtcgctaaacgctagtcggacggccacctactgactagcgactaggctgattaggcg

gcgactagacggattaggcgaattagacgttttttctatttctttcacacaaaaatactgattctagaca

ttcggtactcggtaaatataggcatttaggcaaatagcataaatcatagttaagttcagaaatcatcaag

tccatacaacgcactgacactgcacacgaagcattgggtttagtacaaatctatagggatagcgaacaca

tcagtatagccactgtacatgcacatcataattcataaatcataactttatagatctgaggggctggagc

taggagagaggaagaaaaatttatctggaactggaagaacagaggaggtgaggtggtggaggggagcctg

atcccctgatggcctgatctagggttggcgtaggctgcttgagtggttccgtgcttgactgcttcaatcg

gtgtgtttgccaccttccattgacggcaaggatgaaggatgagakggagagcaggagcagcagaggaggg

ggaagaacgtggagcagcagaggaggagagggaagaacgtagcggcgcggtggaagatcgatgggcgcgg

cgacggagccgtcgctcgtggcggtgtgaaaaagggggtaggcagggattgcgctctctctccaggctct

caaataaggcaacctaaatccccttctataccgagtcggtcccgcgtggcgcttctaaattttatcacgc

ccaaattttgatttctatcgcctagtcgccgcctagacgcactgtcccgttgtctaattgcgcccaggca

ccgtcctgtccaggccgtccaggccgcctaggaccgactaggaccgtctaggcggtaaatcgactaattc

actgcctagggtgtaaactggacaaattgtcgccgactagcgattaatcaccgtctagacgggctaggcg

gccgtttaggcgaacattg

>Os_hAT-N69 Oryza sativa

taagcctggtaatgggctggccaacccatgggttgtacgggttgaaacagtaatgggttgaaaatggtgt

gtcttgtatgggttataggaaggataagggcagggttgggttgggccatataaaatgagggttacgatgg

gctggacactgtagctcagaatggggttcagcccatgggcagtaaaaaagggtcttctttgtgggagaac

ccacagcggtaggagctcggcgactggatcggcggccaccgcccgccgccgccttgcgccgccctctccc

cctccgcttccgcctcctcctctccctccccgtcgcgatcctccgtcgctgtcccgatccctgtccgcga

gcgccgacgctccgtccttcccatccagatcgcgccaccccgtccgccggcgcgtcgtacgcctccacca

gctgtcggtcctccgcctcccggtcaagatccggcaccgcgccgccgtcctccgtcgcctccgcctcctg

ttcgagatccacagcaccgtgcaaccctttctctgcaagtcggtttctgcaaccaaggtttgttttcttt

gctacaggcattgctatttttgttcttggtttcggtgcagcagattagattgctctgctaacatctgaat

gaagcaaatctttatcacatgttgaaattgttgatccagctagggattgcttatttgtttgccgattcac

agtacaaacttgtaaaaagctattaggattagattagacattgaatataatgtataagcttgtatagaga

gatcataggattctcttaaataagaagtatatgcagaggaacgaatattgtttcaaggcattgttattct

tgctagtccctctttgtgtcaaaagccttctttgccactttcagttgtactactgacactgaaatgattt

ttctaacgaatatcatgttttagattattagttctttgtgatgattattccgagaagttaactgtaactc

atattgaattttaggaatctgtaatagagaaaattctgggcatataatgatttgtacttctgcaacttgt

aatacattaagttgtatatatcttggattttaaataaacacacatatataatctgaaactgtgttggtcg

aactagctgctcttctgtttgagattaactgaaagttggtggccatgtgttgatctgaatttgaggatac

aaggatctgatattctgcctttgagtgctgaatatacatttttttcatgccacttaggtagatggcgcat

tgtgctgaatatacttaggctgatgctaaatgcatgctatttgttagaggttgtgtggctacaagttact

gcatgtccttttggcctaagtgaattcttgtgctatataggtaatttaatttatactgtaataaaactta

cttgatgtcaactagtataaattttcaatttgtttctaagtaatatacttagagttacatatctattcag

atgtagcttgatctgcactatatatttattgttgcaccggattgttctgtcctcaacatagacctaaact

ggtgataaatgtttttaacccctgtgttcaacttgattttgcaacttctaattctgtttgtaacttgata

gactatgaattggtgaatgattaagcaactggtaggagtaaagcaattctgaatttccattgcttaagta

taggaacatggattattgtactgatgcctccttcaataggttctaacttctaacctcattttgtttctac

agggagatggaaacattgcactggacaagctgtggcataagaggaaggctgagattaagcatcaatagat

cttgctagtacttttgaatgaaaaaattctgatatgattcatagatgtttattagtttcaatgcgtgcct

atttgtggcctaattttgctaaactattgtgtatgtcttaagaaagttcagtcatatacttaaacctggc

ttagtttgtggtaatctctatttaacttatctgaaaattatttgtcttactccctatagctgtgctatgc

atgtatacaaagcttcattttcatgtatgtgcgaaggctctacagttgttgtacggttgacgtgctgtcg

tgctgagcccaatgggttagtcccttcattgtttatgggttgtccaagggttcacaaagggttagggttc

aacaagcactaagaaggttacaggttggggttggtctgtgctaaatatgcatgttagtgggttagggtgg

ggtttgggtgcaatatataaatagaaatgggcaggggtggggttcggggtgggccactacccattaccag

gccta

>Os_hAT-N6 Oryza sativa

cagggttggaaatttcggaccgaaatttccgaaatttcggtcatttcggtcccctccgatacgatattaa

ttcggccgaaatttttgaatttttcaattttttcatgaatttgggtaatatttgttcaaattcaactaaa

ttttgttcaaaatttcggaaatttcggaccgaaatttccgaaatttcgggatttcggtgacccccgatca

aaacccctaaaccgatagagtgaaccctg

>Os_hAT-N70B Oryza sativa

caggggcggagccaggattatgtcaagagtagggcttaatcactagagtatgaacttcttgaacgaattt

taatagaaaaattagtaaattccttggtattttttctgccaggtgtggggcccgggcccaagctgcccca

cacctggctccgcccctg

>Os_hAT-N70 Oryza sativa

caggggcggaggcagcattttagcaagagtagggctgaaccatatagctataaattttttgtgcaaattc

tacgtaaatttcagtgtttctccattgaaaatttttgccacgtgtggggcccaagcccaagctgccccac

acgtggctccgcccctg

>Os_hAT-N71 Oryza sativa

cagtgttcacctagacgctagacgctagacggacggtgacccatggtctagagtctagccagtctagacg

agtctagacgatataccgtacactatattatcggcatatttttcttggcacacatgtaaatacgcttaac

atgttccatagaatattggagaactagcggtgctatgataggtgcagccactcgagtaaagccgtgggcc

ggagagaggtgggagcaaggtccacggtgacgactagcagaggagagacgacgtgacttcgttgcagtga

ctggcttagagtagaggagaacggtwtggaatggggtcaaaaccataaaccatatgggctttggtctggg

ctcgaagtttcagctcctgccacacaaaaaatgcattggcccattgcctagaccatagtctaaacgacag

gtttacggtctacactcgtctaaatgttgtctaattgtgtggtctagacagtctagaaccgtctttgacc

gactagacggtcaaacggtctaattcacagtttaggccctaaactagacgataacccatcgtctagcatc

tagacgcggtctagaccgtctagactatggtctaggcgaacactg

>Os_hAT-N72 Oryza sativa

taggggtgggcaaaaaaagaccgaaccgaaaaccgaaccgaactaaccgagaccgaaaaattcggtcaca

aattcggtcatcagttgtaactaaccgaattttacacggtcttttcggtcatctcgttcagttaaccgaa

ataaccgaactgaccgaatcactgtgaaagcccaatacagcccaatagcccattagctaagaaaacccta

accttccatcccgtcccccctccttcccactccacccgatcctccccacaaccccactgcgcgctgcgcc

gcctcttcctcgcgtttctcctcctcctctccgcctcttcgctccgctttctcctcctcgcaccacgcct

ctccgccttctcccccgcgcgcgccgccgcctcctcgcaccgcgcctctccgccttctcctcgcgccgcg

ccgcgtcgccgccctctccccacacgtctgcgcgcccgctcccccttccgctcttccgccacttcctccg

ccccgccttccccaggccggcgggggaggctgatgggagacgcgagatggatgggatctggactttctgg

tggagtggttgagatcgacgggatccatggctggattgatttgcttgatttgattttttttccatgaatc

atgctctacttgctacttgttggtggcatcatcatattttagtccttcatcccagttgctttttcatcga

tcttgtacagagtactcaattttttgcaattatttttgtgctgagttcggtctatgaccgagaccgaacc

gaactaaccgaagaccgaaatgctcggtcttgagttttcttgaagaccgatcggtcttgtgatcctaaag

accgaatttttcaaaagaccgaagaaccgaaccgaatttttcggttagaccgaatgcccaccccta

>Os_hAT-N73 Oryza sativa

ctgggttaccctgatacggctgcaaagtcacgtatccgtatcggatacggcaacggatacggatacgtat

cggatacgctccgatacgtatcccgtacgtatcccacatattcgcgatttccaaaaaaataaataaatca

gatacgccagcgatacgccacgaccttcctccgtcttcgctcgcatcagggatgcgccgcctcacgagct

cccaacctgatggctgatgtctctcgtgcgtgcccggcgttgccgccggacgccggctgcagccgcgcgc

gtcgctgccgtgcgcggggccacaacgtcccctccgcgagacggcagagccgccgccgccgggcggcggc

gcacctccttcgccgccaacctcgaggccaggaccgttgccggcgcacgcatagcggggccgtggcaggc

tggcagcatcgaagcacggagcactttttctttgttcgcttgactgcctggccgggtaatccatctcttc

gaaagtttcatgcttgaatctgctgtgcctttcttgtttttcttaggacaagtagtcaagtactatcgtt

tttgttagatacaagtgctagtgcatctagcaatcctagggttactggttagtgcggctgctaatgttga

tgcaaagtcatcaagagatatatgattgaactattaatatgtggtttttgggatcttaatcgcttgttaa

tttgaattgcttatagaagagaaaattgcagaatattgatatggaatcggctacactaatactagtatta

tatcgctatttctttacaaaagttccttgattttttgtatttttaatatatataaatgcaatttccttaa

ttttttgtgtgtttttatatatatatatatatataagcgtatccccgtatccttgttttttggaaaatgc

cgtatcccgtatcgacgtatcgcgtatccgtatccgtatccccgtatctaggtaacgtag

>Os_hAT-N74 Oryza sativa

tagggatgaaaatggtttggaaagtttccggtttccggacgtgttttcggaaatggaaagtgtcggtcgg

attttttttcggaaattttcagaaacggaaacaaattcggaattttttttcttggaaacagaaacgaata

cggtaaggctactatccgtcggaaaccggaatcggtcggaaactttcggaaactcggaaattttccggaa

acgaatttagattttttttctcggaatcggaaacaaattacttttcatatacagctgattagcttcagcc

ttctctactctagatgttcagatttgtactgagtattgaggtggttagttgaatggagatattttttttt

agtccggtgctttagatttgtttttcttttgcctaatgacttttttttttttattaggctatgtcatact

acttacagttatagatgaatatagttcttgttctcttaaatttctagaaagtgaagtgtacaactagttg

tattattattaattgataaaaaataagaaatgtcgatttattgttttataaaaaaataatgagctacatc

tcttcgttgatttgagaagttcagtttgagtggttttcatattccgataaatatctgttaccgtattcgc

tccgattcgtgttcgcttcgttttcgtattcgataatgttcgattctgttttcgtatctggggtttccga

ttccgattccgattccagaaaaaaatatgaaaacggaaatggtagaggtggtttccgtccgtttccgagc

cgttttcatcccta

>Os_hAT-N75 Oryza sativa

caggcccggtcctgtcaatttagtggcccggagcgaagctagaaagatgggccctttaatacaactctga

taatataatgatgtttctaacaaatgtaatgcataaaaaaacattcttatcaaacaagttgtatatcttc

ctctcctgggcttcctaaatctaagttcctatggcctatgggctcctatatccatagcatacatatatgg

ggttgggccccttgacttcgagggcccagtgcggtggcacagctcgacccccctcagggccgggcctg

>Os_hAT-N76 Oryza sativa

caggggcggagctagtatacaagagtggtgtcacaggacaccaatgacatccatattagtctaggtaccc

ttctaattttccaatgagtgacaccagtaaatcttgctttgtgacaccagcgtgtgaataattttgcact

ttcgttagacaagttagcttatgtggacatggacaactggacaacagcaacaaattaactgctagcaaac

tataatttatagttatttgaccatgcacaacggctaattcaacaagactatgcacaacggctaaataaag

ttcatgcatcataagttttcttgagtgattaggtgaaacataggctgagcaaccactattgtgaggaatt

tcttgtatttttcatatgtattattgtttcacttaaaccgtgatcgtcaattagagttgacgaatgacac

catcgacctaaaatcctggctccgcccctg

>Os_hAT-N77 Oryza sativa

cagtggcaaagccagaacaaattgaagggggtgcaccacctcaaccgtacatgtagtggcacatagaatt

ttcaagccatggtatacaagataatgatagactatcaattgttaaacatcatactagatgatggctaagc

gtatgatggaaatgtgattaaaaattcctacttaaaaatgtaaaatcaagggagccacaataaaaaagat

actcagtattgtaagacacatcgaaccattgaataagggtgtgtttttgtgaggagaggaaaaagagatt

gagaagatacgtagaagaaagcgtatgattaattaaatattaattattttaaacttggaaaatgaattaa

tatgatttttaaagtaacttttctatagaagatctttgcaaaaacacactgtagcggttggggaagtatg

tgcgcaaaaaacaagggatttttctctcctcatgcttcaaaccaacgcaccctaaaactggtggagtatg

agtactagatattagaatgtgataaaattcaaaagtcagggagctagtgccataacttctcaaattgaaa

ttttagtacttaggatcaatatacaatatactccactttcctaaatcccaatgtaaaagtataattattt

aaatcctaaactagtatgtagaaacattaaaataataactgaaattgaactctatatttaaactttaact

atttaaggtttgaagtgaagaattcagacaaattgaggaaatacgatgaggaagatctatgcccttgcct

ctatgtcattaacataagcacatacgggagatacacatgatcagtaaaacgaaaaaacaaaagaacaaag

cagtatgctgatgcagcaaattgggcaccaactgaacgcgtgcggccaagtgacctcgcggccgtgcgtc

cgtcccgccgcgctttggccagccgttggggggtgcacgtaagggagctgaggggggtgcacatgtggca

aaaacaggacatcaagcactgaaaacagaaatacaggggttcatgtgcacccccctcagccaatatagct

tcgcccctg

>Os_hAT-N78 Oryza sativa

cactgttatcaagtcgcccgactagtcgcgattagtcgtagattagtcgtaaagtcggtcaagctagtcg

actagagagccacgactcgaccaaatagcaagtcgtgcgattagtcgtggttaggttagtcgtagattag

tcgtaaagtcggtcgtaaattagtcgtgattagtcgtagcaaatcaccaattcaccatgctaattggctt

attgtgtttagtactttagttttatttcttgttgacttattgtcttgctttgctactccatagtccatag

tttatactatatacattgcttagcacagtataatagtagtaaagattatatattgcatcccctgtttttg

catgaccaagtcgtggactagtcgaccaagtcgacaagtcggtccaggggtacctcgactcgactttacg

acttgacaaccatg

>Os_hAT-N79 Oryza sativa

cagggccggccctgactttttggtggcccggtgcgggaaaaaaaatgaaagcccccattcaccataaaaa

tataagataatatttaaaaactcattgtcattaacgaatttcacactaataagtttaaaatacaatcaag

cagtcacgatatgctcgatcgtcgattggtcttctgtctttcctctaagcctatcaggcctgataccata

gtatacatattttggaggcccctaatttttagaggcccagtacggtcgcaccgtccgcactgccccaagg

ccggccctg

>Os_hAT-N7B Oryza sativa

cagtggcggatctaacatgggacatgggggttcagttgaacccccaaacctttttgggaaccatcaaact

gttattagtattaaggttaaggcaaatttctagaacagaagctagggttaacgcaaattcgagaaaaagt

gcgtgggtagagagagagagagatgggaaagaggatgtgcgtaacggatgcggcggaagcagcaggacga

gcgacggcgaccctgctcgctcgctcgcgcgatggatggggatcggcgcaggcggcggtggcggaggatg

tgctcaccaggatggatgcggcggaagcagcaggacgaacgacggcgcccctgctcgctcgctcgcggga

tggatggggatcggcggaggcggaggcggggcgggcgcgagggcaaactgacgaggaggcttcaggataa

ctcacgcacgacagaggcgtcaaaatggcgagggtaatttagtaatttcgcctagcactcaatccacccg

gattcttgcacatcccatccccacctacccaaacggtgaaatctgaaaaatagaaataaaatatccatca

gtcctaatcccattttccaaatggagcccctggggttgaacctttgagacggtggatttttattttattt

ttttatagggttgagatggtggataaataataggtacgtttgggcattgcccgattaggcacggctcacc

gaacgggtcgtgccgtgtcggccccacgtgctgaggctggagcccaggcacgacctagtgagatgtcaat

ctaaagtgggtcatgtcgtgttggcctggcacgattaggttacgtgggttgtgtggaggccggttagccc

aaatttgatatataccaagaaaaaaaatgtgaacagagtctgttgcatcctgctgcacaaacttcggcga

agctctagcgaggtcctccgctcaccgatagcacattcctcttcctcctcgtcaagcaccgtcgtggccc

ttcacttcctacttccttcgccccgagccggtgagcccacaatgaccgacatgcttagtggttgtgctca

cgaatggggttggccacgccacacgctaccgtgcaggcctacgtcgacgagcgcccaccatcgcttcctc

tccctgagcccctggcttcctcgattcttccgttctcctttcctcctggctcctgctcgatggtagttgc

tcgactggcggttgttcgccagtcgtcagtcgccatcatgccgcatggaggacgtgtcgtgccattcctg

gaccatgtcgtgctggctcgctacatgcacttgtcgtgttggcacgccaggccgggtagcaacccaagca

cgacatggctagacgggtcgtgccagcacgacccacagccaaacggaccgtgttgttcctgggctatgaa

ctgcacatgcgtgcccgggcggacacggaaagacccaacccatttgacaacctataggaggatttggcaa

actcatagccattcttatgtttctacggatgagatcaattaagacacctcagtcgcacctaattaatttg

attagtgtcgaattaaagggaaactattttttaaagatgttcccttccataatagcgattacactccagc

caacactaaaaaaaataacacacctttttcccaaaataacatgcaagcacaatataatctagaaaacata

tccattttcttcttgtttttattagatcaatttcaagtagcgttgtctgagacaaaatttatttcaatat

gcagaatgaggataaaccgatctcatggggatatacacatttgtgttgcttatctcattggtgaaaagga

aaatattttctcactaaatcaacactgttaagagagttaattaggagaaattaatctcagctggtagaaa

caaagggatatggtagacatttttgcaactacaccaggtggttttagcaacaactctaggggatccacac

cacacaaggagtcggagtcgacgccgagcagacggggaaatatcctcaaggcacccagcacgacgatttt

ctcttccttcttaggcacaattacgcagggatacttggccacctggaaatatgagatattgatatctttt

tctaacatttttaattgatttttcccttttacttatatgttatttaaactaatatttttaatcaaataga

taattatttttaacccaaaacaaacatattatcaaaatatattaaatgttaattttaatgaaactaattt

agtgttgtagatgttaataatttttaatataaacttggtcaaacttaagtaaatttgactagaaaaaagt

caaaacgacttataatatgaaacggagggagtagtaattatcatgacatattaataatattagttagtag

taatttttacatgagttactagtaattactgtgacatcaagttgcttaactaatttttagaatttaggag

atatttcctaacataatctagaattctcttaataattattatgaagataatattaatgctacatatagtc

atataaatatctataatttatatgataaattagaataaattagagagtgacatttgttataatctaatga

tcatagtgcactataaatattactcaagtgagttgtg

>Os_hAT-N7 Oryza sativa

caggggcggatccaacttgggacatgggggttcagttgaacccccaaacttttggggggaacaaacaaac

tgttacttgtattaaggttaaggctctgaaattaagagaagagcttcttccagatctagaagagaagcta

gggttaacgaacgacgcaagcaaattccagacaaaagtagtcccggtgggagagagagagatgaatcaga

aagaggatgtggatgctcaccaggatcccctcccctcccctgctctgctcgccagatggagatcggcgcc

cctcccctgttcagccgcgagacgtcttggcgcaaggagcggaggagacgacgagacgggagattttctt

ttcttttacgccaagggaaggggaagatactcgagtactccaccctttttatctcaattttttcactttt

aaaact

>Os_hAT-N80 Oryza sativa

caggggcgaagctatagcaagttattggggtcagtagaccccgatgatttcttctgagtcccttatactc

ctttctattttacagctatgaccccggtcaaagaaaacactagaccccggtgattgtttcgttaaagctc

aactggtggcaaggaacgatactattatcagctagcaaatagaaaattagttagcaattgtgattatatg

tcagtctacatagatatacatatacgttttagactggcgctcataaagtggcccttatttaggattaatt

gctccagtagccaactaatcctattattatttcttgattatcatcacaatttggttgaacctagtctctc

aagtctgaatttgaggaaattagagttttgaaatataaacgtttgatttttcaagttcagttacatgatc

atgaggcatgagcctgggggaatggaatataacaaaggtgattttcaccatataagaggttgttatattc

ttttcccaaatatagtaacctagtacaataatgatgtggttgtaattgaggccctgtttagttcccacac

aaaaaatttacaccatatcacatcgaacgtttaacacctgcatgaagtattaaatataggctaaaaaatt

aactaattgcacatattgcgactaatttgtaagacgaatcttttaagcctaattgctccatgatttgata

atgttgtgctacggtagacactttctaatgacagattaattaggcttaataaattcgtctcgctgtttac

tgacggattatgtaattagttttttttattagtgcccgatcaccccatgtgataccctatataatatccg

atgtgacacgccaaaactttacacccctagatctaaacaccccctgatatgtaaaaggtaaccactacta

caacaatagacttaaaattattagaagctatagccaattgttaggatcgttaattaatagtgaccccggt

gaccatctatcctggcttcgcccctg

>Os_hAT-N81 Oryza sativa

caggggcggagggctcggtagggcgaggtgtggcgctcgccatacctcgcgccgcccgctgaccaccccc

cccccccccttcctcgtactcctccgcgcagagcagcagccgggtcagagagagtggcgacgccgcatcg

tgagaagacgagcgacggctgagacgcggcgtggacgcgtccgcgagtgccagtgctggtcctcacgctg

atagctagtaggcccattaggcttgtttagtgtaaatgcggcccgtcttgttttgtcttgaatcccccat

ctgaccatctcctccaccctcccccaatccgccaatcccctccccccatcggcggacggcggctgctgcg

gtgcgcgccgcgcgcgggagggctgggtgaggtgaggagggcgcggccgccaggcttccagagctgcggc

tgcggcatccgctccggccaggcggcggcgcgaccagccgccagggcccaggggggcatgcgagcgcgca

gggcggggggggggcagggtgggacgaatggcacaacagcagtgcaggcgtgcagcaccaagcagcgaca

gcaagcaagccaacagccaagcatccatcactccatcaacagattctacatatgtgaacattaatcaatc

tttggtactaggtatctatctatactcagtactgtattcctattgtgatgtattgttgaaagcatgaatc

ttaggaagttgaggtattttttgagacctactcttctatctatgtttgcaaatggatgatgaatcataac

ttgtgattagttcttgtttgccatgcttaatgtagacttgttgagataatattgcgtcgtataataacgt

gcattgagatttttcgttctgtgctaacttccgtggtaattcgtaggccacacctaaaatttttttccgt

cctccgccactg

>Os_hAT-N82 Oryza sativa

cagtggcgaagctagagcaaaacagagggtggtgcaccactatagtagcgatatactaccatgttcgaaa

atgatatacgtacgacctaccatgttttatcaaagaacattatggtaaacaatataaaaccattgatttc

agtatctacattaacatgaacgagtgatataagaacgaaaacttagcggtaaattataaaactacttgcc

aaaatgggaattcccaatgacgatagttatcagcgcagtgtactgaagaagttaagattaaaatcacaac

caataggttgccaaaattggagaattactcgtccaacatcagcatctcaaggaaaaaaaaagacaaaaca

acttaatttttcgtttgaaattatcgaaaaaatggaatgaaactatgaaagcaaattgcaaaactttctt

ttgacgtcaccccttgcctgctacgattaataggtgataagaccataggctgcggctggccaccgagtgc

attctgtttcaccatgaagtagttaacagcgctgctatgcagggcaaatttgtcaactaaacatgctgca

ccacaagaaaatcctcatggctgtgcgctttctctgttggccttgttacctttgctcggcctgatggtct

gcttgggggtacacattgttgggatgaggggggtgcatatatggtggagatggggtaaccatagctaaaa

cttttctcttacccgggtgcctaggaacccccatagctgcatgtagcttcgcccctg

>Os_hAT-N83 Oryza sativa

ctgtgttacctggatacggcacaaaagtcacgtatccgtatcggatactcgcacggatacggatacgtat

cggatacgctccgatacgtatcccgtacgtatcccagattttcgtgattttcaattaaatagaaaaacac

ggatacttcgccgatacgtcccgaacgttcctgatatggttccatctgctattcctccggatgctcctca

tgctcgtggcctcacgtggtcccggtgccgtcgtcatcgccacacgcggaagcaacagcatcgcagctag

gcgcagcggaagattgcccgcgccaacgccaggcggaggccgtttggccgctgccgccgccgcctccgcc

gctgccgctgcaaccgccggcggcaccgtggaagcacgagtatacttctcttcgttcccctgtgagccaa

atctcctcttctggcttggctttccaagttcccatgcttgatctgtaattttttttcttattttcttgct

acaagtagtcaagcattatcatggattttggatttggaagctcaggctagtgcatgtgcatctagcaatc

ctagtgctagcgcggctgctcatgcagctgctgatttatcaagagaaagcaagggcaagtaacatcttga

tattggtggtggtcgatcggtaggtgctgggtcctcctacttgcctagtcggcacttcacttgatgttgt

aagcacgatgcatcaatttacatgtgttgatttttgtctgaattcgccaacttgaattatctagaaaaga

taatttggagaagattgatacggaattggctgaacactcctattttattgctatttccttaatatatata

aatatatacacgtatccccgtatcctaatattttgaaaaatgacgtatccccgtatcaccgtatcgcgta

tccgtatccgtatccccgtatcaaggtaacatag

>Os_hAT-N84 Oryza sativa

catagtggaaaaaaccggaccgggccggcggttcgaccggaaaaaaccggaaccagaggtctccacggtt

cggttcagctgaaagaccggacatgcattcgacccggattgaaccggctgaaccggscgggttttgacta

aaccgggcattaaaccgcctcggtcggaccggacmaaggagataatgggccacattaaggcccagtccag

gcccacgcgactgtttctttacaaaaatcatagaaaagggtggtgggaggggattcgaacctgggtctgt

gcactgagagcgcttggctctagccatcccaccatttgcttcacttgtgttatagaggaaatacatattg

catatattagttattgaaccgcggttcaaccggcggttctccggtcagaccgtcggaccggtgaaccgag

gggtcgtccgggtcactcaccggtccggttttttgcactatg

>Os_hAT-N85 Oryza sativa

cagtggcgtagccaggaatttttccttgggtagtcctagttcacacgttcatgtcacatggtaaaatttt

cttgcaccataaaactgatatacaaatacaaatttagtgaaaatgcgatagaaataatgtcttccaacat

ccataagcggcttacttaacaagcaagttcaaaatgtgcaaatattacaacatagagagtccaacaatca

acatagatcctacaaacaatagcaattacatttagcacacattcactaatctaacaatttataaattgca

ttctcagttattcacacttcacagactcacaaatctctaatcacaattcacaaatctagccatttaggct

ttaggcagctatttgttacctgggggctgagccacggaggggaggggacggcggcacggcgcggcgtcgg

cgactcggcgcggacggcggggaggccggaggggagggcggcggcgccggcacggagaggggagccggag

cggaggggacggcagcggcggcgccggcgcggaggaggggaggggacggtcgggggtggcggcgcggcgc

cggcgcagagacgcgggggagccggcgtggcgccgccggcgcggagtcgggagggcgccgggccggcggc

ggcgctggctggtggggctgacctgagtggctgggaggggaatccctccaaccctagatgtttctgggct

atttgggccaagcaacctatttgggcctaaattttttggggggtgggggtctctgggccagattttgggg

tagtcccgagcccaccgggactacccctagctacgcccctg

>Os_hAT-N86B Oryza sativa

taggggtggaaacgagccagccggcttggctcggctcgtgaaagcttggcaaagaacatgcttggattgg

ctcggcttgattaactaaacgaactaaaacagcagcttggctcggctcgtttcaaagctcgagccagctc

gagccggcttgtgatgctagcagtaagtaaacactacaacaacaaagcaatcaattttcaacaagtaaac

gtcaagtatgcatcaggatcaaactcctacaggtcatgtaggcctcaaataatgcaaattgcaatgcagt

agcaagaacaactaaaaaagagtgaagtatagcactaacagtaacacagagtcacagatacatcatacat

acactactggtacgacaattttgatttatttgccttaatctttctgtcatcaattcatcacgcacatact

acacgtactattgctccacaatcacaagaccaatagaccacaagtgcacagaccacaacatgctattgct

acatgcataagaaaaataagaaaacacatcccaaaacctcttcatcctgggctcgtcgcagcgaaaactg

ccgtcggtgggttggtgtctcggcgaggtgatgaacaacatcgagtcagggaaggtcggcgcagggagcg

tcgagcggtgctggggaggaaagcacaggggagggaggcggcgcagggagcctcgcgcggtcgcgcccta

ggcctcgagcggtgtcgcctgtcgcgcgcgcgcgttgaaaggcggtggactcggtgtgacgggctggcgg

cgtgagaaggggtctagggaaggaaatgacaagggagtgggagaggaacggtgaggttagggtttttcat

gggccaaatcagctgttaatgggccaggccatttttttgcacaagctcgacaagcttcacgagcggcttg

cgagcagcttgggctcggctcgatttagaagcgagccatggaggcagctcgagctcggctcgtttagttt

tcaagccgagccaagctttacgagctcaagccaagcccgagccaagctcacgagcccgagctttttatcc

accccta

>Os_hAT-N86C Oryza sativa

taggggtggaaacgagccaagccgagccaagctcggctcggctcgtgaaagcttggtcaagaacaggctc

ggctcggctcggcttgataaagagaacgagcaaaaaatgcagctcggcttggctcgtttcaaagctcgag

ccagctcgagccagcttgtgatgcacaacagcaagtaaatacttcaacaatagtgcaatcaattttcagc

aagttaacagcaagtgtgcattacagattctatttactacacaaaacatatggctcgaattaagtatagt

atattagtgtaggtataacaattttgatctatttgcctcaacctttctgtgattgaatcattgataatag

cacacactgcacacatacacatcaattcatcacgcatatacacatcaattcatcacgcatatacacagct

actgagaaatgaatcacaaaaatcagaaaatacatcacgaacctcttcatcctaggctcgtcgctgtgtg

aactgtcgtcgatcggttggcgtctcggcgaggtgatggacggcgtcgaccgtcgagtcagggacgcggg

tagcacaggagagggaggccgaaggcggtgcagggtagagaggcggcgccgcgacggcactggggtcgag

cgtcgctgggcggctggggagggaggccggagccagcgcagccgcgcagggcgcaggggagggaggcggc

tggctggcggaactgaggctgccgcgtcgagcggcgcgccggcgcttctgctagggactgggagggaggc

ggcgggccagcggccggcaactccttgcgccgcccagagggagggactcggtctcggtgcgatggagtag

cggcggcgacgaactgggaagtgggaaggggaatagtttctgtagcaagggaaggttagggttttcattg

ggcctgggatggggaattgttaatgggccaaggtcattttttcaccagcttgacaagcctaccgagcggc

ttgcgagctagctcgggctcggctcaatttacaaacgagctctggaggcggctcgggctcggctcgtttt

cttttcgagccaagccgagctttgcgagctcgagccaagcccgagccgagctcacgagcccgagcttttt

gtccaccccta

>Os_hAT-N86 Oryza sativa

taggggtggaaacgagccaagccgagccaagctcggctcggctcgtgaaagcttgctcaactacaggctc

ggcttggctcggcttgattaattgaacgagcaaaaagtgcagctcggctcggctcgtttcaaagctcgag

ccggctcgagccagcttgtgatgtacagcagcaagtaaatacttcaacaatagagcaatcaattagcaag

ccaaatcaacatgtacgatcaacaagacagcaacaacatattatacatttcacaagctgacaagatcaca

acttcacaagaccacaattccacaatctccatcattcattcacagcctcacactcacaggacatgtccca

acatgacacacataatcttaatagtcatgaccacaccataatagaagcatagagtaatatagttatagtc

ttatagagagtacataacttagagtagaccacattccaaaagtccaaagtctccattctcaaattcacaa

aataattatagaacatctaattattatttcgcaaaatattaaaaggaactctccaaatctccaactccta

gactttctccacactccacagcaacatgttccagccatgagccatccatttgcctacataaatgataaat

catcatgtaatcacaagcgagatagtaagaaataggaaaaaaaatagtgaagtactccatttacttacta

gttgctttgcactacactttgaggaaatgtaatactctcaacatcctcttcttcttctggcctgaaattc

agaaaaatcaaagttaattcactactatgtgttaagaagagaaatgcataatatgcatggcataatagtg

agtaccgtaatcattggagcgcctctaatccagctagaagcacatatcaacgcctcaaccattgttgggc

tagagaactacgataatcacttagaactcttcccccagcactaaaagtagattctgagacactgactggc

tggtatagtcaaaaagttcttagccattcaacttccaccacacaagtagatcaaaagctttgttgctaag

tcctcatttcgttcatcaagatataaaagcatctcagactttgatgcatcttcagaagaagattgcaaat

agactgaaaattgcttgaacagagggtagagacttgtttatgtcaaagttgaaactgatgatgaagctct

caagctctacctttgctagtgccatctttccctatgtccttctcaaacttatcataaagtgtattcaatt

cagttcgaacttcagctaactccttttcaaatcagttggataaatcttcttaaaacaccattcaatgaat

cttaacttgtaccttgggtctagaacagtagcaagcaccattgcattgtttttcacattccaatacttgt

caaatttatccaacatcgcttttcccatattttcaaattagcatctttgaagcacaagcatctctcaaag

caactttcacattgacaatatgggggtaaaaaatatttgcagtgggataggttgatcctgaaaatgcagt

agtaacctcagccaaagacccaagtattggctcaattgatgcatacaaatcccactcttctggctggctt

ccactcataatttaaatctgtttctgcatatgaatctaatgcttgctgatatgcaatgcaagttctcaac

attgataagtgaactccatcttgtggtcacatcaagtttcagccctctcccaattccattgcaagatttc

tacagatttctaaaacttgtgtatacgagatggagatttcttgatgtacttaacagtgaccttatgttct

ttgtcaaagatgccaagggtgctaagccatcattcacaattaaattaataatgtgagcacaacaacgaac

atggaagtatttagcattgtaacaacttgatcgtctagctgctagcttagcttttaaacccttgacagtg

tatcattgttggaagcattgtcaagagtaattgttataaccttgtcttcaattttccattcagctagaca

atcaaagatggcctgacctatgacattcccagtatgtggaggatccaactctcaaagttcagcactcggc

attgcattgtccaatttttatctatgtaatggcaaccaagccatgtagcaaaggttttgatttgatgtcc

acaaatcacatgttaagctaatcattccacactcctaatactcttcatgaggttttctttctcaaatgaa

aaaccttcaagcattcacttcttatggtctttctaccaataaactgatatgaagggtttaaatacctcat

cacacattgaaccaagtatgctctaccatcctaaatgggtactcatgaacaattatcatcttagctatca

gtttacgagtttcctcatggttatatcactagggtgacaatgagagggatatctgagtcacctttctctg

ccgtgaagttgagaacagcttgagccctcttcttggccaactggttcaagtaaggcgtgcacttgttcat

gtgcctcacaagatgtgtagtagctcccccagccttgtaagcatacaatgaatggcaatacttgcactta

gcctttgtttctgtctccctggagttaccttggaagaactttaacctcgtgaagttattccaacaagttg

accgcttcctcccctttacagtagcaactttgggtgaaacctttagcttctttcctcttcggcgtaccca

tgaatcccctagaagcatcactagtaccaccagtcgctgcctcatcaccacttggcattgattgacatgt

catcatgatcaaaagaattatcctttgaattatcgtcatcaatctacagttcataaacaaaacagaattt

aatcaatgcatatcgacatgcaattcatttattcattttagaactaaggtcatgtagggcaagtactgta

ccaggctattatggagataatggtacatcaatcttgtatgccatatcacttgtagaccctaaactgctgg

gaggaggcaccagtagtagagaatgcagaagcaccgacgacttaccatcgaagcacaattgatttggatt

tggatctataacatatttaaagatgagtcaaaattataacaatggaaaaacaagatgtgaagtatatgca

gtaacaaagagtcgcagtcactctcatatataatatactacacaaaacacatggctcaaagcctcaaact

atagtattggtacaacatttttgatttatttgcctcattctttctgtaattgaatcattgacagtttgac

actgacacacacacacacgctactccactgaccacaacaatctacagctacatgcctacatgtacatgca

tcacaaaaatgagaaaatacatcacgaacctcttcatcctaggctcgtcgttgcgggtgatgcatgaact

gtcgtcgatcggttggcgacatggcgtctcggcgaggtcatgatggacggcgtcgagtcagggacgccgg

cgacgcagggggcgtcgagctcggctcgagcggcactggggacggtggacggaggccggaggtggtgcag

ggaactagggaagggaggcggcgccgcggcgggccgacggcacagggggcgtcgccggggagggaggacg

gagccgacgcagccacagtaggggaggtaggcggcgcagggagcgaggcggcgagcccgcggcgcagccg

cgcagggaagggaggtggcgggccggcggcggcggcacagagggggcttcgaagttcgaacggcgctgcc

ggcctgccgctggcggtctggcgctggggaggggatggatgcggcggccggcaactcctcttgccagtcg

ccgccagcgccactgtggctgaggaacggaggaaccggggattaggggagggatcggatctggggcgtca

gggctcacggctcgcgctcggctcagctcaggagtcaggacttagggtttctctctttcatgggccagac

aattgttaatgggcttgggctcgcttagggaactcagttttccataggcttgacaagcctaccgagcagc

ttgcgagctagctcgggctcggctcggtttggaaacgagctgtggagggagctcgggcttggctcgttta

gtcttcgagccgagccgagcttcgcgagctcaagccaagcccgagccgagctcgcgagcccgagcttttt

gtccaccccta

>Os_hAT-N87 Oryza sativa

tagggttgaaagtgattcggataatttccgaccgaccggaccatttttcggatacggatagtttcggtcg

gatatattcgaaaattttcggatacggaaacgaatacggatatttttttgcggatacgaaaacgaatatg

atatagatgatatccgtcggaatcggataacggtcggaaactatccggtttttttcaccgatatctgact

tacaagaaaccctagaagtctccaattctccatccattcagatccagtccactcccagtctcccaccgtc

cagtcccagacagagtcccacaggcgcacgattcctcacctcctcatcggccggcgccgccgcggggcca

agccgtcgtcgcctcctcgctgcggggccaagccgccgccgccgccgggaccaatcgcctcctcgcccag

tcgccacatcgccgccgtcggagcaacccacgccggctctgccgcgccccatcgcctcctcgcgccgtca

cgcgccacatcgccgccgccgggccaagccaacgccgccgccgcggcgccccatcgcctcctcgcctgcc

tcaccgtcaagccccaagatttggtatgacttgtagagtcctcactgatttgatttgtagagctcctcac

tgatttgattttgttttgtgtatatgtgttatgtggcagatttgtgatgaattcctctcatgcaagtagc

agcagcagcttcagcttatgcagtgcgagtgtagcagcattgcaacaccaactcagcattgcagacttca

gagtttagactaaaaagattggttgacagtatctggcatgcctagtagtgctttgccttttcacttcaca

tgatgatggatggtatttcataatatcagtgtgtgcaagtttgtggttgtttattattaattgctaaatt

ttgaaaatgttgatatgtcctattagaaaaaataatgagttatatttcttcgttgatttgtgatgcttag

tttgaggggtttttatattccgattaatattcgtcaccgtattcgtttcgcttcgtatttgctccgtatt

tgtattcgataatattcgattctgttttcatatccgggtatccgtatccgattccgattccgaaaaaaaa

aatgaaaacgaatacgatatagctagtttccgaccgtattcgatccgttttcatcccta

>Os_hAT-N88 Oryza sativa

caggggcggattcaccgttggggcttgagccccagctccatcccggcagaaccccctgccctgcatgcag

agcagcagccgcacagcttcgggagaggagcagcgtatagcataagtgcagaacaaccaggtcgcaggaa

gaaggagcaaacaaagacaacatgtcgtggtccatgggtgattaagtggactaattggctgctaggcctt

ccttattaagctactgtattagtttttgcttttttttttgcctacttcaatggatggaacgatcagggga

ttagggaagcgagtggtttttactcacaaagtaatccaacaattatcaacaattgtgttattgaatttgt

ggttgtgcctttgtcgtattatgtcttccatcatgggaaagcgttttcatccctcgaggggatatcccct

cgttttgtgcatgtcatctaaataatcataaaaaaattaaaaaaatgattaatatgaaatatatcactcc

acaaacatgcaagttaaaattcaacttctacaagttgcaaaaaaaataaatataattgtgaatgtacgat

aactattttcaatttaatttgttatttttgttgcaacttgtagaagttatatatttgtggagtgatatat

ttcatatcaatccatgttgtcaattttttttcaaattatttagaactatttagatgacatgcaagcttcc

atcatacttcgtaaaacacattagaaaggagcaccccctatattttcgttctagattcgccactg

>Os_hAT-N89 Oryza sativa

cagtggcggatctagaaatatttgtgagcctgggcaagcctaattaacaacgccaacggatatataacaa

atcccatataactccatagactatgtaccatacaacaattttttttatcgtattctaatttagaggaaaa

cgatggttgtgtttttgcagactatgggacttagtaatttattatcttgaaaagaaaaaaaatataatgg

gtgctagaaaatttactttgctttcaggttgcagacgtctaatagcttacactcaagtaatgggtacaac

gatctagtaaacaatcatggtgcttcaggttactctagatgattgtgtatgtaacatctattaacagatt

attaaaacatacttatactgctgcatgctgtggctaagcatgttctctaatacttatacaattattaagt

gtcccaaccaagtagtgtgcttatactgctgcatgctgtggctaagcaccatgatttagaactaatctaa

tgatgcatgcggcgccggagcccgggcaactgcccagggtcgccgggccctagctccgccgctg

>Os_hAT-N8 Oryza sativa

cagggtttgaaatttcgtttcgaaatttctgaaatttcggtaatttcgcccccccaccggcaagctcaaa

tctcgcccgaaattttcggttttttgcaatttttttgtgaatttggtcaaattttattcaaattcattca

aaatcagtcaaaatttcaaataatttcggccaaaaatttccgaaatttccgaaatttcggtaattggtcc

ccccggcataaaatccaatttcgaaattgaaaaccctg

>Os_hAT-N90 Oryza sativa

cctagtgtaaaaaaccggaccggaccggcggttcaaccgaaaaaaaccggaaccagaggttggcacggtt

cggtcgaatagaaagaccggacatgcaattgaaccgcttcagaccgcctgaaccggccggttttgtgatg

aaccgggcaataaaccgctttcggtcggaccggactaacacaagtccggtgaatgaatgggccacaacaa

cggcccagtagcatgttttgggccagacttgtccgcgggcttgatttttttagcaaaaattgaaggaaac

gcgcgcgcttgtggggagtcgacccctggtcggttgtttgcaagccaactcccgtagccatcctaccata

cagatctatgtgcttgtatgcaaacacatatgttacatatagttagtgaaccgtggttcgaccggccggt

tcacggttggaccgtcagaccggtgaaccagcagggtgaccgggtcgtctaccggtccggttttttccac

tatg

>Os_hAT-N91 Oryza sativa

taggcgtgggcaaaaaaaatcccgaagactgaaaaccgcaccgaaccgaaccgaagaggcggttttttgg

tcatcggttgattgtccgtttttattttcctgtagttcggtgttggcttttgcttcggtttccatctcat

gaaccgacgagaccgaagaaacagatgagtgttcagccaatccaaaatgctcgaggcccactaacgtgtg

catcaagcccaactttcactgtggcccaaaaaatacatgacagctaacagatccaccaaatcctattccc

tctctccaaatcaagcacaggccctcgggcagagctgggcaggccgtgcgtacttgcgggaagtcgaccg

cggtgtgcggcatagatctttctcggtaaaaactgaaccccgaaaaaaccgaaccgatcttcttcggttt

attttgctcggttgatatttttctagaacttccttcggtttacattttacgaaaaccgaattccaagcaa

cccgaaaaaaccgatccaattaaaccgaggaaaccgaacgcccaccccta

>Os_hAT-N92 Oryza sativa

taggggtggacagaaagctcgtggctcgttagctcgctcggctcgtaacaggctcggctcggctcgtttg

aattttctaacgagccgagctggcattttagctcgttagagataacgagccagctcgagctggctcgcga

gctgctcgcgagccttaacgagctaggactccatcacagcccaaaagcccaatacaacaaaaaggcccaa

gcccaagacactgcaaaccctagctagtcctcactcaattccccactctcccccagccgccgccgccgct

ccgcctctcccccagccgccgccgccgctcmgcctctccccaagccgccgccgccgctgctccgcctctc

ccccagccgccgccgccgccgggagatcgagcacccgccgccgccagtctccgcctctcccccaccagcg

cccgccgccaccggtctcctcctctcccccgccagcgcccgccgccgacgggagatcgagcgcccgccgc

cgccggtctccgcctatcccccacgccagccgccgccgggagatcgagcgccaacggcccaccgccgccg

atctccgcctctcccccagcgatccagcgcccgctgccgccggtgcccgcccctagcgcccgacgctgcc

ggcgagctcgagctcatctcctccaagtgccggcagagctgcatcgagttcatcccctccaccgtcgatg

ccggcgagctcgagctcgtctcctccaagtgccggcggagctgcgtcgagttcatcccctccaccgtcga

gctcctccctgtgtctgtggtcgccggcgagctcgagctcgtttcstccgccgtcaagctcctcccctcc

tcccmgtgctcgccggccgtctcttgcccctagctcgcgagcttaacgagctggctcgagcttgttgacg

agccgagccgagctaggtttctagctcgttaggataacgagccgagccgagctagctcgttatcctagcg

agccagaccgagccgagccgagccagctcgacatccaccccta

>Os_hAT-N93 Oryza sativa

cagtggcggacctacagtaggacatgggggtacagttgtacccctaaattttttaacaatctactgtgat

ttaacaagtaaaatattgtctgatagttcaaaacacccattcttaagcacaaaatcatgctttccaatag

tattataaaatttttcttagctattttgttatttctataataatcatttttatttgcactagtaacccat

gttagcacttcatttttttctcactggacgatgctttggtaggtctgtacccccatcatcgaaatcctgc

gtccgccactg

>Os_hAT-N94 Oryza sativa

tagggatggcaatggggccccgttccccgttccccggtggggaattcctctattagggtgagctatcggg

gtaaattcatcccccacggggtaacaaacggtcaaaagcaggccccgtcgggtgggccgggggcggggcc

gttccaccactccccggccccgtatccccatggggctccaagatatgacgtcccatcagcaaatttctta

ggttgcagtccatttaggcccaattagaagcagtatatatatagagaaaccctaacttttcaatcccgtc

cccactccctcctggccgccgctcgcaggcatggtcgcactcgcacggagagaagtcccgatccccttcg

catttttctggatccgccgatcccctttcctctcttttcactagttcgccggtctagcccgcctctttgc

catcgctattgcagcgccgtcgtcagtttgtgactggagccatggcggcstcgactccccgcggcsgcgc

cctctcctgctccacctccacccccagcgactcacagcggcgtcgccctcattttctccgctcttgctga

ccaggagcgatgatgccgtcgtgagacgcaagcwacctgsacggcattaactcgatctgattttaaagca

tcaacatgtagtacttatctattgctttagtgtgtgcgccactcgtattcactgatcactagtgcatcaa

gtttctgtttctattgtacatctacattggattttgaagttcttcacccttgcttcmtttcwttggtgtg

aactgtgtttgtaaactctttatcatctgttcctgctcaattgctctatgtatwttggtgtttgtttgtc

gggtgckaggacccgcgggtgccccgtacccgctgcggggacggggacggkgggaaaccttcccccgagc

acggggatggggackgsgaacgggggawttccatcacctcgggggcggggacggggtagtgaaacccggc

gggtgcagccccgttgccatcccta

>Os_hAT-N95 Oryza sativa

ctgtgttacctggatacgtccagaaagcgwcgtatccgtatcggatacgtgaagggatacggatackatc

cgatacgtatcgccgtacgtatcagcgattttcacaattttcgaataattaaaataaatctggatacgtt

ctcgatacgtccgtatggcttcatccctagtaasgatgaatgcgctgcctccacttttctcgcccgaggc

cgaccacctcgcggcactttcccatcgtcggcgccgccgcccaccacgccgaaggccgaactcagcaccg

ccgccaaacgccgagcgcaacgccgccggcgccgtcgctgcatgccgggccttcagacgctgaatgcaga

tctgcgtctgggtgtattcaccgcctccaccaccggacgcaacgccaacatggcacaacatcgccgccgt

cgcagccactgacttgattccttcgttccccagtgaggcaagttccattgaacgattgacttgttggcct

cttcgatgcttgagtctgcatgtgattttatctattcttgttcccctttcctgttttcttgttggcctct

tcgatgcttgagtatatatatattttatagatctgatgattattgtgactagaagtgtcaatgcatctag

cactcctagagctagcgcggctgctaatctagctgcagattcatcattcatccaaatgatagtgagaaga

agccgttgtgggatgtggtgatatgtttaggtgttagagaagaagtagaaaagtcagagtatgctgaaat

acatgagtttttttaatcgatggatatctgtttattagtggtgctatttctcaactagtctccacctgaa

tcaactacaagagatcattacaaaagattgctacattgtaatcggctattttctttcaagaattaataat

tttcttatatataaatatatatggacgtatccccgtatccatattttttggaaaatggtgtatccccgta

tcgacgtatcgcgtatccgtatccgtatccccgtatctgggtaacatag

>Os_hAT-N96 Oryza sativa

taaggctggcagtggcgtgcgtgggctgatgcagccgctgggcaggcccgcaccacttacccacttaccc

acttaggccaaaccacttaggagtaagtgggctgatgcggcaagcccaccacgtccggcggcgccgccac

agcccacatcgaggagcatcagaaagaggaggaacgaggagtgaaagaggattggaacaagttcgtcgct

gttgcggcttgaaccctagccgctgccgccatggattcgatgccgcctcctccaccgcctcctcctccgc

cgcctccgccgccgccttcgccccgcctccgccgccgttcccagtgatctcgcagacctccgccagcact

gccgcttccaatgcgccctccaagggaccttcgtggtcatcgccggtcatggcggcgcactcatcgttgc

tcgtcgcacccactccgcccgttctgtcaatttctgtgggtggtgctakaccagcttctgcttcaatccc

gtcgacmtctacacaggtgagttcttgaattctttcccctgatgtggatgcagtgaaaaggagatgcaga

tgcagtgaatctgagtaggwtgaatccagwaagcaaggatgtaattttcagattcaaaccatatttaatt

ttgtaaggatgtacgcatcacttcacctaaaaaaaagaagattatctagttcctgcttgtctgtgattag

ttgctttcgtgacataacaatgtgcagttttgatggcttgcctttacataacatgcttgatggtcacctg

ggatgtaaatatgtgatatgcctgtttgagaaacatcattgaatcttgtacattaatcttatacctttct

gactttgttttgagttatatgaatagactgttctatttgctgttcgattcctgttcagcattattatgta

taaactcgttactatatatacacactgcttcttttttctctaaagttagtgattaaacaacaatttacat

gtaacaacacacatgaaaacaagttaaaaaaataatttgcttacagtaagtccccaattctgacaagcct

tatggttagctccaatggtcagtcgatgccttagattttaagctaagtttagcctgaacacacaagatta

gccttggatagctaagtttcagcactaaaatmattatctgttttgttcatgagtttcagagccccctaga

ttttaagctatgtcaattatataatgcagtcgcttggctgtcaaattccttttatgtcagaagacagttt

aagaatcaaacaaagtgtagcattgcaccgactaaggacaatttcagaagacagtttcaggttgccaacc

catgtttaccttttcatttttcctttttactgctgtaggtgactcgagagggaggggtactggcttcaaa

tacctcgagggccaaagcaaagcgtaatgccccaacatcgaaacaactaaagcattcaacaacaaagcgg

tcaaaaccaactatggtaacaatggtgaggtctccggggattcgtattatgatgtcacccatgctaaggt

ccccaataccacgtgcattgtccccacctccaacacacgagaggtcaccatcttcttcccctgaggaagc

aactacacttcaacatcgtaagtatttttttccaataaaaccttcaattgtcagaaaaaaaaatagattt

tcaattttactgctatttatttaggcaattgatgcatttactgtacattgaatattatgtttgatatttt

cataaatcataatatatgattgatttaaataattaggttcatctagaagcaaatcaaaacgcaaattaag

gtcaactgtttggaaagactttgatccgatatatgaaggagaggtgcttacaggagctcaatgtgttcat

tgtggcaaaatgttaaaggctacacgagatgtagggactagtagttgtcatagacatttactaaaatgta

agggcaaagctagaatggatcaaatgtattctcaactgatgggtgtatcatctgatacatcatgtgccga

cgccctcacaaattggacatttgatcagcatgtgtctcggagaaagcttttggagttaatagttgctcac

gagctaccctttagctttgtggaatactctaagtttagaacttttgtggctagtttaaatccatgtttca

accatatttcaaggaataccataaaggctgattgcattagtgagtatcaaaatcagaaggtagacttaca

agtggttttgagtggctcgaattctaggctttcttcactgccgacatgtggacatcaaagcaaaaattgg

gttatttatgcatcacgtgtcacttcatagatgataaatggaaattgaagaaaaggataattaggtttga

tctgctatttcactgaatttagatgtcaaatcagctttgttcccttttttgtttcccttttggatgattc

aatggagatgtgcatatgactgtgtgcttacattttgcttagacgggacacacctttactgacagacaga

tggtggtgtatttctttctttgcgttaagtggattatattttcatggaattcgatgttatgcaattctgt

cagtatatgcagggttcctgagaagttcctgagaaagtctgaacattgaggtctgtgctgaatgcagcct

ccaaattgtctgaatgttgatttattttatattctttgccagcgtaagaaaattatattctttgccatga

attgcccacatttttttaccaagttttggttataccattctaaacttcctatgcaaactcaattatgcag

aaattgcaggttgttttctctctttggtgcctgcagattcttctgattctttatggctagcaggttgctt

gctaattcctgggatcctgttttttttgctggccaattttcgctgaatagtgcaagcaacatgcatcctt

tgttctttcaaatattcacgaagtagtgcttcctgatgaaaaactcatatataatcatgatttgtcaata

tgcagaacctgaagatttgcaagcgtcaacaccatcaggcattgaagtagagcagctgcaatgaacagtc

ttgtgttattgatacaatcgttagtttgatcttcattaggatattcataatgtcctattattccactcac

attaatattgtaaattcagtgagcaatgcaacggttctgtgacttatgtcattctatttgccatcttcaa

aatcttggaggaaatttcgttgtttgggattgagtgcttagtctgaattcttctcaaacatttcttgtaa

ttgatgccactgtggtttactactacttacaaacactagcacacaaatagcaagctgattatctgatcat

gcatgttcttttaatgtgggcaaagtagaaccagcgggcaggcgggcaaggccgccagaagtttgcgggc

ttgcacgcggactgctcggcgccactaacttgttgcgggcatagcggccgcggggcgggcacgccgcagc

cgcgccactgccagcctta

>Os_hAT-N97 Oryza sativa

ctatgttacccagatacggctgaaaagccacgtatccgtatcggatacgctccgatacgtatcccgtacg

tatcgctatttacttcgattttcgaaaaaataaattaaatcggatacttcgttgatacgtcccctcccgt

cgctcctgatacgcccgaacccatccatggaagacgaagcgccgatccaatccgtcgccgcgccgcctgc

tattgctccaatccctgatgcagttcccacgggccacgtcctccccatcgccgcatccatgcccgaggcg

tgagtcctccgctagctgtcgggttccgccgccgcatcgccaaccaagccgcagggtcccaaccagcccg

caatcgagctcgagcgcctcgcgcgtcgccgcccctgagcgcagctgcgcagggccaccgccggtcgccg

catctcctccaccgggtgcagcgtctccactggccgccaagaccaacaccatcagtacgccaccaagcta

cttggttctttcccattgaagtctcgatctgcatttcttgcgtgtttccccatctcatcttagtgctaag

tgttctctcctctcctaaatccaatgagttttggaagtggcagtgcatctctagcactcgtacgtactgc

tactgctagcgtggctatgcggctgcactttgcagattcattagtagtactagcgaccagacatgggagc

acaatatccatgggagctccaaggtgagcctgtactaaaaaaaattggtgtattgatcaattgatgtcat

gtattggctatctctatttacagcctcatagttatttacctctatatatttattattttaaatatatatt

gacgtatccccgtatcggtgtttttaagaaaatgccgtatcctcgtatccccgtatcgcgtatccgtatc

cccgtatctgggtaacgtag

>Os_hAT-N98 Oryza sativa

caatgttctaaatctccggctatagccggagatagcggattgggagggaaacagctaggatatttcaatt

ttaacgctatgatttatcggccgctaatagctgcacttagccggagatagccgctaaattagccgaatat

agcggcacttagctgtggctaaaaaatttagaaagcaaaactgaaaatgggcttttgacgtgcgtggccc

acgaagttcagcccaacaacgaaccctaaacctccagtcggctcctatcctctcccactcgtgctctgtg

aggcattgcggccggcggcagcagcggctcttgcggcggcagcggggcggcggctcctcccccaagcagc

ggcggtggctgcttcttctcccggtggcggcggctcctcccccaagcagcggcgggcggctgctgcttct

cccagcgacggcggcggctgcttctcccggcggcggctgctgcttctcccggcgacggcggctgcctctt

cccaacaacgaacggccaccattcctcacccgtccccttcaccaatcstcctctatgtgcaatcaagtaa

gtgatatgtcaatttttagtccaaattctatcaaaattttgaattttttcctaatattttcgaaaaccgc

taaatgtcttagctgcagctatagccggctatagctgctcttagctactggagagctcaaccgctaagag

gcttagccggagatttagaacmttg

>Os_hAT-N99 Oryza sativa

ggcctgccagtggactgactttgtttggattaagtgggctggatcctcatttggatccttttggttggat

tgaaacggcctgggattccattttggatcggactggactgggcttacaaaaaatgtggtttggtttgggg

tggatgcgggctgagtaaaataactgatccacggactgaatccacacgcagacgctaactcatcctcctc

ctcgcgtcctcgcgtcctccgctcctcctcctccgccgccgccgccaccatcgccgccgacgggcgtcgc

tgctgctgctcctcaccactccgccctcgctgcaagcggctacggctgcggccccaccggctcccgctcg

tcctccgctccggcgtcatctcccggcgctgctccgccagatcgcgatctcctcttcatcggcgccaaga

agttgctgctgctcgccacttcgacctggctgcaagcggctgcagctccactggctcccgctcgtcctcc

gctccggcgtcatctcctggcaccgctccgccagatcgccatctccgctccgtcggcgccaagaagctgc

tgctctcctccatgcaagatccgctgccgtgtgtccctgccccccacaagatccggcaatactctctccc

ttcaagatccggcaagtactctctcctccatgtattgtggattgtgtgctgaatgccatgcttagaagat

ttgttctaaattgcatttacaagcagctacctttcattaccatgcatgcataagaatattgatttcatgt

atgagttgccatggccacaactatgaatgacctgtatgttttccaagatcagttgtgctaatttaaccat

gggtacctaacaacagtctaaaattatgatctcttttcagcaaactattatatgcacaatagttggatat

ttatcacggcctcaactactgttgtggaacatcattagattactatgtagcatgtactgaacaaaaatct

gatttattttgattttagaatactactggagtccaaagaaatggtagcttgctgctgctgatcgtactgc

accaacctcgcctgcttaagcggagaacatccatactgagttgtcgacctttattcacccacaaacttga

tactcgcccagaatatctttcaggacttatgacttatgcctggtaatactgatttggaaatacctcagat

tgatagtggattctacaagttacaatatgggttgaattagattcgtattaatttggctgtttgtagcagt

tgaatgttaattttttaatttgtcacaagcaggcttattaaacatgtctacacacaattcagaaagttca

actgg

>Os_hAT-N9 Oryza sativa

caggggcggagctagagcgaaacagagggtggtgccacttgcttaatttactctactttagatcgagttt

aagctgatgcgggtgcctaagtttgtattgagggggtgcatacatggtgaaaatagatgaggtatagcta

aaaaattttcttgaccgggttcctgggcacccccctaatatatactagctccgcccctg

>Os_HATORY Oryza sativa

cagcctgggcattttttagccgcaaaccgcaaaccgaaccgaaccgcaccgaaccgaattgttggttttt

tcggttttcggttgtcagttcggtttctgtttttgtgctattcggtgtacggcttcggcttcggttttat

agagaaaccgaaccgaacaaccgattaaaccgacatatcccgcggcggcggttttatagagaaaccgaac

cgaacaaccgattaaaccgacatatcccgcggcggcgcagcccacggcccaccagttcgcagcccatcag

gccatcaagggcaaggccaaccctacgccacgcccccgactcgtcagtcgtcacgcccccgactcgtcac

tcggcgcagccgctagcccggccgccagccgcgcagggtgccgccgcagcgctgccgctgccctacctcg

ccatcgcgggctcgaggcggcacgccgcacggccgcagcgccacactcccctcccctgagcagcagctgt

aggccccggcgagccgcccgcgagcacaccgccccctgcgcccaaagacccaaaccctaactcggcggcc

ggcaaacgggctgctggcggcgggccggcaacaccctcgtggagttctccgaccaagacaacggcggcgc

ggaccgagcagtcctcctatgtaagttcttcacttcttgttcgtttgttctagttgagtccatgttgagt

gaatgagtgttgtattgctgttctatgttggtaagttctgatttagtgatttgaaacatttgatctagat

gggggacacagatgaaactgttgcttgtgagattaatcacactcaatctcaagacactgaaaccggagtg

tcaactactggagtggtagatgcaagcaaggacaaggaggagcagcagcagtccaaggatggagagaaag

ctgatgaagaatccagaaaaagaaaaccaatggctctaagatctgatgtatgggaaagtttcagcaaggt

caaacttgctaatggggacgagagggccaagtgcaagtggtgtaccaagctatttcattgcggctcaaga

acaaatggtacgtcatccttgaaagctcatctgaagatttgcaaaaagaatcctaacaaaccagtagttg

ataatcaaggaactttgcaactgacaccatgtgatggcaatagtactcttggtactgtgaccacttggaa

atttgatccagataaacttagaaggtgttttgctgagatgataattgaagatgaacaaccatttgtccta

tctgagcgttctggtcttaggaaatttatgactttagcatgtccacgatttgttctgccatctagaagaa

caatcactagagcttgtgttaaagtatatgaagatgagaaagaaaagcttaagaaattttttaaggacaa

ttgtgtaagagtttgcctcacaactgatacatggactgctaagaacagccaaaacttcatgtgtgttaca

gcccatttcattgacaatgagtggaacctacagaagaaaattattggttttttttttggttaagggacat

agaggggaggacattggaaaatcattagagaactgcttggctgagtggggcattgacaaggtttttacaa

taacagtagacaatgctagtgcaaacaataatgcaattaagtatatgagaagggtcttgaatgagtcaaa

aggttgtgttgctgaaggagagtacattcatatgcgctgtgctgctcatattatcaacctaatagtaggt

gatggcttgaaggaaattggtacatcaattcaacgtgtccgtgctgctgttaagtttatcagatgtggaa

catctaggttggtaaaatttaagaaatgtgctgagttagccaaggtacagagcaaagcatttctaaactt

agatatttgcactagatggaactcaacctatcttatgttaaatgctgcagagaagtaccaaaaagcattt

gaaagatatagtgatgaagacccatactacaaattagaattagaaggagagaatggtccaggggtaccaa

caagagcagattgggagaaggctaggaagatggctgattttcttgaacacttttatgacctcactctccg

tgtttctgtacaaagtcgtacaacatctcacacttatttccatgagattgctgatgtattacttctgttg

agagaatggtctcatagtgaagacaagttgagcaaggaaatgggtacaagaatgctaatgaagtactaca

agtattggggagagaagtatggtgagaggcagggggacagagagaagagaggagagaaagataaggggga

tcaactactcaacttcactgtcttcttctgtgttgctattgatcctagatacaaactatcaaattgcatt

agaatgggaattaaggtgatgtttggggatacagtaggagaaaaagtatgggaaacagtgaatacctatt

ttcgtgctttgtttgaagagtacaaggaaatgtataccccaaaagataaggcaccacaacctactgaatc

tgaatctacagctgaaaccagcaaaagagtgagttgtaggtggatgtcagtaattactcagcaacttaac

agtgagggtggaagtggaactatcaaatctgaggtagacaagtatctatcagaagataatgagccggaca

caccaaaatttgacattctaaagtggtggaaggccaattcaacaagattcccaatattatctcatttggc

ccgtgatcttttggcaattcctatcacctcagttgcctctgaatcagccttcagtgctggtgggcgaact

ctagatgactttaggacctcactaaccccaagaatggttgagcgtcttgtttgtgctaatgattggcttc

gtggaggaaactatgttagtgttgaagaggacagcgaacaaatggctttgcttgaggaaggtaacataga

aaatttttatttccttccaaattctaatgtgcttcatgattcatacactattgactgttctaactgagat

catctattattttagaacttggtgacctatctatttccaaggaagatactccagctgcaactcagtaatt

cagtcctgatcagtgatcatttggagtcttggacacttggtttggtgtatgcaatgctgttgttgcctgt

tgggtgcctgctacggtgctacctgggactttatatttctctatttgaataatgaagaactcttgaatct

atgtaactgtactcttgagacatgttgatttgtgtactgtgtgactactttgtgtaattctatgcagctg

tgctgctggttattgcttgcttaaggatttgatatatctataatatcatatatgctgttgcttgtgttca

tttttatgtaattctgtattgttgtggctggtggtagtaacttttggttaattcggctaaaaccgaaaac

caaaattaaaaaaccgaaaccgaattttgcggttttcagttttttggagaaccgatcgggtggtgaagcc

tgtaaaccgaattttatagcaaccgaataaaccgaaccgaattgtcggtttaaaccgaacgcccaggttg

>Os_HATOS1 Oryza sativa

caggggcggagctacagtgatgcctatgtattcccaggaatacccaactttttttatcaaaaaacaacta

tacttcaatatatacacataacacatatacacatattagttgtccccactcctccagagtccctcgctcg

gctcacgcctcaccctcacgcgcaaatgcgctgctcgcccgctccccaattccccatttgattccaaacc

ctaggtggcggcgttcggcggcggcgcggcggcgcggcgaccggcggaagcggcaaggcgtccggcgacc

ggcgacgggagcagggagctagaccagcggtggcgcggcgaggcattcggcagcggcgcgacgccgtggc

gaggcattcggcggcggcgcggcgaggccgcgagacggcaaccgacgggcgaccggtgacggcgggagcg

aggccacgaggccgaggggcaggggcgcggcggcggccggccaacagctgcagctcagcgggacagcgcc

cagcggcactgcggcaggcgcaatttcaggtgacaaatcaagagttcaagattcaatcagtcctaaatcc

actcactatgtgtccatgtctgatgtttttttaccttagtctctgagtagtttctttttcctgtactttt

gtagttctgtagattgaagaactctcaaaatgaagaagaaatcgattgatttgaagactttgtgggatag

acattcaaagtctaggaaggtagggttaggttctggttctggatcaacaacacagccagttagaattgac

agtgaagttgctgttccttctgttcagccgattacagatgcaaatgagagtccagttcagattcagatta

ctgcagttgaggttgtgcctgaggtagcagccagtgatgcagcaagagatgtgacagaacctgaaacaga

ggtagctagcaatgatccagacatagcaacagcagtgacacagcaggagcaaccttccacttggtctcct

atcagagattggtctcccatcagagatggtgatgatgaagagacagagtatgactcaagtgatgaagcta

tttatgacattgatttactttgtcatgatcctggaaggcgaattgcaatcaaaaattatgatgtcaatga

acggaattctgtgataaggggatatattgcattagggccatgccaaccacgcagccacaattttcctata

aggaaaattggaggtaagcctcggcgcttccttcctagttggtatgacgaatttaaatggcttgaatata

gtgtggaacaagatgctgcattctgttttatttgttacttattcaagcataaaattaataattctggtgg

agatgcatttgtaaataggggatttaggaattggcacatgagaaaaaggattgcaaaacatgttggtggt

atgactagctttcacaatgtggcacaagataaatacaatcacttccttgcacctaaaacaaatattgttg

agagctttgctgccaccaatgaacaagacaaggctagatatatggctcgtttaacctattcaatgaagtg

cttgaagtttcttttaaggcaaggtttggctgcccgtggacattatgaaagtgaaaagtcacttaataaa

ggaaatttccttgagatgttgagtatgctagcagaaaactttgaagaagttggtaaggtggttttgaata

atgctccaaagaattgtaagttgactgctcccgaaatacaaaaacagatagctaattgttgtgccaaaga

aactactaagcttatcatggaagaccttggtgatgaatattttgcaatacttgccgatgaatctagtgat

gtgtaccaaaaagaacagttggctctttgtttgagatatgttgataaaaaaggaagggtagttgagaggt

tccttggtgttgttcatgttgaaaatactacttccttgacacttaaagctgcaattgaatcattgcttat

ggagcattccttgagcttgtctaaggtccgtgggcaaggctatgacggtgctagtaacatgaagggtcat

gctaatggactaaagaaattgattatggatgagtgcccttctgcctattatgtccattgctttgcacatc

aactccaattaacacttgtggctgttgctaaggaaaacccagattgtgtgtggttctttgagcaacttag

atttttgttaaatcttcttgggaattcttgtaagaagacagaaatgcttagagttgcacaagctcaaaga

attgtagaagaactagatttgggtgacattgaaactggaaagggtttgaatcaagaaatgggtttgggca

ggccagcagatactcgttggggatctcactataaaactgttatgcatgtcctatctttgtatccttcaat

tcaaaaagttcttataatggttggcaaggatcgctcttttggtgcagaatgtgcaaatgcacaaacagtg

ttgacaatattccaatcatttgagtttgtttttatggcacacttgatgcaaacagtacttgggttcacat

ctgacttgaatcatgctttgcagaagagggatcaagacattgttaatgcagtaggactgattttattgac

aaagtttcaattgcagcaattacgcgaggatcctggatgggatgattttcttcaagaagtgcaatctttt

tgtgtgaagcataagatcaagatccctgatatggactctttctatcggccggttggaagagataggaggt

tctttattaaaatcaagaatatgcatcgcttccatgttgacatgtttctaagtgtcattgatagacaact

acaagagcttaatgaacggtttgatgaggtaaacacagatttgctcctttgtatggctgcatttagtcct

atagataactttgctagttgggacaaagataagttgattaagcttgcacggttttatcctaatgatttct

caagcacagagatgaaccatcttccatcagcattgaaactcttcctcactgagatgcgtatagatgaaag

gtttagaaaagtaaaaaatcttgctaatctttccattatgattgttgaaacaaaattgcacaatagacat

gagattgtttacaagcttctcaaattggttctggtacttccagtagcaacagctagtgttgaaagaatat

tttctgccatgaattatgtgaagaataaattgagaaacaggatgggggatcaatacttgaatgattgctt

ggtcacatttattgagcgtgagatgtttttgaaagtcaaggagtgtgatattataaaccgctttcaagcc

atgaaggaacgcagaattaaagctactcttccaagccacgaaggaaccgaaatagaaaattagaggtacc

atgtaataatttgtaattttatttcttagattatcggtattgacatactgtgaaccattgatttttttta

ctatgaatttttttgttagacgggaatacccaactttcaaatcctggctccgccactg

>Os_JINHUA Oryza sativa

caggggtgggcattcggtctgactgtaaatttcggtctcggtcttcggtctttcggtcatttcggtcttt

gaaaagtgaagaccaaattttaacacaaaaatatcatgaccgacaaattcagctcggtcctaaccgaaat

tcaacaacattttcatatatgcaaagaaataatagaaattttcagcacagaaatcacaaaaaatatcata

agcacgtatgagtaaagactcttattaagttgcatgcctagatatgaaaattagagtctaatcctactga

acttagtggattgtaggttgcattatatttagacttttttttgttaatttgatcttttcggtctattcgg

ttaactgaggagactaaccgaattgacggaacaaaattcggtccttcactgcctaggattgaattgatga

ccaaatttttcggtctcagtttcggtcttttcggtttggtccttttctgcccaccccta

>Os_JOUZHEN Oryza sativa

tagaggtgaaaacgggtctggttcggtcggatctcactcttcctatatcctaacctatattttataatcg

gaattgggtcagacacagatagtgccgaatacggatacaaactcggataatgttgggaaaaaagggaatg

aatacgtaccaaaatggatacgtacactatcggttacaaatatttatccggatatcaagtcaaagcaaca

atcttatatatcacatagacaataaccatttaacaatttcatatatacataatacaattatattaggcca

ttaggaccttaggaaataaatagggtagaatacatcatagtagaaccaatacataataccaaaaaatgtc

tcacaaaaatataatacgggtaatatccgtatatttatctgggtaatacccgaattttgtaccggataat

ctgtatccgttggatactacgttcccgaacccaatcccatatctgccataaaaaacccatattcggacct

gatatccgtgaaaagtcctaggtacctgaattttcgacccgaatttgtcccgaaataatgtggccaggcg

ggcgggcgggaaatacccgcctcattttcaccccta

>Os_JOUZHENA Oryza sativa

tagggttgaaaacggagcggatacggacggataatgatcataccatattcgtttcatattttttagcgga

tcggaaacggatacggatagctcggatacggaaacaaatacggattatttcgaatacgaataaggatcga

atatgatcggacacgaatacggaaacaaatttttctcggaacacgaaaaccaactcaacttctaatagaa

acaaatatcaacatatataattagctcattttatataaaatgtagtataatttataaatattttttaaaa

tttaaataatattaatagtatggactagtgttaagagataaactactattaaaatctaaaaaggtatatt

gaaggttatagagttagaaagaaatggggtatgtctcatggcttctgcggatatccgaatagcactgttc

accggatatccgaattattatccgtatccgacggaaaccccgataccatattcgtattcgtatccgggag

aaaatatccgtattcgtatccgtatccgaactatccgagaattatccgatccgaaaggtatccgtatacg

tttttgtccggagcggacggaaactatccgctccgttttcatcccta

>Os_MIDWAY Oryza sativa

ctgtggcggatccaggatttatgtgatgggtattcggaattctaaaatagcagatatatcgataccgatt

gacacattgaatgtacgatacattattttgatgcataatctttactacgaattgtgcgcatatataataa

aatatatagaaacatttataatttcctataatcagcatcaaacatatttctattgatctaagtatgagtg

aagagtaccaaagaattcggaatttttggagattgtatagcataggtcgacaactttatgtagtaaaaat

attctgatttattgctaatgaaggaaaacactcataaaccacgaagaaactagtagctagcaaaggaata

taacttatttccgtaggacaattgatatcaattagacaacaacatgtagagaaaaacaaatgagtgggca

atatatagatgtatatacaatttgcaaagataactatttcctccgtttcataatgtaagactttctagca

gtgtccacattcatataaacgttaatgaatctagacacaaaatcttacgttatgaaacggatgaagtagg

cattaaagtaattgcttattaattgccacaaccaaaatgtaaacaagaaaattaaattagcatgaataca

acccatcccgaaattgctccaggccaagcacgcttaacctcatagttctttcgcgatcagcttccggaaa

aaaagttgcaacttattgatatgagtatcctatcaatcctattaagccatgtgctaggatgtcacaatat

attacaaaagaaagggagggactaggaagactattgatcttattgggctgaggagagctagaaaagggtg

gaattgaattgttagggtataaatatcacaaatctagatagagtatggtagtacatatatattatggtca

atatatattaggtttgtgtccaaaattcatgggtattcagctgaatacccatgaattatgtgggatccgc

ccctg

>Os_QINNIU Oryza sativa

cagtggcggagccaccatgattcttgggtattcccgggaatacccaacattttttatcaaaaaataaata

tacttatagtatatacacatgtacacatatttgtacacctaaatcctcaactctttgatccaaacacagc

ccaactagtctcttgcgatgcttccaaccaacccgacccactccgtcatgacccatcagacatcagttga

cccacttagccaccactcttccagacagccccctctcgctctccctccatgacccatccctccctggctc

ggctccctcgcgccactccccatttgcaaaccctaggtggtggcgcggcgagacggcaatcggcggcggg

agctggaccgtgctaccacggagggggaggccattgccacggcgggcggtgcccgacacttcgccaagct

aaggcggtcaggtcggttgggtggccggcggctgcgaggggcagggacgggcaacggcacagcgcttaga

caagaatatccaactttgaaatcctggcgccgccacta

>Os_TEMPINDAS Oryza sativa

tagggctggccaggaagctcgtggctcgttagctcgctcggctcgtgagaagctcggcttggctcggctc

ggctcgtttggttttcctaacgagccgagcttgcatttcagctcgttagagataacgagccagctcgagc

tggctcgcgagccgctcgcgagccttaacgagccagggcagatgagaatggaggcccagcccaacagccc

aactcggttgccggcctgccgcctgccgaaaaccctagtactcccagacggcctgacgcccagagcccca

gaccccagtctatccccaattccccattccccaattcccgatctggcttggcggccgatctcgtctcctc

cccttctctcgttctcgtctccgaccgactctcctccctggctccccgccggccgccgccagcccgccag

ccaccaccacgggcccacggctgcacggcaccacccgccagccgccaagccagccatcgcaaggcgccgc

cgccagccgccaccgccaagcgctgccaccagtcggcaccgccacgcgctgccaccagccaccaccgcca

agtgccgccgcctgccggcaccgccaagcgccgacgccagccaccaccgccaagcgccgccgccagccgc

caccaccgccaagcgccgccaccagccacccgccagccgccggcgccacgcgtttgagaagcaactcaac

gccgccgtccactgaaggaactcgacgctgccatctcgccatccacattccacgccgctgcttactgaca

tggaggaggcgccgcacccgcaaccaccaagcgtggaatcccagagcaaatgcgcgagcgcgaagccaac

ctcgtcggcagctacaggtgcaggtgcagcggcacgtacacgctccgcacggattgctcaaaccaaggtt

ggccgtagtcgaggatccgcaaccgcaaggaccgcaatcgcaagtggggcaattggatcgccgtcaccgg

catcagcatcacgagctaatgtgtccacgaccgtggtatgtatctaaattccacaatcacttgatttata

ccactgtgaattttgcatttagctttgaatgaatcgctatacctactaggctactaccaaggcagctagc

agctagtaatcagtttgtgtgtgtatgaattgaatttgtgcaggaaatggacatggtggccatttcagat

ggaggggaggatgaagaaaacagctttgacttgaatgatgagatgtcggtgagcctaccttcgtcaagaa

gcagcagtgagggagaggatggagatgacacatcaaagactcaaaaacgaggtgcaaaaaggccaaaggc

aggtatttcactatctccttctaaagttaagataacaagaggaaagcgtgctagttgttggaaatattat

aaggtgatcaatgttccctccaagaaggaaaaagggaaaatggaatgtaaggcaaagtgtaggttttgtc

atcacaactatgcatatcgccctgggggaaccactacaacactaaatcgtcacttagataagtgcacaat

atatctgaacaagcttgcaaaggctaaagctcaaggtacacttgattttcctttagctgatggttctatg

gttgtgcatcctactgagtatgatcatgatcacactaaacttttgattgcccgtatgataattctacatg

attacccatttagaattgttgagcataaagggtttaatgccttgatgaagtggatgaatcctagctatga

attcattggtcgcaaggccataaaaagtgaatgcatgaaattgtatgaatctgaaaaggaacatctcagg

aaaagtcttagggaggctgagaccataagtttaactacagatatgtggacatcaaatcaaaaccttcagt

atatgtgtttggtggctcattacatagatgttaattgggttttgcaatgccgtgtcctaaactttgttga

ggtggagcctcctcacaccggtattgtcatagctcaagctatttttgattgcttggttgattggaaaata

gaggacaaggtcatgacaataacccttgataatgcttcaaacaatgacaccgctgtttcaaatttgaagt

ctaagcttgctgctagaaagaatgctcagtttgatccagattatttccatgttcgttgtgctgcccacat

agttaatttggttgttaatgatggcctgcaacaaattcaatctttgataaccaatgttaggaacactgtg

aagtactttaagaagtctccagcccgtatgtataagtttgtgggtgtgtgcaacacttattcaattaaag

ttggtagagggttgtctattgatgttaaaacaaggtggagttcgacctatcggatgctagagacatgcat

tgagtatagaaatggttttgattattatgctgaatcagacaccaaatatgaatggctacctttacaatct

gaatgggatttgtttgagaaaattcagccaatcttgggaacaatgtctggtgcaaccactgcattttcag

ggtcaacctaccccactgccaatgttttctatccatacatagctaaagttaagattgcaatattagcttc

tagagcacaagcacaaactgcactgttagaagctgcaaggttagggcagcagtctggactttatgaacct

gaccctaatgatgtattgctggtgactatggctgatgcaatgttggaaaaattcaataaatattgggaga

acacaaacaatatcatgatcattgctacaatccttgaccctaggttcaaaatgaggtacattagatggtg

cttcagtgaattttttggtgagacaaggtgtgtaacagaggttgctgccataactgatgagatggaaaaa

ctctacagaaagtatgagcggatatgtcgccacaaccaaggtgggaacagtccacacaatggtcactcgg

cctcatcctcaatctctactaccacctcattggcttcaattattccaagtggatttcaatcctttttgca

gtcaaatgctaaagaatcctcaaagtctgagttactcatctatctagatgaaccaaatgtgtccctcgaa

gatagcactttcaacttactcaattattggaaggtaaatgcccataggtttcctgttgtgtccaacatgg

caaagaggttcttggctgttccagctagcagtgtgtcatcagagtccacttttagtaccgggggaagaat

tcttgatgactaccggagttctttgaagccagaaacagttcaggctttggtttgtgcttcaagctggata

agagcctctcaaaatgacaatagtgcacctatccctgtggtatgtaatgcaatgattattttttattctt

tatttttgtcaagtcttttgtataaaatgttcactttggactgttttcatagggagaaaatggagatgat

gacatcgaaatagtggacttccccaattgtgtggtggcaagcaactagtagggacaatttatatttattt

aataagtttatgactttcacccatttatatcatactctataatttgtttacctgcaggctacaagcctac

accctatgggagaaagggagtgtgggactatgggagaaagggagctgccaattgccatgctatttgtgtt

ttgtgtttatgtgcaactgtgttggatgaaatcatgaaaatgatgaaccttgcaggctgcagctttacta

gttatgcacttatgctaaatattggtgtattgaagtattgaactattgaacttgaagcatggattattgt

actggactgaacctatgtttaattacagtcttttgttgaactattttgaagttaattgcaatatgtgatg

aactgatgatgtatgtcactatgtcagtaactcagaatgtgtcattgtgactgtggagtactggagtgtg

gacgtttatgtacttgtgaaatgtgtttaatccaagactttgagtagattacttatatgtgaatgtgatt

tgttgctttgatattttcatgtaacaatttatcatactatcattgcatgcttggctgcttgctgtgttgg

ctgttgctgagggctaggggcagtttttggatggtttggctcgcgagcttaacgagctagctcgagcttt

taaacgagccgagccgagctgggtttttagctcgttaggataacgagccgagccgagccagctcgttatc

ctaacgagctttaccgagccgagccgagccgagccgagctggctcgatatccaccccta

>Os_TEMPINDAS-N1 Oryza sativa

tagggctggacgaaaaactcgtagctcgttagctcactcggctcgtcactagctcggctcgactcggctc

ggctcgttgtgaattcctaacgagccgagccaatgttttagctcgttagtcataacgagccagctcgagc

tggctcgcgagcagctcacgagctaaacgagctgaggtcaaaagacagcccaacaaaaatatttggaggc

ccagcccaactagacatcaggtatatatgcatagaaaccctaaatccccatcccattatcgatctagatc

ctagcctacggtcccagcgccgcaccctcgctccccccgtctcacttcgagcctccaacgctcccagcgc

ctactcgccctcccccatctcaccaatctcgcgctgccgacagcagggtcgtggccggccgcgggctgcc

ctccatatctcaccgtcggtcatcgctcggtgaatggccggttgaagaccccgagcctatatcctcgccg

ccgtcggcatcagtactgatctgaggccgtcaacatcgagcaagcttgtattccagccgttctgttactt

ctttcattgccgccaacactgaagaccggatctgttgttttctctcttttttttgttttattggcttgtt

tcatttgcaattttcatagttactatttgatattttggtacttctttgggtgaatcgctgattggctata

gattggaacatcatattatggatatatgcatttgctttatttgtttctgccatcttgctcactggattga

tatgtgctagctgtgtactgactgaaccttgcagttttattgctctggctcacgagcttaacgagctggc

tcgagctttccaacgagccgagccgagccagcatcctagctcgttaggataacgagccgagccgagccag

ctcgttatcctaacgagctagaccgagccgagtcgagccagccgagctggctcgctatccagcccta

>Os_TESS Oryza sativa

cagggtttaacttaccgccggtaaccgcgtggttaccgcggttaccgggcttaccgcggtggtacggtaa

tataaataccgcggtaacctccttaaatacaaataaatttaaaaaataatttgaatttttgataaatttt

acacggttttgtatggtttttcacggttaccgcggttaccgcgcggtaaccgtgcttaccgccggggcgc

ggtaaccccggccccggcggtaaggtaaaccctg

>Os_THRIA Oryza sativa

tagccgattttttttaatctaaacacatgcagcccttttattacgtatttgagcacaccatatttataat

tccatttctaagcatgtacatgtacaggaccacgtcaatccctcgatctaatgcctaacaaaataaatgt

accttttgcaaccttgtttctagcgataacgtccacaagttagaagcctggtaatgtaaactcatttatt

tgtaaactagtagcaatttcttaggtctattagcttgctatattcagtttatacttaaaaaatgtgtaac

aatgatttgtaaaatcaaaataacatattttgaaccatacttaataaatatgtcaaacattaatacaaag

taacaaaactcaattcaatggcctattaatttgttagacattatgttatggaacaacacttggacattat

gttatggagcaatgtctgcacttgagcatctcgtctatggattgaaatatgggtttggcgtgcttatatt

tggaattaaactgatgcatgtgcttacatgtaaaaaatcgggca

>Os_TNR9 Oryza sativa

cagtggcggatttaggccatttctgtggggtcggctgaccccacagctttttgaaaaacatcctataaac

ctcgattttcatgtataaatccaaaaaaatttagagaaatgaccccacttaaatacaaaccactagtaat

tgaccccacaggtttaaattcctggctccgccactg

>Os_TWIF Oryza sativa

tagaggtggccaaacgggccgggccaaacgggcggcccggggcacggccgaacctgtagcaggcacggcc

cggcacggcctgctacagtaacgggccgtgccggcacggcccgagtagccgtgccgtgcttgggccgctg

gccgagcccgcgggccggcacggcacggctactgtagcggcacggcccgcggcacggcacggcccgtttc

gccggcggcccgtcaggccgaaagcgccacgtgggcgggcggcggtcgaatgggaaggcgccacgtggca

cactaacggctatttgaccgttcaaatttgaaaataaccgttgggaggctaaaaaattcataaaaatttc

gaaaaaattccaaaaaatctcaaatttcgccccgaacggcccgtgggccgcaggcgtgccgtgccggcac

ggcacggcccggccatccacgggccgtgcttgggccggcggctcggcacgtgggccggcacggcacggcc

cgtttcatcagccgtgcctaacgggccgtgccgaaacgggccgtgccggacccgtgcccgtgccgggccg

ggccgtgccgcccgtttggacaccta

>Os_TWIFB1 Oryza sativa

tatacctggccaaatgggccgtgccaagccgggccggcccaagcacgaccgcactgtagaaggcccaggc

ccggcacagcacgccggcctgtgggccgtgccggcacggcccgtttacccgtgccgtgtctgggccgacg

ccatagcccgtgggccagcacggcacggcacgtttactgtagagggctggcacggcacggcacggcccgc

gagcacggcacggcacgggagcggcctagggtaggcacaccgcgcacgtggcgccaagcggccgagccgc

cgagggcagccgcggggccaggcggcgggaagcgcgcgtcgctgcgttcgcgcgtggcgcgtggcaagcg

gcgtcgcgacgtgtcgctggggctgggaggctgggtcgctctcgctctgactgcctccgtcactccgtgc

ctcgttgggagcagccgagacggcgacaggcgactcagcgagaccccgtacggcggccgaacagctagtc

aaacgacgaatgcgagagtgccacgtgtccccaacggctagtgagctaatccaacgaccgctgtttttga

gaagtagccgttggagagcaaaaaaatggaaaaaaattcgaaaaaaatatgaaatttatttctataaata

ggacacccaccggagcattctgaatcatccatacctcccattttgtgctctgttgtgctctttcgtgtga

tagatcgattttttgatttagacaaaatttgacaaaattttgtctaaaatcaaaaatagtttgtgactaa

aaatagtgcatacaagaggtgtaaagccaagcaaaggtggtaagaaagaagtacgtcatatattcagttt

tatgtattattttattttatgttatgcgaataaatattctgaaatttgtttatgttgttttaaattttca

gaatggacgaatcgaacattccatcgttcacgttaggtgatttcgaccctaactacgtgtcgaggtcatt

cccaactggtgagtatgatgccaccggatcggctccaacaccaccagttatggagccaccggcgggttca

gaagcatccggcactatgagtgggagtgcatcgacgaacaccggctcaaagagatcaagaacttccggtg

tttggcaacatttcgatgaggtggccatgacaggccctgatggaaggcaggtaacattcgcgagatgtag

aatatgcaaaaataagttatctgcaaaatcatctggtggaacaggacatttgaagcggcatgccgaggct

tgtgcaaagaagcaaggaatccaactacgacagcaacaactactactaaatcctgatggtacggtacgta

cgtgggagtatgatcctatggtagctcgagaaaatcttgcccgtttaattgctagacaagatttaccctt

gaactttggtgagagtcctgcatttgaaaattacataaaaaattctcataatcctaggtttcaagctgtt

agtagacaaaccacaacccgtgatttgaaaaatgtctatgacaaaggttatgaatcactgaaggaattat

ttagtacatgcaccttttctgtcagtgtcacctcagacatatggagtagtagggctaaagaggattacct

tagtgtagttgtacatttcattgatgatgattggcaaatgcaaaaaagagttcttggcttaaggttaatt

gatgtttcacatactggtgaaaatatagcagagagaattcgagaggttattgatgagtttaaccttgcag

ataaaatttttgctgtaacaatggataatgcatctgcaaattctagggccatggaaattctacaaccatt

attttgtatttatgctcaatcatttcttctgcatcagcgttgtgcatgccatatcattaatctaattgtt

aaatgtgggtttaagagagttaatgtacacatcgacgctgttcgtcaagcaatcacgtggttaactgctt

caaacccacggattgcacagtggaaaaggtattgttgtgcatcgggtgagcccccacgtaagtttttaac

cgatgcagaccatcggtggaatgccacttattttatgttaaaggttgtattaccttacaaggatttactt

actgttttccttcaaacacgtaatggcccaaaaaacagtgacggccagccaatactgactgatcatacct

ggcacattgttgaaaggttcaatcaatttcttgaaacgtttcatgactgtactcttctgttatctcaagt

atattatccaacagctaatttaattttgcataatattcttgaaattgccactttgttgaaagagtatgaa

aatgatgaccttttaatgcccgttgtctttaatatgaaacaaaaatatcttaaatattggaaagacatcc

ccatgttgtattcttttgcatttattcttgatcctaggggaaaattacggggattcctcaatattctttc

acttattggagatattattaatgttgattattctacctattatgctgatgtcaaaactaaattctatgag

gtatttcgaaagtatgaattaaagtttcagggagatcgcttgcaaagacccccacctgtccctgcagcag

gtaagaaaaaattacagtggagcagaatttggggcggttcatcttctagccatggtggtggtaccagttc

atcagcagcaagtggggacgctagatcgcatggtcctgccgaagagttgtccaactatttggatagcgat

gccatcaggcatgaaacgtcagacttcaacgtactcgggtggtggaatgatcataagatgtcatatcctg

tgctatcaaaactagcacgggatgtgttgacggtgcccgtatcttcggtatcctccgaatcagccttcag

tctatgcggaagaattatcgaggataggagaacaagtctgagcagcgatcatgtggaaatactattaagc

gtcaaagactgggaacttgctgcagaacatgcccaatacactgctgacaaccaagaattggccgcacagt

tcgaaaacctttatttagatgacgaacaattagggtagctagtttatattttttaagtattgacctgttg

gctgtactcttttctttgtcatggttttctcaaatatgagtttttacatgacaaagtttttaacgaggca

gcatgtatcatgtaaacatcaataaaggtcattactcttttttccccatatttttctaatatttttctaa

gtctaattatttttctatttttctccaactatccattaattttctcttagcttagttaactttcggacct

ttctctttgatttgaattgttccactgacagagtgacaggcgatagacacacggacagaggcaagtcact

gagtcagcattcagcaagtccagcgccacgtgtcgcccttcggccggccggtcccgcggccccggccgct

cgctcccgcgtgccgcgtccaaattttcatccgcgcgcgcgccttgtcggcgttgtcgccttgccagctt

gcctgcagtcgatcgtgccaacgggccgaccacgacccatgggccattgacgtgcccgtgccggcacggc

acggcacgacgttccctcgggccgtgcttgggccggggagtaggcacgtgggccggcacggcacggcccg

ctacaggagtcgtgcctaacgggccgtgccctagcgggccgtgccgccggcgtgcccgtgccgtgctggg

ccgggccgcccgtttggccaggtata

>Os_TWIFBIG Oryza sativa

tagaggtggccaaatgggccgggccaaacgggcggcccggggcacggccgaacctgtagcaggcacggcc

cggcacggcctgctacagtaacgggccgtgccggcacggcacgagtagccgtgccgtgcttgggccgctg

gccgagcccgcgggccggcacggcacggcacggctactgtagcgggccggcacggcacggcccgcggcac

ggcacggcacggcacgccggcggcccgtcaggcgcggacagggcgggcggcggtcgaatgggaaggcgcc

acgtggcactaacggctatttgaccgttcaaatttgaaaataaccgttgggaggctaaaaaattcataaa

aatttcgaaaaaattccaaaaaatctcaaatttcgccctataaatagggcatgaaccccagccatttctc

ctcatcccacactcctcatcttgtgctctcaagtgttttaagtgctctctttgttctcaagtgtgcattt

tttttgattttgacaaaatttgctcaaattttgtcaaaaatcaaaattagtttcgtagttcaacagtttg

atcgcagaggtttgaagagctcgcggttggaaagatgtaagtaatattcaaatttgtgtattatttgtat

tgtgtttgtgaattcaataaatattcgaaaatttgtttatgtcggtttaaattttcagaatggatccgaa

ctttccataccagtcgccgtcgttcaccttgggtgatttcgaccccaactacatgtcggggtttgacggt

acctccggatcggctccaactccaccatctgtggaggaggtaccggttcatacggctgtcgttgaggagg

taccggttcaggcggagacagcttcggaaggattttccggaaccgcgagcggaagtgtttcgacacacac

cggctcgaagagatcgagaacctccggtgtgtggcaaagcttcgacgagataaaggaaacatgccccgac

ggaagggaggtatcgaaagcccgttgtagaatatgtaggcaaattttatctgctcgttcttctggtggta

caggtcacctcaagcgccatgcggagtcgtgtgccaagaagcaaggaatacaactccggcagcagcaact

tatggtaaacccagacggtacggtacgcagttgggagtacgatcccatggttgctcgggaatctcttgtc

cggttaatcgccaggcaagatttacccctgaactttggggagtcccctgcttttgaacattacattcagc

aatctcataaccctaggtttaaagctgtgagtaggcaaacatcaactagagatttagagaatgtttatca

caaggaagcaactgcacttaaggaactgtttagtacatgtactttctctgttagtgttacttcagatata

tggagtagtagagctagagaggattatcttagcgtagttgttcattttgttgatgatgattggcaattac

aaaagagagttttagggcttaggttaatagatgtctcacatacaggagaaaacatagctgaaagaattag

ggaagtaattaatgaatttaatcttgctgataaaatatttgctgtcaccctagataatgcatctgctaat

tctagggctattgaaatattgcaacctttattttgtgtgtatgctcaatcttttctactccatcagcgtt

gtgcatgtcatataattaatttgattgttaagactggcatgaagagggtaggtgaccacatcgatgctgt

tcgtcaagcaatcgcgtggttaactgcttctaacccgcggattgctgcatggaagaggttttgcaatgcg

gccggtgtgaaagctcgtaagtttgccaccgatgcagagcatcggtggaatgcaacgtatttaatgttaa

aagttgttttaccttatagtagtttactttctgattttgttcagtcacgtggtggcccaagaaacagtga

cgggtcttcagtactgaacgagcatgtttgggcaattgtccaaaaattttaccaatttctagaaactttt

tatgattgtactctaactttgtcacaagtttattatccaactgctaatataattttgcacaaccttcttg

aaattgctactttatttaaagaatacgaaaatgatgacgttctaactgaacctgtctttcacatgaaaca

aaaatatttgaaatattggaaaaatatacctatgttgtatgctcttgcttttgttttagatcctaggtgt

aaattaaggggattgtctgctattttatcacttgttggagatactataggtgtagattatagttcttttt

atactgaggttagacgtaaattatatgaggtttttggaagatatgaagtaaagtttcaggaagttcgcca

gcagagaccccctcctatccccactacaggtaagaagaagatacagtggggtaggatttggggtggatcg

tcttcaagttcaatccaaggtggtggcagttcgtcggctacaagtggagacgcctcttcgcatgttgtgg

ccgaagagttgtccggttatttggacagcgacgccatccaccacgaagcacaagatttcaacgtcctcgg

gtggtggaatgaccacaagataacatatcctgtgctttcaaaactagcacgggatgtgttgacggtgccc

gtgtcgacggtgtcctccgaatcggccttcagtctatgcggccgaatcatcgaagaccggaggacgactc

tgcgcagcgaccacgtcgaaatgctactaagcgttaaagactgggagcttgctcgacaacatgcccaata

cactgcggacaaccaagaattggccgcccagttcgagcaactctacctggatccagaccaaccccagtag

aattttgttagaagtagttctgacctttgagctgtactcttttctttgtcatggttttctcattttcccc

tatgagtttttacatgacaaagtttttaacgaggcagcatgtatcattgtatcctgtaatgatataaaca

tcaataaaggtcattactatttttaacaaattcttttgcaatattttcgcaagtgtggatttatctttaa

attatttcaaaataatgaatcacaatctatatttttaaatttttcaacacaacaaaaaaataccattttt

tcttttttttaacattagcaaatcattacttttttaaaaaacttttatttccattttttaaataccattt

tttcattttttaacattagtaaatcattacttttttttaaacattttatttccatttttaattttttttt

ccttatacatttcctttgctttttttttaaaaaaaaacactgtgcactgcaggctggcgggctggcggcc

tgccttcacgggccgccgtgccccgaacggcccgtgggccgcgggcgtgccgtgccggcacgggcacggc

ccggccatccacgggccgtgcttgggccggcggctcggcacgtgggccggcacggcacggcccgtttcat

cagccgtgcctaacgggccgtgccgaaacgggccgtgccggaaccgtgcccgtgccgggccgggccgtgc

cgcccgtttggacacctata

>Pt_hAT-1N Populus trichocarpa

taggggtgagcaaaaaaaccgaacaaccgattaaaccaaaaaaaccgaaccgaaaaaaaaaccgaattaa

ccgattaaaaaatcacaaaaaaattccggttcggttcggtttcggttttcaaagtctgaaaccgattgaa

ccgaaccgaaccgaaccgggttcaaccaggccaacacttaaaaaaaaacaagtataaataagatgttttt

ttctaaccctaaacctaaagtaacattctctaaagacagccggccgccccctccctttgctctctgcgtc

tctcttctctctgattttcctctgtactccgcatcccataattgttaagaattgttaggctcctgctgct

ccatgctcctctgtcctcctatcaaaggcacacctttcttcacttttgcttttaattcttttagttctcc

aggcacgcctctctttacttttgcttttgctttttatccttcttctttctatttctcacataaccttgta

attgttatataatttttcatacccatgtaaccgaagaagattgttatgtcgaaccagtaaaaggcttttc

tgtgctttccttgatgttggtcttgtgaggacaacaattggacatcgggtttggttatttattgaagaga

ccgagtataaaaacaccacacactcaatagatggtgtggatctatgtaaactatttgtaattggtttcct

ctcgttctggttttacatttgaataatcatatcttttgatcttcccttttttttttccagtcaaatgact

tcttttctttccttttgtttcgtgtatcttttgcgcaaagattaatttttgagcaaggatctaacaaaaa

gaaaactggggttggatttctggatattatgcacctaaccggtttagcttgttgagaaagccaagatttc

tcagaaaataggcacaaagctatgaaaatagaaataagacgaaccggtttaaactggtttggaaggaaaa

aaaaccgaaccgaaccgaacatggtcggttcgaaccggttttcggttcggttcggttcaaaaacttgaaa

aaaaataatttcggtttggttatttattttggcccaaaaccggaccgaaccgaaaatgctcaccccta

>Pt_hAT-1 Populus trichocarpa

gcagtggcggagccacggagggacaaaagggggcaattgcccccttaatttttttttatattaaaatatt

aatatattagtttagggaattgttactgcagggaaggaaaaagaaagttgttattgtttataattgttga

cttatatgttaatataatataatataatataatatacacagctcattccacaattttttcctttttccct

tttgtttctatatctccgcttaaaatccctgactaacaaagtttttttgttccttttcatgagctgtcgt

tccctgcctctctcgtatgcaaatttctcatcaagtatagttaagcaattaatcttttttgccttttgct

ttatactttaggtaaattttctttattttttctatttatttcattgtttaattttagttttattctttta

taattagttattaaaaatattagggtttatgataatttaggggttttgatatgaatatgttcaaatgttc

aaaataaatgtttaaatctgctaatttaatcaattaaatgtttcttttcttattataaaggaataaatta

atattcatgcaaaaccactattcttgacatctcttgcattggcagagtagtttattgaaatttaatattt

tcacatgacttgtttgtaagtaaaatatcaagtagaaagcatgtttttcaagaattgttcctcttacaca

tacactaggcattgctcctcaattaaatatctagaaatgattaagtataatttaatcaacatacttatta

tatgcatatagatatgaattgaaataggttttgattttgatgaattgacgtgtattttgctatgaatttc

atatggaagatttagaattattgtgtaactcaactgacttttatttaacagtgaattattgtgtagttgt

gttaattattatgtagacactaatgatgataaaaaaatccagcaggttcatattaaattaacatgtcact

agacaagtatttcaagcgtaaatcccttgaggatgaagagtcaatcaaagcttcaagtcatgtaactcaa

tcaagttcaaagaaaagtcatattgaaatcaaccccgacactctccttgctgaccctggcttaagaagac

caatttatgaataccatataaatgatagggatgcaatccgaagagcttatctacaaaaaggtccttgtca

accttcacactgtgattttcctcaaaaacaatttgggaatatatcaacactacgacgctttaatccggct

tggtttggtgcatacccaacatggttagagtacagcatagccaaagatgctgccttttgcttgtattgtt

acctcttcaagtcaaaagggggtgttgattcgtttgtgggtgatgggttttcaaattggaaaaaaaagga

aagatttgatcttcatattggaaagtctaatagtagtcacaatgcagctcggataaaatgtgagaatttg

atgaatgaaaaacaaagtatcatgactttgttatctgagcagacagtaaagagtcaaagtgattatcgaa

ctcgattgaatgcttcaatagagtgtgctcgttttttgttgcaccaaggacttccatttcgtggccatga

tgaatgtgaatgttcaagcaaccaaggaaattatctagagctcttgcattttctttccagaaataatgaa

gctattaaaagagttactttcagcgaagctcctagacataacaaattgacttctccagatattcaaaaag

acattactcaagctgctgcagaggagattacaaatgtgattatcaaagatctaggtgactcattattttc

aattttaattgatgagtcacgtgacatatcaatcaaggaacaaatggcngttgttctacgatatgtagac

aacaatggacatataattgaacgttttcttggcattcaacatgtgcgagatacaactgctagttcactca

aggcagctattgaagctttgttttctaaacatgggctaagcatatcaagattgcgtggtcagggatatga

tggagctagtaacatgcgaggtgaattcaatggcttgaaagcacttattctaaatagcaatccaagtgca

tattatgtacattgttttgctcacagacttcaattgactcttgtggctgttacaaagaagcataatgaag

ttggagatgtcttcaattttatttctagcattataaacatagttggagcatcatgtaaaaggatggaggt

gattagagaaaaacaatatgctagaattattgaaggacttgaaaatggagaaatttctagtggacgaggc

ttgaatcaagaaacttctcttagaaggtatggtgatacccgttggggctcccactatgttacaattattc

gtctacttgcaatgttttcatcagttcttgatgtgcttgagattataagggaggatgggatgaactcaga

acagagaacggaagcagtcgttttaacagatattatggaatcatttaattttgtgttcatgcttcattgt

ttgagaaggatactagcagttactaatgagttctcacaagcattacaaagaaaagatcaagacatagaaa

atgctatgagtttattgaaaacatcaaaggaacgattcaaaatgatgagagagaatgattgggaatcttt

actggaagaagtgtcatctttttgcatcaaacatgatattgatattctaaacatggatgatgagtacaag

cttcgtgggcgttcaaggcgaaaatctcaagggattacaaacctacaccatttccgttatgaattgttta

acaatatcattgacatgcaacttactgagttggatgatcgttttactgagacgagtacagagttacttct

ttgtgtggcatgtttaaacccaagtgactctttctctgctttcaacaaagaaaagcttnttcgccttgct

cttttttatcctagtgaattctctatagtggaccttatggtacttggtgaccaacttgatacgtatatta

ttgatctacgtggtgatgatgagttctctggtattgaaggtattgctagtcttgcagagaaaatggtaaa

aacaaagaagaatttgatatttccattagtatatatgcttatcaaattgtcattacttctaccagttgca

actgctacagtggagagagttttttctgctatgcatattgtcaagagtagattgcggaataggatgggag

ataagtggatgaatgatagtttggttgtatacattgagaaagatatcttcgataagattgataatgaagc

tattatgaagcggtttcaaaatatgaaaactcgaagagaacaattataatgtaagttttttcagtttaaa

agtatttttaaattaattttacttgatatgaattagtatatatgtttcgaattgatttctaatgcatatc

tatataatatagtatttgttaataatttgccccctcattaaaaaaactctggctccgccactgc

>Pt_hAT-2 Populus trichocarpa

caggggcggagccttttacaaggctaaggagggctgtagcccaggttaaaaaaaaattaatgacccactt

tcccctttwtcttttacacagcaaacctatgtcccacccctttcccatcttttacagcaaacctagtgtc

ccacttttccttgcaatacaaattaattactagccattagccgccactttactttccctagcaacactaa

ttaattagccacccaccttcccttgcaatactaattaattagttatattttacagcaaatctatctaaat

ataaataattaattggttcacatgtcttctaagtaacttggggttttagctatttttcttctcaacaata

tacaattttagcttggattttgaagaagaatctatacagagagccgcaagagtgtaaaatcatcaggtaa

gaatttacaagccctgacttttcattttgaattcaatgtttcacattcattacagtagattgttagtgga

gtaaacagtagactgtaccctagaaatggatgcatgctaacacactagtagtctagtactaataatttac

tggttatttcaaagttaagttctccaaatctcccagtagtttgcatcactagtttatccttgtatattct

gcatttcatgaatattctattgtggcattagtatcttttcatcttgcacctgacttccctaatccctttt

acttgaataggttataaattataatggaaaatcaaggaagcaagagaggaaaaactatgttttcattctt

taaaccaaacgaacaaacatcaaccagtaaaggacattctccatccaatgttgatgtctcaaatcgtagt

gaacaaccccctttcaaatctcaaagagttgaaattgatgttaatactcttgaacgagatcctgggttac

gaattccagtgtggaaacatcctattaatcaacaagatgaaattagaagagcttatatcaaaatgggtcc

atatcaacctaagttagcagagtatccaaggactgaatcagggagacagtatcgtcgatttcaatacact

tggtttgatcaatttccttggctagagtactctccatcaaaggatgcagtattttgttttccatgcttta

tctttgaaaacaaagtgcctcgtcatctcacattcaccaccgaaggctttagaagttggaagagggttaa

tgatggggttagatgtgcacttttgatgcatgtgggaagtcccacttcaccacataataatgctgtgaaa

tctgctgaagatttaatgaaagtaagtagacatattgataaagtgttgaatgcacaaactgttgaagaag

ttcagaaaaatcggttgagacttatgacaacaattgaaagtgttcgatggcttagcttacaagcatgtgc

atttagaggtcatgatgaatcttcggcttctaataatcgaggcaattttttggagatgataagacttatg

gggagactgaatgttgacattgatgatgttgtcttagaaaaagctccgaaaaatgcaaagtatacctcgc

cgactattcaaaaagagattttgcatattctcgcgaacaaagtgaggaaaaagatttgtgaagaagttag

agatgcaaagttttgtattttggttgacgaagccaaagatgcatcaaataaagaacaaatggctattgtt

ttgagatttgttgacattcagggttttgtacgagagcgtttttttggtattgtgcatgtttcagatacta

cttcttcaacacttaaaaaagaaatttgtgatgtgctcgctcgatataacttgcatattttcaatatgcg

aggtcaagggtatgatggtgctagcaatatgcgtggcgcatggaatggactacaagctctatttctcaga

gattgtccttatgcatattatgtacattgctttgctcaccgactacaactggcattagttgcagcagctg

gaaatgagatttctatttggttatttttctcaaaattgacaaccattatcaaccttatttgtgcttctcc

caaacgtcataccgagttacattatgctcaggctatagaaattgcacatatggtagctactggagaacgt

gagactggtagaggggctaatcaaattggtaatttacatcgaagtggaactactcgctggagctctcatt

ttgattctatttgcagcttaatagatatgtatggtgcaactattactgtgcttgaaagtatggttcaaga

aggatcttctaattctatacgtggagaagctggtggttgtttgattgtgatgaaatcttttgaatttata

ttcatcttatatttgatgcataaaataatggggattactgatttactttgtcgagctttgcagcaaaaat

ctcttgacatcttaaatgcaatggatcttgtatcaactactaaagcattgcttcaaactttgagagatgc

cggatttgatcttctccttgcaaatgtgcaatctgtttgcacaaaatatgagattgacataccacatatg

aatgcttcgtataaaaaggctacaggtcgttcatgtcaacaacaaggttcagtgacagtttaccagcatt

atcattatgatatatttaactcaacaatagattttcagttggaagaattaaattctagattcagtgatgg

gacagtggaactccttgtacttagctctgctttagaacctaaggacaactttaaatcatttaaagttgat

gctatttacaagcttgctgagaaattttatcctgaagatttcaatgaacaagagatgtattatttgagat

ctcagctagagcattatcagattgatgtgattcatcatgagagctttcagaatatgtctaccatttctga

attatgtcgaggattagctgaaacaaataagtcgcagcactatcatttgattgacaggttgattcgtctt

gttttgactttgcctgtttccactgccactacagagcgggcattttcagctatgaaacatgttaaaactg

tgcttcgcaataaaatgaaagaggagttcttagcagattctatgatgatttacattgaacgagagcttgt

tgaagatattgattcggattcgatcatagatgaattctattctacaaaacatcgaagggtgcaactttga

tagtataatttatttttatttttattttgaattttatgtactttaaattttattttttatatattttatg

ttatgtatttgaattaaaactttattgttaacttgacaaatttatatatccgagtgaatgattgatctta

aaaaataatttatgtgtatttatatcatagcccaggataggaaaaattcctggctccgcccctg

>Pt_hAT-3 Populus trichocarpa

taggggtgttcaaaaaaaccgataaaccgagtaaaccgataaaaccgaaaaaattaaccgaaaaaaccga

accgaaaaataaaaccgattaaaccgattagattatgtagaaaaaaccccggttcggttcggttttcggt

tttgtagtgcagaaaccgggttaaccggaccggaccggttcaaaaaagggtccctaggtataaatagaaa

atgttgcgccgcgcccctctcttctctccagtctactctccgctaaccctaaacctttcaatttcaattt

tcaaacaatctctcctctcactactcagagccacccgccgtccccctcgcccctcgccctctccggcctc

cgctaaggccgcccccgtaccagagcacccgcaacccgtcccctctcgcctcttcgcctctccctctcca

ctctccttagactwagaaaacataaacacagatcgaccatcgacgtttcctcagtctcaactggaccaaa

acagtctgtgtttccatcagaccgacgtttcctcagcctctcaaaaccagtgagttttaattttttttct

cctttctaattaattaattaattcggttaaggttattgttatttttaatacagttaagcttattttttat

acgtttcttcctcagtctccactggatcaacacagtcggtgctcgtgtttccgtcggaccgacgtttcct

gtcagcctctcaaaaccagtgagttttaatttttttttctcctttctaattaattcatacagttaagctt

attgttatttttaatcaattttgttgggtgatgaattttggtttaggtttaaatcagaatttagagctat

ttgggtgattaaagcgaattttttttcctttgtttgaatagaagggatttagcagtttaggatatgatta

tttttaatcaaaattgttgcttacttcttacaattgtttgcagtttaggattagagctgtcttgtacatg

taatttcacagtttattttgctgttttgtaaatgtaatttcaatatctgctactgtaaacttgtgttgat

tctgttgttgtgcctgttgctgtgttgattctgttgctttgttggttgattactaaaaacagatgaattg

ttgatttttttgctgtgttggttgatttataaggtcacaccaagtagtttgattaaaatcatgtttataa

gcctgtgttgattgacttgattctgcaaactagttttgttttgatgggtttttttattcggttttgtttt

gtcattctgtaaaatgtattcattattctgttttggatgatcttgattggcattttgtattcattaaaat

catgttattcttgtattcattgttctgttacttctgtaattatttttcctccattgatagattttctaag

cttgttttgacacattttctaagcttgttttcatgttacttcttaatatttgtttcattttaatttttaa

acatgattttaatctttatgtttattaagtctgatataaattatgcatgattattaacatgttttcattt

ggtaattacagatggaaaaccttcaaaatcaaaatgcttcatccactggcactaccccaacatcaaccaa

tgccccaacttcaaacactaacccaacatcaaccactgcaggatccaccacggataataaaggtaaacaa

cctcaagtccttacatcaaggaaaagaaatgttgatgataaaaaaaagtcacaaatttgggatcacttta

caaaacttgatggtgatcctaaaacccctagagctgaatgtaattattgtggaaaagattatgcatgtca

tactattgttaatgggacaagtaatatgtggagtcatttaaaagtatgcaaaaagtttccttttgtggtt

gataagaagcaaaaagttttggtattagaacctaagaaagaggagggtgaatcgggagatcgaaatgtgg

gaactcttaaggcaataggttataattatgatgaatgtagacaagcactagcgaaaatggttataattga

tgagttgccttttaattttgtggagggtaagggatttagattattttctaggaccatgcaacctagattt

gacattccttctcgtttcactgttatgagagattgtttgaaactttatgttgaagagaaggaaagattaa

ggacagctcttaggggtcaacgattgtgcttaacaacagatacatggacatcaatccaaaacattaacta

tatgtccttaacggctcattggattgataatgagtggaatttgcataaaagaattcttaatttttgtcaa

gtttccaatcatatgggtgagacaattggtcaagttattgagaattgtttgttagagtgggggattgata

aacttttgactgttacagtagacaatgcaagctctaataatgtgactatttcatatttaaagaatgtgat

gaaagattggccaactaatatattgtcaaatgagcatttgcatgttagatgttgtgcacacattgtaaac

ctcattgtgtgtgatggcttgaaagagattaatgtttcagttgttaagattcgaaatgcaattaggtttg

tgagatcttcaccttctaggcaacttgcatttaagaagtgtgcagaaaagttgcatatagagtgtaagaa

atcattgtgtttggatgttgcaactcgatggaattcaacttatcttatgttagaagctgctgaaaagttt

gaaaaggtgtttgtgaggttaggtgaaagtgaacctaggtatatgagttactttttggaggttgattcaa

aggggaataaaaaaaacatagggccacctagtttggaggattgggaaaatgctagaactttggtgaagtt

cttaaagatcttttacatggttacattgagattttctggctcattgcatgtcacatcaaattctttcttc

aatgaattgatttacatgcatacaaacttgttgcaattgtgtaaaagtagagataatcttttaagtggaa

tggcgatgaacatgatgttaaagtttgagaagtattggggttgtgaagcaaatcagaattttttgttgta

tgtggctaatgtcttggatccacgtctcaagttgaaatatgtgaaattttgttttggtgagttgtatgat

tatgacaaagcacaattgctaacaaaaaaggtgaaagataatttggtgagcttgtatgagttttatttga

aagctgatgaagtggtggatgataataggcataaacaagatgttaatgatgctattgatgacgtggaggt

agatgttaacactttggctcgattcaaaaggcatttacaggaggaagatagtgtggaaaatagaaatgag

gttgagaggtatttggttgatggttgtgaggatcctaatgatgataagttagatattttgggttggtgga

agagtaatgcttcgaaatataagatactttcaaaggttgcacaacatgttctggctattcctatatccac

agtggcttctgaatcagcctttagcacaggcggtcgtatactcgaccaatttcgaagttctctatctcca

gcaacagttcaagcacttatttgttgtcaaaattggttgcatcatggaccaattccaactgatattagaa

ccttgatgaatgattttgaaacctacgaaaaccttgagtcaggtaatttttcttataaacttacaccttt

tattttatgatttattaattaacgcgttttcaatctaacatgtctaattttttattttgtagaatttggt

ggaaagttgcatctagcaacggatgataattagttcaaaaatcggatgcatttattgaaaactatggtaa

aatactattttttatttttatatcttctaatttaatttatcatgttataattctagattttgaatttcag

gaatcaaagtgcaatgttcttgatgtgctttttgattgagatgatgtgcacaatgaagatttattttttt

tggcttggagtgttttatgacaatgttatttgttgttttttcttaagccctttgaaggcaatgctttgta

atttggattttaacttattggtgtgtgtgttatttaaacttttcgaagcagaaaaatattaggaagtcaa

aaaatatattgaacacaagattgacattaataatttatataaatgaatattcagtggcaaaaaactataa

acacaagattgacactaacaatttaatacaaatgaatatttagtggtaaaaaaattaaatatatttggaa

tcttgtaagaaaattagacaatattaacattaattacaagcccatcagaaatccattagaagcccattaa

aaagcccattaaaagcccaaaaaagcccaaaaaagcatataggttttccggtttttgataaaaaaccgaa

ccgaaaccgaaccgaaaccggtcggtttggaccggttccggttcggtttcgggtttttttttttttcagt

ttggttgttttttttaggtaaaaaccgaaccgaaccgaaaatgatcaccccta

>Pt_hAT-4 Populus trichocarpa

cagcggcggaaccaaggggaggccccaaattttttttttaaaatataataaataataggctttttaatac

taaattattattttattagttatgtttgactttaaagccatatctccgtatctctatgtaaaaggacaaa

taagcttttcaatttattcccttcctttactgcctctctctctttcttctctgaaccctaaccgaaaaaa

ttaataatacaaagcataaaggttaaagcaaagctgctgccaattaattcttcatcaaacaaggtaatta

gtaaattttttttttgtttgttaagaaatttaatatatatatatatatatatatatatatatatatataa

ttctgcttttttttttcagatctactagttttaccattttttatttcttgttatagtgaatgttgttttt

tctaattaattggatatattttatgggtttgtttgtttcgattatgcttggatttttttatgttttgttt

ttagcagagccaccaaaattatcaattttttctcacgtgtaccttactatcaccaggtccaatatacaat

tagacacgatctttctagccaaattgagttctttgaagagcgtatacccaactttgattcagtgtaagtt

ttcttttttaggaaccagtaatcctattttttatgttagattgcttttatatatcaatttctttttttaa

tatgttaattttgctttcatatgtcagtgttgtttacatgtaaaaatttatttttggttttgattattgc

ttcaacacatttttttttatgttttgtttttagcagagccaccaaaattatcaattttttctcacatata

tgttttgattttaggttgaaccatgaacaaaataagaagaattgattccttttttgagaaaaagaggaaa

aatattgataactcacagcctagtgaaccaactccaatgtgtaatgttgaagttatggttgagcaacccc

aatgtgcttcagtttacgaagaacctgcattgattagtgagcagcctcctactagaatcgacattgctca

tttaattagagatccaagcaatcgtcctcaaatttgggaatacccggttaatcaacaagatgaaattcga

agggcatacattaatttggggccatatcaacctttgatgtctgaatatccgctgactggtaaaaaacatc

ctcgtcgatttcagtctcattggttcaaaagttatccatggcttgaatattcagagaaaaatactgcatt

ttgtttcccttgctatctatttccaagtaagccatctggaaagccaggatcagacacatttactgttaaa

ggattcaattgttggaagaaagttaatgatggggaacgatgtgcttttttgactcatatgggaaaaggtc

caaattcagctcatagatttgctaccaggtgcttggaaaatttgaaaaatcagtcatgtcatattgagaa

ggtagttaagaggcaaactactcaagaaattctaaataatcgattgcgtattaaagcttcaatagatatt

gttcgttggctcacatttcaagcatgtgcttttagagggcatgatgaacgtccagaataaaaaaaccgag

gtaattttcttgaaatgatggaacttttagcatcatacaatgaacaagtaggtgctcttgttttgggtaa

tgctccacaaaatgctaaatacacctcacatcaaattcaaaaagaaattttgcatgtctttgctagaaat

gttcagtcttcaattcgtcatgagattggtgatgcaagattttgtttaattgttgatgaagctcgagatg

aatccagaagagagcaaatggcccttgttattaggtttgttgatagaagtggatttatacgagaacgatt

tttggatatagttcatgtcaaagatacaactgcttcaactcttaaggaagagatttcctttgttttatct

catcacaatcttgatgttcaaaatattaggggccaagggtatgatggtgctagtaatatgcgtggagagt

ggaatggtttgcaagctttattcattaatgattgcccttatgcatattatgtacattgcttagctcatca

attacaattggctcttattgctgcagctagagaaatatctgatgttcacactttctttcagaatttgatt

tttattattaacattgttagtgcttcttgcaagcgtaatgatgaattacgggcttttcaagcagctacaa

ttgaacatttagttgatattggtgagattgaaacgggtaaaggagttaatcaagtaggtggtttgcaacg

acctggagatagcagatggagttcgcacttcaaatcaatttgcagtttgataaaaatgtatggggcaact

tgcttggttcttgaaaacattgctttagatggatctacttattctcaacgtggtgatgcggctttttcat

ttaagttgctaatgtcatttgattttgcattcatcttacatataatgaagaatgttatgggaattactga

tgtgctttgccaagccctgcaacaaaaatctcaagacattttaaatgctatgcatttggtgactaccaca

aagactttaattcagaagttaagagatgatggttgggaaactcttttagaagaagtgacatcattttgta

agcatcaagacattgaagttcctgatatggatgcttgtttttctagtgtgggacgatctcgccgtaaaaa

aaaatcagtaacagttgagcatcactaccgagttgatatatttacagctatcattgatcaacaattgcaa

gagctaaataatagattcaatgagcaggcgatcgagcttcttaagttgagcacaactttagatcctagaa

atagctataaattattcaatgttgaagatatatgcttacttgttgacaagttctatcctgaagatttttc

tgaccaagaaaaaattcatttgagacttcagttgcagcattatgagcttgatgtacccaatcatccaaag

ttaaagaatatgtcatcgattgctgatttatgtcaaggattggttgaaacagaaaaatcaacaatttatc

cactcgttgacaggttgattcggcttattttgactcttcctgtttcgacagcaactactgaacgagcttt

ttcagcgatgaagattgttaaaacaagacttcgcaatcggatggaggatgattttcttgcaaattatttg

attgtctatatagaaaaagaaattgctgaaagattcacaattgatatgataatcgatgatttctattcta

tgaaagaacgacgagcacaattaaaataaatatgtaagaatcattctttattattttttactttatttca

aagtttatcaaacataaataatgcttcgctttagctaatgtttcttatatactttgtatgtttatatttg

ataggtacaaagaccaacaaaattaaagtgatggatacattatgaagatgaaattaacttcatagctttg

actcaatttggtattgctctaaacctcttaccttatacaaagatcatcatatgtttgtagtaagttcttt

gaataaaactaaatcatgcagtttttttttacaactcatgtacaataaattaaaaaaaattatatcttgt

tatattattttggccccccctaacaaataatcctagctccgcccctg

>Pt_hAT-5B Populus trichocarpa

catagttataaaacccggaccggcccggcgggtcgacccgggggctggaccggtccgggtttctcaaaag

accggtgaatgtaaaaaaccggtcagacccggacccggcgggtcgacccatgacccggtcaacccgggtg

agacccgacatatatatacatctgattwactcgcagwagaagtcgttcgagcagaagaacatttaactct

gccctaatttttkttcgagcagawgaagaaacatcgtgattgaggtgaatatttatcactggtkaatcga

tccaaaggcaccactggtgaatcgatcccatccakcttctacaagcggataatatcatgattgaggtgaa

tatttcgctttctccatccagatctcccttgtcccttgggttagtaacaatatcgtgatggttttattgt

tggcttcttctgtcttgcttcagccagttgtsctgggagcttactkttccgsttcagtttgcwgttaaga

gaacattgctgctataataatttccaaggtaatttccaagcctwwactccattaattataagcaaactac

tttataaacacagaatctttgatatgcgttattgctacttttgccccttgtaactttgattcctatctga

catttgggaaaacaaatcaattttttttcatccagcaaattcttaaaagagaggaaaaaaaatgtcaaat

ttcattgcaaagtgaaattagtaacatcaactatatttacacatttgttgcatcctgagcattgcgatgt

gaaagatcacaactgctttccttttcagcataaagatttatcacttccttcccgtgatttgtaaacattg

atagggaaaatcctttgggatcttaagaaactaaaagaaaaaatatacaggtcaaagaaattaactagaa

cagcaaagtttccattaccattttggctatcatttcctccttttgatggcgtggggagattcaaatatac

agtattaccagagcataggcttaagcccttcatcacaagactaatatctcattagccttcagtattttac

cattactagacagagaaccagtacaagtttattatttttctcttaacagtttagttcaatctttatattt

atcctttgattaatttgatattgttttactagttggttcttgagtggtggatgattattagttcaatttg

attaattaaagtcaattttactattttgatttgtctgttagttgccatagattataagaattgatttgtg

aattaaatagtattacggacttaaattcttagtataatttatgtgggtgcttaggtacttagagttgatt

tggtttactacaggtccttaaaatgtcttctcaacatggtagcactccaagtagcgatccttcaactgcc

caatcatctgaaccttcaatttccatatcaacatcaagtggtataagaggaaaaacagatttggcatggg

gtcattgcagagaagctcctgaacttagtgtgggatgtaagaaaaccaaattagtatgcttatattgtgc

caaagtttttgcgggtggaggcattaatcgattcaagcaacatttagctggagctaaaggagaagtggaa

caatgccgcaaatgtcctccggatgttcgtcatcaaatgcttttgaatcttcaggggaatgttgaaaaga

aaaggagagctagagaaatgaaagcagatttcaatccatatagtgctaaacaaagagagcatgaagagag

gatgattagacaattagaagatgatggtaaaggtgatgacgatgatgatgatgaagccgatggtaagaag

caaatgttaccaccgaaggttgccaataaaggaaagagtaaaatcactagcgctgttaaacaatcgactg

caagttgtggaaaacagaaagaaaatgcaacattaggggcatattttattccaagaacaactcatggtgc

tcaaaagtctctccaaagttgttggaaaaacaaagaagctatcgaacgatgtgatcttgctatagcaaaa

tggatgattgatgcatgtgtgccattcaatgctgctaactctgtttactatcagcatgctatagatggtg

taacagccatgggtcctggttataaaggaccaaattttcatgctcttcgtggttattacctggcaaaggc

ggttgatgaagtgaagatttttgttgagagttatcgagaaacttggaagaagactggttgcacattgatg

gctgatggatggacagatcaaaagaggagaactttaattaatttcttagtatattgtcctaaaggaacta

tttttttaaaaacagtagatgcatcagaggcctcaaagactgctgtgttgttgcataagttgtttagaga

ggttgttttatttgttgggcctgaaaatattgtgcatatggtgactgataatgcttctaattatgttgtt

gctagcaagttgttggtggaagaatttccttcaatattttggtctccttgtgctgctcattgcatcaacc

tcatactccaagacgttggtaagctacagtcagtttgttctgttgttgatcatgcttctagtattacaaa

gtatatttataatcattgttatccattgtatttgatgaggaagttcactggagggaaagaaatacttcgg

ccagctcctactcgctttgctactaatttcattgctttgcaaagcattttagtacacaaagataatttga

gagctatggtgacatctagagaatgggtctcctctgcttatgctaaagatagcaaaggaaaaaagtttgt

tgatagtgtgttgaactctatgttttgggaagaatgtgcatcgattgtacgaatgaccgaaccattagtt

cgagttctacgaattgttgatagtgatgatagacctgctatgagatatttgtatgaggctatccattctg

caaaggaagaaatgttgaggagatttcaaaagaaaagggctaaggtgcaacctttcatagacatcatcaa

taatagatgggatggacaactgtataggaagctttatgcagcgggattttggttgaatcctcgatttcaa

tatgatgtcaatctaatggatagatatacgaacaccatttccggacttctagatgttgttgagaagtatg

caaatggaaatgcaattctgctaagcaagcttacaagtgaaatgaagttgtttaggaatgcagaacatga

ctttggtagagtgtctgcgagaaatgatcgcacccttttacctccaggtatattattattttcatattca

aagtaatttaaaaatattatcttgacataatcttacttattaattttgtatatagatgaatggtgggtga

cgtatggaacttgtgctccaaatctccaaaagctagctatacgagttttaagtcaaacttgtagttcttc

aggatgcgagaggaattggagtattttttagcacatccactctaagaagagaaatcgattggagcatcac

agacttaatgacctagtttacgtccactataatttgagattaaaacaaaagtattttcttttctctaatt

cttcaaaagttatttatattattaagtagcatcattaatacttatattgtgttaccttaactttgatttt

gtagaaattattggaaaggacggaaattatgatccaattaattttgaggcattttctgacactgaaaatt

gggtagtagaagatgatccatcatctttgacaaccgaagaagtagagatctttcaccgtgatttatcaac

catgactattcaagatactttaaatgaaggtaatggaagatttgatcctttagttataactttgtttgaa

aataaattattgtttaacatgtaatgtttattggttaattatgtttcaattgtagatttgataaatataa

atgagattgaagatgactgtgatgatgaagatacaaggagcatgatgatgtttcgataggcgttaatgag

gttggctcaattccattgacatttgattcaaattttgctcctatggacactgaagaacttaatgcctaca

ttcaaccaaagtaaagatgttgtgttagaaaagtgtggtcattccaatgaggtcttgtactttaccattc

tttttttaaattctactagtgttaagtgttcaacatgttctaaaatcttttgctttttttatgcagctga

agatgttgatttatggattctaatgcaaaatggagttaggatgttgatttatggattctaatgcaaactg

gagtttggatgttgtcaccttatatctattatgttaaattatttatctctagaacaacttgttattttat

tatccttgtctttaatatctttaaattatgttaaaggtttgtttatttgaagacaaatgcaccttctaat

ctattgatgctaattaattatgctagtacatatgttaatgtgatattttcttgttaattatgctagcata

tatgttaatgtgatattttatgtcaaaacaaccatattaacgtgtgtcttggttcttgatccgggttgac

ccgggtcgacccacaagacccgtgacccagtcattttaccgggtcaactaccgggtcgggttttataact

atg

>Pt_hAT-5 Populus trichocarpa

cagtgttttaaaacccggaccggcccggcgggccgacccgggcctgggaccggtccgggtggaggcaaaa

acccgctcgggagttggcccggtgaaacccggtcgacccggcgggtcgacccgggacccgggccacccgg

tctattttttttatactgatgacgttaaacgacgtcgttttggcctttgttaaaaggccaaaacgacgaa

gaacaatgaagcagaattgagcattcgattacagatagagcaaacctaattaacaaaaaatctttcaaac

tttcaatcgatgagctgaggagcagaggagagcagaagacgatcttgatcattgttgtttcactgcgaaa

aaggttagtttcttgtttctgttcacgaatcttcttctttcactctctcttttctttattcttggccgtg

ttgaacttgcagttctggagcatcattttgctgttccctcgtcgacccatctcttccctgttgatccagt

ctcgttgattcagtcgcagcccagtcatctcaacctcaggtatgtttctctgttctttcttttctgcagc

gtaataacttcctctgctattaggaagtgttgaaaatggatgaggaaactagaatggttcggtgcatttg

cttttggaactgttatagccatagtttgaactattttgtcaagaactttagccagagattgaaattgttg

taagtttttgttatggttagaatattttggctggtgattgttggtgtgtttgttcgttctgcttttgctt

catgaatgaagcaaactgtgatggataatgtaattgactatggatgatgattgctaaatctgttttctgc

taaatcatgaattgctatgattttgttttttgtttttcctttggcgacaatgtgtgattaaatatggatg

ggtcctcctcctcttgaattcgccttttcttgtttaaacgggaggaatattagttgtgtactaatagcat

gaaagaattggaattttatcggtatcattgcctctccttttccggtatatttaatacaaattctccaatt

gtataatttaattaatggattgcaaagtgatttggcttgaaattatattccctcttataaattatgtcag

gtccttaaaatgtcttcacatgatggtagtactccaagtagtgatccttcaacggcccaatcatctcaac

cttcaatttccatgtcaagtggtagtagaggaagaacagatttggcatggggtcattgtagagaagctcc

tgaacttagtgttggatgtaaaaaaactaaattagtwtgtttatattgtgctaaagtatttgcgggtggt

ggcattaatcgatttaagcaacatttagctggagctaaaggagaagttgaacaatgtcgcaaatgtcctc

ctgatgttcgacatcaaatgcttttgaatcttaaaggaaatgctgaaacaaaaaaaagagttagagaaat

gcaagcagatttcaatccatttaatgcacaacaaagggagcatgaagagatgatgattaggcaattagaa

gatgatgatgatggtgatgatgaggaggatgatgaggatgtcaatactaaaaaacatatgttaccaccga

aggttgcaaaaaagaaaaagattcaaagcaccagcactgtaaaacaatcgactacaagttatggaaagca

gaagaaatctgcaacattagggacatatttcatgccgagaacaactcctggtgctcaaaagtctcttcag

aattgttggcaaaggaaggaagcagttgaacggtgtgatcttgctttagcgaagtggatgattgatgcat

gtgtgccatttaatgctgttaactctgtgtattatcagcatgccatagatgctgtaacagccatgggtcc

tggttataaaggaccaaacttgcatgctattcgtggttattacttggcaaaagcggttgatgaagtcaag

atttatgttgagacttatcgagagatttggaagaagactggttgcacattaatggctgatggatggacag

atcagaagaggaggactttaattaacttcttagtatattgtcctaaaggaacagtttttttgaaaaccgt

ggatgtatcagatgtctcaaagactgctagattgttgtatcagttgtttagagaggttgttttgtatgtt

ggggtagaaaacattgtgcatatggtgactgataatgctgcaaattatgttgctgctggcaagttattga

tggaagaatttccttcaatattttggtctccttgtgctgctcattgcatcaacctcatactccaggacat

tggtaaattgcagtcagtttgttgtgttgttgagcatgcttctggtatcacaaagtacatttataatcat

tgttatccattgtatttgatgaggaagttcactggaggaaaagaaatacttcgtccagctcctactcgtt

ttgctaccaatttcattgcattgcaaagcattttagctcataaagatgagttgagagctatggtgacatc

tagggaatgggtctcatctgcttatgctaaagatagcaaaggaaaaaagtttgttgagagtgtgctagac

tctctgttttgggaagaatgtgcaataattgtgcgaatgagtgagcctttagttcgagttctacgaatgg

ttgatggtgatgatagaccttcgatgggatatttgtatgatgctattcatcatgcaaaagaagaaatgat

gaggagatttcaaaagagaaaggctagagtgaaacctttcatagacattatcaataatcggtgggatgga

caattttatagaaatctttatgcagcggcattttggttgaatcctcgatttcaatatgatgcaaatataa

tggataaacatatgagcaccatttctggacttctagatgttcttgagaagtatgcacatggaaatctacc

attgcaaagtaagattacaagtgagatgaagttgtttaggaatgctgaacatgactttggtcgagcgtcc

gcaataaataatcgcacccttatgcctccaggtatataatttttatatttaaaaatattatttgacatag

tctccttttacttattatttttttgtatagatgaatggtggatgacatatggaaccagcgctccaaatct

acaacagttggctatacgagtgttaagtcaaacttgtagttcttcgggatgtgagagaaattggagtatg

tttgaacatattcattccaagaagagaaatagattggagcaccaaaggcttaatgaccttgtttacgtcc

actgcaatctaagattgaaacaaaagtatttttcttttctctaattccttaaatattattttatcttgtt

tagtagcattattaatacttatattgtgtttaccttcacttttaatatataggaattattggaaaggacg

aaattatgatccaattaatgttgagacaatttgtgacattgaaaattgggtagtagaagatgacccgtca

atcttgacaactgaagaagcagagagttttcaccaagctctatcaactatgaccatacaagatactttag

atgatggtaattaatgattgattatttagtgataaatattatttaaaataaagttttgtttaacacataa

tatttatttgttaattgtgtttgaaatgtagatgtcataaatgttaatgatattgaagatgattgtgacg

atgaagtttcaaaggagcatgctgatgatttattaggtgttgacgagattggctcaattccatcgacatt

tgatccaaattttgctcctatggacacagaagaacttaatgtgttcattcaacaaaagtgaatgtgttgt

tgatttagaatttatgttgttggatgttttgttttaaatattttagaatttatatttggtttgtgttttg

gatattgaattaaatttatgttgttggtttaaaatttaaatttggtttatgtaagtgttgcattgtaact

agttatgtgtttttttttataggttttttttttgggttgacccgggtcaacccatctgacccgtgacccg

atcacttgaccgggtcgatgaccgggtcgggtttcaaaactatg

>Pt_hAT-6 Populus trichocarpa

tcaaggttgttaaaatcgcgattcaactcgtaaaatcgtacgtttttacgagttaatataggctttacgt

gctaaatcgttcataaaactcggaaatgggtaaaatcgggtcaaaatcgggtaaaatcgttaaaatcggg

tgaaatcgcctaaaatcgcgatttcaccaccgattgaacgagtttgcccgcaggagaacaaaatgcaatg

tagctgccacgtgtcgaccgcctattggctttaacagatcaaatgcaaagtaactgtgagtctgtgactc

accgaatcagtcttctctcttctcccttcacaattcagaatttcagacattcataattcacgaaatccct

aaattgaaaatccmaaattcaacaaccaacagtcctctctcctgaggattctcctcagtgactcagtctt

ctctcttcagtcttctcttaatctcttgcagtctttctctcagccaacaatcaaccaccgtccacctcca

caacagccctcgctcgcccctcatcttttttatttttccatcatcgacgacgtccttttcaccagccacc

gtgagaaccagagtccacacaacaacaacggtcaacgacgtctctccagtcttcaccaggtaagttacac

tgtccgtttatcttgtttctgtagtcttcmagtgccaggggtgcttttttgtccctgtccgtttcttaat

ctcatcactgatgttaatgttagtttgattggattcgcaggctccaactttgactgcaaacattcactgt

gagtcttgttgcttagtccgttactctgttcatctkctcagcctcggtgctcactgctcagaacagaggt

aagcttcttagtttcttaccattccgtttctttacagtttaatgatgtctaatctgttattgagacatgt

taatattcaagtgtccgtcaatttgagagtatgcctgcgaattttggattgaaatataaccggtgacagt

gtgtgtgtgtgtgacagtatgtgtgtgtgacagtgtctaaatggagagactctgttttttcttaccctgg

atttggaatttagagtggtgattgatgatgttgctgctgagttgtgatgaatggatttattgtaatctgt

tttagagtgctgctgctgctgagttgcgaatgagttgtgaattgtgatgaatggatttattataatttag

aatgatgctgctgctgctgagttgtgatgcatggatttattgtcattttaccgtgatttgggttgtaact

tgtgatgaatggatttgggttgtaaattgtaatttagagtgctggttgactgtttttatgctgcctttgc

tgagtagtaaagggttgtaacttgtaagtttgtaacctgtaatggataataatatagaagatgggttgta

gtgtgtattaaaatcaagtttataacmtgtcataaaatgtttggattgattacagggctgatttgagttg

attcttgaattgttgaacttatggcatctagaaaaaatgcttctggtaataggcttgatgtgggatggca

gcatggtatagatgttgataagaattctagaaaagttctgtgcaagtattgtcaaaaaattattagtgga

ggtatwtttcgtttcaaacagcatttggcttgcactcgtaaggatgttgagccatgtcagcaagtgccag

aaaatgttaagcagatgattttgggtgttttggtgaaaaatctagaggcaactgaaaagaaaagaaaggc

ccttcaatatagtggaaatgatgatgatgatgatgaaataaaagaaattagctccaaggacaaaggaaag

agagtagctagtgggagtggaagtacacaaacaactctaaatcaattgctaaaaaaggatattagagaag

aagcatgtcgacaaattgctaggtttttctacactagtgcaattccatttaattgtgtaaaaaaccctga

gtttattaaggcacttgaaatggttgcaaagcatgggccaggtttcaagcctccatcctactatgatatt

agagagaagtatttgaagcaagaagtggatcaaacaatgaaattgcttgaggagtacaagctagaatgga

aaaaaacaggttgttcaataatgtctgatggatggacagataaaaaaagacgttgtatttgtaacttttt

ggttaatagtcctaaagggacagtttttttgtcatcggtggatacttctaatatgtccaagactgctgat

aaggtatttgagatgttagatgccattgtggagaggattggggaggaaaatgttgtccaagtagtcaccg

ataatgctgcaaattataaggcagcgggacaattattgatggaaaaaagaaagagtttgttttggacacc

atgtgctgcccattgtattgacttgatattagaagattttgagaagaagttagaggttcatcaagtaact

attgctaaggggaggagaatcacctcatatatttattcaagaaccattcttatttccatgctaaggcact

ttacgaaaggaagggatttgattaggcctgctgccactcggtttgctactgcatatttgactctaggatg

tttgaatgatcataaaatgcagctgatgactatgtttacttccaaccagtggagttcatgtaggtttgca

agaatagaagaagggaaacgaattcaaaattgtgttttggacagcaggttttggcatgatgttactatat

gtattaaggcagcgtttcctctaattaaagttcttcgattagttgattctgatgagaaaccagctatggg

ttttatatataaagcaatggatgaagcaaaagagaagatacaagtgaattttggttctgtgaaaaaaagg

tatatatcttgctaatttttatttcaaatgttgtcatctattaatatgaatgtctttcaattgagatatt

cctatattgtaatgatatttttattgtttttttttatatagttatatacctatatggaatattgttgatg

caagatgggaacttcaactccacagacccttacatgcagcagcttattatttgaatcctcattatcatta

taatcctaattttaaggttaatgccaacattaaaattggattatatcaatgcttagaaaggatggtgcct

aatgcaagtgaaaggtgcaaaattgacttgcaacttgaatcattcaaggatgcaaaagggttgtttggca

ttgasgctgccaagacagcaagagataaaaaaaactccagctcaatggtgggattcttatggggatgaat

gtccagaattacaaaggtttgcaatccgagttctaagcttgacttgtacttcatctggatgtgagcgtaa

ctggagtgcatttgaaatggtgagttttttttgttttttattttaaatgttgaaatatttgataaaaatc

ttataaatttgaattgtttatttaggttcatacaaaacgaagaaatcgtttgcaccagagaaaaatgaat

gacttggtatttgtaatgtgcaatctgaaattgaatgataaccaagtcaaaaagcaagctgatgattttg

gtgtagaagatgatctttcatctgatgatgattggataaccgagggagaaaaacatccaaactttgattt

gcttggtgctattgacagtgcaacacgaagacaaaatggtaatgaagatgaaagtgatgaagaagaaatt

cctaatgatgctgaaatggagagtcatggtactgaagatgatttggagattcaaattgatgatattggtg

ttggtactagtagtagcactaatgttcataatattggtgttggtactagtagtgacactaataatcctct

tgatgctaatgatattgatgaatgcctgaggaataatgaggaagatgaagggaatgaagctggttttagt

ttacatgacacaccagcagattgtttgttttaagtttgttaattattagctaatgaaaagttattttaaa

ttatgtatgaatgatttttatttgaaccatgtattttaagatgcttgaactatgtatgaatttttatttg

aaacatgtattttaaggtgcttgaactatgtatgaatttttatttgaagcatgaatttttttttaatgta

ttttaaaattccttacgattttacgattttacgatccgtttttacgatccgagtttgtgtgcggctttcc

gtgccgtgttaaaatcgcgattttaacaaccttga

>Pt_hAT-7 Populus trichocarpa

taggggtgttcaaaaaaaccgattaaccgaaaaaaccgagaaaaccgatggaaaattaaccgagaaaacc

gaaccgagatggaaaaccgattaaaccgatttttaaaaccataaaatcttgccggttcggttcggtttcg

gtttgaccaccgaaaccggtgaaccgaaccggtagtataaataacgcagtaaaccagtaagcctaaccct

aattgacattatctgaagccgccgcccacaccaaagagcgaagacgatttatcgccttctcccttctgca

tccgccttgcccccccnccctcttgactcttccccttcaattttcatcctctctgcgtcttgccccctgc

atctgtctcttctctagctgancaataagtcaacaacccacatctccgcatccatctagtatacttgcgg

ccgaaaaaaaacccgaacaaaaccccttggcttctctcttcacttgcagcagaaagagagggctcctcca

ttagagaaaaggcggcaaggtaaagtttatcctcgtgtgcttgtaagttctactttttgtttcaaacttt

gatgcttctctgacctctgcttctcatttttctatcctcttgcagctagggtttttgcatctttctgtng

atttgtgttcattcaggttcgcccccctttccccggccctctctccaatatttctgttgatttgtgttga

tatcgagttactgattgctttgtgttagcattgttaactttgatatgtatcggtttatcttgattgcttc

gatctgtattatcggtttttttatgctgtggtattatctattatttaatgcctgtatgtttaacctgttg

ttttatctgtattgctttgtttggctatcaatctattgttttgttgtattaattctgggcattgtttgca

agtatttgagttcgttctgttntctgtatattgatgacttcatatttgttgtatnctggcggtaacattc

attctgttcaatgttcatgttcgatcggtatgaactgttttgtcaaaatggatttggacagtgctaatag

ctttatangagngaggttgcgcgagtatccatgcaggggattgaaattattcaatgaaaatctgaataag

ttaattacagtgtataggccttggttgagtacggtgtgttggattaacatgcgtaaatcgncacgtactt

tcagtgttttgcaatattgctctttccttcatttttagaatagaaattggtccgttacggactttttgca

attgaaattggtccattacggactttcaggtccatngcatgtgtaatttacagtatgcaggttttttaga

ataaagaggtgatagatgcctttttgcaattatatgaacctaacatgatgctcattgctcaacttctgct

tgattctcaattgtagtcaaatgttgattcatgttgttgtttttttaatttcagatggataatcgggaag

atcctacaccgaatgaatcaaatccttcaagttcggaacccaattcttcgcctattccagttccagttac

tacctctagtacaaatagcaacactgaagaaggtaatccagcttctagatgtaataaaagaaaaacatcc

caaatttgggatcactttaaaaaactagatggtaatcctaaagctcctagggctgcatgtatgtattgtg

gaaaagactatgcatgtcatactatacttaatgggactagtaacatgtggagtcatctaggtgtatgcaa

aaaatttccttttgtgattgaccgaaaacaaaaaactttagttttagaacctaagcctataatagagggg

ggtgataatggagaggaaaatctggtgactattaaggcagtgggttataattatgaagagtgtaggaaag

ccctaggaaaaatgattatacttgatgagctaccttttaactttgtagaaaaccaaggatttaaatcatt

ttgtcaagtaatgcaacctagatttgatgttccttctcgtttgacgatttggagagattgtttgaaaatt

tatgtagntgagaaggaaaaattaaagaaagctcttaaggatcaacgtttatgtttaacaaccgatactt

ggacatcaatccaaaatattaattatatgtgtttgactgcccattggattgatgaaggttggaacttgaa

taaaagaattctaaacttttgtcaagtttctaatcataaaggtgaaacaattggccaagcgattgagagt

tgtttgttggaatggggaattgataacattttaacagttacagtagacaatgcaagttcaaataatttga

caataaaatatctgaagagagtaacaagtggttgggcaactaatatattgtcaaatgacttcatgcatgt

tagatgttgtgcacatattgttaatcttattgtatgtgcgggattgaaagatattgatgattcagtggtt

aagattaggaatgcagtgaggtttgttagatcttctccttctagacaacttgtttttaaccaatgtgcgg

agaggttgaaaattgggagtaaaaaatctgtttgcttggatgttgcaactagatggaactctacatatat

gatgctagatgcggctgntaaatttgatgtggtttttatgaggttagaagaaaccgatcctaggtatttg

agttactttgaggttgattcaaaagggaaacaaaaaaacttaggtcctcctgctttagaagattgggaaa

aggctagatcttttgtcaagttcttgaaactattttacacggttacattgaaattttctggctcgttgta

tgtgacatctaattctttcttccatgaattgatttccatgcacacaagcatatctcaactttgtagaagt

gaagatgtttatgtaagtaaaatggccaagaatatgatggcaaagtataaaaaatattggggggatcaag

atacacaaaactttttgttgtatgtggctgttgtgttagatccacgtttcaaattgaagtatgtgagatt

ttgttttggaagattgtatgatgttgaagaggctgaaaattttacaattaaggttaaagatactttgcta

aggttgtttgagcattatatgaatgttgatgagaatgttgaggttgttcatagtgttggaactagtataa

atgaagatgttaatgttgatttaatggtggtaaacgatgatatgttggatgacttagcttctcaattcaa

aaaacatttagaggaagaaggcggtgtacaaaaaaaaaatgaggttgagaggtatttgggtgatgattgt

gaggatcctaatgattttaaattagatattttgggttggtggaggcgtaatgctacaaaatacaagattc

tttctaaggtggcacaacacgtgttagctatcccggtatctacggttgtttctgaagcagctttcagtac

tggcggtcgtatattagatccatttcgaagctctttatctccatcgacagtgcaagcacttgtttgttgt

cagaattggttgagtttagcaccaattccaatcaacattagaaccttcatggattatattgaaaattccg

agatgattgagtcaggtaatttctcttacaaatttaaatgtagttattttataatatcgtttatttacat

atgtttaatctaacattttaatttatgtttttgtagaatttggtgaaagtttaaaaatctcaactgattg

tttgtaagtagtattgatgacgttggtgcaatcctggaatataattgttttgggtaattaatttacttta

ccttgttaattccctttaaaattttgcattgttaattcttttaatagtcgaactctaatatgttgctaat

ttgctagtgtttctttatagcattatccttgctggatcttttggcttttgccgttgctggaagttttgga

tgctggtgttggatttttttcttcaaactatgccggatgtttatgataatttattgctggtttgctaatg

tatccgtttgctaatgttaacttttctagtgcgaacacctcttttaatttgaggtgttcaaatttgatca

tgcatcttgcatattgcattgatttgaattttcatagcttatttctttgcatattaaagatagcaagata

gaacatttcaaatttattgttactataagcatttcaaaaaaaattcaatattttaatttattaaaaagct

tataacaaacttaatcaaattagaaaattttaaagagttttgattaaaaagcctataaacttagcccaac

aagaaaatttaaaagcccaatttaatctaagcccatttaaaaaaaaccaaaaaagcccattaatganatg

attaggtttcccggttttttagcnaaaaaaccggatttaaccgaaccgaaccggttcggtttgaaccggn

ttccggtccggttcggtttgaatttttaaaaatacgagttactcggttcggttggtttttntagtccgaa

ccgaaccgtgaacaggccta

>Pt_URR1aL Populus trichocarpa

cactgtatggggggacactgtaaggggggacactgtgtatgggggggctttgtgtatggggggtactgtg

tatgggggggcactgtgtaaggagggctactgtgtaaggagggctactgtgtatggggggcactgtgtat

ggggggcactgtgtatggagggtactttctatgggggctactgtctgtggggcaattgagggcatagtct

atggggtcaattgggggcactgtctatgggggggtactgtttatgggggcaaatgaggcactgtgtatgg

ggggcactgtatgggggtgctgtctattgggccatcgtgggtactattttaaaaatggggtgtggcaatt

ggggcgtggctgcgcgcaccaggtctttttgtgggggtcaccacaacatgaggaaatgtattaaagggtc

gcggcattaggaaggttgagaaccactg

>Pt_URR1L Populus trichocarpa

cagcgcttctcaacctgtgggtcgggacccctttgggggtcgaacaaccctttcacaggggtcgcctaag

accatcagaaaacacatatttccgatggtcttaggaataattttatggttgggggtcaccacaacatgag

gaactgtattaaagggtcgcggcattaggaaggttgagaaccactg

>Pt_Charlie3L Populus trichocarpa

caggggtccccaaccaccgggccggggaccagtgccgggccgtgggctgtgctgaactgggccacctctg

gtcctaaatacctgatatttcaattctccaatgtgttacacagccatgacaacagcaatgtgaagccctg

gaagctttctactgattgcaacaagttccatactaagcatcagaggatgtcaacccttcggtgggagtaa

gaaaagcaagggtgctgggcgcatctagttgcagggaaacaagctcaggtcccccactgagtttgcatta

tgatgagctgtattatattttataatataataaaataaagtgcaataataattacatttacattatatat

gtaataaccccccaaccacaaccccggtccttggaaaaattgccttgcttgaaactggtccgtggtgcaa

aaaaggttggggaccactg

>Pt_Chap4L Populus trichocarpa

cagtgctgtccaactggcggcccgcgggccgcatgcggcccgcgacccccctctgtgtggccccccacct

gtctggctgctttgatggcttactcttgtgtaagctttaaatggtatcagtactgtgattaactgccccc

ctgcatggttctcacctcagattcaggctgtaatcaggctgtattgtttaaatatgtaaaacctgtgttt

ttcacaccttttagtttctgtattgttcaccccctgcagtgttcacacctctggctcaggctgtaatcac

ccccattgttcccctgttcacacctcaggagcagtagaaacccacaaataatccctgcacactgcaaaaa

gaacatatactgaggtggtacttcaattaaaaagttttttaatatatagttattttgcagactgtaggag

cagtgccagcattgtgtcactgtaggctgcctgtgtgtgccatacacacaggcatcatagggcaagcaga

gtatggcacacacaggcagggtagggaaggcagagtatggcacacacaggcagagtatggcacacacagg

ccaagtatggcacaaaccagccaagtatggcaaacacagtgagagtatggcacacgcaggcagggtaata

aaggcagagtatggcacacacaggcagggtagggcaggcagagtatggcacacacaggccaagtatggca

caaaccagccaagaatggcaaacacagtgagagtatggcacatgcaggcagggtaatgaaggcagagtat

ggcacacacaggcagggtatggcacacacaggcagggtatggcacacacaggccaagtatggcacaaacc

agccaagaatggcacacacagtgaaagtatggcacacagacagggtagggaaggcagagtatggcacaca

caggcagggtagggaaggcgagtatggcacacacaggcagggtagggcaggcagagtatggcacacacag

gcagggtagggcaggcagagtatggcaggtttttgctgtactacaaccattaatatgggtatggtcatgt

gataacatgggtgtggtttcaagtaggtgcggtttcaaaaaggggagtggtcaaaacgggcttccattat

cggccctccaccacgtaggtcggaaaaattccggccctcggtacaacagaagttggacagcactg

>Ta_HAT-1 Triticum aestivum

aaattcattgttcaaaatatcagccgatataccgataaatcggtcgatttatcgcttatcagggtctaac

cggtaagataaatgcatatccctggtgtttatcggtctggacgatttatcgactgtcagagcgatttatc

agcagtcagagcgattttggccggtaaatccaggggggccacctgtttgaaatcacaaaaaaatctcctg

tagcgcaccccttccccagaaccctcgcacgggacacatccctcccgagtcccgatagggtttggcgttt

gggcttcgccggcagccactccccgcaattcaccatgccgccgccaccatggtctcctccggtggccgga

cctagctcgcccgcccctcacctcgtccccctgtccagccccaccccgcacctcgtcggctcgtccccgt

ctccagccctaccccgcacctcatccctcctctcatccccgtctccagcattggccagccgcgcctagtc

cttgaggattccacacggcggcgccccgtccttgaggcttccacacggcggcgcctaggagcgggacgtt

tggagcagctccccgcccacacccctccccaggatccagcagcggcgtgcctcaaccccccgccaaccct

tcgtccacgatcgagagcagcagaagctccacacgccacagcatggattcttctcattcccgattctatt

ctctggacaagtagtgatgcttgcttgttgctttattttggttgagttttgtagatggctgattgtatcc

ggtagattttaattatattagtcaattattgcttcattagtaaactatgtagatggctgatacatggttg

tttcctttatttatgtagatgctgatgcatggttatttccttttatttatgtagatggctggttcaggtt

catctcaacttgcaagttcatcacttaagaggaagtcggatgacatggcttgggagtttgccgagttgat

tgatcctagtgatccgcaaaggctaaagtgcaagctttgtggaaaaattatgtcgggaggggtcactagg

atgaaggagcacattgggcagatcaaaggcgctgtgaccagctgtatgcaagctacacaagattagatta

ctcgagcttttgcatcacacaaggcaccaaggaggaagaaattggcaaagcaaaagcgcgaagaagaagt

taaagttgtgatcaccatagaggtcaacgaggatgatcaagaacagctgaatgaagttggtgaaggtact

ccaaagaagggggcaatggatcaatatcttgctccaattgatccttcgatcccattgaaatataaaaaat

agtatactataaatgatagtgttgataaggaaagaagctacaaagttaggcaatatttggctagatggat

gtacaagaaaaaattacccttcaatgctatcaatgatgatgatttcaaacagttttgtgaggctcttggt

cgctatggtcctgattggaagcaaccctcgcaatatatgatcagggagaagatgttggtgcaggaggtgg

aaaggactagggatttgttgaagccacatgaggtcgagagggcagatacagggtgctctgtcatgactga

tgcttggacggacaagaaaaagaggagcatcatgaacttgtgcgtgcattgcaagttggggaccgtctat

cttggatcaaaggaggtgtcggccgatgcacatacaagtctatacattttcaattatgtggatgaatgca

ttgagaaaataggtatgaatcatcttcaacttgctctcattcgtttcttttcaagtttattacttgctga

attttagtacttgctgaaaattacttgcttattgtgttttgtaggtgctaaaaatgttgtgcaagtagtc

atggataatgcttcaaacaacatgggagaaaaggtgatgttgaaagataaaaggcccaagttattttgga

cctcgtgtgcaactcacaccatcaacttgatggtagaagcggttgcaaagttgaagcattttggttctac

aataaccaaagcaaaagaaatgactactttcctttatgcacatcatactacattggtcctgatgaggtcc

tatacaaaaaagagagacattgttagaccgggggtgactagatttgcttcggcatttcttaccttgcaaa

gtcttgatgctaagaggaagcaactaaaggaaatgtgttgtagtgatacttgggaaggatgcaagcacac

gagaacaaagaaagggcaggtggctcatgccgcaataatgagtagggcattttggaaaaatgtgtctctt

tgcattaaggtattgtttactaaacttcaaattgtgcttgtgtatgttggaatgcatgtgtatatgttaa

atagtaatgttgtgcacttgtgcttgggtatgttggaaacttggaatggaaggtttttgagccattggtg

aagatacttcggttggctgatggcgatggacaatccatggcctctatgtatggagaaataatagaggcaa

agaaggcaatattggttgcggttgataattcggacaaggactataaggcgatcacaacggccatggagag

caagatgaatgggaggctagataccccattgcacattgctgcatatgccttgaatccatattatagttat

gctaccacaagcatgtttaccaatgtggaagtcatgtccgacttgatggaagttattgagcaattttatc

tcgatgatgatgagaagcaaaacaaagtgcttaacattgacttgcccaaatttaaaaagaaggaacgcat

gtttgggaaggtggttgccaccaaagcaattagcaatgcaaatttcaatgccggtaagcttgttctcaag

ataatattgtcatattgcaattatgaattttttgggctgctgcataatactttgcatttttcctttaaat

gtaggggagtggtgggcaacttatggactacaaactcctacattgatgcacattgctttgaggatactca

acttgaccacaagttcatccggatgtgaaagaaattggtgtgtttttgaacaagtaagtctcaacttgca

aatactcaaatctgattcgtatattatttttgatagaatgtaaatcactaagcttcctttcgcaattttt

aggtggatgcaaagaggagaaataaactagacgtgcatcatagggacgatctagtttatattcaattcaa

tggaagaatgatagacaagaggaaggagtactcctcatcttgtgatgttcttcttggtgaagatgctttc

atggcacaagattggatatgtgaaggtgcttatgttgatgccatggaggaggttgatgcaatgggagctt

ccgagtttgttgagctacatagaagttcaagagtgagagaacttcatgaagtggaagaatttgtttccga

tggggaagaatctgatcacgggcttgtcaatgaggatgacatagaattcaagtccgatgatgatggggtg

atacaaggcgccaatgaagatgaggagggggacccgatggagccttaatggggcttcaagccttcaatgc

tttgcactatgtgtgcttttttatgtttgctagctatttatttcagttgtgacttgtgagagaactatga

accactatgtgtgctgtttgctagtttcctattttgctttgtcatgcaatgatgcaactatgcaaagcta

tggttatttatgaccttactatatatgtaagtatgtattgctggactatgtttatttctcattgtttgtc

atgtgtgtgcacctacattttgcaatttatttgatttttgaaggcttggagcacaactgatggtacatgg

tcatgtatgtccttgtgcgtgtgtgtgtgtgtgtacagtttaatatactaatatatcattttttaaaggc

ttggagcacaacgtgatggtagatggtcatgtatgtgcttgtgtatgtgtgcactgtgtaatatattaat

atagcacattttttaaggcttaagaggacaatttatttgtcatgtgtgtcggtgtgtgtaatattatagc

atattttacattattttggttgggaaaacctgagatttaaaaaaaaacttgccgataaatggttgacaga

taaaaacgataaatcggccgataagccgatttatctcttctcttgggtcgatcgataagttaacgagaag

cgatatcccgaaca

>Zm_AC Zea mays cagggatgaaagtaggatgggaaaatcccgtaccgaccgttatcgtataaccgattttgttagttttatc

ccgatcgatttcgaacccgaggtaaaaaacgaaaacggaacggaaacgggatatacaaaacggtaaacgg

aaacggaaacggtagagctagtttcccgaccgtttcaccgggatcccgtttttaatcgggatgatcccgt

ttcgttaccgtattttctaattcgggatgactgcaatatggccagctccaactcccatccataaccactg

aggcccagcccatgtaagaaatacctagcgaacgctgctctgcctctctcccaggcggccaggcaccaca

cgagtaacagcatcacacattcacacgccgccacgcgcccacgccggagtccggacgccgccagccgcac

gccgacgccggcgacgcgtctcgctctcgcctgctctctccgactctccctgtctcccagccggccggcc

gctgggctgcaccaggcaccacacgcggtgacggccgtgacgcggcacgccggacgcagacgccgccatc

cacggtccgccctccactccactgctcgcgactcgcccatccgcgccgcggtccgcccatccgcccagac

ctccactccactgctcgcccatccgcggtccgcccatccgccatccgccatctgcggtcagcggtcctcc

agacctccactgctcggcgctcgcccatccggccatccgcggtctccctgtctccacggctgctcacagg

ctcacagcacttagcagtacagcacgtcagcaccattgcaccaagctgttgtgtcatttgtgtgctgtcc

aggggctctgcaacacctgctgattgctgtccagccgtccaggtgctcacaagtcacagcagtacagcac

caagctgattgctgaacacctgctgtccagggctctgctctccacttcggctagccggctacgactccat

tcctcagatgacgcctccggttggaaataatcctccctcaggctcagccataagattggccaagttgatg

tctaccacaagagcgccttctactcgcaaaacaaattccgtattctctgcatatgctcaaggtatatatt

agaaaaacagtagcaatagcattagcattactaattggttgtagattgggaagcatcatattgactgtag

aataatacgaaaaatctgtttataacagggttgaaaagaaaagctgaagcctcttctagtcggattcaga

atgtacgtgcacgtgcgcgtgggcatggatgtggccgcacatcaccatcatcatcaacagctgaggccga

gaggcattttattcagagtgtaagcagtagtaatgcaaatggtacagctacagatccgagtcaagatgat

atggctattgttcatgaaccacaaccacaaccacaaccacaaccagaaccacaaccacagccacaacctg

aacccgaagaagaagcaccacagaagagggcaaagaagtgcacatcggatgtatggcagcatttcaccaa

gaaggaaattgaagtggaggtcgatggaaagaaatacgttcaggtatggggacattgcaactttcctaat

tgcaaggctaagtatagggctgagggtcatcatggaacaagcggatttcgaaatcacttgagaacatcac

atagtttagttaaaggtcagttgtgtctaaaaagtgaaaaggatcatggcaaagacataaatctcattga

gccttataagtacgatgaagtggttagcctaaagaagcttcatttggcaataatcatgcatgaatatcct

ttcaatattgtagaacatgagtactttgttgagtttgttaagtctctgcgccctcactttccaataaagt

cccgtgtcactgctagaaaatatatcatggatttgtatttggaagaaaaagaaaagttgtatggaaaact

aaaagatgttcagtctcgcttcagtacaactatggatatgtggacatcttgtcaaaataagtcatacatg

tgtgtcaccatccattggattgatgatgattggtgtctccaaaaaagaattgttggcttttttcatgttg

aagggcgccacactggccaaaggttatcacaaaccttcactgcaatcatggttaagtggaacattgagaa

aaaattgtttgccttgtctttggataatgctagtgcaaatgaagtagctgtgcacgatataattgaggat

ttgcaggacactgattcaaatctagtttgtgatggtgctttctttcatgtgaggtgtgcttgtcacatac

tgaacttggttgcaaaggatggcttggctgtaattgcaggaacaattgagaaaatcaaagcgattgttct

tgctgtaaaatcttctcctttgcagtgggaagaactaatgaagtgtgctagtgaatgtgacttggataaa

tctaaagggatctcatatgatgtctcaactagatggaattcaacctatttgatgttgagggatgccttat

attataagcctgcactaataaggcttaaaacaagtgatcctcgcaggtatgtttgtctcaattgttgtac

atgtcatcattataaattctcaattaatcaaatgtcaattattgtaggtacgatgcaatttgtcctaaag

ccgaggagtggaagatggcattaactctttttaagtgtttgaagaagttttttgatctcactgaactcct

atctggtactcaatattccactgcaaatttattttacaaaggtttctgtgagataaaggatttgattgac

caatggtgtgttcatgaaaaatttgtcattaggagaatggccgttgcaatgagtgaaaagtttgagaaat

attggaaagtgtctaatattgcactagctgtagcatgcttccttgaccctaggtacaagaaaatattgat

tgagttctatatgaaaaaatttcatggtgattcatacaaagttcatgtagatgactttgttagggtcatt

agaaaattgtatcaattctattctagttgtagtccttcagctccaaagacaaagacaactactaatgata

gtatggatgataccttgatggaaaatgaagatgatgaatttcaaaactatttgcatgagttgaaggatta

tgatcaagtagagtcaaatgaattggataaatatatgtctgaaccccttttgaagcatagtggtcagttt

gatattttatcatggtggaggggaagggttgcagaatatcctattctcacccaaattgcaagggatgtgc

tagcaatacaagtgtcaactgttgcttctgagtctgcgttcagtgctggtggtcgtgttgttgatcctta

ccgcaatcgtcttggttcggagattgttgaagctttgatatgcacaaaagattgggtagcagcatctaga

aaaggtgaatgcatatatgttataatgaagttccaatttatagttattcaacaattattttacttatatt

gatgcatatttgtgtcattcaaggtgctacatattttccaacaatgattggtgatctcgaggtgctagac

tctgttattgctgctgcaacaaatcatgagaatcatatggatgaggtatttaaagattattatttacttc

gtgcatgggctattaatttgctattattcactactgttttgatgcatgggctgtttgctgtcgccttgtt

ttgatgcatgcgccttgctgcccagccgtgttatactccctgcatggctggcattaacagatttttgatc

tcactgcatgcgccttgtcgccttgttttgattggctgctagctgctagctgttaggctcccagctgtta

ggcgctagctgctagctgcctagctcccagccgtgttagttcacagattcatgttctcctatatgtattt

atttatactccctgcatatcaattcatgttcttctatatgtatttatttatccaaaactgacttattttt

gtgtattaacaggatgaagacgcaatagaattttctaagaataatgaagatgtagcaagtggctcctctc

catgagcaatgtgtcttatgtttgttgacagatgagccttggttgtaatagtttatgcatgctaagtgat

ccagatgtgagcaagtgattatgaatatgtgttttaaactttatattgtgtcatgtgtgctagtagactt

atatggcttcttatgttagccaagagcccaagacttatcacttatgtgctacattaaactatgtgtgctc

cagatttatatggattttatctatgtttaattaagacttgtgtttacaattttttatatttgtttttaag

ttttgaatatatgttttcatgtgtgattttaccgaacaaaaataccggttcccgtccgatttcgacttta

acccgaccggatcgtatcggttttcgattaccgtatttatcccgttcgttttcgttaccggtatatcccg

ttttcgtttccgtcccgcaagttaaatatgaaaatgaaaacggtagaggtattttaccgaccgttaccga

ccgttttcatcccta

>Zm_DS Zea mays

tagggatgaaaacggtcggaaacggtatttattcggtaatcagttttttggtcgtttttctttgattgcg

aataaataggatatataatgcactatacaaatttgtattcttgtttataacattgagcttgtaaagattc

ataaaagttaatcctcaaattcatcatatattttctcaaatgatagatataaaattcggtatggattcga

aaacaaattcggtaattttttcaactttttttgttgtagggagcaaataatacataaaacaatttatgta

atattttattcttatttgtaataatgtgcttgagaacataacacaagatcaccatcaaattttatacata

tctattttaaaatattaaatttgtcctaacagttcagattaccactttcatccctacccgagggt

>Zm_hAT-10N1 Zea mays

tatagatggccaaacgggccgcccggcccggcacggcactaagcactattaggcacggcactattaggca

cgacactattaggcactattagtaatcgtgtcgtgccgtgccggcactagtgcctaaccagcggcccagg

cactacactatagtgcctaatcgtgtcgtgccggcactatacgacactaacacctaatagtgcccgtgcc

agcccagacactatttcattttaaaagctcaaaaaatatagaagatctttaagattatatatataatttt

aaaacttgaatatttataccattataaagtgaattcattatgtttaaaatcataacaaataacaatttac

aatcacatgcacatatcacaatcacatctataaaccacatgttggtctaagtcgtgcctaaccgtgccgg

ccacttaatcgtgtcgtgctcgtgccagcccatcgtgccggggtggcggcccaagcacggcactagactc

gtgtcgtgccggcactggcactatatgaatcgtgcccgtgtcgtgccgaagcactatgggccgtgccgtg

cttagtgccggcccatttagcacggcccatttggccatctata

>Zm_hAT-14N1 Zea mays

caggcccggccctgagggtaggcagggtatgccgccgctcagggcccccaaagttagtagggccccagtc

cgacctaaatagatatatactcttatatatttattctattcccaaaatctctagccgctgctcgctggta

accattgctaaagatattgcaaatgatatgggtgtaaatccatcatttccaataaagcgtaaagcggtga

gaaagaaacaatttgatgaaagtagttgccatgaagaaattctagaaaatgagagagcttttgaagtcaa

ttattttttggtattagttgatatggcgatcgcttcattgaggastagatttgaagaactctacatattt

aaaggtatatttgggttttttattgagctcaagcacactaaggtcattaratgatagagaacttaaagag

tgttgcactaaatttgcaaacactttctctcatgatggttcatgtgatgttgagttaaatgatttgattt

ctgaattgaatattctaaartttactttgcctaatgactcaatgtctgctctggagatttttgagcatat

tagagatgcggattgttatcctaatgcctctattgcttatcgaattttatttactgtgcctgttactgtt

gcatctgctgaaagaagcttctcaaaattgaagttgttgaagaattatttaagatcaacaatgactcaag

aaaggttgaatggtttggcgactttatgtatcgagaagaagctattggatgatattgatatcgatagtat

aatcgatgattttgcatcaagaaatgttagaagaaattttataagatgagaagtgacaagtaaattttta

ttatttcagctaatgttattttataaataataatgtgtaatattctatattacagtaacttatatacaga

tttctttactgatatatatttagtagatgtgtagatkatatatatatatatatagttatatggggcctct

atataaatttttgcttagggcccccgaaatgtcaggaccggccctg

>Zm_hAT-14N2 Zea mays

cacggccggccctgagggggtgcggcgggtgcggccgcaccgggcccccaaaaacagagggcccctctgg

ccactggacgttagcacttttttccgctaagcatatctaccctagacataaatacgcaagagcgatgcac

tatcgatcagtttcgtttagattgttaaactaatgtctcaattacttatctaatattcacttcgttccaa

aatagtatttgttttaactcttgatttttatgtctatattcaactagatgatgaggaatcaactagagaa

gcaaaataaattctattttgatacagagagagtattatttactatacacatgatggttgcatcgacggag

agaagatttttaaaattgaaatattttttgaactatttaaggtcaacaatgacttaagagaggttaaatg

gtttggcgattatatgtatctaaaagaagttgttggatgagattgatcttaatggtataagcgatgattt

gtatcacaaaatgttagaagatatttttaatgacatcggataacaagtatgtgtttttgctatattttat

atttgtcatcttataaacttgaaaatagtgcataataatttgtatacgttgaatattatatatattgtgt

gtggatggggcctccatttcggatttcgcaccgggcccctaaaaagtcaggaacggccctg

>Zm_hAT-15 Zea mays

tatacatgtccaaaacgggccggcccgtggtgcagcccggcacccggcacgcccaagcacgggaaaagcc

cggcccggcacgacggccggcgtgcccgtgccagcccggcccggtgcccgtgccgtgcttgggctgccak

gtgggcccgtggtgcaggcacgagcccggcccacgtaagccgcggcacggcgccggcccgttaagccgta

taaggcgtcggcggcgtcgcgtaaggagtacgcgtaaccctagcatcccatttttctgtctcggcctctc

gccctctccgtctcctgtgggctcgtgaccggcgacaggcgactcggctccgccgctccggtgtcctcgc

ctccggcctccggtgtcgacggagcggtgaactccggtgtcctcgcctcctgctcgctgctcctctcctc

tcttgctccatcctcgctcctctctccctcgaccctcactaaccctaaccctagaactcgatcttcgcca

agctcgctcctctgcctctgccgtcagccatccggttggtggatccggtgctccggtgttgcaggtaagg

ttttcttgctcttgctctcgtctctctcttaatctgattcgtcttcttgtgcttgatcttgttgcttcga

ttcgtctaggtgtgcaccgtgtgtgtgcgccgcgcgccgtgaggagccggagaagccgaccgaggcggtg

gccatgtccacggccgatgattcggctgacgacgagctcagtctcgaagcagtggtcgaggagcggaggc

ttgaagcgcagaacgaggcagacgatgcccggtgcctcctccttggtaccacctccgctggtgccatcga

tctggacggcgccggtgcacaaacggggacgggctccacaagcccgacggccgacgcgagcacctctgtc

cctgtgcctggtaaacgttctaggaggagaggtccgacctctaaggtatggctgcactttgaggaggtaa

ctgcgatgcaaaatggtaaggaggtaagagtatctgccatttgtcttcactgcaagaatagcatgtctgc

taaatcttcatctggaactggccatttgatccgacacctggatgtttgccctgctaagaaagaaaaagac

agaactggaaaaactcagtctttgcttaaatataatgctgatggatctgttaatcactgggagtattccc

cttctgttgctagaactgagctttgtcgtttaatagctaggctagacctgccactttgttttggtgaatc

tagtgcttttcaggagtatataacycrtgcccataatccaaggtttattaaatcttctaggcaaactact

gctagagacttgattcgactgtttaatgatcgtgttgaacaactkattgaagtacttaaacatgtttcat

ctgttgctttgacatctgatatatggtctggtaaagctaaagaggactatataagtgttgttgctcattt

tgtgaactctgattggtgtttagaaaagagattgcttggtcttagacctatagaggtagctcatacaggt

cttaacattgctgagcgtgttgaaatggttgcaaatgactatagcattactgataagatttttgctattg

tgcttgataatgcctcatctaacaaaactgctattgatgttctgaaacctgtcttttctggttatattgg

ccatttgatacctgcgcctactaggaatgagagtgatttgagtaatatttttttgcatcagcgttgtgct

tgccatattattaatctgattgttaaaagttgtttgaaacgtttgcaaccatatcttgaagactttagga

ctgctataacatttttaaactcttcaaatcaaagaattgcttcttataaacaatattgcatgagtgttgg

tgttagaccacgtaaatttggagtagatatggatgttagatggaattcaacattyctgatgcttaaacat

ttagttccttatcaaagcactttctctgtgtggattagaactaatyatccttgcaaagatgatgggtctt

ttttactgagtgataatcattggtytattgcagaaaaattgttgtcttttcttcagttattctatgattc

aactgttgcactatctggtgtttattaccctacatcacccttaatgttgcatcatattttgaaaattgct

agacatctaaatgcatatgagaaccatgaactkttaagaaatgctgttgttcctatgaaawcaaartttc

tgaaatattggagggamatacccattctttatgcatttgctttcattcttgatcctagagcaaagatgag

ggggtttcataaagttcttcaaagattatctactctwaatggtactgattatagtagatatccttcatgt

attmgwaccaaattaactaagatgtatcaaatttatgagrccaaatttggwggagtttgcttgactcctc

aaccacagtcaggtagtggttctagtaaggccacagaggcttgggatgatatatatggtgatgatgaaac

ttatagyaatactggtacaggtggtacatctggtacatctactggtttatctgagttaacttcttacctt

gayagtgacactgaaactaagtttgggcctgatttcaacattcttatctggtggcaacgccataatcaaa

cttatcctattctttctatacttgctaaagatgttatgactgtccctgtttctacaatatcttcagaatc

tactttcagtctagctagcagggtgctcgaggagcggcgacgacggctgacaacagacatggtggaggtg

ctctcatgcatcaaggattgggagttggctgaccagcacaagcaacatactgtggagaaggaaaccaagg

accttgaagcaacctttgaagmtatgtacctagatgatgagcagcaagtatcaccagggarcaaaagaaa

ggaagcagcaccacagagttcaagaagggcaggactatgaacttattctctctgtttctacctatttatg

ttctctacttctcttatctgtgtaatatgctcatatggactgtggactgaacactgaactatgaacttat

aaatactwtaaggagctggctgtactcttttccttcctagggttttctcacgaggtgtgagtttttacct

aggaaggtttttaatgaggcagcattgcactaaggctccaaaccatttaaagactattttgtagcctatt

tgaattgtaatcttgtattctgttaactttgatgwattttgtcatgtgtattatgtaactgggcccgagc

ccggcacggcactaaygggctgaccgtgcccccggcccggcccggcccgtttaacattaggcgtgccgtg

tctgggcagggatttgggcccacgtgccagcccggcccggccygacactggcgtgccgtgcctgggcacg

tccmtgtcgtgccgggtccagccgtgcccgtgccgggcyggcccggcacgmccatttggacatgtata

>Zm_hAT-16 Zea mays

tatagttggccaaatggcactaacacgacgggccggccckggcacggcactaaaaagcacggcactggca

cggcacggcacggtccttttagtgccagtgccgggcacggcccgtacatagtgccgtgcctgggccacca

cttgggcccgtagggtcggcccaggcacggcacggttaattgggctggcacggcgccggcacggcccacc

cagcagccgsytagccgccgcccccggcccccactatcccagtccaccctccccagccgcggccgctgag

cgtgtactcacgctcaccgtgcctcactcactcactcgcgctctcgctcaaaccctaactcgctctctct

cagccgcgggcccgcgggcgattcggcggcgaccggcgagctcccctctccctcgttccctccggcctcc

gcctccgctaagcgctccggtctccatctccgcctccggtctgcggtctccggctctccgcctcggcgcc

tccgctcggactcggatcgatcgtcgctcgtcgtcggagaatggagatcctctccggctctcccccgctc

ggatctgcgcatttgtgaaccgggtaactcagatccataacccccgctcggatctcttgtcttcttctcc

ccctccggtgtgatctgcggagatcccggtgctccggcttattagggtttacacgcgcatttgtgaaccg

gtgcgatctgcgcttctgcggagatcccggtgctccgttctccctcatctctctcctctctcctgtcttc

ttctccccctccggcctccggtgtgatctgcgcttctgcggagatcccggtgctccggcttactccgttg

aggaaccgggactccggggtccggtacggcggcactggcactggcactggtactctcctctctcctagct

ccggtctccctctcctatcttcttctccccctccggtactgtccggcactggcacacagatccggtctcc

ctctccccctccggcactctccggcactgtgatctgcgcttctacggagatctctgtgctccggcttact

ccggttgaggaaccggcgtctcctctctcctagctacggtgctctggtctccctctcctatcttctagct

ccggtctccctctcctctgtcctggctctggtctcactctcttctctcctagctccggtctccctctcct

ctctctcctagctccggtctccctctcctctctcctagctctggtctccctcccctctctcctagctccg

gtctccctctcctctctcctagctctggtgctccggattcaaatccgtggaggaaccggttggccggacc

ggtcgtcccctcctctctcctagctctggtgctccggcttactccgttgaggaaccgggactccgagtcc

gaggtgggtcgatggccgtctccgacgatgaaactgttgagatcgactgcaacgaggagcgacggttgtg

tgggttggccggagatgacgatgaggatgacctccgggaggatgctgcagcgctctttggccwtagcagt

gagcatcccgtccatgtcggtgatgaacaacaagtcggtgatgaacaacaagtcggtggtcaggataacg

aaggtgaaggggacgaacacagtgcaggatcaggaaggtgaaggggacgaacacagttccaagcgttgtc

gtccttctacttctgcagtgtggttagatttcgagaaacttttcaaaatcgtgaacggtaagaaggtaag

gtttgcagctaagtgcatacattgctctaagcaatattctgctctctctagtggtggcactggccacctg

acccgacatagagatagatgtcccaggaggcgtgaaaaaacccgcatgtctcagtctcagatatctttta

atcctgatggtagtatgcgtaactgggagtactgtcctatggttgcacgtaatgaattggttcgattgct

tgctaggctagaaattcctattagtttgggtgaaaatgctgcatttgaacactacattagaactgctcat

aatcctaaatttgtgcctgtctctaggcaaaccaccaccagagacatggttaaatacttcactgacaaaa

aggctaagcttgttgagactctttcttcttctgatgttaattgtgtgtgtttgacctctgacatttggtc

tggtaatgccaaagaggactaccttagtgttgttgcccattacataaaccctgattggcaactagagaaa

agggtgcttgctttagtgcttattgatgtgtcacacaatggacaaaacattgctgatcgtgttgctggtg

tgcttgctgattatggcttgactaataaggtgtttgcttttaccctagacaatgcatcatctaatgctgc

tgccatgaggttgcttagacctataatgtcaccatatcttggtattgataatggggcagattctgaaatg

ttcttgcatcagcgttgtgcatgtcatataatcaatttgatagttaaagaggccctagattctcttaaga

atttgattgaaacatttagaactgcaatatcttttttaaactcctctaatcagagaattgctgcatataa

aagttattgcattgctactaatattaggcctagaaaattccagttggacatggaggttaggtggaactct

acatatctgatgcttaagcatttgtttcctcataggatacctttcactactttcattcatgcaaactatc

ctagggctgaaggtgatcaatttattttaactgatgaacattgggttattggagacaaagtacttaaatt

tcttgagctattttatgattctacagttgcattgtctggtgtttattatcctacatctccacttatgctt

cattttcttgttaagattgctatacatctaaagaactatgctaatgacactcacatcagaggtgtcattc

aacctatgrtagataaatataataaatattggaggaacatccctttactttactcttttgcattcatctt

ggaccctagagctaaaatgaaaggttttagtagagtgcttagaagattaatgaacctgacaagtacagat

tatgctgtttatcaggttaccactagagctaagcttactgatgtttataataaatatgaagagaaatatg

gatctgttagattgaacagggttgtccctcctaacttgtctggtaagaaacggtctgcttgggatgaaat

ttatgatgatgctgatgatgtgggtacttctgttggtatgcattcttttgcttctactctaaacattgct

agagatacctctgcaactgctctactgcatgctgcaagctcttcagcttctactgcttctgaactcattt

cttacttagattgtgacactgtcaaccagttaactgatgatttcaacatcttgcactggtggcatcagca

caaactcacttatccagtactgtcaattatggctaaagatattttaactgttcctgtgtctaccatctct

tcagaatccacttttagtttaactggcaggatcatcgacgagcggaggaggagattgaagtctgatgtgg

ttgagatgttgacatgcattaaagattgggaggatgctgaagcaaggatgcaacacatggtggatgacaa

agagcttgaagaaacatttgaaaatctttatcttgattagtctagtatcttactctctttgtgaccattt

ctgtaatgggactgtaatattatgcacctggactggactttrttgtaaggacaataaactttttaatgag

ctgggctgtactcttttttcctgtctagggtttctcacaagggtgagttttacctagacaggtttttaac

gaggcagccattgcactacagctcataaaattgttgtttgattagtctcttactctctttgtgaccattt

tatgttgtttgaaacttgtatctgtttgattgctagaagtcataataaaattatctgttattctgtttga

ctgtttgaatgctgytgctggtatttctatacatagtgcccgtgctggcactagcactggcgtgccggcg

tgcctgttggcacggcacggcacggttaagcactgrtagtgccgtgcctgggccgagggcccagcacgct

ggcactaacaggcacggcacggatcttytaccgtgcctagccgtgccgtgccctgtcgtgccgtgcctgg

tcgtgctagtgccagtgccgtgccgtgcggcccggttggccatctata

>Zm_hAT-17N1 Zea mays

tagggatgtaatatggttcggaaaatatccgtccggatccggattcgaagatggtgaacactggttcgga

tggaattttcggatatccggactttttttcggataacggatacgaatacggatattttgttcggatatcg

aattcgaatacaatatgtctgatatccgtcggatatcgaatatccggatattttctcggataaatcttct

cggatatccggatatcttatccggatagctgtgtgcattttgttgattttttgtagaaaaaaattaataa

tacacaaatagcctcagaaattcatgaaaatttatggaggcataccatatgtccacatataatcctccaa

aatatttggaccataaaatccacaatatgatagtgtttctttcatttcactttcacttgttgtgtgaatt

acacgtgaaatcactctaagtatccaagttcactttatcattttcatgtggagacttgaggttatattct

tatagaatgtttattagttttggtaacttatttgcattttgatgattttatgcaaaataaattaataaca

cgcacataggctaaaaattcatgaattttacaaaaaaacgtgacatatgtacacatataatacaaaaaat

gtttggactcaaggagtggatacatgattacaaccatgatatgtgtggaatgatgtcttacacgatatcc

gacaaaaaattcgttaccgacaatatccgtgtccgactagttccgtattcgacacacagctatccgtatt

cgtatccgagaacatccgtattcgtattcgtatccgaagctatccgtattcgaattcgaatccgaataaa

aatatgaaaacaaatatgatttcagtgatatccgtccgtattcgatccgattacatcccta

>Zm_hAT-17 Zea mays

tagggatgtaatatggttcggaaaatatncgtccggatccggattcgaagatggtgaayactgattcgga

tagaattttcggattttcggattttttcggataacggatacggatacggatagtctgatcggatgtcgga

tccgaatataatatgactgatatccgtcggatatcgaatatccgcgactttgatcggatgtatccgacat

ttatccgaacaaatatccgacagatcacagcctacgccccagccaaacccagcccacggccacagcccac

atggccaaggcrgcaaggcaacagcccacatggccacactcatggctacagcccacaacgcacatggcca

carcccacctggccacgstcacgcacgcacaagcaaagcactgaaagcaggcaacttcagcacgtaaggc

cgtaagggtacagcggcacgccacacgcacgcacaagccacgcactcgcactgcacagcccgtcagccac

gcggtcagcggcacgcagactcccaattccaaaccctagacctagaggttggcggcgacggcaggtcgac

agctccagccaggcagccagccacgagcccacgacgaccgcggacgtccgctcctcacggcctcacctcc

tcggctcttcatctcacgacggcgcagcctgcaggcgggaggccgctctgatccctcgcgccggacttga

cgacgggtgactgcctgactggtgagtgaatcttccattcttggtccgcttacggcgacggcggtgctgt

aaggtcccataatcgtygacgtttatctacgctggctttgagggattgtatcgtgtcaaagcagttttta

aattgttgtagcattcaaatgactctcactagtcaagtgcagcattcactggtgattaattgttgttaaa

ctgactataacacttgcttttaaattgaatcttaytgatgtagcatggcatctgcaagtggcagtgggtg

cgcaagcactggagcatcccttgttgctgcgaacaccctggctgtggctgtgcaagaaacaactccagtt

aatggagctggagaaggagaagatggagaaagaacaactgatattggagtgaaagagtctgatacaaaga

aaaggaaaccaaaaagacaaacctctggggtttgggactactttatcaagtccactgtgacagagaaggg

agataagggcgagaccgaggaacaaatttgggcaaaatgcaagaagtgcaagttcaaaactcgtggtgaa
[truncated: 374,178 more chars]
