# Supplementary material for: In silico Phylogenetic Analysis of hAT Transposable Elements in Plants
Source: Genes (Basel). 2018 Jun 6;9(6):284. doi: 10.3390/genes9060284 (PMC6027215; doi:10.3390/genes9060284)
Supplement: Supplementary file 1 [file genes-09-00284-s001.zip › TextS2.docx]

>At_ATHAT1

MAQYQINVDSQLLHQLFLGNSQDAGVAKLLESVLNQVLQAQVSEQVEADRYERTENRKAYRNGSYPHGLHTRVGTITLSVPRIRGGKFTTELFSRYQRSEQALILAMMEMVVNGVSTRKVSQVTEELCGTEFSKSTVSDLCKRLDPIVTAWNNRSLADSLFPFVLVDAMYLKVREDGRVRSRGIMIAIGVNTEGYREVLGLMLGDTESEASWSEFFSSLKGRGLRGVDLITSDDHGGLVRAVRQQLQGVTWQRCQTHFTRNVLEASPKALKDEIHGRLRSILDAPDTGTARFLLKQTLAAYEDKAGKAMGVLESGFDDATAVLMLPERYRKRLRTTNSVERLNEEVRRRERVIRIFPNRESVIRLIGALLMEQDEKWAAG

>At_ATHAT2

MEFLSSLSSQSPPSEASSNRSRMDSSPFPSNSDVGNEKVTEPESDAANKRKDVQEDSGATSKPPKTKKPSQRSFVWDHYTRFEDNPKRCKCNYCHRTYGCDSKDGTSNLKNHLRICKHYQAWSQKAKQTVFNNQGHLQSGKVNLYAPHSRRTCMKDIVKMYVGMKASLKTWIATTNPRVSLTTDIWTAKATVASYMVITAHFVDSAWKLRKLIIGFKYITDHKGATIARILLECLAECGIEKIFTITVDNETTNTSALKKFQETFSLRSNEAFVLEGEAIRNSVQYVRSSTSRCDSFDQKVVSGKMTRGSLPLDIKTRWNSTYLMLKRAMEFRLAFDKMEAEDKLYNDYFNEVDDGKARSGHLQELIGMQSRVASYKCYGEIVTIETNLVSLGNNFDRDLKIKAKAMLGKFVKYWDGTRNINVYLIVASVFDPRKKMQFANMCFAKLYGKDTTDAKEMAEKVNNVLTSLFKEYSSRFQKTSSGSGPSSQSTQTSITASQGEPSDLMSDSMGYERMDFAYKELVDEIGVDDGRDELDVYLKEKVENPKTIIGLEWEVLSWWKLNCGRFPVLSAITKDVLAMQVSSVASESAFSNSGRVIEPHRSCLTHYMVKVLLCTEQWMKNVNHLGNKSVVTIKELLADIVELDNIEKEFDGNSA

>At_ATHAT3

AYQHKSQQAGNPNSFVSAVSFFREVKNSTRSEMDSSANPNNTESNTKGNEDEGRTRPIDNSKGKRKSDVADDGDSANPCKPKKKLQTRSWVWDHFTRKDGDDDQCKCHYYKRFFGCSTKSGTSNLKKHLDCCKHYSAWKGRQSQNVINQEGNLQSGKVSKEVFREATNEMLVLGQLPLSFVESVAWKHICSKANLYKPHSRRTATRDIILMNVARKASLKDLVSANKRRVSLTTDIWTAQATGASYMVITVHFIDEYWRLRKFIIGFKYIADHKGATISRVLLECLTEWGIERIFTITVDNATANTSALRKFQRALQSQRADSLVLNGDFMHMRCCAHIINLIVKEGLHKLGNHVEAIRNGVLYVRSSTSRCDSFEQKVVTGKMTRGSLPLDVKTRWNSTYLMLTRAIKFKVAFDKMEAEDKLYNDHFLEVVDGEKRIGPPTTIDWREVERLVKFLGLFYTATLVVSASSIVCSYKCYGEIVTIEKNLLGMTHSYDKELREKAVEMREKFDKYWDGQKNINRMLIIASVFDPRQKMEFAKMCFEKLYGVDTSEAKEMYNSVYDVMKAMLKEYTVIFKGPNTQSSQSNPPSSTAARDTFACELAEDSNVEFERMDRSYKEMVNEIRVTDPKDELDIYLKAEVENPKTLPGMEWDVLSWWRLNSQKYPVLSEIARDVLAMQVSSVASESAFSTSGRLLEPSRSCLTHYMVETLVCLEQWLKSEIKLSENTFLTNAQLLADIELLDKLEK

>At_ATHAT7

MASNETLPPNDFDDVELSGNDDDDVVQHTPTSGNKRKRKEKETGDEGDGGNSSKKKKTSSRSYVWDHFSRKKGNPNKCNCHYCGKELACPSKSGTSTLKKHLELSCKAFKAWKSTNTDQTQTVIGRDGGDGSLTMYKVSELVIREASIEMLILGELPLSFIESVAWRHFCSKAKSYKPVSRRTTTREIVMLYVKKKVAMKKILGKSQERMSLTTDIWVSNNTGESYMVITAHFVDVDWKLKKMIIGFKHVTDHNCGTICKVLLECLAEWDIRRIFCITVDNATANNTALTKFKKTMKLIGDDALVLKGEYMHMRCDAHILNLVVKEGLTEVDASVTSIRNGIQFVRSSTNRLKSFDLRCDAGTISRGSFPLDVKTRWNSTYLMLEQAVKFRIAFEKMEAEDKLYNDYYLEKVDGEKKIGPPMSSDWDAAERLIQILAIFYKSTLVLSGSTYVTSHKMYNEIINMARNLTTLNTDTFFDEQLKKKAIAMLGKLKKYWDPFGEGVEMNRLVMVATVFDPRKKMKFVELCFGKMYGLGSVEVVLLSDSVIQILKDLYDEYSRANLLRINGGSDSMPSSQSQGSWSQSQEQDRSGAYERTINKTGIQLEDMENLFDEIVKETCIHKSSNELDLYLNEAVETPHLLMGIESDVLDWWKLNSGKFLVLSLIAKDIFAMQVSSVASESAFSTSGRVLDPFRSCLTHYMIEVLMCTEQWLKSEISINEKGLSTIHELLADQVDEDELMRGNLFPLNSL

>At_ATHAT8

MLDQALKFRLAFEKMEAEDKPYNDYFAEKEDGNKRIGPAVTKDWENVDRLVQILEIFYKSTLVLSASNYVASHKLYNEIVSITINLGALTYDDDGLKDKAEAMLAKLAKYRDAFGEKVEFNRLVIVASVFDPRKKMKFAELCFERLYGQGSTKATQLQDSVYNILSDLFDEYTRNNLLNKSSTGTTGSSTQSEWSQDQVVNEDSERPVLRNGFLYENMKYEFDEIVKETGLHNTANEVDRYLKEPVEKPIILKGTEYDLLNFWRINNGKYPVLSLIAKDILAMQVTFVASESAFSTSGRVVNPFRSCLSHYMVEVLMCLEQWLKCEIHLNERGVSTIKQLLSEITLEDHLMRSKFRFSSLFLVITS

>At_ATHAT9

MDQQSLERIALLEAENERTEMEMNAEVETQGQNQTESVTQPCQRAKRLRKKQRALCWDEFTSVGIEEDGKERARCHHCGIKLVVEKSYGTSTMNRHLTLCPERPQPETRPKYDHKVDREMTSEIIIYHDMPFRYVEYEKVRARDKFLNPDCKPICRQTAALDVFKRFEIEKAKLIDVFAKHNGQVCLTADLWSSRSTVTGYICVTSHYIDESWRLNNKILAFCDLKPPHNGEEIAKKVYDCLKEWGLEKKILTITLDNASANTSMQTILKHRLQSGNGLLCGGNFLHVRCCAHILNLIVQAGLELASGLLENITESVKFVKASESRKDSFATCLECVGIKSGAGLSLDVSTRWNSTYEMLARALKFRKAFAILNLYERGYCSLPTEEECDRGEKICDLLKPFNTITTYFSGVKYPTANIYFIQVWKIELLLMKYANCDDVDVREMAKKMQKKFAKYWNEYSVILAMGAALDPRLKLQILRSAYNKVDPVTAEGKVDIVRNNLILLYEEYKTKSASSSNSSTTLTPHELLNESPLEADVNDVSIIIYLNIFH

>At_ATHAT10

MSSDSFRSNIGTPIEDVHNSTPAINTQESATEEQANTSSGKQRLNASDIFKVHFRKEKIDGKMKACCNYCNKSFFWKSGSGYGSYRYHLVNNHPEKYGGETQNATSANLSNFHYSDKRNREELAKCVAVDHLSFSFGEKLGFNNYCQKALNPQAKRVPRTTLTRTLKSLYRKTKKRFRMFVFLPNNVSICSDIWSDQWQIHHYMGITSHWLDSDWIIQKRIIAFRVFYERHTSDNIFKLIKIILEEYNLTNKIFSISFDNASANTASIDELINNCSPILGGKYFHVRCICHVLNLCVQDGLLVSQNNLISPIKTALNYLWGHPQLMKKWFRFCKMHNVSPKRFSRDVPTRWNSTYDMLVDSIGYKDLLCSFIQQNCSSLTLWPTHWDDCNALLKLLKCFNDATFLLSGVYYHTSHLLLYECVNIADVMHEHENNTVLSSCITSMRDKWLKYYREIPPLNLLASVFDPRTKFDGLYDYLVAYYDLLHLSDSINVPSIISNSRKDIENLYDEYYRLYEHLVPQGTESSLQNDMSSTSTLSLAERMRRRKRQRPSQGNNAELEKYLSTNFEFSDADAGNNFQIIHWWKSHQSQYPILAMIAKDVLSSPVSTVSVERAFSMGGQILDETRSRMSPDSLEAQACLDDWTRAGYRQQEFLRENEEELEDIDSDVSSTRSEDSD

>At_TAG2

MNRHMRSCEKTPGSTPRISRKVDMMVFREMIAVALVQHNLPYSFVEYERIREAFTYANPSIEFWSRNTAAFDVYKIYEREKIKLKEKLAIIPGRICLTTDLWRALTVESYICLTAHYVDVDGVLKTKILSFCAFPPPHSGVAIAMKLSELLKDWGIEKKVFTLTVDNASANDTMQSILKRKLQKDLVCSGEFFHVRCSAHILNLIVQDGLEVISGALEKIRETVKYVKGSETRENLFQNCMDTIGIQTEANLVLDVSTRWNSTYHMLSRAIQFKDVLRSLAEVDRGYKSFPSAVEWERAELICDLLKPFAEITKLISGSSYPTANVYFMQVWAIKCWLGDHDDSHDRVIREMVEDMTEKYDKYWEDFSDILAMAAVLDPRLKFSALEYCYNILNPLTSKENLTHVRDKMVQLFGAYKRTTCNVAASTSQSSRKDIPFGYDGFYSYFSQRNGTGKSPLDMYLEEPVLDMVSFRDMDVIAYWKNNVSRFKELSSMACDILSIPITTVASESAFSIGSRVLNKYRSCLLPTNVQALLCTRNWFRGFQEVGNIHILNSFIIVNLYGFWTAAFRLIARFFLL

>Cr_Gulliver

MEKRGTKLGANDNVFHPGAKASVWTKGGNKVMNMTLFNKHLLSKNHLDVIGAAKNHTKMSNIVEKQKVKLNPTLCSLIRSAYACGKDAMPLTHYVKMVKLQAANCADCKHDCKAHTCRKCDASKACGSGITISGPYHTAEKASEMLACLSEAISEEQLKNIRASPVISMMIDESTDRTVSHNLAVYITYVAPDDSIKTEFLQLEAMNNGATAVNIYDRLKEVFTESKIDWSKLVAFTSDGANVMVGKHCGVATRIKTDWPCVLTSHCAAHRLALACADFFKEFPALVKVDNMLSKIYNYAKTSTVRTAALNDMYKERKAKAYKILKPHTVRWLSRSECVRRIKITYPILLAFFNERKKDKKDVAAAEIYEWLRQVDNLLLITCIDGVLAATAELSKWFQQSDLALVDVHQYLELGLRHLINAYTYHKEGETSNAPPSFTAPIQALIKDLAAKDGVFHGHQMVLTTPPTTIAPATTTTATTTAESDSDNDGSDTNTDTTAESGSDDDDTAPTAPSWAAFPQTLFRSKLALSSMVKKLVENIKMRFPADVSVAAKFGVLGPRALAADSGVPKYGEEEVAALAQHFKPVLGDDCLLAVDQWVMARARLIAVAKEQKKSGDVMKARPFYERLLSWLSGMGRELTVLVQIMLVLQPSTAEVERGFSAMNDIKTPGRASMKLGTLDVLMRVRLVGPPIAYQQRPVAGVSLAPYAEFDATLLGPAVQKFAAKLGRVPQRSSHNARPSRVKHRVCEIDVKALLKEAEEEAVQNVDVSC

>Hv_HAT-1

MASEPQEIMGIEASAPVPTSTLIVAGAVLTNMEKASVDGELQAGETEKRDGGKAQVVEVEKKDGRRQMVSRSEMWNHFEKVMMDGVLQKGKCNYCKSDISAHPVINGTSALRKHFNICKRNPHRNIGDDKQAVLQINQGDSVHAWKFDPDAIRAAFAEMIIEDELPFAFGEKSGFKKFMSKACPRFSVPSRRTCTRDVVRAFFEQKAKLKLFFKGQCERVCLTTDGWTSQQQDSYMAVTAHFICNEWKLHKKVISFFKVNGHKGDDIGKYLQKTLIDWGIEKVMTITVDNASNNDGGIGYMKKELNKAKTCIAGGKYLHMRCAAHIVNLIVTDGLKELDVSIKRVRAAVRYVKNSPNRLTKFKECADLEKLDTKAFLILDVSTRWNSTFFMLKAAICYEKVFARYADEDPYFAVDLLSDKGKDKGVGVPDEQDWENVKKMADFLGHFAELTTRVSASLHVTANKFFHEIGEVNLLVRSWMESEDGLQIAMAERMKDKFDKYWGNWHELDTSKKGKEKENFNLLIFIATTLDPRCFIYIIAPQIEVGTLLLHLRYKLSEYTQFAILEMYGEDKGPKVCTAVSTCLRDLFEEYRVMYGPNIPSYETSASHEVQSSGGRVSMMKSLIAKKMRLNSGGSSSSKSELDKFLAEEPEDDGPKFDILDWWKINSSRFPILACLARDVLAVPISTVASESAFSTSGRILDDFRTSLTPFMVEALVCTQDWLRYGTYINIAENTEELTKLEQGNLI

>Mt_HAT1

MLQELQSVCASNMRDNDIDSSGSHENMCVDGEIESQAREIPLVPPIDQNAQEVSSDPKDKKRKGKAKAKDKSKGKALTSDVWLYLVKVGIVDGVEKCRCKACHKLLTCESGSGTSHLKRHVRSCSKTIKNHDVGEMMIDVEGKLRKKKFDPMANREFLARIMITHGAPFNMVEWKVFREYQKFLNDDCVFVSRNTIAKEILNVYRDEKQKLKSQLAQIRGRVCLTSDCWTACSNEGYISLTAHYVNVNWKLESKILAFAHMEPPHSGRDLALKVLEMLDDWGIEKKIFSITLDNASANNSMANFLKEHLSLSNSLLLDGEFFHIRCSAHILNLIVQDGLKVVSDALHKIRQSVAYVRVTEGRTLLFSECVRIVGDIDTTIGLRLDCVTRWNSTYIMLQSALVYRRAFYSLSLRDSNFKCCPTSEEWRRAEIMCEILKPFFTITNLISGSSYPTSNLYFGEIWKIECLIRSYLTSEDLLIQKMAENMKVKFDKYWSDYNVVLAVGAVLDPTKKFNFLKFAYEKLDPLTSEEKLKKVKMTLGKLFSEYIKNGIPSNLSSSQVQPSYGGGTRITSSSYDVSSYIFSLFYSFINYL

>Mt_HAT2

MQENMDVDDVPTNAVENPIDVDSDHDLQGEAAGDVGKNRKRSRAWDHFVPDGKRAKCIYCNMTYAAEGNTHGTTNLNKHYKKCPKNPNRVIDKAQKTLVLGKQVEGDSNVSFKLVEFNQLECRMELAKMIIIDELPFKHVEGVGFKGFMSRAQPRLKIPSRVTVAKDCMELYKEEKVILRSLLSLNQQMVSLTTDTWTSIQNMNYMCVTGHFIDEGWELQKKILGFGLIANHRGDTIGKALEKCLKDWGITKLCTVTVDNASSNNVALSYLTRNMSAWNGNTLLKGEYMHLRCCAHILNLIVFDGLSLIDSSISKIRAACKHVKSSPSRLALFKVCVKDANISSSQKVVIDVATRWNTTYLMLEVASKYEEAFNRLEGEDPSYVSELEVVGGTPTVYDWNRARVFINFLKIFYDATLTFSSSLHISANCFFRKLVKIHTALSSWIQGEDVVLKNMALTMKAKFEKYWSDENINYLLFVAVYLDPRYKMEYLDFCFGWMYGVEKAKMIIAKLHELIGKLFDYYKSVQPIGFVSSDSSSSSTNTMQSDVVTFGVGGNVDMDALHRRRVKRRQSEHNSSELVRYLEDEVEDDYEGFEHLKWWKSKSTKYIVLSLIARDILSIPISTVSSESAFSTGGRVIDPYRSSLKAETVEALICTQNWIKPVTRSVLDESKAFDVLEFETGINCFLAFIFLFYLLVLFLHCLFFYVIRNVIFEWSRGVCS

>Mt_HAT3

MSSQANEEPTQAGANTTQAEANATQEEETQAEPIVGRKRKKTSVIWKDFDEKEITKGVFRAVCKHCKAQYTTGTVGSSTSQMKRHLVSCTAKKLQDATEKRQAAIPFKRVSSGNPFLTSGVGYSNERMREIIATAVMVHEYPFNVVEDDVWMWAFEYANPEFRKVTHKTTRSDCLKLFENEKKILKKQLESVSKISLTTDMWKSSHQVVEYMVITGHFIDAGWNLQKRVLSFVKVPAPRRGIDVADAIHKCLKTWRIESKIFTVSVDNAAYNDLCLKYLKDNISMSRKLILNGDLFHVRCCAHILNLLVQDGLSKIKDIIFNIRESVKYVNHNDARLKNFCDVVEQKGLKERKLVIDCPTRWNSTFNMASTALKFKIVFSAYKEREPHYDHAPSFEEWDKVEKVCKLLEVFNSATHVISGSFSTILSFNITILNVGSEYPTANLYLPEVWRVKQVLDMADEDEDLFMREMAKPMKKKFDKYWGESNLLMAIASVLDPRCKFHSVCICFPKIYKSKEVSDENIEKVRRSLELLYDEYVALSLEESYLMPAVNLDNSSSSQTNVKNATGIDDLLQTIREQQAISPTKSELQDYLDQGVHVVPNSESFSALEWWRNNSMKYKILSKMAADILAIPISTVASESTFSAGGRVIDEFRSRLNEESVEALICGGDWFRHKYGVKNKSKVILCHDILLIFIHIFIWYCLVQ

>Mt_RAHAT

MKFQRIDSIFKRKAVDIQKDEVIISSSEPEQVHENPRIEENESRPSKINRVDPDDIENSLERDPGKCIPIYQYPPNQKDAIRRAYLKWGPYQSNLENYPMSGIGKAQRRFQNSWFSLFSSWLEYSPSEDAAYCLQCYLFSNKPSGRLGSEVFISTGFRSWRKVRNGENCSFLKHIGKDPRSPHNNAMKACQDFLNQDGHLRNVIEVQSSSQILNNRLCLKTSIDTVRWLTIQACAFRGHREGNKSRNQGNFLELLKLLASYNDEVAKVVLKNAPEKCNYTSHQIQKEILQILSSRVRKHIREEIGDSKFCIVVDEARDESKKKQMALVLRFVDKAGLIQERFFDVARVNDTASLTLKEAVCGILSRHNLDVSNIRGQGYDGASNMRGEWNGLQALFMKDCPYAYYVHCFAHRLQLALVTASREVASIHKFFEKLTFVVNVVGSSTKRHDELQAAQAKEIENLLETGEIVTGKGKNQVGTVKRDGDTRWGSHFNSICSLISMYEATCTVLKIIAKDAKKFAQRADADSSYNHLKSFDFIFILHLMKEIMGTTDLLCQALQKQSQDVVNAVILVRSTKALIQDLRENGWDKLFANVVSFCEKHDIEVPDLNDCHSTTRFGRSRLEENQVTIEHYFRVEFLFTTIDKQLQELNSRFSEQAMDLLTLSCALSPEDGYKAFDINTICTLVEKYYPMDFSDQEKINLPFHLKHFLFEARESSTLKNLSTIQELCSCLAAAVPANGQPKKHLLLDRLLRLVMTLPVSTATTERSFSAMKIIKSKLRNKMEHGFLANSMSVYIERDISECISSESIIDDFKSLRKRKVRL

>Mt_SHATAG

MASSEAPTQPSAEASTQESQTQRNVRAKTDIAWGHAKIVLDGDKEKPQCIYCNKVMKGGGINRLKLHLAGETGQVEACSQAPEEVRFKMKQNREEQTQREQPRSVVVASQKGTNNGSFDNYFLPRTTPGSQPTIKSVLQTKEVVEKCDLALAKWFIAASIPFNAANSPYFQSAVDALCCMGAGYKAPSIHDLRGPLLNKWVDETKKKIEKYREIWKNTGCTLMADGWTDGVRRTLINFLVYCPKGTVFIKSVDASGASKTGEMLFKLFKEVVLYIGSENVVQIVTDNAANYVAAGRLLMKKSSLACIGLLVQLIALT

>Mt_METRAHAT

MFSVMESISQNLSSTLTSGSANVAEPTEQVAVATQVPVVGLPPLPCLAKRRKPNAGGPRRTSPAWDHFIKLPDEPEPTAACIHCHKRYLCDPKTHGTSNLLAHSKVCFKNPQNDPTQASLMFSNGEGGTLVAASQRFNPAACRKAIALFVLLDEHAFRVVEGEGFKLLCRQLQPLLTIPSRRTVARDCFQLFLDENLRLKTYFKSDCVRVALTTDCWTSGQNFSYMTLTAHFINNDWKYEKRILSFCTVPNHKGDTIGRKVEEILKEWGIRNVSTITVDNASSNDVAVAYLKKRINNMGGLMGDGSFFHLRCCAHILNLVVGDGLKQNELSISSIRNAVRFVRSSPQRSAKFKECIEFARITCKKMLCLDVQTRWNTAYLMLDGAEKFQPAFEKLEGEDSGYLEFFGEAGPPSIHDWENVRCFVRFLKIFYDATKEFSSSQEVSLHKAFHQLASVHCELKRSAMNLNTVLASMGSDMKQKYNKYWGKIENINKLIYFGVILDPRYKFSYVEWCFNDMYGDQPTFFTDLIAVIHTQLFKLFNWYKDAYDQQHNSGHPSASPSESSYVSENVIPAEVPSHLARAEAFKEHLKLKESIVKKNELERYLDEERAEDVNFEILLWWKQNSCRYPVLSSMVRDVLATPVSTVASESAFSTGGRVLDTYRSSLNPQMAEALICAQNWLKPTLNQFKDLNINEEFELSATVVSGI

>Ta_HAT-1

MYKKKLPFNAINDDDFKQFCEALGRYGPDWKQPSQYMIREKMLVQEVERTRDLLKPHEVERADTGCSVMTDAWTDKKKRSIMNLCVHCKLGTVYLGSKEVSADAHTSLYIFNYVDECIEKIGAKNVVQVVMDNASNNMGEKVMLKDKRPKLFWTSCATHTINLMVEAVAKLKHFGSTITKAKEMTTFLYAHHTTLVLMRSYTKKRDIVRPGVTRFASAFLTLQSLDAKRKQLKEMCCSDTWEGCKHTRTKKGQVFEPLVKILRLADGDGQSMASMYGEIIEAKKAILVAVDNSDKDYKAITTAMESKMNGRLDTPLHIAAYALNPYYSYATTSMFTNVEVMSDLMEVIEQFYLDDDEKQNKVLNIDLPKFKKKERMFGKVVATKAISNANFNAGEWWATYGLQTPTLMHIALRILNLTTSSSGCERNWCVFEQVDAKRRNKLDVHHRDDLVYIQFNGRMIDKRKEYSSSCDVLLGEDAFMAQDWICEGAYVDAMEEVDAMGASEFVELHRSSRVRELHEVEEFVSDGEESDHGLVNEDDIEFKSDDDGVIQGANEDEEGDPMEP

>Pt_hAT-1

MSLDKYFKRKSLEDEESIKASSHVTQSSSKKSHIEINPDTLLADPGLRRPIYEYHINDRDAIRRAYLQKGPCQPSHCDFPQKQFGNISTLRRFNPAWFGAYPTWLEYSIAKDAAFCLYCYLFKSKGGVDSFVGDGFSNWKKKERFDLHIGKSNSSHNAARIKCENLMNEKQSIMTLLSEQTVKSQSDYRTRLNASIECARFLLHQGLPFRGHDECECSSNQGNYLELLHFLSRNNEAIKRVTFSEAPRHNKLTSPDIQKDITQAAAEEITNVIIKDLGDSLFSILIDESRDISIKEQMAVVLRYVDNNGHIIERFLGIQHVRDTTASSLKAAIEALFSKHGLSISRLRGQGYDGASNMRGEFNGLKALILNSNPSAYYVHCFAHRLQLTLVAVTKKHNEVGDVFNFISSIINIVGASCKRMEVIREKQYARIIEGLENGEISSGRGLNQETSLRRYGDTRWGSHYVTIIRLLAMFSSVLDVLEIIREDGMNSEQRTEAVVLTDIMESFNFVFMLHCLRRILAVTNEFSQALQRKDQDIENAMSLLKTSKERFKMMRENDWESLLEEVSSFCIKHDIDILNMDDEYKLRGRSRRKSQGITNLHHFRYELFNNIIDMQLTELDDRFTETSTELLLCVACLNPSDSFSAFNKEKLXRLALFYPSEFSIVDLMVLGDQLDTYIIDLRGDDEFSGIEGIASLAEKMVKTKKNLIFPLVYMLIKLSLLLPVATATVERVFSAMHIVKSRLRNRMGDKWMNDSLVVYIEKDIFDKIDNEAIMKRFQNMKTRREQL

>Pt_hAT-2

RSEQPPFKSQRVEIDVNTLERDPGLRIPVWKHPINQQDEIRRAYIKMGPYQPKLAEYPRTESGRQYRRFQYTWFDQFPWLEYSPSKDAVFCFPCFIFENKVPRHLTFTTEGFRSWKRVNDGVRCALLMHVGSPTSPHNNAVKSAEDLMKVSRHIDKVLNAQTVEEVQKNRLRLMTTIESVRWLSLQACAFRGHDESSASNNRGNFLEMIRLMGRLNVDIDDVVLEKAPKNAKYTSPTIQKEILHILANKVRKKICEEVRDAKFCILVDEAKDASNKEQMAIVLRFVDIQGFVRERFFGIVHVSDTTSSTLKKEICDVLARYNLHIFNMRGQGYDGASNMRGAWNGLQALFLRDCPYAYYVHCFAHRLQLALVAAAGNEISIWLFFSKLTTIINLICASPKRHTELHYAQAIEIAHMVATGERETGRGANQIGNLHRSGTTRWSSHFDSICSLIDMYGATITVLESMVQEGSSNSIRGEAGGCLIVMKSFEFIFILYLMHKIMGITDLLCRALQQKSLDILNAMDLVSTTKALLQTLRDAGFDLLLANVQSVCTKYEIDIPHMNASYKKATGRSCQQQGSVTVYQHYHYDIFNSTIDFQLEELNSRFSDGTVELLVLSSALEPKDNFKSFKVDAIYKLAEKFYPEDFNEQEMYYLRSQLEHYQIDVIHHESFQNMSTISELCRGLAETNKSQHYHLIDRLIRLVLTLPVSTATTERAFSAMKHVKTVLRNKMKEEFLADSMMIYIERELVEDIDSDSIIDEFYSTKHRRVQ

>Pt_hAT-3

MENLQNQNASSTGTTPTSTNAPTSNTNPTSTTAGSTTDNKGKQPQVLTSRKRNVDDKKKSQIWDHFTKLDGDPKTPRAECNYCGKDYACHTIVNGTSNMWSHLKVCKKFPFVVDKKQKVLVLEPKKEEGESGDRNVGTLKAIGYNYDECRQALAKMVIIDELPFNFVEGKGFRLFSRTMQPRFDIPSRFTVMRDCLKLYVEEKERLRTALRGQRLCLTTDTWTSIQNINYMSLTAHWIDNEWNLHKRILNFCQVSNHMGETIGQVIENCLLEWGIDKLLTVTVDNASSNNVTISYLKNVMKDWPTNILSNEHLHVRCCAHIVNLIVCDGLKEINVSVVKIRNAIRFVRSSPSRQLAFKKCAEKLHIECKKSLCLDVATRWNSTYLMLEAAEKFEKVFVRLGESEPRYMSYFLEVDSKGNKKNIGPPSLEDWENARTLVKFLKIFYMVTLRFSGSLHVTSNSFFNELIYMHTNLLQLCKSRDNLLSGMAMNMMLKFEKYWGCEANQNFLLYVANVLDPRLKLKYVKFCFGELYDYDKAQLLTKKVKDNLVSLYEFYLKADEVVDDNRHKQDVNDAIDDVEVDVNTLARFKRHLQEEDSVENRNEVERYLVDGCEDPNDDKLDILGWWKSNASKYKILSKVAQHVLAIPISTVASESAFSTGGRILDQFRSSLSPATVQALICCQNWLHHGPIPTDIRTLMNDFETYENLESGNFSYKLTPFI

>Pt_hAT-4

MMELLASYNEQVGALVLGNAPQNAKYTSHQIQKEILHVFARNVQSSIRHEIGDARFCLIVDEARDESRREQMALVIRFVDRSGFIRERFLDIVHVKDTTASTLKEEISFVLSHHNLDVQNIRGQGYDGASNMRGEWNGLQALFINDCPYAYYVHCLAHQLQLALIAAAREISDVHTFFQNLIFIINIVSASCKRNDELRAFQAATIEHLVDIGEIETGKGVNQVGGLQRPGDSRWSSHFKSICSLIKMYGATCLVLENIALDGSTYSQRGDAAFSFKLLMSFDFAFILHIMKNVMGITDVLCQALQQKSQDILNAMHLVTTTKTLIQKLRDDGWETLLEEVTSFCKHQDIEVPDMDACFSSVGRSRRKKKSVTVEHHYRVDIFTAIIDQQLQELNNRFNEQAIELLKLSTTLDPRNSYKLFNVEDICLLVDKFYPEDFSDQEKIHLRLQLQHYELDVPNHPKLKNMSSIADLCQGLVETEKSTIYPLVDRLIRLILTLPVSTATTERAFSAMKIVKTRLRNRMEDDFLANYLIVYIEKEIAERFTIDMIIDDFYSMKERRAQLK

>Pt_hAT-5

MSSHDGSTPSSDPSTAQSSQPSISMSSGSRGRTDLAWGHCREAPELSVGCKKTKLVCLYCAKVFAGGGINRFKQHLAGAKGEVEQCRKCPPDVRHQMLLNLKGNAETKKRVREMQADFNPFNAQQREHEEMMIRQLEDDDDGDDEEDDEDVNTKKHMLPPKVAKKKKIQSTSTVKQSTTSYGKQKKSATLGTYFMPRTTPGAQKSLQNCWQRKEAVERCDLALAKWMIDACVPFNAVNSVYYQHAIDAVTAMGPGYKGPNLHAIRGYYLAKAVDEVKIYVETYREIWKKTGCTLMADGWTDQKRRTLINFLVYCPKGTVFLKTVDVSDVSKTARLLYQLFREVVLYVGVENIVHMVTDNAANYVAAGKLLMEEFPSIFWSPCAAHCINLILQDIGKLQSVCCVVEHASGITKYIYNHCYPLYLMRKFTGGKEILRPAPTRFATNFIALQSILAHKDELRAMVTSREWVSSAYAKDSKGKKFVESVLDSLFWEECAIIVRMSEPLVRVLRMVDGDDRPSMGYLYDAIHHAKEEMMRRFQKRKARVKPFIDIINNRWDGQFYRNLYAAAFWLNPRFQYDANIMDKHMSTISGLLDVLEKYAHGNLPLQSKITSEMKLFRNAEHDFGRASAINNRTLMPPGI

>Pt_hAT-5

BMSSQHGSTPSSDPSTAQSSEPSISISTSSGIRGKTDLAWGHCREAPELSVGCKKTKLVCLYCAKVFAGGGINRFKQHLAGAKGEVEQCRKCPPDVRHQMLLNLQGNVEKKRRAREMKADFNPYSAKQREHEERMIRQLEDDGKGDDDDDDEADGKKQMLPPKVANKGKSKITSAVKQSTASCGKQKENATLGAYFIPRTTHGAQKSLQSCWKNKEAIERCDLAIAKWMIDACVPFNAANSVYYQHAIDGVTAMGPGYKGPNFHALRGYYLAKAVDEVKIFVESYRETWKKTGCTLMADGWTDQKRRTLINFLVYCPKGTIFLKTVDASEASKTAVLLHKLFREVVLFVGPENIVHMVTDNASNYVVASKLLVEEFPSIFWSPCAAHCINLILQDVGKLQSVCSVVDHASSITKYIYNHCYPLYLMRKFTGGKEILRPAPTRFATNFIALQSILVHKDNLRAMVTSREWVSSAYAKDSKGKKFVDSVLNSMFWEECASIVRMTEPLVRVLRIVDSDDRPAMRYLYEAIHSAKEEMLRRFQKKRAKVQPFIDIINNRWDGQLYRKLYAAGFWLNPRFQYDVNLMDRYTNTISGLLDVVEKYANGNAILLSKLTSEMKLFRNAEHDFGRVSARNDRTLLPPGILLFSYSKMENQGSKRGKTMFSFFKPNEQTSTSKGHSPSNVDVSN

>Pt_hAT-6

MASRKNASGNRLDVGWQHGIDVDKNSRKVLCKYCQKIISGGIFRFKQHLACTRKDVEPCQQVPENVKQMILGVLVKNLEATEKKRKALQYSGNDDDDDEIKEISSKDKGKRVASGSGSTQTTLNQLLKKDIREEACRQIARFFYTSAIPFNCVKNPEFIKALEMVAKHGPGFKPPSYYDIREKYLKQEVDQTMKLLEEYKLEWKKTGCSIMSDGWTDKKRRCICNFLVNSPKGTVFLSSVDTSNMSKTADKVFEMLDAIVERIGEENVVQVVTDNAANYKAAGQLLMEKRKSLFWTPCAAHCIDLILEDFEKKLEVHQVTIAKGRRITSYIYSRTILISMLRHFTKGRDLIRPAATRFATAYLTLGCLNDHKMQLMTMFTSNQWSSCRFARIEEGKRIQNCVLDSRFWHDVTICIKAAFPLIKVLRLVDSDEKPAMGFIYKAMDEAKEKIQVNFGSVKKRYISC

>Pt_hAT-7

GVFKKTDPKKPRKPMENPRKPNRDGKPIKPIFKTIKSCRFGSVSVPPKPVNRTGSINNAVNQAPLTLSEAAAHTKERRRFIAFSLLHPPCPPXLLTLPLQFSSSLRLAPCICLFSSXISQQPTSPHPSSILAAEKKPEQNPLASLFTCSRKRGLLHRKGGKVKFILVCLVLLFVSNFDASLTSASHFSILLQLGFLHLSXDLCSFRFAPLSPALSPIFLLICVDIELLIALCHCLYVSVYLDCFDLYYRFFYAVVLSIICLYVPVVLSVLLCLAINLLFCCINSGHCLQVFEFVLXSVYLHICCXLAVTFILFNVHVRSVTVLSKWIWTVLIALXEXGCASIHAGDNYSMKIISLQCIGLGVRCVGLTCVNXHVLSVFCNIALSFIFRIEIGPLRTFCNNWSITDFQVHXMCNLQYAGFLERGDRCLFAIITHDAHCSTSAFSIVVKCFMLLFFFQMDNREDPTPNESNPSSSEPNSSPIPVPVTTSSTNSNTEEGNPASRCNKRKTSQIWDHFKKLDGNPKAPRAACMYCGKDYACHTILNGTSNMWSHLGVCKKFPFVIDRKQKTLVLEPKPIIEGGDNGEENLVTIKAVGYNYEECRKALGKMIILDELPFNFVENQGFKSFCQVMQPRFDVPSRLTIWRDCLKIYVXEKEKLKKALKDQRLCLTTDTWTSIQNINYMCLTAHWIDEGWNLNKRILNFCQVSNHKGETIGQAIESCLLEWGIDNILTVTVDNASSNNLTIKYLKRVTSGWATNILSNDFMHVRCCAHIVNLIVCAGLKDIDDSVVKIRNAVRFVRSSPSRQLVFNQCAERLKIGSKKSVCLDVATRWNSTYMMLDAAXKFDVVFMRLEETDPRYLSYFEVDSKGKQKNLGPPALEDWEKARSFVKFLKLFYTVTLKFSGSLYVTSNSFFHELISMHTSISQLCRSEDVYVSKMAKNMMAKYKKYWGDQDTQNFLLYVAVVLDPRFKLKYVRFCFGRLYDVEEAENFTIKVKDTLLRLFEHYMNVDENVEVVHSVGTSINEDVNVDLMVVNDDMLDDLASQFKKHLEEEGGVQKKNEVERYLGDDCEDPNDFKLDILGWWRRNATKYKILSKVAQHVLAIPVSTVVSEAAFSTGGRILDPFRSSLSPSTVQALVCCQNWLSLAPIPINIRTFMDYIENSEMIESGNFSYKFKCSYFIISFIYICLIHFNLCFCRIWKFKNLNLFVSSIDDVGAILEYNCFGLIYFTLLIPFKILHCFFSNSNMLLICCFFIALSLLDLLAFAVAGSFGCWCWIFFFKLCRMFMIIYCWFANVSVCCLFCEHLFFEVFKFDHASCILHFEFSLISLHIKDSKIEHFKFIVTISISKKIQYFNLLKSLQTSNKILKSFDKAYKLSPTRKFKSPISKPIKKPKKPINXMIRFPGFLXKKPDLTEPNRFGLNRXPVRFGLNFKYELLGSVGFXSPNRTVNRP

>Vc_hAT-1

MRGRKKVKPVSQRGIAECMTGPYIAKHAKGNDLNAEQTPKQQDKPEIFPDKGVWDGGAVVGNASAMVLLPAATDDTGAITPAFHGGCDQHKRKLSPNQLDNNECGEAPNPSARSAKRAQTQRIRKGMSDGAWRDKQKENPHRWQIAWAARYPWAQPVHADVDAGEVHNQVRCIPCTLMKKSDYLIMCRAATLDKHVTSEVHKTSVEHQQSQKARLALQQQRNATLPQLYTPGNTEAEKAKHQQLIQLFWQLTRGRPITDFMASKEAFEALSVPHISSSHWSGFSAWNLAEALDGVLMDKVRELVRSARFISLSMDECVGIDKNSRLSLHVYVMDAQWSRLPLLIDVSFNGWTGCRDWGFAAFVLFSTSCNIIPHKFLSSELVSTLSIPASPQLAHVRGAPDAENITQLVLDTLNVEGDLGEQDLARKLVAVSTDGASVMTGVHNGVSARLSQTAPFLLSIHCMAHRTDLAIAVLEKAPVVKMIVSVIKDVYNYFSGSAKRVSMFEDVAKAAGTDGNRLKRIVETRWICAREPAASLLAEYPALLPLFCEEEEKLYNTLRDARLYVSMHALMPALVTIDMFIKLCQSRGLYIGELAKALQRTKDRLREMYLRPETRYTTVEFGDFKELLSVDGDIKGTAWVFNPDTPGEERIGLMCGSSGFYPFTADPPRTGRDRTPPPLTPDRLAQVVKSVQADMSAAVEELIGELSSRFPPHPLLEAASIVYSEYWENRPTAADFKAKLKILKDYYCRERVGANSSKVPPMLDSALLDEQANLFASSMNQLAAQQRLRRAREQTERQRQQAAAVDPDAPTSKREHRVLLDDTPTARVWQAISNCAVLAGGLTEYSALAQLVLVMVPGSVEDERRFSAMGYLQDPTRNRLDEHLALCVRMFTQDLFDLGTFPFRDALRKWLDSSSRGRYMMNR

>Vc_hAT-2

MAQPPSAPAAPEVVDLSGKLPKAPVSDMGQAAFVEKYPWWQQTGKNKQGQAIGGCSICQQHKGSGGGPKLSPIVKDSNDLKKHDESVGHVEAVCIAKAATGQGPIDKATRVAAQQVAAGLASVLQLLILCVLWMVKEHIPALKLSSLLLFLAVARVDVVRHKYSHHRYFMKALRALSQVVLSMQIAEILQSPYFSILCDSGTDISGEDHLLFYVRYLNTDTFMSVTQYLCAVRIIDKGAENIHLVTEAVLAALRLDQTKMVGYGSDGASVYLGRNSGVAVRLRQKLCYLVAIHCCAHRTALVMGDVKEELQQMSAMDALLKSVHNLFRSSTMREKWEKFAAERGCTRLKFPVYNSTRWFSRMQCVVTLGLNAHVLLLFLNKYKKNWPQGRAVHEQLSNVYMLATLFALRDLLAPCERLSKAFQADSLKAHEIHDTLSDTKDSLLKACGFSSGRLKGKHYAGFKSGLQGRSWVVVSNRVKVSGTLDEGKLQSFACRFVGLILKHLDARFGDTELLKCFRIFDPATYVNMSEEERRGYGRKEFVKLLVHFCHSANPNRLFAFDGNELDGLHKEFSVMKSKMALQLELGRTSWERAWAGMRQWVHFFPRLNQLVYVACVLPTHTACVERGFSVHRIIKNRLRNRLSVPSIDSLMRVGLLGPTVDMKCKPLVIKAAEEYVAKKPGWAPAPYCQGGRLCSA

>Vc_hAT-3

VFFFGNILLFWEMGVAWKKWNNWSVGRTGSLLRPVAPNAFCTYKAEHLELIHYLCIKYLMISPDGPLLHSTVNGAFVHSPKQTAQKVRRRNAPNIKKNIIQKTRIGMPLPLLKSVKPAIAKLRIPRRSGNTQLQQSATQMLSSLILTRKGHRPKMSPRHHRHLQGRVLRSTRRWFKSRRGASKHSVCRKAKAMGAGGRTRECVGAVPAPSAGGGTSRWMPGRPRKATSSPKNTKKPCAATKRGPFSTSGTSQRGRSTTFCPEHSASPRGELCSSCGSSSSCWITGAPSSTMSALRTCSRCWTCHTTVISTGAATVPGSLPRPWMRCSRTKSAGWSPPTALYPAWTSPGALTRGPGSAFTSMSWRAGAGSRCALMGRDDGKVNRNAGVNCWEPSSFNRYSFVHISSVTASSFNRFFWPWIDPIKVSVCLSFRVLQLAKLEAAPTADNITSLLLEVLADRAGLTANELAQKLVAVSTDGASVMTGVHNGVSTQMTDLAPHIVTIHCMAHRTQLAAGAQEQAAVISTLKALVRVIYSHFSNSPKRLLAFEAAAEEADTAGNHPLRDVEIRYAKPRIYGGTGLQGPLRTCQTPFALYYHHLTVCFCRPRRWLSLLLPLQRVVSEYGALLLYMEEAEEQFIFTQMTDARLYLAMHALLPALRRTNFFIKLCQSRHLFIGHLNIELERLKADLRRMYLDSATCYQGADFVDYTQLLPTGPAGDSLDGTQWELSPEDESVGLVFGDRFIAFHAMQQRRGRGGCMVSLTAELITSTAAAVQAELTAAVQVLADELEARFPPTALLDAFQILYSEYWEARPTAADFRSKLKVIKTHYGKECELDDGSKVPPKLNIAALDAKATEFEQLMRALATKQRLARIRGQTTKLREQLEAERAAAGSKVVQKLREEQEESPTIQLWKQLENAPVHAESLKEYVALAKLVFVMVPGSVEDERRFSAMNFVQSKVRNRLDEHLALCVRMFTQELYDLRTFPFDVAVKKWREAVIKGRYRMNDLLMWCRGLASNWSAAGAMQWCWCRQCTGYIFVHTMDSYLMEDYTYIYITLPSRAHDVYGQSKWLRALLTETLAQMQLGAQNDGGTACVFPDVRCCLQHTVRPLRGGSGGGAAPPSDYTNRNCNRKKHPVYYSRVLGPKRKKNT

>Vc_hAT-4

GLARVLYKRVLYYNSTIYSTIYYAGLTGTSLRRSYRVRTNSIVKIVLYSTRFGVGCPVLVGPSRDLQSSCILKGYSRVFELCYVVIDEYQRNPVRMRRRSEACFLVQTSRLKFSVLGTSFLTSFVTRRRTPRRVVIGTTSMWSGGVVYQSSACSAMSRSAQTHPLPTRTTSSKVLARLSNSVQRHSASNSNRVEPALLRRAPARRISRPSPSAGAALMARPSSQFPRLSRGKSWRSLACSSTGTTCLFTSRSPSCGTYSGCWASPCRTGSSWGQCWTAPTARLRRRWRRSGSACVAKLHSAQMAGASVPLSRGCRSSTSCCCCQRARPSCVSCVQLVYGRTRSTSKRYVMCGYVSLVPSAVIVIYNRTANPALQIHLDVVAEAFTKPDDCIGFVMDNTATNLAAIQLLRIENPRWLGVGCGVHGMALVFKDLAKEKNVKWAAKIFNASITISTVVGDSERIRALLGVHQMEKYGKKSAITANTPTRFAVNYFVLSDVLANEDALKAMVRNKEAWAGASDGTSKAQEFKAMVEGEGSSRNLGLFSKGAKLAEFCEPISKALHKLECDRPMLSQVQRFKGLGERMCVPCLPQTPSGITFHSVTRPPYASHVFSHPSQMYPVWKALLATARDFDEANTDCPGLYDLLKRRYDKQVNACWFAAFLLDPRNIDVSLTNRYMLPLGQLELEEKSAAKVLIQDLAAGKDAAKRDRVGEEWTRLGLGIPKAFDEWSELMRMHKEKLLPDSSTRVMGASLDDCISYWDVHLAAQFPLIASAAIRLLLMHASTCSSERNWSAWGLVYTKARNRLAIEWAEKIVYIRGNAGLQGNQGAAADEVELILQQCEEDEQEQEAQQQQQQQQGNVHPNTQIDDATIDAAAVCIGGLHTGPLCRNFTSMASLCCLVNAGSSICDYVFMCHNTPDMGFEVHLPGTEVTKSVLDDHSDLLSHVVNIQKEQKKTFPTVFSGTTVGRTPPFYRELVPAYHSTILLYIVLFHTILYYLGKSIYFCQP

>Zm_AC

MTPPVGNNPPSGSAIRLAKLMSTTRAPSTRKTNSVFSAYAQGLKRKAEASSSRIQNVRARARGHGCGRTSPSSSTAEAERHFIQSVSSSNANGTATDPSQDDMAIVHEPQPQPQPQPEPQPQPQPEPEEEAPQKRAKKCTSDVWQHFTKKEIEVEVDGKKYVQVWGHCNFPNCKAKYRAEGHHGTSGFRNHLRTSHSLVKGQLCLKSEKDHGKDINLIEPYKYDEVVSLKKLHLAIIMHEYPFNIVEHEYFVEFVKSLRPHFPIKSRVTARKYIMDLYLEEKEKLYGKLKDVQSRFSTTMDMWTSCQNKSYMCVTIHWIDDDWCLQKRIVGFFHVEGRHTGQRLSQTFTAIMVKWNIEKKLFALSLDNASANEVAVHDIIEDLQDTDSNLVCDGAFFHVRCACHILNLVAKDGLAVIAGTIEKIKAIVLAVKSSPLQWEELMKCASECDLDKSKGISYDVSTRWNSTYLMLRDALYYKPALIRLKTSDPRRYDAICPKAEEWKMALTLFKCLKKFFDLTELLSGTQYSTANLFYKGFCEIKDLIDQWCVHEKFVIRRMAVAMSEKFEKYWKVSNIALAVACFLDPRYKKILIEFYMKKFHGDSYKVHVDDFVRVIRKLYQFYSSCSPSAPKTKTTTNDSMDDTLMENEDDEFQNYLHELKDYDQVESNELDKYMSEPLLKHSGQFDILSWWRGRVAEYPILTQIARDVLAIQVSTVASESAFSAGGRVVDPYRNRLGSEIVEALICTKDWVAASRKGATYFPTMIGDLEVLDSVIAAATNHENHMDEDEDAIEFSKNNEDVASGSSP

>Zm_ZhAT2

MKRKKGPIVDLKDFLQRAANKKKTQEQTQGNTDPMSTTKESQMQLVVYEGRPKYASGSGATSVPLEPESEIGDFAISDSESKSSDEGNDESTYNIEHDPGLRSPISGYHINDHDSVRRAYIALGPCRPKMKKDDFPQHNCGGMRRFQPKWFGEFNWLEYSVHKDAAYCFVCYLFKDSDHGGDGFISGGFRNWNMKDRFRKHVGAINSAHCEAEEKYNLFIKPKTSIRESVASNTVEFKAQYLARLTWSLKCIRFLLHQGLAFRGHDEGKDSHNKGNFRELLEWLAGNFEEVNKVVLGNAPKNCQLIDHKIQKQLITCCADETTKFIIEELGDECFAILADESSDAYQQEQLAICLRYVNRMGEPVERFLGLVKVEDTTSLTLKEAIQSMLIKYQLPLSKVRGQGYDGASNMKGHVNGLKKLIMEESPSAYYVHCFAHQLQLTLVAVGKDNIDCQWFFGQLAYLLNVLGMSCKKISMLRVAQAEYMIEALKLGEIETGQGLNQEMGLARPGDTRWGSHYRTVVHVMALYPSIKKVLSKIGKEHKGGESLGARTMLQVFQSFEFVFMMHLMNEIFGYTNDLCIALQRREQDIVNAMDLLEFTKVELSVLREDDGWNEFNGKVNSFCEKHKVKVVDMDGKYKPIQRDKKFYKNAINYHRFHADMFLGVIDRQLMELNSRFDEVNTELLRCMACFNPINSFAAFNQEKLVRLAGFYPHDFEFEEMNQLPFQLNRYIENMKRDDNFKNLRSLAELSMMFVKTQMTLRYDIVYKLLKLVLVLPVATAGVERVFSSMNHIKSKLRNKMGQEFLNGCLATFIEREFFLQAKDKDIIARFQNMKDRKLVL

>Zm_ZhAT3

GTGTAATPSTATTTASTTSTDTICHKRGRKSTSDVWNDFEQLFKDINGKKVRYAAKCLYCKSQLTASSTAGTGHLKRHRSTCASKAQRAAKTQSLIQFNADGSVRSWDYNPDIARTELCRLICTLDLPLGIGSTAAFERYIKHAHNPRFTVVCRQTTTRDFVKFYNQSRTSLIDVLKSSVSCVALTSDIWSGNAKEDYISVVAHYVNADWVLEKRVIGMRLIDVSHNGANIADRVKTVIAHFGLTDKVFSITLDNASANTNAMNILSPQFSGYVGSLFLHQRCACHIINLIVKSRLKRLKPYVEDFRTAVSFLNSSNQRIASYKSYCIAMGVRPRKFGLDMDVRWNSTYLMLKHLIPYKSTFSVFIQTHYQQHMTQPLLTETHWEVGEKILTFLELFYDCTVALSGVYYPTSPMMLHHIIEIAEHLNNFENDIMLRDTVVPMKSKFLKYWKSIPILYSFAFVLDPRAKIRGFSNALQVLSRYVTTDYSEYFNVIRSELTNMFSKYEIKFGAAKLRRPTHTTSTSRKKTAWGKIYASPEVSEVSDAVSSSSFGSQVSELAMYLDSDPITVFDDGFDILSWWHEHKSNYPILSLLAKDVLTVPVSTISSESVFSLAGRLIEERRRSLTSEMVEILTCLKDWQLGESREQHNIANNDMEEHYKNMYLDEEREAGVGRS

>Zm_ZhAT5

MPSAPPPPTLYDVSRLPHDPGERQPIASYHANDHDAIRRAYILRGPFQPYAHEFPNRKIGDRDRHFNFVWFQNFPWIEYSVKKDAAFCFMCYLFKSKANKGKGTSAFTSDGWNNWNKGSEALLKHVGSMSHKTAEEKYLGFINPNAAIDNKIEKWSDDDRNLYKIRLTYSLRCLKFLLHQGLSFRGHDESEESSNRGNFIEILKFLAVNSEEVNKYVLNNAPGNCTLTSPKIQKQIIQCCAIETRKKIIEELGEEPFAILADESSDISHKEQLALCLRYVDALGRPCEHFIGVVHVDDTSSLSLKDAIEALLVSHGLTLTRIRGQGYDGASNMRGDIKGLKTLIMQESPSAYYIHCFAHQLQLVLVVVAKGNNDCVWFFDQVTLLLNIVGVSCKRHGMLRDARLENLMKSLECGELETGRGLNQEMGLPRPGETRWGSYYKTICNIITMYPVIRDVLMILGEDTTIRSDWTRIHSMLGAFESFDFVFDAHLMFVILGYTNDLSVCLQRREQDIINAISLVNVAKRRMQQLRLDGWDQFLQRITSFCNKHDIQVPAMDGNYKPYGRSSRFVHNQTNDDHFRREVYIGVIDQISLELDSRFSEANMELLSCMSALDPSNSFASFDAHKVRKLADFYPNDMSSTDLLKLDLQLDNYIDGIREDDRFKDITNLVDLSVKLVETKRHKVFDMVYLLLKLVLLLPVATASVERAFSAMSLVKTKLRNKMCDSLLDDCLVTYIERDIFFEVNEEDIIETFMALRKRRPDK

>Zm_ZhAT6

MQRRMESARKGKLLLQDQMYGSTSARLNLIMGMKESSASIVQNCFACDPKINGTSSMKAHLKVCKKNPNKMVVDNQGTLQLQPSQGNSSVGTVSTWKFDPDDLRNSFAEMIIEDEQPFVLPERPGLRKFLAKACPRFVLPSRRTATREVVKVYDVQKEKLKNFLXENCERVSLTTDTWTSNTNQNYMCVTAHFIDESMEAPQENHRFFLGKRS

>Zm_ZhAT7

MLCRWLTFQGCPFRGHDESQNSLNQGNFLEMVKLLASYNKEVKGVVLQNAPRNAKYTSSDVQKEILSIVARKVQKLIREEIGTSKFCIMVDEARDESKKEQMAIVIRFVNKEGLIKERFLDLVHVKDTTALTLKNSICAVLTANSLSIEDIRGQGYDGASNMRGEWNGLKALILNECPYAYYIHCMAHQLQLALVAASREVQQVHNFFQHATFIINVVSSSTKRNDELLAAQAEEIAREIELGELDTGQGANQMSSLQRPGDTRWSSHYKSIQSLKKMFSATISVLRSIANDRSVSKYSRGDAAGALQIIVKFDFVFILLMMEKIMKITDVLCQTLQKKSIDILNALDSVSNTKVLLGNLRNDGWDPLLQEVNYFCEKNDIDILDLNHKYVSFG

>Zm_ZhAT8

MADDDDVNPVTGNDDLRAAGLVPDDEDDIQADAAALLGIDLTSAPVDLSSNPTNAGGGPATATDTPVGSTSTDGGTGTSSVGKRKSTVWVDFDEVFEKVNGSNVRIAAICRMCKTRLSARSSAGTGHLLRHQKACRKKVDHAARVQSRLAFNPDGSLHNWEYDPIFARTELCRLIARLDLPLGIGETQAWEDYIARAHNPRFTKVSRQTTTRDLLKLFTERRNVLINSVLPATSSVALTSDIWSGNAKEDYISVVCHYVNADWELQKRVIGLRLIEVRHTGQNIAERIGVVVEEFCLVDKIFSVTLDNASSNSKAMDTLTPLFAGYLGPDPSPESDPNRRNYTLMHQRCACHIINLIVKSGMKRLKPFLEDFRTAINFLNASNSRIATFKEYCKARGVRPRKFGLDMDVRWNSTYLMLKHLVPYKTVFSVFINSHYESQLLPPNHWYVAEKILEFLELYYDSTVVLSGVYYPTSPLVLHHILEIASHLKACERDLNLRTIVFPMQLKFLKYWSDIPLLYSYAFILDPRAKMRGFFNVLHLLGDCTGCEYSTYYADVKNELYKMFTKYETKFGAVRAQRAAQPSIHTGKRKQAWGRIFGDSSSGVVGPPPISTPSASSSSAICELSAYLDSDNVTSYEDDFDILLWWRDHKLTYPVLSIMARDIMSVPVSTVSSESCFSLTGRIIEERRRRLLPENVEMLTCLKDWELGEKREQHAVDNPELEDSFQNLFLDEDEPVAA

>Zm_ZhAT9

MLSKHSLSIKNLRGQGYDGASNMRGEFHGLQRRILDENPYAFYIHCFAHQLQLVVVSVAKCCSSVMDFFNYINLIVNTVNASCKRHDQXAQXHHDNLVQRLENGEMFXGRGKNQATNLARPGDTRWGSHHKTLCRLQLMWSAVLEVLENVCQDATNLAQRTTATGLLEKMESFEFVLVLHLMIKVLGKTNDLSQCLQKKDQNIIRAIGLVGVTLQKLNEIRHHGWDAIFEEAKDFCLKHNIVVPDMSAEIVRRGCSRGRRGQLVTYYEHLHHEIFNVVLDQVIVEFNNRFAERSTQLLRCVACLDPSNSFANYDEDKLVELAQIYVDDFTEYDCIVLRDQLDTFITEGREDSSFLSCCDLGNLAMKMVKTDRHTVFPLVYRLIELALILPVATATVERAFSAMKIIKTDLRNKMGDEWMSHCMICYIERDIFASIEDRQIIEHYQAMRTRREQIPKTSKSGTFSSNNSILCSLFSLITYVIT

>Zm_ZhAT10

MDLDPAAEXEEAERQLEDHNEAMDARQALLGVGVTHLDPIXLEDFVASRTGTATDSTSPPASTTASAGGGSRKRSKVWNDFDELTNLVNGKXVRYAARCKHCKQTXSARSTSGTGHLLRHNCXAKKAHERSGQVQSVLKYNPDGSLQRWEYSPSVARTELCRLIARDDLPLWHGSTDAFQXYITRAHNPRFVHVSRQTTARDMIKFYNERMVNLIGTFKTDVSSVCLTSDIWSGKAKEDYLSVVAHFVNSKWELEKRLLGLRLIDGKHSGVNIANLVATVIDEYGLTDKVFAITLDNASSNNTAMKFLRPFLSGYLGVPAPVVTDDDDDSFTPTDDELSTMFLHQRCSCHVINLIVKAGLEPMKTYLDDFRTAITFLNASNQRIAAYKSYCMSMAIRPRKFGVDMDVRWNSTYLMLKHLVPHKSTFSVFIKTQYPLNSDGTPLLTDNHWAIAEKVLSFLELFYESTVALSGVYYPTATLMLHHILRIARHLNAFENDRLLRGAVVPMKDKFLKYWRDIPILYAVAFILDPRAKMRGFNKLLVRLSSLTGTDYSSLPIDVSTKLTKIFQLYETKFGDTRLRANQHPSSLGSGKKKMAWDDIYGDDDXDGEFSAPLGRRPGSTSISTXASTSELASYLDSDTITEFDEDFNVLSWWHQHKLTYPILSLLAKDVLTVPASTISSESTFSLAGRVIEERRRRLAPDMVEVLSCIKDWELADAHLQHTVEEDTQELEAEHENLYLDDPEEHNQ

>Zm_ZhAT12

MLADLLEKKVDEIGRDKVVQVVTDNGANYKAAGKLLMDRIPTLFWSPCAAHCLDLMLEDIGNLKEFKKPIARARRVTTFIYRHGRILAAMREKTGGADLVRPAATRFATSFLTLKSLYKHKDALKALFASTAWTDNRLSKTSAGLDVYNIVFSTQFWNSVEDCLRASGPLLIVLRVVDGDERPAMPEVQALMKCAKEKINQSFAVQSKKTLLKKIITIIERRWEKQMDHPLYGAAMYLNPGKLHPLIRNDDDATVGQLRGCFLDVLARMVDDEETRDKINSQAMDYEFLRGAAFSNKMAKDNLQTMTPRKCLWLLLVYYLLVVASSLLNCCDVIAVEWWRSYGGRAIELQRFARRLVSLCASSSGCERNWSTFEFVSYYVCLLKSSSTYLATFVSNISGLNDQVFSLFLFCRSIQRKETDCCIRG

>Zm_ZhAT13

MATNSEANDSYDPLKDQSRRPKSNDPGWKYAYYVEAGKRELIQCALCPKQIKGGIKRVKQHLAGGYGDIVKCPNTTREIAKEMNEALNNGKRARPLYLDDDVMEDEAGQDDDVQVMGSRSMSASSKASSCTVPSSGTAAKRNKAIFQLKPHPIAAPKKSIASMIRRTPQEVVAERHAKGPAQTTISATMKPKEERNSVCLEIAKFFYECGIPFNAANSGI

>Zm_ZhAT14

MSSRRYLSGSEKRKRKKRVDDLIDSQRGAIDKFFKSNASASTNPNDXFALAIVPVGTEEPTNENSREEEHVDINADDNNVSDHENMSNSSDAHAQFGSVDEQPDIYDPRNWDNLDNKARDILIEKGPIREEGIKFPLDDASRHFSYAHYYRKLSNGELYDRKWLVYSKHVDKVFCFCCKILKSSTSNSLSSLAHNGCRNWRNISTKLREHENSVEHFQNMNKWNELRTRMHKEETIDKDLQKQINKEKERLRQVLLRIIAIVKFLGKXNLAFRGSIEQLYSDSNGNFLACVEMIAEFDLVMQDHLRRIQNKEIHYHYLSPKIQNELIVLLSSNITRSIIKIVKEAKYFSVILDCTPDISHQEQMTLLVRCINLSNGKINIEEYFLGFLNVDDTSGLGLFSVLLDSMKSFGLNINDIRGQGYDNGSNMKGKHQGVQKRLLDINPRALYMPCACHSLNLTLCDMAKSCGKAVSFFGIVQRIYVLFAGSTKRWNVLCKHVPIFTLKSLSNTRWESRISSIXAIRYQAKELRSALFELSHASDVEPKDKSDAKSLFDALGNFEFLLGMVIWHDILYSVNKVSKKLQSPDMCIDSALNQIQXMVQYFEAYRNEGFPSSLIISKGIADEMGLEASFPVKRRALRKKQFDESNDQEEILEAERGFKVKYFLVVVDMAITSLKTRFEELMVFKDIFGFLLRSTILKSLNDTXLEESCTKLADTFSHNGSSDVEVHDLISELKILKFTLPDGILSAMEIFEHVRDLDCYPNVSIAYRILFTMPVTVASAERSFSKLKLLKNYLRSTMTQDRLNGLAILCIEKKMLDDIDLNGIIDDFVSQNVRRN

>Zm_hAT-15

MSTADDSADDELSLEAVVEERRLEAQNEADDARCLLLGTTSAGAIDLDGAGAQTGTGSTSPTADASTSVPVPGKRSRRRGPTSKVWLHFEEVTAMQNGKEVRVSAICLHCKNSMSAKSSSGTGHLIRHLDVCPAKKEKDRTGKTQSLLKYNADGSVNHWEYSPSVARTELCRLIARLDLPLCFGESSAFQEYITXAHNPRFIKSSRQTTARDLIRLFNDRVEQLIEVLKHVSSVALTSDIWSGKAKEDYISVVAHFVNSDWCLEKRLLGLRPIEVAHTGLNIAERVEMVANDYSITDKIFAIVLDNASSNKTAIDVLKPVFSGYIGHLIPAPTRNESDLSNIFLHQRCACHIINLIVKSCLKRLQPYLEDFRTAITFLNSSNQRIASYKQYCMSVGVRPRKFGVDMDVRWNSTFLMLKHLVPYQSTFSVWIRTNXPCKDDGSFLLSDNHWXIAEKLLSFLQLFYDSTVALSGVYYPTSPLMLHHILKIARHLNAYENHELLRNAVVPMKXKFLKYWRXIPILYAFAFILDPRAKMRGFHKVLQRLSTLNGTDYSRYPSCIXTKLTKMYQIYEXKFGGVCLTPQPQSGSGSSKATEAWDDIYGDDETYSNTGTGGTSGTSTGLSELTSYLDSDTETKFGPDFNILIWWQRHNQTYPILSILAKDVMTVPVSTISSESTFSLASRVLEERRRRLTTDMVEVLSCIKDWELADQHKQHTVEKETKDLEATFEXMYLDDEQQVSPGXKRKEAAPQSSRRAGL

>Zm_hAT-16

MKLLRSTATRSDGCVGWPEMTMRMTSGRMLQRSLAXAVSIPSMSVMNNKSVMNNKSVVRITKVKGTNTVQDQEGEGDEHSSKRCRPSTSAVWLDFEKLFKIVNGKKVRFAAKCIHCSKQYSALSSGGTGHLTRHRDRCPRRREKTRMSQSQISFNPDGSMRNWEYCPMVARNELVRLLARLEIPISLGENAAFEHYIRTAHNPKFVPVSRQTTTRDMVKYFTDKKAKLVETLSSSDVNCVCLTSDIWSGNAKEDYLSVVAHYINPDWQLEKRVLALVLIDVSHNGQNIADRVAGVLADYGLTNKVFAFTLDNASSNAAAMRLLRPIMSPYLGIDNGADSEMFLHQRCACHIINLIVKEALDSLKNLIETFRTAISFLNSSNQRIAAYKSYCIATNIRPRKFQLDMEVRWNSTYLMLKHLFPHRIPFTTFIHANYPRAEGDQFILTDEHWVIGDKVLKFLELFYDSTVALSGVYYPTSPLMLHFLVKIAIHLKNYANDTHIRGVIQPMXDKYNKYWRNIPLLYSFAFILDPRAKMKGFSRVLRRLMNLTSTDYAVYQVTTRAKLTDVYNKYEEKYGSVRLNRVVPPNLSGKKRSAWDEIYDDADDVGTSVGMHSFASTLNIARDTSATALLHAASSSASTASELISYLDCDTVNQLTDDFNILHWWHQHKLTYPVLSIMAKDILTVPVSTISSESTFSLTGRIIDERRRRLKSDVVEMLTCIKDWEDAEARMQHMVDDKELEETFENLYLD

>Zm_hAT-17

MASASGSGCASTGASLVAANTLAVAVQETTPVNGAGEGEDGERTTDIGVKESDTKKRKPKRQTSGVWDYFIKSTVTEKGDKGETEEQIWAKCKKCKFKTRGESSRGTTIFWNHLKSRHYIHRGQQELQVQKGEGNNAIVQNFKYDPEASLRKFHLAIVMHEYPFKMVEHEYFVDFIKSLRPHFTFLCRTTTRNEIMTMYMDEKKKLFEQLKSLSCRFSATMDMWTSNQNKGYMSITIHWIDDKWQMQKRIICLPHVKGHHNGELLASEFVKGVMSWNLEKRLFALTLDNAAANDKCVRLVVKELNKLARIQKYPPLVCGGVFFHVRCLCHILNLVAQDGLSVIASPLKNIRAIIVIVKNSTLQWEEFEKCAQFFDLNNKSGLPLDVPTRWNSTYDMLSHAMYYRSAFERLVYLHKDKYWHCTPSSDEWDMAESFCKCLKQLNDATVLFSGCQYPTTNLFWWKFYEIKLALREWCESANITIASMAEAMQKKYDKYWKKSNIALAVACFLDPRYKKKLVEYFMLKIYKDSAVAELTRFMDVVNQLFQSYLSSTRDVSITQTDLAASTSNSICINTDIDEFIYEDNDAAKGEINELDVYMKDKPIRWVDPTGKGLQFDILSWWKANQVTYPVLSRLARDVLAVQVSTVASECAFSSSGRVVSKYRSRLEHETVEALVCTKDWNLASNKGKTDSTYLLFLFCLLLSILSYH

>Zm_hAT-18

MCRGGWRRERAAAVEVIMGGSYIGEESTGGGDAAAILATTASAGGSTGAGGTASSVAPDAASLTEKAIAALPPALAAQAVDSKRKARSQDPGWNYGWWPDPTKKDFVQCIFCQKIVPAGIKRFKQHLAGGFGDTVKCARVPELVSKDMLAYLRMNSRALPLINVDEEEDATEEFAAAEPSSGTKCKQAKKRSAQSTMTSFVVSAATKPITQKPSKSIASMLCKTPEEVVAERHKSKTSQSTLEHCTKKGKEAKQIVDDHVADFLYENKIPLHVVKSRSWEIMLESIGQYGPGYRGPSYHEARVPWLERAVKRTSELRSKHEASWREYGCTLMSDGWTDTXHRHLINFLVNSPAGTYFLASVDASSEVANANMLADLLEKQIDKIGREHVVQVVTDNGANFKAAGRILMDRIPHLFWTPCAAHCLDLLLEDIGKIKDFNSCINMAKKVSRFIYKHGRIHNLMREKLGGDLVRPGVTRFATSFLTLASMLRHRNGLKSXFVSDEWHLTKFPNTEEGRQITRIVLSMPFWEKVENCLRASQPLLIALRIADGDETPAAPEIMAAMDVAKATIKESLKDKPRLQADVLSCFEKRWENQMEQKLYGAALYLNPSKFFALRDKDRRQAARLRSMFNDVMWKMINDDDEQTKISKQADDYERSEGECFSKQGAIRDRERKNPSKFFLYFSHTIFSYLVFANELTKLPF

>Nt_SLIDE

MALPIDHEAGLHDETNNLVPGAGRPLDDGTEARIEVPEIRAEVPLDVNSQVALEANQRSEPEKSIQGSTRSIIMASQNEENLPASSSNAISLDDESQRPLQQDEVELGKRRYSRAWKHFKPVKDNGISYGLCKYCDRRYKNARNCGTKSMLDHIPKCPNRPRDVQNEGDTGGSYFDQDISRKQLAHAIILHEYPLSIVDHVGFRNFVASIQPMFKMVSRNTIKNDIMKFFDNLKSQTSKLLEKVTSRIAITTDMWTSNSNKKGFMAITGHFIDDSWRLQSHILRFAYVPAPHDKDALCGALVNCLFDWNLERKISTITVDNSSTNNAMIKTLLDEKLNKKDLLLTGRVFHVRCAAHILNLIVQEGLKVIGDSISKVRDSVLYWIGSAGRIERFEEAARLVHCSCNKKLEYDCPTRWNSTYLMLRTAIEYKEVFRKLSLTDTNYQSYPAEEQWSNAEDVSDKLILFYRITEQFSGTQYPTSSQYFTKVCEIKLELEAWVKEFNPLISDMASAMLLKFKKYWDDLHILMGVAAIFDPRYKMRLVEFFLPLIYGEEASTKIQEVRSNCYNLFQDYKSKLSGPHDSLASSSSEVTSFIEGDRLSSFDRFVASSGAIVETRSELDMYLEEGLLPRTPSFDNLSWWKTNGLKFPTLQKMARDLLAIPVSTVASESAFSTSGRLISPHRSRLHPTTLEALMCARTWLWNDLNGLSSTIDKVSCPTLLDEEEEPDSSGLSQHC

>Os_CRATA

RDGNGYPVPGPRSPVENSPIRWWYLCLHGESNGEKVFSVGSGGGGFGSPSPVPALCGGPIKYVCSNILCIPIWPIKTRPKASISNCVTLTIPILIPSVPIPSTPPSVGHSDAAPAPDLAPAPARAGVRALRAAAPAVSASAPAPGRHARRVAVRSSARPPRQVPAPRHAVPFPSLPASQVRGRRGLGTGHRTSASSSTLRQYLIRHVGLFVLLMVDAGEGFGGKKGENDESTRRPAARVRHGFAFTTAKLRCKVSTSSCPTPNSVPLPRRLPIVPYPPPPPLNSSSMAASLSRVTNSRSAIHNKGRQCGVGPAIACYKRCSCSCSHTRCRACCWICHCSCGQIWFLQLRCKGLLSAAATTYKKDHHVKKPSCDCRSTFCFDGRSSFCPRTPEASSSANGLRLFKSMGFDFTLTLYTLLHFRNPKFLTIHVQFINLQLAFSILNVVFRVSVENISLHVYCMVAHMLLSYKYVMNLSFNRNPILCSVDHKKFIILAVADFSMIYVRFFISSLPRLFYRLKDILIYIYLSFVRFTTNSTAGTGCGSSSQQESQSLPHKQSLLMILSQYKLKMMSQKMILRILAQRGSLLLLFGRISRKSKSVVMSRLNAFIATSGLEGRAAMGLHIYMTIKFAHERSRWGQRHLHNLHGSIPREGKFLWTHTHLIQQWLGESLLPYYMSILYLLLTILAFEDLLVHFSHYSRWLGTLLGNFSSSIIYLLVLHFNLISVYLMFEYRKDIMDTYEEEKKRALAYMAGAKSRVGITTDLWTSDNQKRGYMAITAHFIDDSWTLRSIIMRYMIHKLSSCHHLKFNILILLQYSICRFIYVPAPHTAEVICEHLYEALVEWNLDEKISTLTLDNCTTNDKVISELIKKIGKRKLMLEGKLLHMRCAAHILNLIVRDGLDVIKDSIAKVRESVAFWTATPKRVEKFEEIAKHVKVKMENKLGLDCKTRWNSTYRMLSIALPCARAFDRATRVEKLFDCAPSEEEWAFASEVVDRLKLFNDITAVFSGTNYVTSNIQLLKICEAKEQIRKWAVCGDSTIEEMSVEMIQKFDKYWKDIQGPMGLATILDPRFKIDYLLGFIETITGQSSEECATKVAEVKDTLYDLMKEYEVEDDEDNTESSAPPLVNSDLLSSITARVTSRRPAAIRVKSELDRYLEDELVSINTENFKILDWWKVAGTSFPTLRKVARDIFAIPVSTVASESAFSTSGRVLSEHRSRLTPELLEALMCSQDWLRNKYRGTYDVLFIHHAHRFYNAMDAKLLLLSCSRSSKLLELPSRYPRSDGILSTTNQSNSYCHYLLYPYKILDLNIFCYFFNLGTCSCLKTMYGHALFLQFWMFVTPVMCHDKLVNHGNAFGYLAQKTYSELWFYVCMDNMCCCCNVVITNELMNDESCIIVLLLNLGIWILYSWAHFCCTACCCTAVVSDELMMCICELMMCICEFVIICELVFLLLFMCNWLELLFVLIFYCLIEMLHVDCVSRGWRLHGDKLPARRRWGKMLPRCISRWWGKFPTAEAGMVEPPTGNSPLPSL

>Os_HATORY

MSGTYRRKLLVFFLVKGHRGEDIGKSLENCLAEWGIDKVFTITVDNASANNNAIKYMRRVLNESKGCVAEGEYIHMRCAAHIINLIVGDGLKEIGTSIQRVRAAVKFIRCGTSRLVKFKKCAELAKVQSKAFLNLDICTRWNSTYLMLNAAEKYQKAFERYSDEDPYYKLELEGENGPGVPTRADWEKARKMADFLEHFYDLTLRVSVQSRTTSHTYFHEIADVLLLLREWSHSEDKLSKEMGTRMLMKYYKYWGEKYGERQGDREKRGEKDKGDQLLNFTVFFCVAIDPRYKLSNCIRMGIKVMFGDTVGEKVWETVNTYFRALFEEYKEMYTPKDKAPQPTESESTAETSKRVSCRWMSVITQQLNSEGGSGTIKSEVDKYLSEDNEPDTPKFDILKWWKANSTRFPILSHLARDLLAIPITSVASESAFSAGGRTLDDFRTSLTPRMVERLVCANDWLRGGNYVSVEEDSEQMALLEEGNIENFYFLPNSNVLHDSYTIDCSN

>Os_HATOS1

MKKKSIDLKTLWDRHSKSRKVGLGSGSGSTTQPVRIDSEVAVPSVQPITDANESPVQIQITAVEVVPEVAASDAARDVTEPETEVASNDPDIATAVTQQEQPSTWSPIRDWSPIRDGDDEETEYDSSDEAIYDIDLLCHDPGRRIAIKNYDVNERNSVIRGYIALGPCQPRSHNFPIRKIGGKPRRFLPSWYDEFKWLEYSVEQDAAFCFICYLFKHKINNSGGDAFVNRGFRNWHMRKRIAKHVGGMTSFHNVAQDKYNHFLAPKTNIVESFAATNEQDKARYMARLTYSMKCLKFLLRQGLAARGHYESEKSLNKGNFLEMLSMLAENFEEVGKVVLNNAPKNCKLTAPEIQKQIANCCAKETTKLIMEDLGDEYFAILADESSDVYQKEQLALCLRYVDKKGRVVERFLGVVHVENTTSLTLKAAIESLLMEHSLSLSKVRGQGYDGASNMKGHANGLKKLIMDECPSAYYVHCFAHQLQLTLVAVAKENPDCVWFFEQLRFLLNLLGNSCKKTEMLRVAQAQRIVEELDLGDIETGKGLNQEMGLGRPADTRWGSHYKTVMHVLSLYPSIQKVLIMVGKDRSFGAECANAQTVLTIFQSFEFVFMAHLMQTVLGFTSDLNHALQKRDQDIVNAVGLILLTKFQLQQLREDPGWDDFLQEVQSFCVKHKIKIPDMDSFYRPVGRDRRFFIKIKNMHRFHVDMFLSVIDRQLQELNERFDEVNTDLLLCMAAFSPIDNFASWDKDKLIKLARFYPNDFSSTEMNHLPSALKLFLTEMRIDERFRKVKNLANLSIMIVETKLHNRHEIVYKLLKLVLVLPVATASVERIFSAMNYVKNKLRNRMGDQYLNDCLVTFIEREMFLKVKECDIINRFQAMKERRIKATLPSHEGTEIEN

>Os_TEMPINDAS

MCPRPWYVSKFHNHLIYTTVNFAFSFEIEFVQEMDMVAISDGGEDEENSFDLNDEMSVSLPSSRSSSEGEDGDDTSKTQKRGAKRPKAGISLSPSKVKITRGKRASCWKYYKVINVPSKKEKGKMECKAKCRFCHHNYAYRPGGTTTTLNRHLDKCTIYLNKLAKAKAQGTLDFPLADGSMVVHPTEYDHDHTKLLIARMIILHDYPFRIVEHKGFNALMKWMNPSYEFIGRKAIKSECMKLYESEKEHLRKSLREAETISLTTDMWTSNQNLQYMCLVAHYIDVNWVLQCRVLNFVEVEPPHTGIVIAQAIFDCLVDWKIEDKVMTITLDNASNNDTAVSNLKSKLAARKNAQFDPDYFHVRCAAHIVNLVVNDGLQQIQSLITNVRNTVKYFKKSPARMYKFVGVCNTYSIKVGRGLSIDVKTRWSSTYRMLETCIEYRNGFDYYAESDTKYEWLPLQSEWDLFEKIQPILGTMSGATTAFSGSTYPTANVFYPYIAKVKIAILASRAQAQTALLEAARLGQQSGLYEPDPNDVLLVTMADAMLEKFNKYWENTNNIMIIATILDPRFKMRYIRWCFSEFFGETRCVTEVAAITDEMEKLYRKYERICRHNQGGNSPHNGHSASSSISTTTSLASIIPSGFQSFLQSNAKESSKSELLIYLDEPNVSLEDSTFNLLNYWKVNAHRFPVVSNMAKRFLAVPASSVSSESTFSTGGRILDDYRSSLKPETVQALVCASSWIRASQNDNSAPIPVVCNAMIIFYSLFLSSLLYKMFTLDCFHREKMEMMTSK

>Os_TWIFB1

MDESNIPSFTLGDFDPNYVSRSFPTGEYDATGSAPTPPVMEPPAGSEASGTMSGSASTNTGSKRSRTSGVWQHFDEVAMTGPDGRQVTFARCRICKNKLSAKSSGGTGHLKRHAEACAKKQGIQLRQQQLLLNPDGTVRTWEYDPMVARENLARLIARQDLPLNFGESPAFENYIKNSHNPRFQAVSRQTTTRDLKNVYDKGYESLKELFSTCTFSVSVTSDIWSSRAKEDYLSVVVHFIDDDWQMQKRVLGLRLIDVSHTGENIAERIREVIDEFNLADKIFAVTMDNASANSRAMEILQPLFCIYAQSFLLHQRCACHIINLIVKCGFKRVNVHIDAVRQAITWLTASNPRIAQWKRYCCASGEPPRKFLTDADHRWNATYFMLKVVLPYKDLLTVFLQTRNGPKNSDGQPILTDHTWHIVERFNQFLETFHDCTLLLSQVYYPTANLILHNILEIATLLKEYENDDLLMPVVFNMKQKYLKYWKDIPMLYSFAFILDPRGKLRGFLNILSLIGDIINVDYSTYYADVKTKFYEVFRKYELKFQGDRLQRPPPVPAAGKKKLQWSRIWGGSSSSHGGGTSSSAASGDARSHGPAEELSNYLDSDAIRHETSDFNVLGWWNDHKMSYPVLSKLARDVLTVPVSSVSSESAFSLCGRIIEDRRTSLSSDHVEILLSVKDWELAAEHAQYTADNQELAAQFENLYLDDEQLG

>Os_hAT-1

MRRSKKRQHESTTGPSQRSVDARQLSPGMDDYGMDDRHMDGLEGDDDDQPSPGEANFSVDADATTLPTPNSDNQPSARRAECWRHFEVFTEMVDGKSIPRAQCKYCDKILSAATSSGTGHLNRHYLAHLKNKAPAGARQTQLSFGPDGSVSTWTYDPKIAREEIAKFIVAEDLPIRMGESKHFERMIQKAFCPQYKKVSRKTTKNDITAIYRSKLSVLKQTFSTTSFSFAVTSDIWTSQHQRTSYLSVVLHYLDNNRSLNKRVIGFKLMTSHTGDAIATTILEVLREFNLQSRVVSITLDNASANTTAMSILEPDLRSYVGGFVIHQRCICHIINLIVQPGTNVINNLLILVYAAYCLFILMYATYLFCRHDCSGCMRPICFAGMIVLDKLLNKIRRSVRIIGGNTVVKARFQDYCKAKKKPGRMFGIDVKHRWNTTYLLLRQLKGYEELISVFINSMHVRMKDTDDDGDGEILILTDQDWEIATRVRKFLKPFYNATVQLSGIYYPTSCLVLEWIWKLALVFDENRSDRILSSIVKPMEEKFLKYFTAIPHLYCFALILDPRKKLEIAKVAMNSIGDAVGLDYSEAFQHVNDELYRVFRLYRTKLGGTPRVPEQTSQKKASKSSAVNLWKQYIGNDQASPSSENKSTWNPDSELNHYLVTNHTEHDPTLDGDDVDLLGWWKEKERTLPVLAHFARDILLVPASSVSSEQAFSVTGRIIEERRSCLTPETVESIFCLKDWMEADERTQHRLXDQELADAVEDALAEICLTKDDGGVDPE

>Os_hAT-2

MDDDPTSVNYELRTMGMRGDDDDDVEEDRVEVFGNTSDIPIHVNVDDDDPPVDDSGNGTPTGSSATCTNKKTKTSKVWDDFEELYETTNGNRVRVSAKCNYCHKTLSARSSAGTGHLLRHIKSCKPRKLGSNALPQSMLRFSADGSVIPWEYSPEVARFELCRLIAREDLPISFGQSPAFVNYIKAAHNPRFVPVSRQTTTRDFYKLFKDRRSVIIDRLNSASSIALTSDIWSGHAKEDYLSVVAHFVNSDWQLEKRVLGLRLIDESHTGANIAERVIAVAEEYGITDKVFSITLDNASANSKAMDTLTPALSGYIGDLFLHQRCACHIINLIVKAGLDKFKPMLNDIRAAISFLNASNQRIATYKNVCIAAGYRPRMFGLDMDVRWNSTYLMLKHLIPHREPFTVFISTQHPFVNDHPLLTDLHWACAESVLCFLEQFYDSTVVLSGVYYPTSPLIMHHILEIAGHLNTYGNVQNLANVVGPMKTKFMNYWSKIPILYSFAFILDPRAKIRGFSKVLQIMAQLIGDDYSAYLTTVRASLSDTFAKYERKFGSVRLHSSTIPGPSTGKKRTAWGKIFGSVVAAGLGAGNAGASPGAGNAGASPGAGLGAGNAGASPGAGLGAGSLSRMTSATALLQAASSTANLNSSELSAYLDSDTVNQYDDDFNILSWWQQHKLTYPVLSILAKDVMTVPVSTISSESTFSLTGRIIEDRRRRLNPRLVEILAVIKDWELADAKSQHTTENVELQNAYENMYLDDEIDVNP

>Os_hAT-3

MWAKFAFCIGNPSSAASVSVEAGPKQAGINSDDPAWAHCFCPDITKKHHLRCKYCDKVCTAGITRIKYHLAGIKGFNTTKCQKVPSPVQQEMFDLLTKKTSEKEQKNKEKEVARAEVDIENSDCESGSEGSDHGNNVLVVKPKETTGSSSSRSVAGGHTIDKYYKPPSIEESASMTQRVIKLSNKVQTALTTQKREERRNRTCEYICQWFYEASIPHNTVTLPSFAHMLEAIGQFGRSLKGPSPYEMSGSFLQKRKEKVMDGFKEHKESWELTGCSIMTDAWTDRKGRGVMNLVVHSAHGVLFLDSVECSGDRKDGKYIFELVDRYIEEIGEQHVVQVVTDNASVNTTAASLLTAKRPSIFWNGCAAHCLDLMLEDIGKLGPVEETIANARQVTVFLYAHTRVLDLMRKFLNRDLVRSGVTRFATAYLNLKSLLDNKKRVSKTITTKKELVRLFKSDEMEQLGYLKQAKGKKASKVIRSETFWKNVDIAVNYFEPLANVLRRMDSDVPSMGFFHGLMLEAKKEISQRFDNDKSRFIEVWDIIDKRWDNKLKTPLHLAGYYLNPYYYYPNKQEIESDGSFRAGVISCIDKLVDDEDIQDKIIEELNLYQDQHGSFGHEIAVRQRKNKNFNPGSDLNM

>Os_hAT-4

MSDSRDPVWEHGENIPPGWRCKYCHTKRGGGGATRLKQHLAARGKGVTYCNSVPPDVREFFCRELDRIKDAGDQRKSDSGRRVEAARVNYYDLTGDADEEEQMEAAIAASRQDENFRRDVEERGGTYEHGGGSGSAQPEARKGRSNPITNMLRRATSHRESPAVRDYNLASAKAPVQPRIDTGFFTKKGKQARQAIGESWARFFFTAGIPGRNADNPYFVSAVRETQKWGTYASIKELLFFLIAHYLFHTSIVGESVPSPTGNEIDGKYLDSTEKDVKKQFDRFKKDWDEYGVTIMCDSWTGPTSMSVINFLIYCNGIMFFHKSIDATGQSQDANFVLKVCMVHATNYFITCYRQCFLLCYLPMQEIRKVVREIGSEHVVQIITDNGSNYKKACRLLRQEYKTIVWQPCVAHTVNLMLKEVGKMPDHEMVIESARKICRWLYNHNKLHAMMVLAIGGELVKWNATRFGTNYMFLQSFLRKRDLFMQWMASSVFMQSKFSGTLEGRYAHACLSSLSWWENLEAVVNSVQPMYSFLRFADEDKNPNLSEVLLRYQLLKMEYDSLFANQRDKFEAYMEIVNRRMHDLTNETLINAGKYWSPVPCFVAAALNPRTHYAYSPSATVFQDLRQAFEWMTDIDTAAAALLEVEMYRRKTGEFGRALARRMAIDGKTSPGMFLIMELCIQNYEMTCYLAFAAQWWSMFASDTPNLKKLALRLVGQCCSSSGCERNWSTFAFVHTKVRNRLTHKKLNKLVYVNYNLRLRIQQANAQIRVEDDDPLQRLADLSFYETNNHISAWMDNARSNACPELDEDSAESDAPLPSQLVSDLVNLDDLRRTTGASSIAEWADTNVGDTHIGKRKTRKPPKARPSKKVKGKGPRSTSVDSDEETQGSPEYQESNDSSSRTETDDGDDDGQGGQGTTNVPPRGHTQQSDHHSPVQFTGVIGSHPTIRSPQPRSIHWCDIIQLQFQPSSSNSVLDNFLQFFDLQVRATSLMLLRTRIMVLQALNELPLHRESDNSNNSVTYNRIALAHSVPLALKVAIQRMCTTVPKLLIILLLHGCMNGKSLSGMLNCTLSGKQHHHGQVKVGRNTRRGCYIHMA

>Os_hAT-5

MKKSEDGTEGAVETYRYDETVSLKKFYMAIIMHEYPFNIVEHDYFVDFIKSLRPTFPIKSRITVRKDILNIYEEEKKKLWQHLLTLSCRFSTTMDMWTSNQNKSYMCITVHWIDDTWKMQKRIIKFMHVEGHHSGSNMCKEFYDSIVDWNLDRRLVGLTLDNASSNDVCVKGVILKLRKISPLICDGIFFHVRCFNHILNLVAQDGLKQITGAVLKIRNTISLNSKALATAVILKHSPLQFEASIQKCALEVSLDNTKGLSMDTPTRWNSTFLMLKNAIYYRNAFDRLFLRHGRKYAKCAPTKVDWSMAIALCKCLKPFHEATELFSGTTYPTANHFFGKFCSIKLYIADWCNSTDGTIKTMANAMQTKYDKYWEKSNMALAVACFLDPRYKTSSIEYYGMKIYGLEAAEKFDEFNGVIKKLFDVYALTSACATSKKKGAEMHVHQLQIQSDPVHNTDEFDDIFNENDSSHDHEQHFQRFLLERSQPICSDKTELQIYMEQPLLLWTSKDPFDILSWWKLKQAEFPILCKLARDFLMYSSVNCCF

>Os_hAT-6

MDSSPTGKRPTGQHNRQQPAHIQKAHQTKCQAQTLAFTSQFRSGQNAGRQTARQHRAARRMRASRLQHRRRAFAVRRPPSGYAATPTDHRPLSHVAAWVCDKPTTVRLPPRRPPPQGPAWPLASFGSSTPRAEGRRATSSSSWVVKSTLSDSSSHPGASPAGTVAVGTGHSALAPNALVLNDATTATGGGGGSSEPVVIPDEEDGEFTGEVEPVAKRQKKCTSKVWDYFIKYTDKAKNKVTGVEEQQRQQELQVKGEVQTFRYDPEKIYPDKYEEEFKRVLAAIDKFFRAYKSCVARSSKPTAAGSSENSQPHGNTSLGHNEIEKFLYDDAAANKEDDINELDVYMKEKPIRWVDPTGEGVEFDILAWWKNNQMTFPILSTLARDVMAVQISTVASESAFSAGGRVVGPFRSSLHPEMIEALVCTKDWIRASRKGPFKDKIATILDELDVQIEDKDGAKTDNDEERVKEHHIVLTECYVLFRVRQRIVFEAEHVADWRAGVFGD

>Os_hAT-7

SPWVSLQNKPNYPHSTPNPTLPARACARCAAAHCRRRRHNRAPSPPRAVTAVRCHRRAPSPPFAATVAGLTQEGRWQICGCVTSILDLGELGGLFLEVVLGSILIMWVGSIGDAEEWWVSVGFNAAAGLLNMSMQNSESSSANSSSHELPSQTLRKQPVENFVFDPKKTEELMIKYFIHAEIPFHKIEDPYLDDWMASMQPTFKLVGRQSIRDKIYNYYNRLKQELHAELENLDSRVCLTSDMWTSNQNLGYMVVTAHYVDAEFKMKKKIISLKPVKYPHTSFAIEEAMMRCLTEWGLSSKLFTLTLDNASNNTAACQELVKTLKDELVLEGKHFHVRCCAHILNLLVQDGMRVIRAAIDKIREILKYIEHSPSRIQAFNSIASSKSLPPKSGFTLDVPTRWNSTFKMIREILPYKAILNSYASENCELLPTDEEWLHAESICEFLKAFEEATRGVSAHRTPTAHTFLPHVLCIRHALSDPDWQTSDLLKRLAAPMHTKFAKYWDEKLANNFNLALVITTVLDPRRKRDYLDFFYEKVSPHGSNVESKVDSIIEEMKSYFHVYEGIARRRGVSYMSQSSERVSVVGSPVLGKRKLEHEFTLFKSNRKVARTQKSEIDTYLEEVCEDDSEDFDVLAWWKKNSKKFPVLAIMARDFLAIPLSTVPSESAFSSGGRILGDTRSSLTPEMLEALVCAKDWLHRAKKQGD

>Os_hAT-8

MERFYKRKVPEPNSANNACNSCLDDINWEDEIKYDPGLRKQIDEYHPNLRERVRRKYLENGPCQPRTFAFPMTGSRRFVPEWFDEFGSWLEYSESKNRAYCFFYFLFREKKDGGYEAFVKNGWNGFHRKERLKLHVGDVGGSHYQAMKKCDDLLQKRQHIDVAFHSVRETGKRDYLTRLNGSIDVARMLVKLGLPFRGHDESKESYNRGNFREFRDYTAEQNPSLRKAIGTKKSDNSLLVAPEIQRDIVKCFAKEVLHAILEEIGHDVFCLLVDESRDVSCKEQMAVVLRYVDKYGIVRERFVGLVHVTETTSAYLKSSIDALFAELKLSLKQVRGQGYDGASNMRGEFNGLQSLIMRENSSAYYVHCFAHQLQLVLVAIVRKHKGVSDFFTKISILLNVVGGSSKRRDLIRDINVKEMSKALGCGQLQTGTGLNQEQCLQRPGDTRWSSHYKTLKSLVGMFATIVKVLEIVEKDKNDWKIRDQASNLLEYFQSFDFVFYLHLMLTILTITNSLSLALQRKDQDIVNAMKCVKSTRLNLDELRREKWEKVLDEVSDFCDKYDIVKLEMEDTYIDPKKHRHKSGITNKHYYQVDCFNDVIDWILQELDNRFSETSSQLLICSSAFSPRDSFHDFNLENLMSLAKLYPSDFNSGNLRDLSHQLGLYIADVRDDGRFSNIQTIAELSQIMVETRKHLCYPLVYQLLKLVLVLPVATATVERCFSAMKNVKTYLRNKIGDEYLSDSLICYVEKEEMKKVTNEAVVRRFMKMQGRRFDDD

>Os_hAT-9

MKRNGDIASLFRKHEAKAKKRADLVDEEAQIEEEEPPLVEPQPEPTLVVEATNEEQDAPPEYDADHLQYDPGLRSPIASYDVNEQDAVQRAYILKGPNQCYPHDFPVREIYGKKRHFNFVWFHKYQWLEYSVAKDAGFCMVCYLFGXGTSNFVKDGWRNWNKADALDKHVGGITSAHNKAQEKYNLFVSGGPSIDNVIVKVSNESEIRYKARLTYSLRCLRYLLNQGLAFRGHDESEESSNRGNFIELLKWLAENNKEVDRLVLKNAPGNCILTCSSIQREIIHCCADETTKRIIEELGDDHYAILADECSDLSHKEQLALCVRYIDKLGRVCERFLGVVHVASTTSAALKKAILTLLSDHHLTPSQIRGQGYDGASNMKGRLMGWAENIDHERVSFAYYIHCFAHQLQLVLVSVAKGNDDCVWFFTQVSHLLNIVGTSCKRHDMLRDVRAQKIMEALELGEIESGVGLNQEMGLARPGDTRWGSHYKTILHIIGMYPTIHEVLITLGKDPTQRDDWPRIHAVVGAFESFDFVFSAHLMLVILGYTNELCLCLQKRDQDIVNAMSLVTLAKERMQKLRSEGWEEFFQGTVVSFCNKHSIQVPTLDGKYVPHGRSPRFYPDQTNDDHFRREVYIGVIDKISQELNSRFDEVNMELLICMSALNPFNSFASYDAQQVLKLAKFYPKDFSPMDLIRLELQLGTFIDDMRKDERFKGLETLAELSIKLVETNKHVLYDWVYLLLKLVLILPVATASVERVFSALSVVKSKLRNSMCDKLLNDCLITFIERDVFSQVSEEDIIKNFMSMKTRRVEKK

>Os_hAT-10

MWKEFDPIRTNDKLSHAKCIHCNKVFVASRSSGTSQCLRHLKVCKVRLRMHHLIEHMHANLSPTADVMKNWKFDQEVSRKELLRMIVLQELPFSIVEHVGFRRFVASLNPYFKVISRTTLRNDCMAAYEDHKLALFDVLKSSNSRVSLTADMWTSIQNLGYLCVTCHYIDNEWKLQKRIIKFALVPTPHDGITMFSEMLKAIQEWHIENKLFSVTLDNASVNDTMMTHLKTNLVGKTMLPCDGVLLHFRCAAHIFNLIVQDGLKTMSNAINSIRESVKYVRSSQSRGQRFEEMIAQVGIKTNRRPSLDVSTRWNSTYLMLESSLLVRMAFEALDRHDINYLHQPFDYQWTMAEKLCALLKVFYEATVAVSGTLYPTSTCYFHELWKIKMVLDKEATNEDVTIASIVKEMKEKFKKYWDAQYLQICFPVIFDPRYKYKFIEFRLKSAFGAAATPYLKEIKSNMQKLFDEYSAKYGGSNNINSQPETSVEQNVDASNQFADWRQFLHDKSRSKVKSELSRYLADMPQEGDFQDGHDFDILNWWMVNKTKYPVISRMARDVLAIPATSVASEAAFSTGERIISDYRSRLSSSTVEALICLQDWMRAEGLGDFFARDLAESDDQNVQHSGI

>Os_hAT-11

MAEETGNDNQVVQGNEIVPSNEEAQAEEVQGDELVPAEDLTQGDEVQGNELVSAEISTPPTLRRRRKKSLVWEHFTIEAVSGGATRACCKLCKQTFAYSSGSKIAGTSHLKRHITLGSCPKIKNQEHKLALTPAGGTDNDGEGTVERPSKRRYRYTGYANAAFDQDRSCSYLAKMIILHDYPLHIVQQPAFTTFIDSLQPRFRVVDVETMEGEVYAVYQKEKENLTQAFSTMPGRISLTIGLWTTSQTLGYVSLSGQFIDSEWKIHRRMLNFMMVSSPHSENALSEAISASLSDWNMKDKLFTITLDNDCSSHDIYSANLRDYLSNKNNLMLKGQLFVVRCYAHILNAVAQDVIASIHGVIYNIRESIKFIKASPTREEKFAEIALQLEIPSTKTLCLDVTTQWNTTYLMLLAALDYKQAFSTLETSDDNYNEAPSAEDWKKVEAACNYLKLLYDSAHSIMAAANPTSNLFFHEAWKLQLELSNATGHEDPVFSSIAKDMHERFDKYWKDCNLVLAIAVVMDPRFKMKLVEFSYSKIYGVEAAKYVKVVDDAVHELYKEYVAQPLPLTPAYVEQGEGNNAPASENSTQTTAPSTGDGLVDFDMYLSEIATSQPTKSELEQYLDESLTPRIQEFDILNWWKLNTLKFPTLSRMARDILAIPMSMVSSGNSIFSAGTGTRMLDDYRSSLRPEIVEALVCAKDWLQYLPATPEAPSTTLVKVDAP

>Os_hAT-12

MTKMTWRRNACRCSESLRHLLTSMGRSRRRSRTWTVPVAVTVMVAQARRPAPTPVLPTSGQDHPRYGMTSKNSLNHAMARRFAFLLNAITAKKTLSAHSSGGTGHLLRHLKSCKPRTASNLSQSMLKFNADGTVRPWEYDPDYARTELVKLIAVEDLPLNFGQSPAFEEYIQNAHNPRFXAVSRQTISRDVFKYFDKSRAMLIERFKSVNSVALTSDIWSGNAKEDYLSVVAHFVNSDWQLEKRILGLVLIDVKHTAENISERVLSVVEEYGLTDKVFSITLDNASSNTKAMDFLKPKLSAYVGDLYLHQRCACHIINLIVKAGLEVFKPMLQDFRTAISFVNASNQRIALYKNWCIAKGVRPRKFGLDMDVRWNATYLMLKHLFPHKELFSLFIETHYPRENGRLLLTDLHWTIAETVLLFLEQFYDSTVILSGVYYPTSPLIMHHILEIAGHLNTYENDXNFRNVVVPMKSKFLAYWSEIPFLYSFAFILDPRAKIRGFSNVLQIMSQILTSDYSTYLTEVRAALSDIFSKYESKFGAVRLQRTTPGSTAGKKKIAWGKIFGASDALGHGAGASPGSGLGAGLGASASPGSGLGAGPFSRRTSATALIQAVSSNANLNASELSAYLDSDTVNQFDDDFNILNWWHEHKHTYPVLSILARDVLTVPVSTISSESAFSLTGRIIEERRRRLGPDMVQALALIKDWVQADKKLQHTAENVELIKSFENLCLDDVTSGSATGTG

>Os_hAT-12

BMDGDPTSCNYEARVMGTQGDDEDDLEEERIEVFGNTASPLRDLSQPEPHDDGTGADGNGAPSGSSASNKRSRSEVWDDFEELFEERNGAQVRVSAKCNYCHKTLSARSTGGTGHLLRHIKSCKPRNVGALSQSMLRFNADGSVSQWEYKPDVARTELVRLIAREDLPLTFGQSAAFEEYIQNAHNPRFSVVSRQTISRDVFKVFDKRRAMLIDTLKSVSSVALTSDIWSGNAKEDYLSVVAHFVNSNWQLEKRILGLVLIDVSHNAENISERVLSVVQEYGLTNKIFSITLDNASANSKAMDSLKPALSGYIGDLYLHQRCACHIINLIVKAGLEIFKPMLQDFRTAISFINASNQRIALYKNFCIAKGIRPRKFGLDMDVRWNATYLMLKHLLPHRVIFSVFIASHHPMADGQPLLTDLHWTIAETVLLFLEQFYDSTVILSGVYYPTSPLIMHHILEIAGHLNTYENDWNLRNVVVPMKNKFLSYWSEIPFLYSFAFILDPRAKIRGFSNVLQIMGQLISGDYSAYLNEVRAALSDMYAKYESKFGAVRLQRATPSSSSGKKKTAWGKIFGAAACGSSLGAGLGAGNAGASASPGSGFGAGASPGSGFGASASPGSGLGDAGASAGSGLGAGAFSRRTSATALIQAVSSTANMNASELFAYLDSDTVNQYDDDFNILNWWHEHNHTYPILSILARDVLTVPVSTISSESAFSLTGRIIEERRRRLAPDMVQALALIKDWEQADAKMQHTMENLELINSFDNLFLDEVTTATGTG

>Os_hAT-13

ACESSNTSFNKEIQIEAWKEFVPILIDNEVGAGKCKHCDTEIRAKRGAGTSSLRKHLTRCKKRISALKIVGNLDFTLMSPNSVRLKNWSFDPEVSRKELMRMIVLHELPFQFVEYDGFRSFAASLNPYFKIISRTTIRNDCIAAFKEQKLAMKDMFKGANCRFSLTADMWTSNQTMGYMCVTCHFIDTDWRVQKRIIKFFGVKTPHTGVQMFNAMLSCIQDWNIADKIFSVTLDMLQPMIQWLSC

>Os_hAT-14

MLFVDRMSNPAATVESQVALTPSSTSTPTPTNAPSVEINDSQGTEVEHDNKRLKSAAWQDFVKKKINGAWKAECKWCHNKLGAESRNGTKHLLDHIKTCKSRXARKGLTQSNLKMGIDAEGRVTVGKYVFDQEVARKELALMICLHEYPLSIVDHVGFRRFCGALQPLFKVMTRNTIRKDIIDLFGVNKISISNYFHKLQSRVAITTDLWTATHQKKGYMAITAHFIDDEWKLKSFLLRYIEYVLHIMLYHASYICLTLYLYTNVLLWCFMFCRFIYVPAPHTADLISEIIYEVLADWNLESRLSTITLDNCSTNDKLMENLLGTMLDKLPADTLMLNGSLLHMRCCAHILNLIVKDGMTILDKIIEKVRESVSFWTATPKRHEKFEKQAQQINVKYEKVIALDCKTRWNSTYLMLSTAVLYQDVFTKLGTREKMYTPYCPSNDDWKFARELCDRLKIFYDATEAFSGSKYCTANLFFPKACGIYLAMRKWSTSADPNIITMTKLMSAKYEKYWKDVHGILAIASVLDPRYKLHMLNAMFIQIYGEEVALRKVNAVKEDLYKLVLQYQNHVEEGVGTSDGVNASSSVAPPGGFDLVDDIFDQYMSGQTVASSSQIRTELDLYLEEKPLPRTQDFDIINWWKFGGIRYPTLRQIARDILAIPITTVASESAFSTGGRVITPNRNQLKPDLVEALMCVQAWGRADMLGNFIFAFVSFKFNLSFHVSSISSNCKSHYMLIAEIANKTNALNTVLDDESESVCPTLPLYCFYYYNIQCLVASSNFDFRKHLLSLKLEVEIS

>Os_hAT-15

MSKRTLLTYYSSSSNTDPSPSTENLSQPKRPRAEFSQSDIIGDPGLRKPIEAYPPEIRDQVKRAYALSGPTQPNITIFPRKWQGGEWRSFQKTWFNEFDWLEYSVSKDAAYCLYCYIFFEPGKPEKFGSAVFAKEGYVNWKKGKDRLTVHSNCKTHNDARNKCEDFMNQRTSVSKKIEIVSKEEEIRYKIRLTSSLDVVRFLIEQGDAFRGHDESDTSLSKGKFKEMVDWYKDKVPEVKDAYEKGLKNCQMVSHHIQKDLTKACAEKVTAVIMDEIGNRNFSVLIDESRDVSIKEQMGVILRLVVHICSFICLIFQILFLLLCRFVNDEGKVMERFLGLQHIERCTAIALKEALFGMISSHKLTISKIRGQGYDGASNMRGEFNGVQKLIRDENPYAFYVHCFAHQLQLVVVAVSTSTPAIADFFNYVPLIVNTVGASCMRKDALLAKHHDVLLEKVENGEITTGRGLNQESSLARPGDTRWGSHLKTLLRILVMWEAIIDVLEIVKKDSTKPTFNGGAFGLMGKMQSFDFVFIMHLMIDMLSITDDLSRALQRKDQDIVEAMSLLIDVKELLQDMRENGWEPLLNRVISFCNKHEIKVPKMDKEVNERGTSTHRRHKVTNKHYYHVEIYLAAIDAILVEMNHRFSEVSSELLVCMSSLNPRNSFSNFDVDKLVRLAEIYAEDFLVGDLMLLRTQLGNFISNVRRSKEFLGCKDLAKVAELMVQTGKNRTYHLVYRLIELSLILPVATASVERVFSAMSLIKTDLRNKMGDEWLNDLMICYTEKQIFRSISDEKIIQHFEEMKKRRMLVPQQKLVVCSMPPFSP

>Os_hAT-16

MSVNLQRQLSIEIESPSNRDGIAATEAQPNQQLVPGEVRGEEGQEGNPTKATRKKKSPLWDFFEECTVPSKKKKGEMENKVKCKACETLLTKNSSGTTTHWRRHLEQCDYHKLQQKSIKQQNINFPSLDEGDVDLDAPCVSVPGYYDPTKIRELICKMIIVHELPFCLVEYTWFNVLLKRLNPSYKKVSRNTIRSDCMRLYESEKEKLKRTFKDVRKISLTCDLWTSNQTICYMSLVAHYIDADWSMHCRVINFLELEPPHTGVVIANAISDCLASWRIEDKIASITFDNASSNDSAANLLLAKFTKRGSLWFYGKFLHIRCCAHILNLIVQDGLAVIKHIIDKVRDTIKYIKKSNNRAYKFSADIDSLNLKSDMGLAIDSCTRWGSTFKMLQSAFFYRSALDVYAAGDANYRWLPTPEEWNLYCEVNDALSVIHAATEEFSGSTYPTSNLFYSHIVDIKRVLGQLLKSKDPILKXMANAMLEKFEKYWGPECNTLFAVALVLDPRFKMGMINYTFPALYEETVLPKKLANVESTLKSLHASYESELQSTSKENDATTQSTSTSLGTTSSHFSAASQFHEYMKSKNAASLPKSDLKRYLDDPVEDIPAKSFNLLQWWRMNELKYPIVAKLAKDILTIPITSVSSESAFSTGGRVISDYRSSLLPSTVQALVCTSSWIRGGHHKSTDLVSSYIFMFLTN

>Os_hAT-18

MDLKSMWGRAEKKLKSIVDSNNISEHTAERKDPEPATIHASATEEIGCSRACEVGFAETKTQVPYESIEVSRPQDVDGSIEVSKPHVANSESEEEDDWLEDVDEDVGFLPHDPGKRMAISDYSVNQQDKVRMKYIALGPCRPPLEQFPPRNCGGKRRFIRSWFDKYSWLEYSEEKDAAFCFVCYLFKDRANYVGGDSFVNEGFRQWNHLDRFDKHVGSVSSTHNLAQEKYDMFIRPNTAIGENLRKRSKHDKVVYKARLTYSLKCLRFLLRQGLACRGHNESEESLNRGNFLELLNWLARNFKEVNEVVLKNAPKNCKMTSPRIQKQLIRCCATETTKLLMEDLGDEFFSILADESSDVYQNEQLALCLRYVDKKGRVVERFLGVVHVENTTALVLRDAIKNLLMQQSLSLSKVRGQGYDGASNMKGEVNGLKKLIMNESPSAYYVHCFAHQLQLTLVAVARENGDCVWFFEQLGYLLNTIGISCKKHQMLRVAQAQEIVEALELGEIESGQGLNQEMGLGRPGDTCWGSHYKTVQHILIMYRSIRKVLLQVGKDRSQSAEAIKAQTAFQSFVSFEFVFMAHLLNTILGYTDDLNTALQRREQDIVNAVELIVLTKMQLEMLRQDDGWENFLKEVTSFCMKNEVKVPQMDARYKPVGRSPRFFGKVQNLHRFKIEMFLSVIDRQLRELNDRFDEVNTDLFVCMASFNPTNSFGSFDKEMLVKLAQFYPNDFSANDIMHLRTYSA
