# Supplementary figures and images for: In silico Phylogenetic Analysis of hAT Transposable Elements in Plants
Source: Genes (Basel). 2018 Jun 6;9(6):284. doi: 10.3390/genes9060284 (PMC6027215; doi:10.3390/genes9060284)

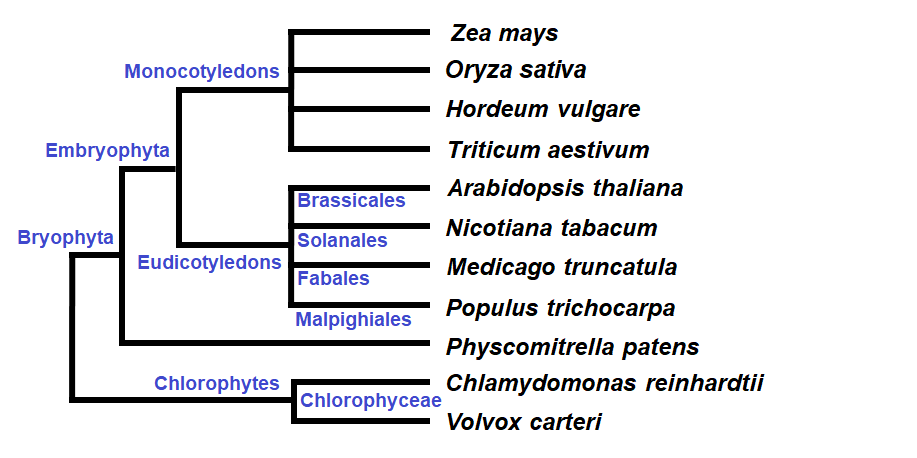

Supplement: Supplementary file 1 [file genes-09-00284-s001.zip › FigureS1.tif]

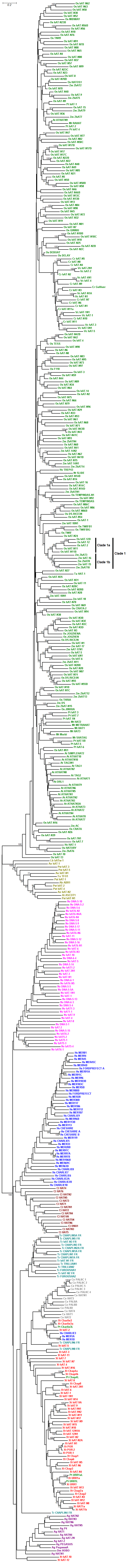

Supplement: Supplementary file 1 [file genes-09-00284-s001.zip › FigureS2.tif]
